# Supplementary material for: Epidemiology of hepatitis B virus and/or hepatitis C virus infections among people living with human immunodeficiency virus in Africa: A systematic review and meta-analysis
Source: PLoS One. 2022 May 31;17(5):e0269250. doi: 10.1371/journal.pone.0269250 (PMC9154112; doi:10.1371/journal.pone.0269250)
Supplement: S2 Table — (PDF) [file pone.0269250.s003.pdf]

S2 Table: Search strategy in PubMed

**Search**	**Virus**
#1 Condition	Hepatitis C OR Viral hepatitis C OR Hepatitis C virus OR HCV OR Hepatitis B OR Viral hepatitis B OR Hepatitis B virus OR HBV
#2 Population	HIV OR Human Immunodeficiency Virus OR Immunodeficiency Virus, Human OR Immunodeficiency Viruses, Human OR Virus, Human Immunodeficiency OR Viruses, Human Immunodeficiency OR Human Immunodeficiency Viruses OR HIV-1 OR HIV-2 OR Human T Cell Lymphotropic Virus Type III OR Human T-Cell Lymphotropic Virus Type III OR Human T-Cell Leukemia Virus Type III OR Human T Cell Leukemia Virus Type III OR LAV-HTLV-III OR Lymphadenopathy-Associated Virus OR Lymphadenopathy Associated Virus OR Lymphadenopathy-Associated Viruses OR Virus, Lymphadenopathy-Associated OR Viruses, Lymphadenopathy-Associated OR Human T Lymphotropic Virus Type III OR Human T-Lymphotropic Virus Type III OR AIDS Virus<br>AIDS Viruses OR Virus, AIDS OR Viruses, AIDS OR Acquired Immune Deficiency Syndrome Virus OR Acquired Immunodeficiency Syndrome Virus
#4 Context	Africa* OR Algeria OR Angola OR Benin OR Botswana OR "Burkina Faso" OR Burundi OR Cameroon OR "Canary Islands" OR "Cape Verde" OR "Central African Republic" OR Chad OR Comoros OR Congo OR "Democratic Republic of Congo" OR Djibouti OR Egypt OR "Equatorial Guinea" OR Eritrea OR Ethiopia OR Gabon OR Gambia OR Ghana OR Guinea OR "Guinea Bissau" OR "Ivory Coast" OR "Cote d'Ivoire" OR Jamahiriya OR Kenya OR Lesotho OR Liberia OR Libya OR Madagascar OR Malawi OR Mali OR Mauritania OR Mauritius OR Mayotte OR Morocco OR Mozambique OR Namibia OR Niger OR Nigeria OR Principe OR Reunion OR Rwanda OR "Sao Tome" OR Senegal OR Seychelles OR "Sierra Leone" OR Somalia OR "South Africa" OR “South Sudan” OR "St Helena" OR Sudan OR Swaziland OR Tanzania OR Togo OR Tunisia OR Uganda OR "Western Sahara" OR Zaire OR Zambia OR Zimbabwe OR "Central Africa" OR "Central African" OR "West Africa" OR "West African" OR "Western Africa" OR "Western African" OR "East Africa" OR "East African" OR "Eastern Africa" OR "Eastern African" OR "North Africa" OR "North African" OR "Northern Africa" OR "Northern African" OR "South African" OR "Southern Africa" OR "Southern African" OR "sub Saharan Africa" OR "sub Saharan African" OR "sub Saharan Africa" OR "sub Saharan African”
#3	#1 AND #2 AND #3
#4	Limit #3 in English and French
