# Supplementary material for: Epidemiology of hepatitis B virus and/or hepatitis C virus infections among people living with human immunodeficiency virus in Africa: A systematic review and meta-analysis
Source: PLoS One. 2022 May 31;17(5):e0269250. doi: 10.1371/journal.pone.0269250 (PMC9154112; doi:10.1371/journal.pone.0269250)
Supplement: S4 Table — (PDF) [file pone.0269250.s005.pdf]

S4 Table. Main reasons of exclusion of eligible studies

| N° | Author, Year | Title | Reason of exclusion |
|---|---|---|---|
| 1 | Abdalla, 2005 | Comparing walk-in and call-responsive donors in a national and a private hospital in Nairobi. | No data on HBV and/or HCV in PLHIV prevalence or case fatality rate |
| 2 | Abdelaziz, 2021 | Real-life experience of treating HCV co-infection among HIV-infected population in Egypt: single-center experience. | No data on HBV and/or HCV in PLHIV prevalence or case fatality rate |
| 3 | Abdulkerim, 2014 | Prevalence of hepatitis B and C virus infections among patients with chronic hepatitis at Bereka Medical Center, Southeast Ethiopia: a retrospective study. | No data on HBV and/or HCV in PLHIV prevalence or case fatality rate |
| 4 | Abou, 2009 | Seroprevalence of Hepatitis B virus and Hepatitis C virus among blood donors in Nyala, South Dar Fur, Sudan. | No data on HBV and/or HCV in PLHIV prevalence or case fatality rate |
| 5 | Adegoke, 2008 | Seroprevalence of hepatitis C virus infection in Nigerians with type 2 diabetes mellitus. | No data on HBV and/or HCV in PLHIV prevalence or case fatality rate |
| 6 | Adesina, 2010 | Human immuno-deficiency virus and hepatitis B virus coinfection in pregnancy at the University College Hospital, Ibadan. | Duplicates |
| 7 | Adeyemi, 2021 | Hepatitis B virus infection among men who have sex with men and transgender women living with or at risk for HIV: a cross sectional study in Abuja and Lagos, Nigeria. | No data on HBV and/or HCV in PLHIV prevalence or case fatality rate |
| 8 | Adjei, 2007 | Correlates of hepatitis C virus infection among incarcerated Ghanaians: a national multicentre study. | No data on HBV and/or HCV in PLHIV prevalence or case fatality rate |
| 9 | Adjei, 2008 | Correlates of HIV, HBV, HCV and syphilis infections among prison inmates and officers in Ghana: A national multicenter study. | No data on HBV and/or HCV in PLHIV prevalence or case fatality rate |
| 10 | Agbodjan, 1995 | [Serologic and genomic research by PCR of hepatitis C virus in different populations in Lome (Togo)] | Full text or abstract not found |
| 11 | Agha, 1998 | Transplacental transmission of hepatitis C virus in HIV-negative mothers. | No data on HBV and/or HCV in PLHIV prevalence or case fatality rate |
| 12 | Ahmed, 2008 | Brief report: acute viral hepatitis and poor maternal and perinatal outcomes in pregnant Sudanese women. | No data on HBV and/or HCV in PLHIV prevalence or case fatality rate |
| 13 | Ajayi, 2011 | Seroprevalence of other antibodies (herpes, CMV, rubella, varicella, hepatitis B and C, syphilis, chlamydia, mumps, toxoplasmosis) in HIV-positive patients. | Full text or abstract not found |
| 14 | Akanbi, 2019 | High frequency of drug resistance mutations in the HBV genome in ART-experienced HIV-coinfected patients in southwestern Nigeria. | Study with already HBV and/or HCV known result |
| 15 | Akanbi, 2016 | HBV and HIV Co-Infection: Prevalence and Clinical Outcomes in Tertiary Care Hospital Malaysia. | Study outside Africa |
| 16 | Akinyemi, 2017 | Demographic and epidemiological characteristics of HIV opportunistic infections among older adults in Nigeria. | No reported diagnostic target |
| 17 | Akmleye, 2013 | Blood safety and prevalence of transfusion transmissible viral infections among blood donors in Lagos, Nigeria. | Full text or abstract not found |
| 18 | Alao, 2010 | The sero-prevalence of hepatitis C virus (HCV) infection among prospective blood donors in Makurdi, Nigeria. | No data on HBV and/or HCV in PLHIV prevalence or case fatality rate |
| 19 | Alhuraiji, 2014 | Viral hepatitis B and C in HIV-infected patients in Saudi Arabia. | Study outside Africa |
| 20 | Ali, 2012 | Association of hepatitis c virus infection with type II diabetes in ethiopia: A hospital-based case-control study. | No data on HBV and/or HCV in PLHIV prevalence or case fatality rate |

| | | | |
|---|---|---|---|
| 21 | Alii, 2010 | A serosurvey of blood parasites (Plasmodium, microfilaria, HIV, HBsAG, HCV antibodies) in prospective Nigerian blood donors | No data on HBV and/or HCV in PLHIV prevalence or case fatality rate |
| 22 | Alkan, 1993 | Serological markers for hepatitis B and treponemal infection among HIV carriers from Ethiopia. | Study outside Africa |
| 23 | Allain, 2009 | Deferred donor care in a regional hospital blood center in Ghana. | No data on HBV and/or HCV in PLHIV prevalence or case fatality rate |
| 24 | Allain, 2010 | Relative safety of first-time volunteer and replacement donors in West Africa. | No data on HBV and/or HCV in PLHIV prevalence or case fatality rate |
| 25 | Amadi, 2009 | The epidemiology of hepatitis C virus infection among patients attending the federal dental clinic, Enugu. | No data on HBV and/or HCV in PLHIV prevalence or case fatality rate |
| 26 | Anderson, 2018 | Molecular Characterization of Near Full-Length Genomes of Hepatitis B Virus Isolated from Predominantly HIV Infected Individuals in Botswana. | No data on HBV and/or HCV in PLHIV prevalence or case fatality rate |
| 27 | Anderson, 2015 | Molecular characterisation of hepatitis B virus in HIV-1 subtype C infected patients in Botswana. | No data on HBV and/or HCV in PLHIV prevalence or case fatality rate |
| 28 | Anteneh, 2021 | Hepatitis B virus infection and its determinants among HIV positive pregnant women: Multicenter unmatched case-control study. | Study with already HBV and/or HCV known result |
| 29 | Aoudjane, 2014 | Hepatitis B virus sub-genotype A1 infection is characterized by high replication levels and rapid emergence of drug resistance in HIV-positive adults receiving first-line antiretroviral therapy in Malawi. | No data on HBV and/or HCV in PLHIV prevalence or case fatality rate |
| 30 | Apea-Kubi, 2006 | HTLV-1 and other viral sexually transmitted infections in antenatal and gynaecological patients in Ghana. | No data on HBV and/or HCV in PLHIV prevalence or case fatality rate |
| 31 | Attiku, 2021 | Circulation of hepatitis delta virus and occult hepatitis B virus infection amongst HIV/HBV co-infected patients in Korle-Bu, Ghana. | Study with already HBV and/or HCV known result |
| 32 | Ayana, 2020 | Occult Hepatitis B virus infection among HIV negative and positive isolated anti-HBc individuals in eastern Ethiopia. | Study with already HBV and/or HCV known result |
| 33 | Ayolabi, 2006 | Sero-prevalence of hepatitis C virus among blood donors in Lagos, Nigeria. | No data on HBV and/or HCV in PLHIV prevalence or case fatality rate |
| 34 | Bakaj, 2012 | Evaluation and treatment of hepatitis C in patients with human immunodeficiency virus. | Study outside Africa |
| 35 | Balogun, 2010 | Hepatitis C virus co infection in HIV positive patients. | Full text or abstract not found |
| 36 | Balogun, 2006 | Low prevalence of hepatitis-C viral seropositivity among patients with type-2 diabetes mellitus in a tertiary hospital. | No data on HBV and/or HCV in PLHIV prevalence or case fatality rate |
| 37 | Barbour, 2013 | Transient Hepatitis B Surface Antigenemia After Hepatitis B Virus Vaccine in an HIV-Infected Patient. | Comment on an article |
| 38 | Batina Agasa, 2010 | Multiple transfusions for sickle cell disease in the Democratic Republic of Congo: the importance of the hepatitis C virus | No data on HBV and/or HCV in PLHIV prevalence or case fatality rate |
| 39 | Bekondi, 2010 | [Etiopathological factors of hepatocellular carcinoma in Bangui, Central African Republic: clinical, biological characteristics and virological aspects of patients] | No data on HBV and/or HCV in PLHIV prevalence or case fatality rate |
| 40 | Benhammou, 2018 | HBV or HCV Coinfection in HIV-1-Infected Pregnant Women in France: Prevalence and Pregnancy Outcomes. | Study outside Africa |
| 41 | Berhe, 2007 | Intensity of Schistosoma mansoni, hepatitis B, age, and sex predict levels of hepatic periportal thickening/fibrosis (PPT/F): a large-scale community-based study in Ethiopia. A | No data on HBV and/or HCV in PLHIV prevalence or case fatality rate |

| | | | |
|---|---|---|---|
| 42 | Bharti, 2011 | Clinical variables identify seronegative HCV co-infection in HIV-infected individuals. | Study outside Africa |
| 43 | Biggar, 2006 | Hepatitis C Virus Genotype 4 in Ugandan Children and Their Mothers. | No data on HBV and/or HCV in PLHIV prevalence or case fatality rate |
| 44 | Bivigou-Mboumba, 2017 | Hepatitis B, C, and E infection among HIV-infected patients in Franceville, Gabon: retrospective cross-sectional study. | Duplicates |
| 45 | Blackard, 2014 | Evidence of Distinct Populations of Hepatitis C Virus in the Liver and Plasma of Patients Co-Infected With HIV and HCV. | Study with already HBV and/or HCV known result |
| 46 | Blanton, 2002 | Populationbased differences in Schistosoma mansoni- and hepatitis C-induced disease | No data on HBV and/or HCV in PLHIV prevalence or case fatality rate |
| 47 | Boyd, 2019 | Effect of hepatitis B virus (HBV) surface-gene variability on markers of replication during treated human immunodeficiency virus-HBV infection in Western Africa. | No data on HBV and/or HCV in PLHIV prevalence or case fatality rate |
| 48 | Boyles, 2011 | The prevalence of hepatitis B infection in a rural South African HIV clinic. | Study with already HBV and/or HCV known result |
| 49 | Breskin, 2015 | Factors Associated With Hepatitis C Infection Among HIV-Infected Men Who Have Sex With Men With No Reported Injection Drug Use in New York City, 2000-2010. | Study outside Africa |
| 50 | Bwogi, 2009 | Hepatitis B infection is highly endemic in Uganda: findings from a national serosurvey. | Study with already HBV and/or HCV known result |
| 51 | Campo, 2014 | Intra-host diversity and evolution of hepatitis C virus endemic to Cote d'Ivoire | No data on HBV and/or HCV in PLHIV prevalence or case fatality rate |
| 52 | Candotti, 2003 | Frequent recovery and broad genotype 2 diversity characterize Hepatitis C virus infection in Ghana, West Africa. | No data on HBV and/or HCV in PLHIV prevalence or case fatality rate |
| 53 | Cantaloube, 2010 | Analysis of hepatitis C virus strains circulating in Republic of the Congo. | No data on HBV and/or HCV in PLHIV prevalence or case fatality rate |
| 54 | Ceriani, 2014 | Prevalence of HBV, HDV, HCV, and HIV infection during pregnancy in northern Benin. | Sample size < or = 10 participants |
| 55 | Chadwick, 2013 | Response to antiretroviral therapy in occult hepatitis B and HIV co-infection in West Africa. | Study with already HBV and/or HCV known result |
| 56 | Chamie, 2007 | Factors associated with seronegative chronic hepatitis C virus infection in HIV infection. | Study outside Africa |
| 57 | Chasela, 2014 | Hepatitis B virus infection among HIV-infected pregnant women in Malawi and transmission to infants. | Prevalence estimated with multiple specimens from participants |
| 58 | Chen, 2011 | Prevalence of hepatitis B and C in HIV-infected patients: a meta-analysis. | Review |
| 59 | Chin'ombe, 2009 | Seroprevalence of HBV and HCV in primary hepatocellular carcinoma patients in Zimbabwe. | No data on HBV and/or HCV in PLHIV prevalence or case fatality rate |
| 60 | Chotun, 2015 | Hepatitis B virus infection in HIV-exposed infants in the Western Cape, South Africa. | No data on HBV and/or HCV in PLHIV prevalence or case fatality rate |
| 61 | Chukwurah, 2005 | Seroprevalence of Hepatitis C Virus (HCV) infection among blood donors in a South-Eastern State of Nigeria. | No data on HBV and/or HCV in PLHIV prevalence or case fatality rate |
| 62 | Cohen, 2007 | Awareness of hepatitis C infection among women with and at risk for HIV. | Study outside Africa |
| 63 | Dahaba, 2014 | Trends in hepatitis C infection among hemodialysis patients in Senegal: Results of a decade of prevention. | No data on HBV and/or HCV in PLHIV prevalence or case fatality rate |
| 64 | Daramola, 2002 | Hepatitis C virus and lichen planus in Nigerians: any relationship? | No data on HBV and/or HCV in PLHIV prevalence or case fatality rate |

| | | | |
|---|---|---|---|
| 65 | Dash, 2007 | Determinants of the variability of aflatoxin-albumin adduct levels in Ghanaians | No data on HBV and/or HCV in PLHIV prevalence or case fatality rate |
| 66 | Daw, 2014 | Seroprevalence of HBV, HCV & HIV co-infection and risk factors analysis in Tripoli-Libya. | Not possible to extract data on HBV and/or HCV in PLHIV prevalence or case fatality rate |
| 67 | de Lalla, 1990 | HIV, HBV, deltaagent and Treponema pallidum infections in two rural African area | Not possible to extract data on HBV and/or HCV in PLHIV prevalence or case fatality rate |
| 68 | de Waal, 2006 | Mass needle stick injury in children from the Western cape | No data on HBV and/or HCV in PLHIV prevalence or case fatality rate |
| 69 | Di Lello, 2012 | Low prevalence of occult HBV infection among HIV-infected patients in Southern Spain. | Study outside Africa |
| 70 | Diarra, 2009 | HIV, HCV, HBV and syphilis rate of positive donations among blood donations in Mali: Lower rates among volunteer blood donors | No data on HBV and/or HCV in PLHIV prevalence or case fatality rate |
| 71 | Diop, 2009 | [Prevention of transfusion transmitted malaria in endemic area]. | No data on HBV and/or HCV in PLHIV prevalence or case fatality rate |
| 72 | Diop-Ndiaye, 2008 | Hepatitis B, C seroprevalence and delta viruses in HIV-1 Senegalese patients at HAART initiation (retrospective study). | Prevalence estimated with multiple specimens from participants |
| 73 | Dokekias, 2003 | [Seroprevalence of viral hepatitis C in polytransfused patients at Central University Hospital of Brazzaville]. B | No data on HBV and/or HCV in PLHIV prevalence or case fatality rate |
| 74 | Dong, 2012 | Nevirapine pharmacokinetics and risk of rash and hepatitis among HIV-infected sub-Saharan African women. | No data on HBV and/or HCV in PLHIV prevalence or case fatality rate |
| 75 | Dray, 2005 | [Prevalences of HIV, hepatitis B and hepatitis C in blood donors in the Republic of Djibouti]. | Full text or abstract not found |
| 76 | Dunn, 2021 | New Insights on Long-Term Hepatitis B Virus Responses in HIV-Hepatitis B virus Co-infected Patients: Implications for Antiretroviral Management in Hepatitis B virus-Endemic Settings. | Duplicates |
| 77 | Durojaiye, 2014 | Seroprevalence of human T lymphotropic virus antibodies among healthy blood donors at a tertiary centre in Lagos, Nigeria | No data on HBV and/or HCV in PLHIV prevalence or case fatality rate |
| 78 | Durotoye, 2014 | Sero-prevalence of hepatitis B and C among mentally ill patients attending a tertiary hospital in Nigeria | No data on HBV and/or HCV in PLHIV prevalence or case fatality rate |
| 79 | Duru, 2009 | Rapid screening for co-infection of HIV and HCV in pregnant women in Benin City, Edo State, Nigeria. | No data on HBV and/or HCV in PLHIV prevalence or case fatality rate |
| 80 | Egah, 2007 | Hepatitis B surface antigen, hepatitis C and HIV antibodies in a low-risk blood donor group, Nigeria. | No data on HBV and/or HCV in PLHIV prevalence or case fatality rate |
| 81 | Ejiofor, 2009 | The role of blood transfusion on the prevalence of hepatitis C virus antibodies in children with sickle cell anaemia in Enugu, South East Nigeria. | No data on HBV and/or HCV in PLHIV prevalence or case fatality rate |
| 82 | El-Amin, 2007 | Hepatitis C virus infection in hemodialysis patients in Sudan: two centers' report. | No data on HBV and/or HCV in PLHIV prevalence or case fatality rate |
| 83 | Eller, 2012 | Single-cell level response of HIVspecific and cytomegalovirus-specific CD4 T cells correlate with viral control in chronic HIV-1 subtype a infection. | No data on HBV and/or HCV in PLHIV prevalence or case fatality rate |

| | | | |
|---|---|---|---|
| 84 | Elsheikh, 2007 | Hepatitis B virus and Hepatitis C virus in pregnant Sudanese women. Vi | No data on HBV and/or HCV in PLHIV prevalence or case fatality rate |
| 85 | Erhabor, 2006 | The risk of transfusion-acquired hepatitis-C virus infection among blood donors in Port Harcourt: the question of blood safety in Nigeria | No data on HBV and/or HCV in PLHIV prevalence or case fatality rate |
| 86 | Erhabor, 2007 | Epidemiology and management of occupational exposure to blood borne viral infections in a resource poor setting: the case for availability of post exposure prophylaxis | No data on HBV and/or HCV in PLHIV prevalence or case fatality rate |
| 87 | Etard, 2003 | Hepatitis C antibodies among blood donors, Senegal, 2001. | No data on HBV and/or HCV in PLHIV prevalence or case fatality rate |
| 88 | Falade-Nwulia, 2017 | High hepatitis C cure rates among black and nonblack human immunodeficiency virus-infected adults in an urban center. | Study with already HBV and/or HCV known result |
| 89 | Fang, 2003 | Human immunodeficiency virus-1 and hepatitis C virus RNA among South African blood donors: estimation of residual transfusion risk and yield of nucleic acid testing. | No data on HBV and/or HCV in PLHIV prevalence or case fatality rate |
| 90 | Fasola, 2008 | Trends in transfusion-transmitted viral infections from 2001 to 2006 in Ibadan, Nigeria. | No data on HBV and/or HCV in PLHIV prevalence or case fatality rate |
| 91 | Ferns, 2016 | Hepatitis C virus quasispecies and pseudotype analysis from acute infection to chronicity in HIV-1 co-infected individuals. | Study with already HBV and/or HCV known result |
| 92 | Fessehaye, 2011 | Transfusion transmitted infections - a retrospective analysis from the National Blood Transfusion Service in Eritrea. | No data on HBV and/or HCV in PLHIV prevalence or case fatality rate |
| 93 | Firnhaber, 2012 | Prevalence of hepatitis B virus (HBV) co-infection in HBV serologically-negative South African HIV patients and retrospective evaluation of the clinical course of mono- and co-infection. | No data on HBV and/or HCV in PLHIV prevalence or case fatality rate |
| 94 | Firnhaber, 2009 | Occult hepatitis B virus infection in patients with isolated core antibody and HIV coinfection in an urban clinic in Johannesburg, South Africa. | Duplicates |
| 95 | Fischer, 2010 | Hepatitis C and the Risk of Kidney Disease and Mortality in Veterans With HIV. | Study outside Africa |
| 96 | Floridia, 2010 | Declining HCV seroprevalence in pregnant women with HIV. | Study outside Africa |
| 97 | Fonquernie, 2006 | [Characteristics of newly managed HIV-infected patients: hospital Saint-Antoine, Paris 2002-2003]. | Study outside Africa |
| 98 | Foupouapouognigni, 2011 | Hepatitis B and C virus infections in the three Pygmy groups in Cameroon. J | No data on HBV and/or HCV in PLHIV prevalence or case fatality rate |
| 99 | Franceschini, 2006 | Immunosuppression, hepatitis C infection, and acute renal failure in HIV-infected patients. | Study outside Africa |
| 100 | Froeschl, 2021 | Hepatitis B, C and D virus prevalence in children and adults in Mbeya Region, Tanzania: results from a cohort study 2002 - 2009. | No data on HBV and/or HCV in PLHIV prevalence or case fatality rate |
| 101 | Gachara, 2017 | Characterization of occult hepatitis B virus infection among HIV positive patients in Cameroon. | Study with already HBV and/or HCV known result |
| 102 | Gasim, 2012 | Epidemiology of hepatitis B and hepatitis C virus infections among hemodialysis patients in Khartoum, Sudan | No data on HBV and/or HCV in PLHIV prevalence or case fatality rate |
| 103 | Gebo, 2003 | Hospitalization rates differ by hepatitis C satus in an urban HIV cohort. | Study outside Africa |
| 104 | Gededzha, 2016 | Complete genome analysis of hepatitis B virus in human immunodeficiency virus infected and uninfected South Africans. | Sample size < or = 10 participants |
| 105 | Gitau, 2016 | High Prevalence of Liver Fibrosis in Patients with Human Immunodeficiency Virus Monoinfection and Human Immunodeficiency Virus Hepatitis-B Co-infection as Assessed by Shear Wave Elastography: Study at a Teaching Hospital in Kenya. | Study with already HBV and/or HCV known result |

| | | | |
|---|---|---|---|
| 106 | Goni, 2013 | Hepatic transaminase and alkaline phosphatase enzyme levels in HIV/HBV co-infected and HIV mono-infected patients in Maiduguri, Nigeria. | No data on HBV and/or HCV in PLHIV prevalence or case fatality rate |
| 107 | Hall, 2004 | Hepatitis C virus infection in San Francisco's HIV-infected urban poor. | Study with already HBV and/or HCV known result |
| 108 | Hassall, 2012 | The microbiologic safety of umbilical cord blood transfusion for children with severe anemia in Mombasa, Kenya | No data on HBV and/or HCV in PLHIV prevalence or case fatality rate |
| 109 | Hawkins, 2017 | Brief Report: HIV/HBV Coinfection is a Significant Risk Factor for Liver Fibrosis in Tanzanian HIV-Infected Adults. | Study with already HBV and/or HCV known result |
| 110 | Hladik, 2006 | Prevalence and screening costs of hepatitis C virus among Ugandan blood donors. | No data on HBV and/or HCV in PLHIV prevalence or case fatality rate |
| 111 | Hønge, 2016 | Comment on Lô et al.: Prevalence of hepatitis B markers in Senegalese HIV-1 infected patients. | Comment on an article |
| 112 | Horth, 2020 | Hepatitis C Coinfection and Mortality in People Living with HIV in Middle Tennessee. | Study outside Africa |
| 113 | Igetei, 2010 | P53 codon 249 mutation and other risk factors among Nigerians with hepatocellular carcinoma. | No data on HBV and/or HCV in PLHIV prevalence or case fatality rate |
| 114 | Imarengiaye, 2006 | Risk of transfusiontransmitted hepatitis C virus in a tertiary hospital in Nigeria | No data on HBV and/or HCV in PLHIV prevalence or case fatality rate |
| 115 | Ionita, 2017 | Seroprevalence of hepatitis B virus and hepatitis C virus co-infection among people living with HIV/AIDS visiting antiretroviral therapy centres in Nepal: a first nationally representative study. | Study outside Africa |
| 116 | Iroezindu, 2013 | Prevalence of hepatitis B e antigen among human immunodeficiency virus and hepatitis B virus co-infected patients in Jos, Nigeria. | No data on HBV and/or HCV in PLHIV prevalence or case fatality rate |
| 117 | Jaka, 2014 | Hepatocellular carcinoma: Clinicopathological profile and challenges of management in a resource-limited setting. | No data on HBV and/or HCV in PLHIV prevalence or case fatality rate |
| 118 | Jeremiah, 2009 | Seroepidemiology of transfusion transmissible viral infection among university fresh students in Port Harcourt, Nigeria. | No data on HBV and/or HCV in PLHIV prevalence or case fatality rate |
| 119 | Jeremiah, 2008 | Prevalence of antibodies to hepatitis C virus in apparently healthy Port Harcourt blood donors and association with blood groups and other risk indicators. | No data on HBV and/or HCV in PLHIV prevalence or case fatality rate |
| 120 | Jespersen, 2015 | Cohort Profile: The Bissau HIV Cohort-a cohort of HIV-1, HIV-2 and co-infected patients. | No data on HBV and/or HCV in PLHIV prevalence or case fatality rate |
| 121 | Jiang, 2007 | Association of vitamin A deficiency with decrease in tumor necrosis factor-alpha expressing CD3-CD56+ natural killer cells in Ghanaians. | No data on HBV and/or HCV in PLHIV prevalence or case fatality rate |
| 122 | Johnston, 2010 | HIV risk and the overlap of injecting drug use and high-risk sexual behaviours among men who have sex with men in Zanzibar (Unguja), Tanzania. | Sample size < or = 10 participants |
| 123 | Jombo, 2005 | Hepatitis B Virus and Human Immunodeficiency Virus co-infection in Zawan community of Plateau State | Full text or abstract not found |
| 124 | Kabinda, 2002 | Viral hepatitis B and C in individuals infected with human immunodeficiency virus in Bukavu (South-Kivu), Democratic Republic of Congo. | Full text or abstract not found |
| 125 | Kaswa, 2020 | Prevalence of Hepatitis-B virus (HBV) coinfection among people living with HIV in Mthatha region of South Africa. | Full text or abstract not found |
| 126 | Katwesigye, 2016 | Low sero-prevalence of hepatitis delta antibodies in HIV/ hepatitis B co-infected patients attending an urban HIV clinic in Uganda. | No data on HBV and/or HCV in PLHIV prevalence or case fatality rate |

| | | | |
|---|---|---|---|
| 127 | Kauru, 2005 | Prevalence of HCV and HIV/HCV co-infection among volunteer blood donors and VCT clients | Full text or abstract not found |
| 128 | Kauru, 2005 | Prevalence of HCV and HCV/HIV co-infection among in-patients at the Kenyatta National Hospital. | Full text or abstract not found |
| 129 | Kellerman, 2003 | Prevalence of chronic hepatitis B and incidence of acute hepatitis B infection in human immunodeficiency virus-infected subjects. | Study outside Africa |
| 130 | Kerubo, 2015 | Hepatitis B, hepatitis C and HIV-1 coinfection in two informal urban settlements in Nairobi, Kenya | No data on HBV and/or HCV in PLHIV prevalence or case fatality rate |
| 131 | Kim, 2017 | Human Immunodeficiency Virus (HIV) and Hepatitis Virus Coinfection among HIV-Infected Korean Patients: The Korea HIV/AIDS Cohort Study. | Study outside Africa |
| 132 | Kirk, 2004 | The Gambia Liver Cancer Study: Infection with hepatitis B and C and the risk of hepatocellular carcinoma in West Africa | No data on HBV and/or HCV in PLHIV prevalence or case fatality rate |
| 133 | Kitundu, 2001 | Post-transfusion hepatitis C seroprevalence in Tanzanian children. | No data on HBV and/or HCV in PLHIV prevalence or case fatality rate |
| 134 | Klugman, 1991 | Serological markers of sexually transmitted diseases associated with HIV-1 infection in pregnant black women | No data on HBV and/or HCV in PLHIV prevalence or case fatality rate |
| 135 | Koate, 2005 | Seroprevalence of hepatitis C virus among blood donors in Rivers State, Nigeria. | No data on HBV and/or HCV in PLHIV prevalence or case fatality rate |
| 136 | Kone, 2012 | [Seroprevalence of human immunodeficiency virus, hepatitis B virus and hepatitis C virus among blood donors in Segou, Mali]. | No data on HBV and/or HCV in PLHIV prevalence or case fatality rate |
| 137 | Konidena, 2011 | Hepatitis C virus infection in patients with oral lichen planus. | No data on HBV and/or HCV in PLHIV prevalence or case fatality rate |
| 138 | Kouame, 2018 | No Impact of Hepatitis B Virus Infection on Early Mortality Among Human Immunodeficiency Virus-Infected Patients in Southern Africa Reply. | Comment on an article |
| 139 | Kouegnigan Rerambiah, 2014 | Evaluation of blood donors questionnaire in a developing country: The case of Gabon | No data on HBV and/or HCV in PLHIV prevalence or case fatality rate |
| 140 | Kurbanov, 2005 | A new subtype (subgenotype) Ac (A3) of hepatitis B virus and recombination between genotypes A and E in Cameroon. | No data on HBV and/or HCV in PLHIV prevalence or case fatality rate |
| 141 | Kuti, 2017 | HCV co-infection is associated with metabolic abnormalities among HAART naive HIV-infected persons. | Study with already HBV and/or HCV known result |
| 142 | Larsen, 2008 | Prevalence of hepatitis C and hepatitis B infection in the HIV-infected population of France, 2004. | Study outside Africa |
| 143 | Layden, 2015 | High Frequency of Active HCV Infection Among Seropositive Cases in West Africa and Evidence for Multiple Transmission Pathways. | No data on HBV and/or HCV in PLHIV prevalence or case fatality rate |
| 144 | Llenas-García, 2012 | [Clinico-epidemiological characteristics of HIV-positive immigrants: study of 371 cases]. | Article not in English or in French |
| 145 | Luetkemeyer, 2006 | Clinical presentation and course of acute hepatitis c infection in HIV-infected patients. | Sample size < or = 10 participants |
| 146 | Ly, 2018 | The Prevalence of Hepatitis C Virus Antibody in HIV-Negative Persons in Kenya, 2007. | No data on HBV and/or HCV in PLHIV prevalence or case fatality rate |
| 147 | Ma, 2019 | Hepatitis C care cascade in HIV patients at an urban clinic in the early direct-acting antiviral era. | Study outside Africa |
| 148 | Mabayoje, 2010 | Prevalence of hepatitis B surface antigen, hepatitis C and Human Immunodeficiency Virus antibodies in a population of students of tertiary institution in Nigeria | No data on HBV and/or HCV in PLHIV prevalence or case fatality rate |

| | | | |
|---|---|---|---|
| 149 | Maitha, 2021 | Influence of hepatitis B virus co-infection on virological and immunological response to antiretroviral treatment among HIV patients attending comprehensive care clinics in Makueni County, Kenya. | Study with already HBV and/or HCV known result |
| 150 | Malagnino, 2019 | HBcAb seropositivity is correlated with poor HIV viremia control in an Italian cohort of HIV/HBVcoinfected patients on first-line therapy. | Study outside Africa |
| 151 | Marite, 2011 | Occult hepatitis B in Cuban HIV patients. | Study outside Africa |
| 152 | Matthews-Greer, 2001 | Comparison of hepatitis C viral loads in patients with or without human immunodeficiency virus. | Study outside Africa |
| 153 | Mbanya, 2005 | Blood safety begins with safe donations: update among blood donors in Yaounde, Cameroon | Sample size < or = 10 participants |
| 154 | Mboto, 2005 | Hepatocellular carcinoma in The Gambia and the role of hepatitis B and hepatitis C | No data on HBV and/or HCV in PLHIV prevalence or case fatality rate |
| 155 | McGovern, 2006 | Hepatic steatosis is associated with fibrosis, nucleoside analogue use, and hepatitis C virus genotype 3 infection in HIV-seropositive patients. | Study outside Africa |
| 156 | Menendez, 1999 | Prevalence and mother-to-infant transmission of hepatitis viruses B, C, and E in Southern Tanzania. | No data on HBV and/or HCV in PLHIV prevalence or case fatality rate |
| 157 | Meschi, 2010 | The prevalence of antibodies to human herpesvirus 8 and hepatitis B virus in patients in two hospitals in Tanzania. | No data on HBV and/or HCV in PLHIV prevalence or case fatality rate |
| 158 | Miri-Dashe, 2014 | Comprehensive reference ranges for hematology and clinical chemistry laboratory parameters derived from normal Nigerian adults. | No data on HBV and/or HCV in PLHIV prevalence or case fatality rate |
| 159 | Modet, 2019 | Clinical characteristics and outcome of HIV infected patients with chronic kidney disease in Sub Saharan Africa: an example from in Cameroon. | No reported diagnostic target |
| 160 | Mogtomo, 2009 | [Screening of infectious microorganisms in blood banks in Douala (1995-2004)]. | No data on HBV and/or HCV in PLHIV prevalence or case fatality rate |
| 161 | Mohr, 2015 | Liver Fibrosis in HIV Patients Receiving a Modern cART Which Factors Play a Role? | Study outside Africa |
| 162 | Mohsen, 2005 | Prevalence of hepatitis C in an ethnically diverse HIV-1-infected cohort in south London. | Study outside Africa |
| 163 | Mosendane, 2012 | Nurses at risk for occupationally acquired blood-borne virus infection at a South African academic hospital. | No data on HBV and/or HCV in PLHIV prevalence or case fatality rate |
| 164 | Muasya, 2008 | Prevalence of hepatitis C virus and its genotypes among a cohort of drug users in Kenya. | No data on HBV and/or HCV in PLHIV prevalence or case fatality rate |
| 165 | Mudawi, 2007 | Epidemiology of HCV infection in Gezira state of central Sudan. | No data on HBV and/or HCV in PLHIV prevalence or case fatality rate |
| 166 | Mudawi, 2007 | Prevalence and common genotypes of HCV infection in Sudanese patients with hepatosplenic schistosomiasis | No data on HBV and/or HCV in PLHIV prevalence or case fatality rate |
| 167 | Mullaert, 2015 | Diphtheria, tetanus, poliomyelitis, yellow fever and hepatitis B seroprevalence among HIV1-infected migrants. Results from the ANRS VIHVO vaccine sub-study. | Study outside Africa |
| 168 | Nagi, 2007 | Seroprevalence of Hepatitis B and C Viral Infections among blood donors in Shendi, River Nile State, Sudan. | No data on HBV and/or HCV in PLHIV prevalence or case fatality rate |
| 169 | Namululi, 2013 | [Prevalence and incidence of HIV and hepatitis B among blood donors and estimated residual risk of transmission of HIV and HBV virus by blood transfusion. A study at the Provincial General Referee Hospital Bukavu, Democratic Republic of the Congo]. | No data on HBV and/or HCV in PLHIV prevalence or case fatality rate |

| | | | |
|---|---|---|---|
| 170 | Ndako, 2009 | Occurrence of hepatitis C virus infection in type 2 diabetic patients attending Plateau state specialist hospital Jos Nigeria | No data on HBV and/or HCV in PLHIV prevalence or case fatality rate |
| 171 | Ndong-Atome, 2008 | Hepatitis C virus prevalence and genetic diversity among pregnant women in Gabon, central Africa. | No data on HBV and/or HCV in PLHIV prevalence or case fatality rate |
| 172 | Ndong-Atome, 2009 | Absence of intrafamilial transmission of hepatitis C virus and low risk for sexual transmission in rural central Africa indicate a cohort effect. | No data on HBV and/or HCV in PLHIV prevalence or case fatality rate |
| 173 | Ndong-Atome, 2008 | High prevalence of hepatitis C virus infection and predominance of genotype 4 in rural Gabon. | No data on HBV and/or HCV in PLHIV prevalence or case fatality rate |
| 174 | Ndububa, 2005 | Chronic hepatitis in Nigerian patients: A study of 70 biopsy-proven cases. We | No data on HBV and/or HCV in PLHIV prevalence or case fatality rate |
| 175 | Ndumbe, 1993 | Hepatitis C virus infection in different populations in Cameroon. | No data on HBV and/or HCV in PLHIV prevalence or case fatality rate |
| 176 | Nerrienet, 2005 | Hepatitis C virus infection in cameroon: A cohort-effect. | No data on HBV and/or HCV in PLHIV prevalence or case fatality rate |
| 177 | Ngounoue, 2015 | Human Immunodeficiency Virus and Hepatitis C Virus Co-infection in Cameroon: Investigation of the Genetic Diversity and Virulent Circulating Strains | No data on HBV and/or HCV in PLHIV prevalence or case fatality rate |
| 178 | Ngueko, 2021 | Mass screening for hepatitis B and C viruses in a population of persons with disabilities with and without HIV status in Cameroon. | Full text or abstract not found |
| 179 | Njouom, 2003 | Hepatitis C virus infection among pregnant women in Yaounde, Cameroon: Prevalence, viremia, and genotypes. | No data on HBV and/or HCV in PLHIV prevalence or case fatality rate |
| 180 | Njouom, 2005 | Low risk of mother-tochild transmission of hepatitis C virus in Yaounde, Cameroon: the ANR | No data on HBV and/or HCV in PLHIV prevalence or case fatality rate |
| 181 | Njouom, 2003 | High rate of hepatitis C virus infection and predominance of genotype 4 among elderly inhabitants of a remote village of the rain forest of South Cameroon | No data on HBV and/or HCV in PLHIV prevalence or case fatality rate |
| 182 | Njouom, 2012 | Phylogeography, risk factors and genetic history of hepatitis C virus in Gabon, Central Africa. | No data on HBV and/or HCV in PLHIV prevalence or case fatality rate |
| 183 | Nkrumah, 2011 | Hepatitis B and C viral infections among blood donors from rural Ghana. | No data on HBV and/or HCV in PLHIV prevalence or case fatality rate |
| 184 | Nna, 2014 | Occult hepatitis B viral infection among blood donors in South-Eastern Nigeria. | No data on HBV and/or HCV in PLHIV prevalence or case fatality rate |
| 185 | Ntagirabiri, 2014 | Prevalence of hepatitis C virus in Burundi: A nationwide survey | Not possible to extract data on HBV and/or HCV in PLHIV prevalence or case fatality rate |
| 186 | Ntagirabiri, 2014 | Hepatic steatosis and metabolic syndrome in black African adult: Burundi case. | No data on HBV and/or HCV in PLHIV prevalence or case fatality rate |
| 187 | Ntakarutimana, 1995 | [Seroprevalence of hepatitis C virus among persons visiting the Burundi health services]. | No data on HBV and/or HCV in PLHIV prevalence or case fatality rate |
| 188 | Nurutdinova, 2011 | Risk factors associated with Hepatitis C among female substance users enrolled in community-based HIV prevention studies. | Study outside Africa |
| 189 | Nwankiti, 2009 | Hepatitis C Virus infection in apparentenly healthy individuals with family history of diabetes in Vom, Plateau State Nigeria | No data on HBV and/or HCV in PLHIV prevalence or case fatality rate |

| | | | |
|---|---|---|---|
| 190 | Nwankwo, 2012 | Seroprevalence of major bloodborne infections among blood donors in Kano, Nigeria | Sample size < or = 10 participants |
| 191 | Nwokedi, 2006 | Hepatitis C virus infection among teaching hospital patients in Kano, Nigeria: A retrospective study | No data on HBV and/or HCV in PLHIV prevalence or case fatality rate |
| 192 | Nwokediuko, 2011 | Risk factors for hepatitis C virus transmission obscure in Nigerian patients | No data on HBV and/or HCV in PLHIV prevalence or case fatality rate |
| 193 | Nwokediuko, 2008 | Hepatitis C virus infection in Nigerians with diabetes mellitus | No data on HBV and/or HCV in PLHIV prevalence or case fatality rate |
| 194 | Obuseh, 2010 | Aflatoxin B1 albumin adducts in plasma and aflatoxin M1 in urine are associated with plasma concentrations of vitamins A and E. | No data on HBV and/or HCV in PLHIV prevalence or case fatality rate |
| 195 | Ogboghodo, 2013 | Hepatitis C Virus and Human Immunodeficiency Virus-I (HIV) Co-Infection in Children in Benin City, Nigeria. | Sample size < or = 10 participants |
| 196 | Ogunro, 2007 | Prevalence of antihepatitis C virus antibodies in pregnant women and their offspring in a tertiary hospital in Southwestern Nigeria. J | No data on HBV and/or HCV in PLHIV prevalence or case fatality rate |
| 197 | Oje, 2012 | Dual positivity of hepatitis B surface antigen and antihepatitis C virus antibody and associated factors among apparently healthy patients of Ekiti State, Nigeria | No data on HBV and/or HCV in PLHIV prevalence or case fatality rate |
| 198 | Okonkwo, 2017 | Prevalence of hepatitis B, hepatitis C and human immunodeficiency viruses, and evaluation of risk factors for transmission: Report of a population screening in Nigeria | No data on HBV and/or HCV in PLHIV prevalence or case fatality rate |
| 199 | Okonkwo, 2011 | The clinical and pathological features of hepatocellular carcinoma in Nnewi, Nigeria. Nigerian Journal of Medicine: | No data on HBV and/or HCV in PLHIV prevalence or case fatality rate |
| 200 | Ola, 2002 | Serum hepatitis C virus and hepatitis B surface antigenaemia in Nigerian patients with acute icteric hepatit | No data on HBV and/or HCV in PLHIV prevalence or case fatality rate |
| 201 | Ola, 2009 | Occult HBV infection among a cohort of Nigerian adults | No data on HBV and/or HCV in PLHIV prevalence or case fatality rate |
| 202 | Olokoba, 2011 | Risk factors and clinical presentation of hepatitis C virus infection in Nigerians with chronic liver disease | No data on HBV and/or HCV in PLHIV prevalence or case fatality rate |
| 203 | Onakewhor, 2009 | The prevalence of dual human immunodeficiency virus/hepatitis C virus (HIV/HCV) infection in asymptomatic pregnant women in Benin City, Nigeria. | No data on HBV and/or HCV in PLHIV prevalence or case fatality rate |
| 204 | Onakewhor, 2009 | Seroprevalence of Hepatitis C viral antibodies in pregnancy in a tertiary health facility in Nigeria. | No data on HBV and/or HCV in PLHIV prevalence or case fatality rate |
| 205 | Onyekwere, 2002 | Prevalence of serological markers of chronic hepatitis B virus infection in diabetics in the Lagos University Teaching Hospital, Lagos. | No data on HBV and/or HCV in PLHIV prevalence or case fatality rate |
| 206 | Opaleye, 2014 | Occult Hepatitis B Virus Infection among HIV Positive Patients in Nigeria. | Study with already HBV and/or HCV known result |
| 207 | Opaleye, 2010 | HBV, HCV co-infection among blood donors in Nigeria. | No data on HBV and/or HCV in PLHIV prevalence or case fatality rate |
| 208 | Opaleye, 2011 | Prevalence and association of human parvovirus B19V with hepatitis B and C viruses in Nigeria. | No data on HBV and/or HCV in PLHIV prevalence or case fatality rate |
| 209 | Osasona, 2021 | Comparative serologic profiles of hepatitis B Virus (HBV) between HIV/HBV co-infected and Hbv mono-infected patients in Ile-Ife, Nigeria. | Study with already HBV and/or HCV known result |

| | | | |
|---|---|---|---|
| 210 | Osazuwa, 2012 | Sero-epidemiology of human immunodeficency virus, Hepatitis B and C among pregnant women in rural communities of Abaji Area Council, Nigeria | No data on HBV and/or HCV in PLHIV prevalence or case fatality rate |
| 211 | Otedo, 2003 | Seroprevalence of hepatitis B and C in maintenance dialysis in a public hospital in a developing country. S | No data on HBV and/or HCV in PLHIV prevalence or case fatality rate |
| 212 | Ouedraoga, 2012 | Prevalence of anti-CMV antibodies in blood donors in ouagadougou (Burkina Faso) | No data on HBV and/or HCV in PLHIV prevalence or case fatality rate |
| 213 | Owusu-Ofori, 2005 | Predonation screening of blood donors with rapid tests: implementation and efficacy of a novel approach to blood safety in resource-poor settings. T | No data on HBV and/or HCV in PLHIV prevalence or case fatality rate |
| 214 | Pasquier, 2005 | Distribution and heterogeneity of hepatitis C genotypes in hepatitis patients in Cameroon. | No data on HBV and/or HCV in PLHIV prevalence or case fatality rate |
| 215 | Patel, 2020 | Prevalence and genetic variability of occult hepatitis B virus in a human immunodeficiency virus positive patient cohort in Gondar, Ethiopia. | Study with already HBV and/or HCV known result |
| 216 | Pepin, 2010 | Iatrogenic transmission of human T cell lymphotropic virus type 1 and hepatitis C virus through parenteral treatment and chemoprophylaxis of sleeping sickness in colonial Equatorial Africa. | No data on HBV and/or HCV in PLHIV prevalence or case fatality rate |
| 217 | Pérez-Molina, 2009 | Clinicoepidemiological characteristics of HIV-infected immigrants attended at a tropical medicine referral unit. | Study outside Africa |
| 218 | Peto, 2014 | Efficacy and effectiveness of infant vaccination against chronic hepatitis B in the Gambia Hepatitis Intervention Study (1986-90) and in the nationwide immunisation program. | No data on HBV and/or HCV in PLHIV prevalence or case fatality rate |
| 219 | Phinius, 2020 | Incidence of hepatitis B virus infection among human immunodeficiency virus -infected treatment naive adults in Botswana. | Full text or abstract not found |
| 220 | Pippi, 2008 | Serological response to hepatitis B virus vaccine in HIV-infected children in Tanzania. | No data on HBV and/or HCV in PLHIV prevalence or case fatality rate |
| 221 | Piroth, 2010 | Management and treatment of chronic hepatitis B virus infection in HIV positive and negative patients: The EPIB 2008 study. | Study outside Africa |
| 222 | Piroth, 2007 | Epidemiology, diagnosis and treatment of chronic hepatitis B in HIV-infected patients (EPIB 2005 STUDY). | Study outside Africa |
| 223 | Powell, 2016 | Functional analysis of 'a' determinant mutations associated with occult HBV in HIV-positive South Africans. | No data on HBV and/or HCV in PLHIV prevalence or case fatality rate |
| 224 | Puato, 2007 | Does HCV infection have a more favourable outcome in Tanzanian people? Data from the Lugalawa study. | No data on HBV and/or HCV in PLHIV prevalence or case fatality rate |
| 225 | Ramos, 2012 | Prevalence of HIV, HBV, HCV, HTLV and Treponema pallidum among patients attending a rural hospital in Southern Ethiopia. J | No data on HBV and/or HCV in PLHIV prevalence or case fatality rate |
| 226 | Randriamanantany, 2012 | Prevalence and trends of hepatitis C virus among blood donors in Antananarivo, from 2003 to 2009. | No data on HBV and/or HCV in PLHIV prevalence or case fatality rate |
| 227 | Reddy, 2009 | mpact of individualdonation nucleic acid testing on risk of human immunodeficiency virus, hepatitis B virus, and hepatitis C virus transmission by blood transfusion in South Africa. | No data on HBV and/or HCV in PLHIV prevalence or case fatality rate |
| 228 | Rerambiah, 2014 | The risk of transfusiontransmitted viral infections at the Gabonese National Blood Transfusion Centre. | No data on HBV and/or HCV in PLHIV prevalence or case fatality rate |
| 229 | Reuter, 2011 | Prevalence and characteristics of hepatitis B and C virus infections in treatment-na < ve HIV-infected patients. | Study outside Africa |
| 230 | Rodrigues, 2008 | HIV/HCV coinfection in Infectious Disease Units in Mozambique and Brazil: a comparative study | Not possible to extract data on HBV and/or HCV in |

| | | | |
|---|---|---|---|
| | | | PLHIV prevalence or case fatality rate |
| 231 | Rwegasha, 2019 | Hepatitis B virus coinfection is associated with high early mortality in HIV-infected Tanzanians on antiretroviral therapy. | No data on HBV and/or HCV in PLHIV prevalence or case fatality rate |
| 232 | Ryan, 2017 | High Rates of Occult Hepatitis B Virus Infection in HIV-Positive Individuals Initiating Antiretroviral Therapy in Botswana. | Duplicates |
| 233 | Sajadi, 2010 | Hepatitis C infection in HIV-1 natural viral suppressors. | No data on HBV and/or HCV in PLHIV prevalence or case fatality rate |
| 234 | Salazar-Vizcaya, 2016 | Hepatitis C virus transmission among human immunodeficiency virus-infected men who have sex with men: Modeling the effect of behavioral and treatment interventions. | Study outside Africa |
| 235 | Sarkar, 2013 | Racial/ethnic differences in spontaneous HCV clearance in HIV infected and uninfected women. | Study outside Africa |
| 236 | Segala, 2021 | Prevalence of Sexually Transmitted Infections and Factors Associated with HIV Status Among Vulnerable Women in Northern Uganda: Baseline Results from Pe Atye Kena Cohort Study. | No data on HBV and/or HCV in PLHIV prevalence or case fatality rate |
| 237 | Segbena, 2005 | [Human immunodeficiency virus, hepatitis C virus and hepatitis B viruses in patients with sickle-cell disease in Togo]. | Sample size < or = 10 participants |
| 238 | Seremba, 2017 | Hepatitis B incidence and prevention with antiretroviral therapy among HIV-positive individuals in Uganda. | No data on HBV and/or HCV in PLHIV prevalence or case fatality rate |
| 239 | Shao, 1993 | Association of hepatitis B and human immunodeficiency virus infections in Tanzanian population groups. | Not possible to extract data on HBV and/or HCV in PLHIV prevalence or case fatality rate |
| 240 | Sherman, 2005 | Viral kinetics in hepatitis C or hepatitis C/human immunodeficiency virus-infected patients. | No data on HBV and/or HCV in PLHIV prevalence or case fatality rate |
| 241 | Shibayama, 2005 | Characterization of seven genotypes (A to E, G and H) of hepatitis B virus recovered from Japanese patients infected with human immunodeficiency virus type 1. | Study outside Africa |
| 242 | Shores, 2008 | Sexual transmission is associated with spontaneous HCV clearance in HIV-infected patients. | Study outside Africa |
| 243 | Singh, 2019 | Response of hepatitis B virus to antiretroviral treatment containing lamivudine in HBsAg-positive and HBsAg-negative HIV-positive South African adults. | Duplicates |
| 244 | Siransy, 2015 | ABO/Rh Blood Groups and Risk of HIV Infection and Hepatitis B Among Blood Donors of Abidjan, Cote D'ivoire. | No data on HBV and/or HCV in PLHIV prevalence or case fatality rate |
| 245 | Siza, 2020 | Proportion and Characterization of Co-infections of HIV and Hepatitis C or Hepatitis B among People with HIV in Alabama, 2007-2016. | Study outside Africa |
| 246 | Smith, 2005 | Prevalence of GB virus type C in urban Americans infected with human immunodeficiency virus type 1. | Study outside Africa |
| 247 | Steiner, 2017 | Predominance of Hepatitis B Virus Genotype A Among Treated HIV Infected Patients Experiencing High Hepatitis B Virus Drug Resistance in Nairobi, Kenya. | No data on HBV and/or HCV in PLHIV prevalence or case fatality rate |
| 248 | Stevens, 2008 | Baseline morbidity in 2,990 adult African volunteers recruited to characterize laboratory reference intervals for future HIV vaccine clinical trials | No data on HBV and/or HCV in PLHIV prevalence or case fatality rate |
| 249 | Strand, 2003 | Infectious aetiology of jaundice among pregnant women in Angola. | No data on HBV and/or HCV in PLHIV prevalence or case fatality rate |
| 250 | Sun, 2010 | Factors associated with isolated anti-hepatitis B core antibody in HIV-positive patients: impact of compromised immunity. | Study outside Africa |

| | | | |
|---|---|---|---|
| 251 | Sunmonu, 2012 | Cognitive function in patients with liver cirrhosis without overt hepatic encephalopathy: Assessment using an automated neuropsychological test battery. | No data on HBV and/or HCV in PLHIV prevalence or case fatality rate |
| 252 | Szczech, 2003 | The clinical characteristics and antiretroviral dosing patterns of HIV-infected patients receiving dialysis. | Study outside Africa |
| 253 | Szymanska, 2004 | Ser-249 TP53 mutation in tumour and plasma DNA of hepatocellular carcinoma patients from a high incidence area in the Gambia, west Africa. | No data on HBV and/or HCV in PLHIV prevalence or case fatality rate |
| 254 | Tagny, 2014 | Screening for hepatitis C virus infection in a high prevalence country by an antigen/antibody combination assay versus a rapid test. | No data on HBV and/or HCV in PLHIV prevalence or case fatality rate |
| 255 | Tamandjou Tchuem, 2020 | Viral hepatitis B and C in HIV-exposed South African infants. | No data on HBV and/or HCV in PLHIV prevalence or case fatality rate |
| 256 | Tao, 2014 | Seroepidemiology of hepatitis B and C viruses in the general population of burkina faso | Sample size < or = 10 participants |
| 257 | Tedaldi, 2003 | Prevalence and characteristics of hepatitis C virus coinfection in a human immunodeficiency virus clinical trials group: the Terry Beirn Community Programs for Clinical Research on AIDS. | Study outside Africa |
| 258 | Teresa Alvarez-Munoz, 2014 | Occult hepatitis B virus infection among Mexican human immunodeficiency virus-1-infected patients. | Study outside Africa |
| 259 | Thio, 2013 | Characterization of HIV-HBV coinfection in a multinational HIV-infected cohort. | Study with already HBV and/or HCV known result |
| 260 | Thng, 2012 | The perils of relying on anti-hepatitis B total core antibody in screening individuals infected with HIV. | Case report |
| 261 | Tiruneh, 2008 | Seroprevalence of multiple sexually transmitted infections among antenatal clinic attendees in Gondar Health Center, northwest Ethiopia. | Full text or abstract not found |
| 262 | Tonetto, 2009 | Hepatitis B virus: molecular genotypes and HBeAg serological status among HBV-infected patients in the southeast of Brazil. | Study outside Africa |
| 263 | Torimiro, 2018 | Rates of HBV, HCV, HDV and HIV type 1 among pregnant women and HIV type 1 drug resistance-associated mutations in breastfeeding women on antiretroviral therapy. | No data on HBV and/or HCV in PLHIV prevalence or case fatality rate |
| 264 | Torrecilla García, 2016 | Clinical, epidemiological and treatment failure data among HIV-1 non-B-infected patients in the Spanish AIDS Research Network Cohort. | Study outside Africa |
| 265 | Ugbebor, 2011 | The prevalence of hepatitis B and C viral infections among pregnant women. | No data on HBV and/or HCV in PLHIV prevalence or case fatality rate |
| 266 | Ukonu, 2012 | The prevalence of hepatitis C Virus (HCV) among lichen planus patients and its clinical pattern at the University of Abuja Teaching Hospital, Gwagwalada, Abuja, Nigeria. | No data on HBV and/or HCV in PLHIV prevalence or case fatality rate |
| 267 | Ummate, 2014 | Risk factors for hepatitis C virus sero-positivity among haemodialysis patients receiving care at Kidney Centre in a tertiary health facility in Maiduguri, Nigeria. | No data on HBV and/or HCV in PLHIV prevalence or case fatality rate |
| 268 | Vardas, 2002 | Viral hepatitis in South African healthcare workers at increased risk of occupational exposure to blood-borne viruses. | No data on HBV and/or HCV in PLHIV prevalence or case fatality rate |
| 269 | Vetter, 2020 | Sensitivity and specificity of rapid diagnostic tests for hepatitis C virus with or without HIV coinfection: a multicentre laboratory evaluation study. | Study with already HBV and/or HCV known result |
| 270 | Viegas, 2015 | Incidence of HIV and the prevalence of HIV, hepatitis B and syphilis among youths in Maputo, Mozambique: a cohort study. | No data on HBV and/or HCV in PLHIV prevalence or case fatality rate |
| 271 | Vray, 2006 | Molecular epidemiology of hepatitis B virus in Dakar, Senegal. | No data on HBV and/or HCV in PLHIV prevalence or case fatality rate |

| | | | |
|---|---|---|---|
| 272 | Wahome, 2017 | Hepatitis B Virus Incidence and Risk Factors Among Human Immunodeficiency Virus-1 Negative Men Who Have Sex With Men in Kenya. | No data on HBV and/or HCV in PLHIV prevalence or case fatality rate |
| 273 | Weitzel, 2020 | Hepatitis B and C virus infection among HIV patients within the public and private healthcare systems in Chile: A cross-sectional serosurvey. | Study outside Africa |
| 274 | Winter, 2016 | Absence of hepatitis delta infection in a large rural HIV cohort in Tanzania. | Study with already HBV and/or HCV known result |
| 275 | Yamaguchi, 1993 | HTLV-I, HIV-I, and hepatitis B and C viruses in Western Province, Papua New Guinea: a serological survey. | Study outside Africa |
| 276 | Yami, 2011 | Hepatitis B and C viruses Infections and Their association with Human Immunodeficiency Virus: A cross-sectional study among blood donors in Ethiopia | No data on HBV and/or HCV in PLHIV prevalence or case fatality rate |
| 277 | Yousif, 2014 | Genotyping and virological characteristics of hepatitis B virus in HIV-infected individuals in Sudan. | No data on HBV and/or HCV in PLHIV prevalence or case fatality rate |
| 278 | Zeba, 2014 | Characterisation of hepatitis C virus genotype among blood donors at the regional blood transfusion centre of Ouagadougou, Burkina Faso | No data on HBV and/or HCV in PLHIV prevalence or case fatality rate |
| 279 | Ziglam, 2012 | Prevalence of antibodies to human immunodeficiency virus, hepatitis B, and hepatitis C in prisoners in Libya. | No data on HBV and/or HCV in PLHIV prevalence or case fatality rate |
