# Supplementary material for: Epidemiology of hepatitis B virus and/or hepatitis C virus infections among people living with human immunodeficiency virus in Africa: A systematic review and meta-analysis
Source: PLoS One. 2022 May 31;17(5):e0269250. doi: 10.1371/journal.pone.0269250 (PMC9154112; doi:10.1371/journal.pone.0269250)
Supplement: S5 Table — (PDF) [file pone.0269250.s006.pdf]

S5 Table. Characteristics of included studies

Characteristics	Overall (568)	CFR (5)	HBV prevalence (313)	HCV prevalence (210)	HBV and HCV prevalence (40)
**Year of publication ; range**	1990-2022	2008-2018	1990-2022	1991-2021	2006-2021
**Period of inclusion of participants; range**	1987-2022	2004-2016	1987-2020	1988-2022	1998-2019
**Study Design**					
Case control	24 (4.2)		13 (4.2)	8 (3.8)	3 (7.5)
Clinical Trial (Baseline data)	18 (3.2)	1 (20.0)	15 (4.8)	1 (0.5)	1 (2.5)
Cohort (Baseline data)	65 (11.4)	4 (80.0)	34 (10.9)	21 (10.0)	6 (15.0)
Cross sectional	461 (81.2)		251 (80.2)	180 (85.7)	30 (75.0)
**Sampling**					
Non probabilistic	484 (85.2)	4 (80.0)	266 (85.0)	183 (87.1)	31 (77.5)
Probabilistic	84 (14.8)	1 (20.0)	47 (15.0)	27 (12.9)	9 (22.5)
**Sampling method**					
Consecutive sampling	452 (79.6)	4 (80.0)	249 (79.6)	170 (81.0)	29 (72.5)
Convenience sampling	32 (5.6)		17 (5.4)	13 (6.2)	2 (5.0)
Respondent-driven sampling	2 (0.4)		1 (0.3)	1 (0.5)	
Simple random sampling	56 (9.9)	1 (20.0)	33 (10.5)	17 (8.1)	5 (12.5)
Snowball sampling	4 (0.7)		1 (0.3)	3 (1.4)	
Stratified sampling	8 (1.4)		5 (1.6)	2 (1.0)	1 (2.5)
Systematic sampling	14 (2.5)		7 (2.2)	4 (1.9)	3 (7.5)
**Number of sites**					
Monocenter	395 (69.5)	4 (80.0)	208 (66.5)	150 (71.4)	33 (82.5)
Multicenter	170 (29.9)	1 (20.0)	104 (33.2)	59 (28.1)	6 (15.0)
Nationally representative	3 (0.5)		1 (0.3)	1 (0.5)	1 (2.5)
**Timing of samples collection**					
Prospetively	454 (79.9)	4 (80.0)	253 (80.8)	168 (80.0)	29 (72.5)
Retroprospectively	5 (0.9)		2 (0.6)	3 (1.4)	
Retrospectively	109 (19.2)	1 (20.0)	58 (18.5)	39 (18.6)	11 (27.5)
**Countries**					
Angola	2 (0.4)		1 (0.3)	1 (0.5)	
Benin	1 (0.2)		1 (0.3)		
Botswana	8 (1.4)		6 (1.9)	2 (1.0)	
Burkina Faso	26 (4.6)		13 (4.2)	11 (5.2)	2 (5.0)
Burundi	1 (0.2)			1 (0.5)	
Cameroon	33 (5.8)		17 (5.4)	13 (6.2)	3 (7.5)
Central African Republic	2 (0.4)		1 (0.3)	1 (0.5)	

Chad	3 (0.5)		1 (0.3)	1 (0.5)	1 (2.5)
Democratic Republic of the Congo	8 (1.4)		6 (1.9)	2 (1.0)	
Egypt	1 (0.2)			1 (0.5)	
Equatorial Guinea	2 (0.4)		1 (0.3)	1 (0.5)	
Ethiopia	39 (6.9)		18 (5.8)	16 (7.6)	5 (12.5)
Gabon	4 (0.7)		1 (0.3)	3 (1.4)	
Gambia	6 (1.1)	1 (20.0)	2 (0.6)	3 (1.4)	
Ghana	26 (4.6)		13 (4.2)	11 (5.2)	2 (5.0)
Guinea	2 (0.4)		2 (0.6)		
Guinea-Bissau	6 (1.1)		3 (1.0)	3 (1.4)	
Ivory Coast	25 (4.4)	2 (40.0)	18 (5.8)	5 (2.4)	
Ivory Coast, Benin, Burkina Faso, Cameroon, Kenya, Senegal, South Africa, Togo, Uganda, Zambia	1 (0.2)		1 (0.3)		
Kenya	26 (4.6)	1 (20.0)	15 (4.8)	8 (3.8)	2 (5.0)
Lesotho	6 (1.1)		3 (1.0)	3 (1.4)	
Libya	2 (0.4)		1 (0.3)	1 (0.5)	
Malawi	22 (3.9)		13 (4.2)	7 (3.3)	2 (5.0)
Mali	6 (1.1)		3 (1.0)	3 (1.4)	
Morocco	4 (0.7)		2 (0.6)	2 (1.0)	
Mozambique	12 (2.1)		8 (2.6)	4 (1.9)	
Namibia	2 (0.4)		1 (0.3)	1 (0.5)	
Niger	2 (0.4)		1 (0.3)		1 (2.5)
Nigeria	121 (21.3)	1 (20.0)	57 (18.2)	47 (22.4)	16 (40.0)
Republic of the Congo	1 (0.2)			1 (0.5)	
Rwanda	9 (1.6)		4 (1.3)	4 (1.9)	1 (2.5)
Senegal	11 (1.9)		7 (2.2)	3 (1.4)	1 (2.5)
Sierra Leone	14 (2.5)		7 (2.2)	7 (3.3)	
South Africa	57 (10.0)		42 (13.4)	15 (7.1)	
South Africa, Tanzania, Uganda, Zimbabwe	2 (0.4)		2 (0.6)		
Sudan	3 (0.5)		2 (0.6)	1 (0.5)	
Swaziland	1 (0.2)		1 (0.3)		
Tanzania	26 (4.6)		13 (4.2)	11 (5.2)	2 (5.0)
Togo	4 (0.7)		3 (1.0)	1 (0.5)	
Tunisia	3 (0.5)		1 (0.3)	2 (1.0)	
Uganda	23 (4.1)		12 (3.8)	10 (4.8)	1 (2.5)
Zambia	12 (2.1)		8 (2.6)	3 (1.4)	1 (2.5)

Zimbabwe	3 (0.5)		2 (0.6)	1 (0.5)	
**UNAIDS Region**					
East and Southern	249 (43.8)	1 (20.0)	148 (47.3)	86 (41.0)	14 (35.0)
East and Southern, West and Central	1 (0.2)		1 (0.3)		
North Africa and Middle East	13 (2.3)		6 (1.9)	7 (3.3)	
Unclear	1 (0.2)		1 (0.3)		
West and Central	304 (53.5)	4 (80.0)	157 (50.2)	117 (55.7)	26 (65.0)
**WHO Region**					
Africa	555 (97.7)	5 (100)	307 (98.1)	203 (96.7)	40 (100)
Eastern Mediterranean	13 (2.3)		6 (1.9)	7 (3.3)	
**UNSD Region**					
Central Africa	55 (9.7)		28 (9.0)	23 (11.0)	4 (10.0)
Central Africa, Eastern Africa, Southern Africa, West Africa	1 (0.2)		1 (0.3)		
Eastern Africa	173 (30.5)	1 (20.0)	93 (29.7)	65 (31.0)	14 (35.0)
Eastern Africa, Southern Africa	2 (0.4)		2 (0.6)		
Northern Africa	13 (2.3)		6 (1.9)	7 (3.3)	
Southern Africa	74 (13.0)		53 (16.9)	21 (10.0)	
West Africa	250 (44.0)	4 (80.0)	130 (41.5)	94 (44.8)	22 (55.0)
**Country income level**					
Low-income economies	188 (33.1)	1 (20.0)	99 (31.6)	75 (35.7)	13 (32.5)
Low-income economies, Lower-middle-income economies	1 (0.2)		1 (0.3)		
Low-income economies, Lower-middle-income economies, Upper-middle-income economies	2 (0.4)		2 (0.6)		
Lower-middle income economies	301 (53.0)	4 (80.0)	158 (50.5)	112 (53.3)	27 (67.5)
Unclear	1 (0.2)		1 (0.3)		
Upper-middle-income economies	75 (13.2)		52 (16.6)	23 (11.0)	
**Age range**					
Adults	290 (51.1)	3 (60.0)	157 (50.2)	110 (52.4)	20 (50.0)
All ages	63 (11.1)		37 (11.8)	20 (9.5)	6 (15.0)
Children	42 (7.4)	1 (20.0)	24 (7.7)	15 (7.1)	2 (5.0)
Unclear/Not reported	173 (30.5)	1 (20.0)	95 (30.4)	65 (31.0)	12 (30.0)
**Setting**					
Community-based	22 (3.9)		11 (3.5)	11 (5.2)	
Hospital-based	544 (95.8)	5 (100)	302 (96.5)	197 (93.8)	40 (100)
Hospital/community based	2 (0.4)			2 (1.0)	

**Rural/Urban**					
Rural	11 (1.9)		7 (2.2)	4 (1.9)	
Unclear/Not reported	528 (93.0)	1 (20.0)	95 (30.4)	65 (31.0)	12 (30.0)
Urban	8 (1.4)		7 (2.2)	1 (0.5)	
Urban/Rural	21 (3.7)		10 (3.2)	7 (3.3)	4 (10.0)
**ART status**					
ART naïve	74 (13.0)		47 (15.0)	22 (10.5)	5 (12.5)
ART naïve/ On ART	104 (18.3)	1 (20.0)	56 (17.9)	40 (19.1)	7 (17.5)
On ART	128 (22.5)	3 (60.0)	74 (23.6)	40 (19.1)	11 (27.5)
Unclear/Not reported	262 (46.1)	1 (20.0)	95 (30.4)	65 (31.0)	12 (30.0)
**PLHIV Study population**					
Blood donors	42 (7.4)		22 (7.0)	16 (7.6)	4 (10.0)
Commercial sex workers	1 (0.2)			1 (0.5)	
Deceased people	2 (0.4)		1 (0.3)	1 (0.5)	
General population	426 (75.0)	5 (100)	235 (75.1)	155 (73.8)	31 (77.5)
Injecting drug users	9 (1.6)		5 (1.6)	4 (1.9)	
Men who have sex with men	2 (0.4)		1 (0.3)	1 (0.5)	
Patients with cancer	4 (0.7)		2 (0.6)	2 (1.0)	
Pregnant women	79 (13.9)		45 (14.4)	29 (13.8)	5 (12.5)
Prisoners	3 (0.5)		2 (0.6)	1 (0.5)	
**HBV and/or HCV**					
CFR	5 (0.9)	5 (100)			
HBV	313 (55.1)		313 (100)		
HBV and HCV	40 (7.0)				40 (100)
HCV	210 (37.0)			210 (100)	
**HBV and/or HCV_diagnostic_method**					
ADVIA Centaur chemiluminometric immunoassay system	7 (1.2)	1 (20.0)	6 (1.9)		
Agglutination test kits	2 (0.4)		2 (0.6)		
Auszyme Assay	2 (0.4)		2 (0.6)		
Chemiluminescent enzyme immunoassay (CLEIA)	16 (2.8)		11 (3.5)	5 (2.4)	
Chromatographic immunoassay technique	1 (0.2)		1 (0.3)		
Classical PCR	7 (1.2)		5 (1.6)	2 (1.0)	
Classical RT-PCR	8 (1.4)			8 (3.8)	
Direct ELISA	118 (20.8)	2 (40.0)	107 (34.2)	9 (4.3)	
Direct ELISA, Indirect ELISA	22 (3.9)		1 (0.3)		21 (52.5)

Electro-chemiluminescence immunoassay (ECLIA)	6 (1.1)		5 (1.6)	1 (0.5)	
Enzyme immunoassay (EIA)	85 (15.0)	1 (20.0)	49 (15.7)	31 (14.8)	4 (10.0)
Enzyme immunoassay (EIA), Indirect ELISA	1 (0.2)				1 (2.5)
Enzyme immunoassay (EIA), Rapid Diagnostic test	1 (0.2)				1 (2.5)
Immunoassay kit	28 (4.9)		21 (6.7)	6 (2.9)	1 (2.5)
Immunochromatographic test	24 (4.2)		12 (3.8)	9 (4.3)	3 (7.5)
Indirect ELISA	81 (14.3)		2 (0.6)	79 (37.6)	
Indirect ELISA, Direct ELISA	1 (0.2)				1 (2.5)
Inter Second Antibody Immunoassay (ISAI)	3 (0.5)		1 (0.3)	1 (0.5)	1 (2.5)
Microparticle Enzyme Immunoassay (MEIA)	1 (0.2)		1 (0.3)		
Neutralization test	2 (0.4)		2 (0.6)		
Passive haemagglutination test	1 (0.2)			1 (0.5)	
Radioimmunoassay	1 (0.2)		1 (0.3)		
Rapid Diagnostic test	110 (19.4)	1 (20.0)	64 (20.5)	41 (19.5)	4 (10.0)
Real-time PCR	6 (1.1)		5 (1.6)	1 (0.5)	
Real-time PCR, Real-time RT-PCR	1 (0.2)				1 (2.5)
Real-time RT-PCR	4 (0.7)			4 (1.9)	
Reverse passive hemagglutination assay	2 (0.4)		2 (0.6)		
Serological tests	1 (0.2)		1 (0.3)		
Transcription-mediated amplification assay	1 (0.2)			1 (0.5)	
Unclear/Not reported	24 (4.2)	1 (20.0)	95 (30.4)	65 (31.0)	12 (30.0)
VERSANT HCV RNA Qualitative Assay	1 (0.2)			1 (0.5)	
**Target detected**					
Anti-HCV	193 (34.0)			193 (91.9)	
Anti-HCV, HBsAg	39 (6.9)				39 (97.5)
HBeAg	34 (6.0)		34 (10.9)		
HBsAg	270 (47.5)	5 (100)	265 (84.7)		
HBsAg + IgM anti-HBc	2 (0.4)		2 (0.6)		
Viral DNA	12 (2.1)		12 (3.8)		
Viral DNA, Viral RNA	1 (0.2)				1 (2.5)
Viral RNA	17 (3.0)			17 (8.1)	
**Risk of bias**					
Low risk of bias	321 (56.5)	4 (80.0)	179 (57.2)	114 (54.3)	24 (60.0)
Moderate risk of bias	247 (43.5)	1 (20.0)	134 (42.8)	96 (45.7)	16 (40.0)
