# Supplementary material for: Epidemiology of hepatitis B virus and/or hepatitis C virus infections among people living with human immunodeficiency virus in Africa: A systematic review and meta-analysis
Source: PLoS One. 2022 May 31;17(5):e0269250. doi: 10.1371/journal.pone.0269250 (PMC9154112; doi:10.1371/journal.pone.0269250)
Supplement: S6 Table — (PDF) [file pone.0269250.s007.pdf]

S6 Table: Individual characteristics of included studies

Author	Year of publication	Study Design	Sampling	Sampling method	Number of sites	Timing of samples collection	Countries	UNAIDS Region	WHO Region	UNSD Region	Country income level	Study period	Age range	Setting	Rural/Urban	ART status	PLHIV Study population	HBV and/or HCV	HBV and/or HCV_diagnostic_method	Target detected
Abera	2014	Cross sectional	Non probabilistic	Consecutive sampling	Monocenter	Prospetively	Ethiopia	East and Southern	Africa	Eastern Africa	Low-income economies	May/2014	Children	Hospital-based	Urban/Rural	ART naïve/ On ART	General population	HCV	Direct ELISA	Anti-HCV
Abera	2014	Cross sectional	Non probabilistic	Consecutive sampling	Monocenter	Prospetively	Ethiopia	East and Southern	Africa	Eastern Africa	Low-income economies	May/2014	Children	Hospital-based	Urban/Rural	ART naïve/ On ART	General population	HBV	Direct ELISA	HBsAg
Abreha	2011	Cross sectional	Non probabilistic	Consecutive sampling	Monocenter	Prospetively	Ethiopia	East and Southern	Africa	Eastern Africa	Low-income economies	Jun/2006-Sep/2006	Unclear/Not reported	Hospital-based	Unclear/Not reported	Unclear/Not reported	General population	HCV	Indirect ELISA	Anti-HCV
Abroug	2020	Cross sectional	Non probabilistic	Consecutive sampling	Multicenter	Prospetively	Nigeria	West and Central	Africa	West Africa	Lower-middle income economies	Nov/2017-Apr/2018	Adults	Hospital-based	Unclear/Not reported	Unclear/Not reported	Pregnant women	HBV	Immunochromatographic test	HBsAg
Adekunle	2011	Cross sectional	Non probabilistic	Consecutive sampling	Monocenter	Prospetively	Nigeria	West and Central	Africa	West Africa	Lower-middle income economies	Jan/2009-Mar/2010	Adults	Hospital-based	Unclear/Not reported	ART naïve	General population	HCV	Rapid Diagnostic test	Anti-HCV
Adekunle	2011	Cross sectional	Non probabilistic	Consecutive sampling	Monocenter	Prospetively	Nigeria	West and Central	Africa	West Africa	Lower-middle income economies	Jan/2009-Mar/2010	Adults	Hospital-based	Unclear/Not reported	ART naïve	General population	HBV	Rapid Diagnostic test	HBsAg
Adesina	2016	Case control	Non probabilistic	Consecutive sampling	Monocenter	Retrospectively	Nigeria	West and Central	Africa	West Africa	Lower-middle income economies	Jan/2006-Dec/2013	All ages	Hospital-based	Unclear/Not reported	Unclear/Not reported	Pregnant women	HCV	Indirect ELISA	Anti-HCV
Adesina	2016	Case control	Non probabilistic	Consecutive sampling	Monocenter	Retrospectively	Nigeria	West and Central	Africa	West Africa	Lower-middle income economies	Jan/2006-Dec/2013	All ages	Hospital-based	Unclear/Not reported	Unclear/Not reported	Pregnant women	HBV and HCV	Direct ELISA, Indirect ELISA	Anti-HCV, HBsAg
Adesina	2016	Case control	Non probabilistic	Consecutive sampling	Monocenter	Retrospectively	Nigeria	West and Central	Africa	West Africa	Lower-middle income economies	Jan/2006-Dec/2013	All ages	Hospital-based	Unclear/Not reported	Unclear/Not reported	Pregnant women	HBV	Direct ELISA	HBsAg
Adewole	2009	Cross sectional	Non probabilistic	Consecutive sampling	Monocenter	Retroprospectively	Nigeria	West and Central	Africa	West Africa	Lower-middle income economies	Unclear/Not reported	Adults	Hospital-based	Unclear/Not reported	Unclear/Not reported	General population	HCV	Direct ELISA	Anti-HCV
Adewole	2009	Cross sectional	Non probabilistic	Consecutive sampling	Monocenter	Retroprospectively	Nigeria	West and Central	Africa	West Africa	Lower-middle income economies	Unclear/Not reported	Adults	Hospital-based	Unclear/Not reported	Unclear/Not reported	General population	HBV	Direct ELISA	HBsAg
Adewumi	2015	Cohort (Baseline data)	Non probabilistic	Consecutive sampling	Multicenter	Prospetively	Nigeria	West and Central	Africa	West Africa	Lower-middle income economies	Sep/2010-Jan/2011	All ages	Hospital-based	Unclear/Not reported	On ART	General population	HBV	Direct ELISA	HBsAg
Adeyemi	2020	Cross sectional	Non probabilistic	Consecutive sampling	Multicenter	Prospetively	Nigeria	West and Central	Africa	West Africa	Lower-middle income economies	Jun/2017-Dec/2017	Adults	Hospital-based	Unclear/Not reported	On ART	General population	HBV	Direct ELISA	HBsAg
Adoga	2009	Cross sectional	Non probabilistic	Consecutive sampling	Monocenter	Prospetively	Nigeria	West and Central	Africa	West Africa	Lower-middle income economies	Apr/2007-May/2007	Adults	Community-based	Unclear/Not reported	Unclear/Not reported	Prisoners	HBV	Direct ELISA	HBsAg
Adoga	2009	Cross sectional	Non probabilistic	Consecutive sampling	Monocenter	Prospetively	Nigeria	West and Central	Africa	West Africa	Lower-middle income economies	Apr/2007-May/2007	Adults	Community-based	Unclear/Not reported	Unclear/Not reported	Prisoners	HCV	Enzyme immunoassay (EIA)	Anti-HCV
Agaba	2014	Cross sectional	Non probabilistic	Consecutive sampling	Monocenter	Prospetively	Nigeria	West and Central	Africa	West Africa	Lower-middle income economies	Jan/2005-Dec/2010	Adults	Hospital-based	Unclear/Not reported	ART naïve	General population	HBV	Enzyme immunoassay (EIA)	HBsAg
Agaba	2014	Cross sectional	Non probabilistic	Consecutive sampling	Monocenter	Prospetively	Nigeria	West and Central	Africa	West Africa	Lower-middle income economies	Jan/2005-Dec/2010	Adults	Hospital-based	Unclear/Not reported	ART naïve	General population	HCV	Enzyme immunoassay (EIA)	Anti-HCV
Agbaji	2013	Cohort (Baseline data)	Non probabilistic	Consecutive sampling	Monocenter	Prospetively	Nigeria	West and Central	Africa	West Africa	Lower-middle income economies	Oct/2004-Jun/2006	Unclear/Not reported	Hospital-based	Unclear/Not reported	On ART	General population	HCV	Direct ELISA	Anti-HCV
Agbaji	2013	Cohort (Baseline data)	Non probabilistic	Consecutive sampling	Monocenter	Prospetively	Nigeria	West and Central	Africa	West Africa	Lower-middle income economies	Oct/2004-Jun/2006	Unclear/Not reported	Hospital-based	Unclear/Not reported	On ART	General population	HCV	Direct ELISA	Anti-HCV
Agwale	2004	Cross sectional	Non probabilistic	Convenience sampling	Monocenter	Retrospectively	Nigeria	West and Central	Africa	West Africa	Lower-middle income economies	Oct/2004-Jun/2006	Adults	Hospital-based	Unclear/Not reported	On ART	General population	HCV	Transcription-mediated amplification assay	Viral RNA
Ahmed	1998	Cross sectional	Non probabilistic	Consecutive sampling	Monocenter	Prospetively	Malawi	East and Southern	Africa	Eastern Africa	Low-income economies	1993-1995	Adults	Hospital-based	Rural	Unclear/Not reported	Pregnant women	HBV	Direct ELISA	HBsAg
Ahmed	1998	Cross sectional	Non probabilistic	Consecutive sampling	Monocenter	Prospetively	Malawi	East and Southern	Africa	Eastern Africa	Low-income economies	1993-1995	Adults	Hospital-based	Rural	Unclear/Not reported	Pregnant women	HBV	Direct ELISA	HBeAg
Ahmed	1998	Cross sectional	Non probabilistic	Consecutive sampling	Monocenter	Prospetively	Malawi	East and Southern	Africa	Eastern Africa	Low-income economies	1993-1995	Adults	Hospital-based	Rural	Unclear/Not reported	Pregnant women	HCV	Indirect ELISA	Anti-HCV
Ajayi	2011	Cross sectional	Non probabilistic	Consecutive sampling	Monocenter	Prospetively	Nigeria	West and Central	Africa	West Africa	Lower-middle income economies	Jan/2009- Mar/2010	Adults	Hospital-based	Unclear/Not reported	ART naïve	General population	HCV	Rapid Diagnostic test	Anti-HCV
Ajayi	2011	Cross sectional	Non probabilistic	Consecutive sampling	Monocenter	Prospetively	Nigeria	West and Central	Africa	West Africa	Lower-middle income economies	Jan/2009- Mar/2010	Adults	Hospital-based	Unclear/Not reported	ART naïve	General population	HBV	Rapid Diagnostic test	HBsAg
Akinbami	2020	Cross sectional	Non probabilistic	Consecutive sampling	Monocenter	Prospetively	Nigeria	West and Central	Africa	West Africa	Lower-middle income economies	Jan/2010-Mar/2010	Unclear/Not reported	Hospital-based	Unclear/Not reported	Unclear/Not reported	General population	HCV	Rapid Diagnostic test	Anti-HCV
Akinniyi	2021	Cross sectional	Non probabilistic	Consecutive sampling	Monocenter	Prospetively	Nigeria	West and Central	Africa	West Africa	Lower-middle income economies	Unclear/Not reported	All ages	Hospital-based	Urban	Unclear/Not reported	General population	HBV	Immunochromatographic test	HBsAg
Akinniyi	2021	Cross sectional	Non probabilistic	Consecutive sampling	Monocenter	Prospetively	Nigeria	West and Central	Africa	West Africa	Lower-middle income economies	Unclear/Not reported	All ages	Hospital-based	Urban	Unclear/Not reported	General population	HBV	Immunochromatographic test	HBeAg
Alemayehu	2011	Cross sectional	Non probabilistic	Consecutive sampling	Monocenter	Prospetively	Ethiopia	East and Southern	Africa	Eastern Africa	Low-income economies	Oct/2008-Dec/2008	Adults	Hospital-based	Urban/Rural	On ART	General population	HCV	Indirect ELISA	Anti-HCV
Alidjinou	2014	Cross sectional	Non probabilistic	Consecutive sampling	Monocenter	Prospetively	Republic of the Congo	West and Central	Africa	Central Africa	Lower-middle income economies	Unclear/Not reported	Unclear/Not reported	Hospital-based	Unclear/Not reported	Unclear/Not reported	Blood donors	HCV	Indirect ELISA	Anti-HCV
Ambassa	2016	Cross sectional	Non probabilistic	Consecutive sampling	Monocenter	Prospetively	Cameroon	West and Central	Africa	Central Africa	Lower-middle income economies	Nov/2015-Feb/2016	Adults	Hospital-based	Unclear/Not reported	Unclear/Not reported	General population	HBV	Immunochromatographic test	HBsAg
Amstutz	2020	Cross sectional	Non probabilistic	Consecutive sampling	Multicenter	Prospetively	Lesotho	East and Southern	Africa	Southern Africa	Lower-middle income economies	May/2014-Jun/2014	Children	Hospital-based	Unclear/Not reported	On ART	General population	HCV	Rapid Diagnostic test	Anti-HCV
Amstutz	2020	Cross sectional	Non probabilistic	Consecutive sampling	Multicenter	Prospetively	Lesotho	East and Southern	Africa	Southern Africa	Lower-middle income economies	May/2014-Jun/2014	Children	Hospital-based	Unclear/Not reported	On ART	General population	HBV	Rapid Diagnostic test	HBsAg
Amstutz	2020	Cross sectional	Non probabilistic	Consecutive sampling	Multicenter	Prospetively	Lesotho	East and Southern	Africa	Southern Africa	Lower-middle income economies	May/2014-Jun/2014	Adults	Hospital-based	Unclear/Not reported	On ART	General population	HCV	Rapid Diagnostic test	Anti-HCV
Amstutz	2020	Cross sectional	Non probabilistic	Consecutive sampling	Multicenter	Prospetively	Lesotho	East and Southern	Africa	Southern Africa	Lower-middle income economies	May/2014-Jun/2014	Adults	Hospital-based	Unclear/Not reported	On ART	General population	HBV	Rapid Diagnostic test	HBsAg
Andersson	2013	Cross sectional	Non probabilistic	Consecutive sampling	Monocenter	Retrospectively	South Africa	East and Southern	Africa	Southern Africa	Upper-middle-income economies	2008	All ages	Hospital-based	Unclear/Not reported	Unclear/Not reported	Pregnant women	HBV	Neutralization test	HBsAg
Andersson	2012	Cross sectional	Non probabilistic	Consecutive sampling	Monocenter	Prospetively	South Africa	East and Southern	Africa	Southern Africa	Upper-middle-income economies	Jul/2008- Oct/2009	Adults	Hospital-based	Unclear/Not reported	Unclear/Not reported	Pregnant women	HBV	Neutralization test	HBsAg
Andreotti	2014	Cohort (Baseline data)	Non probabilistic	Consecutive sampling	Monocenter	Prospetively	Malawi	East and Southern	Africa	Eastern Africa	Low-income economies	Feb/2008-Feb/2009	Adults	Hospital-based	Unclear/Not reported	Unclear/Not reported	Pregnant women	HCV	Direct ELISA	Anti-HCV
Andreotti	2014	Cohort (Baseline data)	Non probabilistic	Consecutive sampling	Monocenter	Prospetively	Malawi	East and Southern	Africa	Eastern Africa	Low-income economies	Feb/2008-Feb/2009	Adults	Hospital-based	Unclear/Not reported	Unclear/Not reported	Pregnant women	HBV	Direct ELISA	HBsAg
Andreotti	2014	Cohort (Baseline data)	Non probabilistic	Consecutive sampling	Monocenter	Prospetively	Malawi	East and Southern	Africa	Eastern Africa	Low-income economies	Feb/2008-Feb/2009	Adults	Hospital-based	Unclear/Not reported	Unclear/Not reported	Pregnant women	HCV	Rapid Diagnostic test	Viral RNA
Anigilaje	2013	Cross sectional	Non probabilistic	Consecutive sampling	Monocenter	Prospetively	Nigeria	West and Central	Africa	West Africa	Lower-middle income economies	Jun/2008-Jun/2012	Children	Hospital-based	Unclear/Not reported	On ART	General population	HBV	Direct ELISA	HBsAg
Anigilaje	2013	Cross sectional	Non probabilistic	Consecutive sampling	Monocenter	Prospetively	Nigeria	West and Central	Africa	West Africa	Lower-middle income economies	Jun/2008-Jun/2012	Children	Hospital-based	Unclear/Not reported	On ART	General population	HCV	Indirect ELISA	Anti-HCV
Anigilaje	2013	Cross sectional	Non probabilistic	Consecutive sampling	Monocenter	Prospetively	Nigeria	West and Central	Africa	West Africa	Lower-middle income economies	Jun/2008-Jun/2012	Children	Hospital-based	Unclear/Not reported	On ART	General population	HBV and HCV	Direct ELISA, Indirect ELISA	Anti-HCV, HBsAg
Anyanwu	2020	Cross sectional	Probabilistic	Simple random sampling	Multicenter	Prospetively	Nigeria	West and Central	Africa	West Africa	Lower-middle income economies	Feb/2019-Jul/2019	Adults	Hospital-based	Urban/Rural	Unclear/Not reported	General population	HCV	Indirect ELISA	Anti-HCV
Anyanwu	2020	Cross sectional	Probabilistic	Simple random sampling	Multicenter	Prospetively	Nigeria	West and Central	Africa	West Africa	Lower-middle income economies	Feb/2019-Jul/2019	Adults	Hospital-based	Urban/Rural	Unclear/Not reported	General population	HBV and HCV	Direct ELISA, Indirect ELISA	Anti-HCV, HBsAg
Anyanwu	2020	Cross sectional	Probabilistic	Simple random sampling	Multicenter	Prospetively	Nigeria	West and Central	Africa	West Africa	Lower-middle income economies	Feb/2019-Jul/2019	Adults	Hospital-based	Urban/Rural	Unclear/Not reported	General population	HBV	Direct ELISA	HBsAg
Archampong	2016	Cross sectional	Non probabilistic	Consecutive sampling	Monocenter	Prospetively	Ghana	West and Central	Africa	West Africa	Lower-middle income economies	2012–2014	Unclear/Not reported	Hospital-based	Unclear/Not reported	ART naïve	General population	HBV	Direct ELISA	HBsAg
Ashir	2009	Cross sectional	Non probabilistic	Consecutive sampling	Monocenter	Prospetively	Nigeria	West and Central	Africa	West Africa	Lower-middle income economies	Sep/2007-Dec/2007	Children	Hospital-based	Unclear/Not reported	ART naïve	General population	HBV	Direct ELISA	HBsAg
Atina	2004	Cross sectional	Non probabilistic	Consecutive sampling	Monocenter	Prospetively	Kenya	East and Southern	Africa	Eastern Africa	Lower-middle income economies	2000	All ages	Hospital-based	Unclear/Not reported	Unclear/Not reported	General population	HCV	Indirect ELISA	Anti-HCV
Attia	2012	Cross sectional	Non probabilistic	Consecutive sampling	Multicenter	Prospetively	Ivory Coast	West and Central	Africa	West Africa	Lower-middle income economies	Jun/2006-Sep/2006	Adults	Hospital-based	Unclear/Not reported	ART naïve	General population	HBV	Immunoassay kit	HBsAg
Augusto	2019	Cross sectional	Probabilistic	Simple random sampling	Multicenter	Prospetively	Mozambique	East and Southern	Africa	Eastern Africa	Low-income economies	Jul/2011-Aug/2012	Adults	Community-based	Unclear/Not reported	Unclear/Not reported	Prisoners	HBV	Direct ELISA	HBsAg
Ayana	2019	Case control	Non probabilistic	Consecutive sampling	Multicenter	Prospetively	Ethiopia	East and Southern	Africa	Eastern Africa	Low-income economies	Sept/2017- Feb/2018	Adults	Hospital-based	Unclear/Not reported	On ART	General population	HBV	Direct ELISA	HBsAg
Ayele	2002	Cross sectional	Non probabilistic	Convenience sampling	Monocenter	Retrospectively	Ethiopia	East and Southern	Africa	Eastern Africa	Low-income economies	1994	All ages	Community-based	Unclear/Not reported	Unclear/Not reported	General population	HCV	Direct ELISA	Anti-HCV
Ayelign	2021	Cross sectional	Non probabilistic	Convenience sampling	Monocenter	Prospetively	Ethiopia	East and Southern	Africa	Eastern Africa	Low-income economies	Unclear/Not reported	Adults	Hospital-based	Unclear/Not reported	Unclear/Not reported	General population	HBV	Rapid Diagnostic test	HBsAg
Ayelign	2021	Cross sectional	Non probabilistic	Convenience sampling	Monocenter	Prospetively	Ethiopia	East and Southern	Africa	Eastern Africa	Low-income economies	Unclear/Not reported	Adults	Hospital-based	Unclear/Not reported	Unclear/Not reported	General population	HCV	Rapid Diagnostic test	Anti-HCV
Ayuk	2013	Cross sectional	Non probabilistic	Consecutive sampling	Multicenter	Retrospectively	South Africa	East and Southern	Africa	Southern Africa	Upper-middle-income economies	2008	Adults	Hospital-based	Unclear/Not reported	ART naïve	General population	HBV	Direct ELISA	HBsAg
Bado	2013	Cross sectional	Non probabilistic	Consecutive sampling	Monocenter	Prospetively	Burkina Faso	West and Central	Africa	West Africa	Low-income economies	Oct/2008-Dec/2008	Unclear/Not reported	Hospital-based	Unclear/Not reported	ART naïve/ On ART	General population	HBV	Direct ELISA	HBsAg
Balew	2014	Cross sectional	Probabilistic	Systematic sampling	Monocenter	Prospetively	Ethiopia	East and Southern	Africa	Eastern Africa	Low-income economies	Feb/2012-Apr/2012	Unclear/Not reported	Hospital-based	Unclear/Not reported	Unclear/Not reported	General population	HBV	Rapid Diagnostic test	HBsAg
Balew	2014	Cross sectional	Probabilistic	Systematic sampling	Monocenter	Prospetively	Ethiopia	East and Southern	Africa	Eastern Africa	Low-income economies	Feb/2012-Apr/2012	Unclear/Not reported	Hospital-based	Unclear/Not reported	Unclear/Not reported	General population	HCV	Rapid Diagnostic test	Anti-HCV
Balew	2014	Cross sectional	Probabilistic	Systematic sampling	Monocenter	Prospetively	Ethiopia	East and Southern	Africa	Eastern Africa	Low-income economies	Feb/2012-Apr/2012	Unclear/Not reported	Hospital-based	Unclear/Not reported	Unclear/Not reported	General population	HBV and HCV	Rapid Diagnostic test	Anti-HCV, HBsAg
Balogun	2010	Cross sectional	Non probabilistic	Consecutive sampling	Monocenter	Prospetively	Nigeria	West and Central	Africa	West Africa	Lower-middle income economies	Mar/2006-Aug/2006	Adults	Hospital-based	Unclear/Not reported	Unclear/Not reported	General population	HBV	Direct ELISA	HBsAg
Balogun	2012	Cross sectional	Non probabilistic	Consecutive sampling	Monocenter	Prospetively	Nigeria	West and Central	Africa	West Africa	Lower-middle income economies	Mar/2006-Aug/2006	Adults	Hospital-based	Unclear/Not reported	On ART	General population	HCV	Indirect ELISA	Anti-HCV
Balogun	2012	Cross sectional	Non probabilistic	Consecutive sampling	Monocenter	Prospetively	Nigeria	West and Central	Africa	West Africa	Lower-middle income economies	Mar/2006-Aug/2006	Adults	Hospital-based	Unclear/Not reported	On ART	General population	HBV and HCV	Direct ELISA, Indirect ELISA	Anti-HCV, HBsAg
Balogun	2012	Cross sectional	Non probabilistic	Consecutive sampling	Monocenter	Prospetively	Nigeria	West and Central	Africa	West Africa	Lower-middle income economies	Mar/2006-Aug/2006	Adults	Hospital-based	Unclear/Not reported	On ART	General population	HBV	Direct ELISA	HBsAg
Barros	1996	Cross sectional	Non probabilistic	Consecutive sampling	Monocenter	Prospetively	Guinea-Bissau	West and Central	Africa	West Africa	Low-income economies	Unclear/Not reported	Adults	Community-based	Unclear/Not reported	Unclear/Not reported	General population	HBV	Direct ELISA	HBsAg
Barth	2011	Cross sectional	Non probabilistic	Consecutive sampling	Monocenter	Prospetively	South Africa	East and Southern	Africa	Southern Africa	Upper-middle-income economies	Sep/2008-Nov/2008	Unclear/Not reported	Hospital-based	Rural	ART naïve	General population	HCV	Electro-chemiluminescence immunoassay (ECLIA)	Anti-HCV
Barth	2011	Cross sectional	Non probabilistic	Consecutive sampling	Monocenter	Prospetively	South Africa	East and Southern	Africa	Southern Africa	Upper-middle-income economies	Sep/2008-Nov/2008	Unclear/Not reported	Hospital-based	Rural	ART naïve	General population	HBV	Electro-chemiluminescence immunoassay (ECLIA)	HBsAg
Baseke	2015	Cross sectional	Probabilistic	Systematic sampling	Monocenter	Prospetively	Uganda	East and Southern	Africa	Eastern Africa	Low-income economies	2007	All ages	Hospital-based	Unclear/Not reported	On ART	General population	HCV	Indirect ELISA	Anti-HCV
Baseke	2015	Cross sectional	Probabilistic	Systematic sampling	Monocenter	Prospetively	Uganda	East and Southern	Africa	Eastern Africa	Low-income economies	2007	All ages	Hospital-based	Unclear/Not reported	On ART	General population	HBV and HCV	Direct ELISA, Indirect ELISA	Anti-HCV, HBsAg
Baseke	2015	Cross sectional	Probabilistic	Systematic sampling	Monocenter	Prospetively	Uganda	East and Southern	Africa	Eastern Africa	Low-income economies	2007	All ages	Hospital-based	Unclear/Not reported	On ART	General population	HBV	Direct ELISA	HBsAg
Beghin	2017	Cross sectional	Non probabilistic	Consecutive sampling	Monocenter	Prospetively	South Africa	East and Southern	Africa	Southern Africa	Upper-middle-income economies	Sep/2014-Dec/2014	Children	Hospital-based	Unclear/Not reported	Unclear/Not reported	General population	HBV	Electro-chemiluminescence immunoassay (ECLIA)	HBsAg
Bell	2012	Cross sectional	Non probabilistic	Consecutive sampling	Monocenter	Prospetively	South Africa	East and Southern	Africa	Southern Africa	Upper-middle-income economies	Jul/2009-Nov/2009	Unclear/Not reported	Hospital-based	Rural	ART naïve	General population	HBV	Direct ELISA	HBeAg
Bell	2012	Cross sectional	Non probabilistic	Consecutive sampling	Monocenter	Prospetively	South Africa	East and Southern	Africa	Southern Africa	Upper-middle-income economies	Jul/2009-Nov/2009	Unclear/Not reported	Hospital-based	Rural	ART naïve	General population	HBV	Direct ELISA	HBsAg
Benjelloun	1996	Cross sectional	Non probabilistic	Consecutive sampling	Monocenter	Prospetively	Morocco	North Africa and Middle East	Eastern Mediterranean	Northern Africa	Lower-middle income economies	Unclear/Not reported	Adults	Hospital-based	Unclear/Not reported	Unclear/Not reported	General population	HCV	Indirect ELISA	Anti-HCV
Bessimbaye	2014	Cross sectional	Non probabilistic	Consecutive sampling	Monocenter	Prospetively	Chad	West and Central	Africa	Central Africa	Low-income economies	2012	All ages	Hospital-based	Unclear/Not reported	Unclear/Not reported	General population	HCV	Enzyme immunoassay (EIA)	Anti-HCV
Bessimbaye	2014	Cross sectional	Non probabilistic	Consecutive sampling	Monocenter	Prospetively	Chad	West and Central	Africa	Central Africa	Low-income economies	2012	All ages	Hospital-based	Unclear/Not reported	Unclear/Not reported	General population	HBV and HCV	Enzyme immunoassay (EIA), Rapid Diagnostic test	Anti-HCV, HBsAg
Bessimbaye	2014	Cross sectional	Non probabilistic	Consecutive sampling	Monocenter	Prospetively	Chad	West and Central	Africa	Central Africa	Low-income economies	2012	All ages	Hospital-based	Unclear/Not reported	Unclear/Not reported	General population	HBV	Rapid Diagnostic test	HBsAg
Bhattacharya	2021	Clinical Trial (Baseline data)	Probabilistic	Simple random sampling	Monocenter	Retrospectively	South Africa, Tanzania	East and Southern	Africa	Eastern Africa, Southern Africa	Low-income economies, Lower-middle-in	Unclear/Not reported	Unclear/Not reported	Hospital-based	Unclear/Not reported	ART naïve/ On ART	Pregnant women	HBV	Enzyme immunoassay (EIA)	HBeAg
Bhattacharya	2021	Clinical Trial (Baseline data)	Probabilistic	Simple random sampling	Monocenter	Retrospectively	South Africa, Tanzania	East and Southern	Africa	Eastern Africa, Southern Africa	Low-income economies, Lower-middle-in	Unclear/Not reported	Unclear/Not reported	Hospital-based	Unclear/Not reported	ART naïve/ On ART	Pregnant women	HBV	Enzyme immunoassay (EIA)	HBsAg
Bivigou-Mboumba	2016	Cross sectional	Non probabilistic	Consecutive sampling	Monocenter	Prospetively	Gabon	West and Central	Africa	Central Africa	Upper-middle-income economies	Mar/2010-Jan/2013	Unclear/Not reported	Hospital-based	Unclear/Not reported	ART naïve/ On ART	General population	HCV	Indirect ELISA	Anti-HCV
Boateng	2019	Cross sectional	Probabilistic	Simple random sampling	Monocenter	Prospetively	Ghana	West and Central	Africa	West Africa	Lower-middle income economies	May/2016-Apr/2017	Adults	Hospital-based	Urban	ART naïve	General population	HCV	Rapid Diagnostic test	Anti-HCV
Boateng	2019	Cross sectional	Probabilistic	Simple random sampling	Monocenter	Prospetively	Ghana	West and Central	Africa	West Africa	Lower-middle income economies	May/2016-Apr/2017	Adults	Hospital-based	Urban	ART naïve	General population	HBV	Rapid Diagnostic test	HBsAg
Bouare	2012	Cross sectional	Non probabilistic	Consecutive sampling	Monocenter	Prospetively	Mali	West and Central	Africa	West Africa	Low-income economies	Unclear/Not reported	Adults	Hospital-based	Unclear/Not reported	Unclear/Not reported	General population	HCV	Enzyme immunoassay (EIA)	Anti-HCV
Bouare	2012	Cross sectional	Non probabilistic	Consecutive sampling	Monocenter	Prospetively	Mali	West and Central	Africa	West Africa	Low-income economies	Unclear/Not reported	Adults	Hospital-based	Unclear/Not reported	Unclear/Not reported	Pregnant women	HCV	Enzyme immunoassay (EIA)	Anti-HCV
Bowring	2013	Cross sectional	Probabilistic	Snowball sampling	Multicenter	Prospetively	Tanzania	East and Southern	Africa	Eastern Africa	Lower-middle income economies	Jun/2011	Adults	Community-based	Unclear/Not reported	Unclear/Not reported	General population	HCV	Rapid Diagnostic test	Anti-HCV
Bowring	2013	Cross sectional	Probabilistic	Snowball sampling	Multicenter	Prospetively	Tanzania	East and Southern	Africa	Eastern Africa	Lower-middle income economies	Jun/2011	Adults	Community-based	Unclear/Not reported	Unclear/Not reported	Injecting drug users	HCV	Rapid Diagnostic test	Anti-HCV
Brandful	1999	Cross sectional	Non probabilistic	Consecutive sampling	Multicenter	Prospetively	Ghana	West and Central	Africa	West Africa	Lower-middle income economies	Apr/1996-Jul/1997	Adults	Hospital-based	Unclear/Not reported	Unclear/Not reported	General population	HCV	Unclear/Not reported	Anti-HCV
Brandful	1999	Cross sectional	Non probabilistic	Consecutive sampling	Multicenter	Prospetively	Ghana	West and Central	Africa	West Africa	Lower-middle income economies	Apr/1996-Jul/1997	Adults	Hospital-based	Unclear/Not reported	Unclear/Not reported	General population	HBV	Unclear/Not reported	HBsAg
Burnett	2007	Case control	Non probabilistic	Consecutive sampling	Monocenter	Retrospectively	South Africa	East and Southern	Africa	Southern Africa	Upper-middle-income economies	1999-2001	Unclear/Not reported	Hospital-based	Unclear/Not reported	Unclear/Not reported	Pregnant women	HBV	Classical PCR	Viral DNA
Burnett	2007	Case control	Non probabilistic	Consecutive sampling	Monocenter	Retrospectively	South Africa	East and Southern	Africa	Southern Africa	Upper-middle-income economies	1999-2001	Unclear/Not reported	Hospital-based	Unclear/Not reported	Unclear/Not reported	Pregnant women	HBV	Auszyme Assay	HBsAg
Buseri	2009	Cross sectional	Non probabilistic	Consecutive sampling	Monocenter	Prospetively	Nigeria	West and Central	Africa	West Africa	Lower-middle income economies	Sep/2007-Jan/2008	Adults	Hospital-based	Unclear/Not reported	Unclear/Not reported	Blood donors	HCV	Rapid Diagnostic test	Anti-HCV
Buseri	2009	Cross sectional	Non probabilistic	Consecutive sampling	Monocenter	Prospetively	Nigeria	West and Central	Africa	West Africa	Lower-middle income economies	Sep/2007-Jan/2008	Adults	Hospital-based	Unclear/Not reported	Unclear/Not reported	Blood donors	HBV	Rapid Diagnostic test	HBsAg
Calisti	2015	Cross sectional	Non probabilistic	Consecutive sampling	Monocenter	Prospetively	Uganda	East and Southern	Africa	Eastern Africa	Low-income economies	Jun/2009-Dec/2011	Adults	Hospital-based	Unclear/Not reported	On ART	General population	HBV	Rapid Diagnostic test	HBsAg
Carimo	2018	Cross sectional	Non probabilistic	Consecutive sampling	Multicenter	Prospetively	Mozambique	East and Southern	Africa	Eastern Africa	Low-income economies	Jun/2012-Oct/2012	Adults	Hospital-based	Unclear/Not reported	ART naïve	General population	HBV	Rapid Diagnostic test	HBsAg
Chakraborty	2003	Cross sectional	Non probabilistic	Consecutive sampling	Monocenter	Prospetively	Kenya	East and Southern	Africa	Eastern Africa	Lower-middle income economies	2000	Children	Community-based	Unclear/Not reported	ART naïve	General population	HBV	Enzyme immunoassay (EIA)	HBsAg
Chakraborty	2003	Cross sectional	Non probabilistic	Consecutive sampling	Monocenter	Prospetively	Kenya	East and Southern	Africa	Eastern Africa	Lower-middle income economies	2000	Children	Community-based	Unclear/Not reported	ART naïve	General population	HCV	Classical RT-PCR	Viral RNA
Chambal	2017	Cross sectional	Non probabilistic	Consecutive sampling	Multicenter	Prospetively	Mozambique	East and Southern	Africa	Eastern Africa	Low-income economies	May/2012-Nov/2012	Adults	Hospital-based	Unclear/Not reported	ART naïve	General population	HBV	Chemiluminescent enzyme immunoassay (CLEIA)	HBsAg
Chasela	2012	Cross sectional	Non probabilistic	Consecutive sampling	Monocenter	Prospetively	Malawi	East and Southern	Africa	Eastern Africa	Low-income economies	Unclear/Not reported	All ages	Hospital-based	Unclear/Not reported	ART naïve/ On ART	Pregnant women	HBV	Chemiluminescent enzyme immunoassay (CLEIA)	HBsAg
Cherry	2010	Cross sectional	Non probabilistic	Consecutive sampling	Monocenter	Prospetively	South Africa	East and Southern	Africa	Southern Africa	Upper-middle-income economies	2008-2009	Adults	Hospital-based	Unclear/Not reported	Unclear/Not reported	General population	HCV	Enzyme immunoassay (EIA)	Anti-HCV
Chiesa	2020	Cross sectional	Probabilistic	Systematic sampling	Monocenter	Prospetively	Uganda	East and Southern	Africa	Eastern Africa	Low-income economies	Apr/2016-Jun/2016	All ages	Hospital-based	Unclear/Not reported	On ART	General population	HBV	Rapid Diagnostic test	HBeAg
Chisenga	2018	Cohort (Baseline data)	Non probabilistic	Consecutive sampling	Multicenter	Prospetively	Zambia	East and Southern	Africa	Eastern Africa	Lower-middle income economies	2013–2014	Adults	Hospital-based	Unclear/Not reported	On ART	General population	HBV	Direct ELISA	HBsAg
Chisenga	2018	Cohort (Baseline data)	Non probabilistic	Consecutive sampling	Multicenter	Prospetively	Zambia	East and Southern	Africa	Eastern Africa	Lower-middle income economies	2013–2014	Adults	Hospital-based	Unclear/Not reported	On ART	General population	HBV	Rapid Diagnostic test	HBsAg
Coffie	2017	Cross sectional	Probabilistic	Simple random sampling	Multicenter	Retrospectively	Ivory Coast, Benin, Bu	East and Southern, West and Central	Africa	Central Africa, Eastern Africa, S	Low-income economies, Lower-middle-in	Unclear/Not reported	Unclear/Not reported	Hospital-based	Unclear/Not reported	On ART	General population	HBV	Serological tests	HBsAg
Coffie	2017	Cross sectional	Non probabilistic	Consecutive sampling	Multicenter	Prospetively	Burkina Faso	West and Central	Africa	West Africa	Low-income economies	Mar/2012-Dec/2012	Unclear/Not reported	Hospital-based	Unclear/Not reported	On ART	General population	HBV	Enzyme immunoassay (EIA)	HBsAg
Coffie	2017	Cross sectional	Non probabilistic	Consecutive sampling	Multicenter	Prospetively	Ivory Coast	West and Central	Africa	West Africa	Lower-middle income economies	Mar/2012-Dec/2012	Unclear/Not reported	Hospital-based	Unclear/Not reported	On ART	General population	HBV	Enzyme immunoassay (EIA)	HBsAg
Coffie	2017	Cross sectional	Non probabilistic	Consecutive sampling	Multicenter	Prospetively	Mali	West and Central	Africa	West Africa	Low-income economies	Mar/2012-Dec/2012	Unclear/Not reported	Hospital-based	Unclear/Not reported	On ART	General population	HBV	Enzyme immunoassay (EIA)	HBsAg
Collenberg	2006	Cross sectional	Non probabilistic	Consecutive sampling	Multicenter	Prospetively	Burkina Faso	West and Central	Africa	West Africa	Low-income economies	Jul/2003-Feb/2004	Adults	Hospital-based	Unclear/Not reported	Unclear/Not reported	General population	HBV	Enzyme immunoassay (EIA)	HBsAg
Combe	2001	Cross sectional	Non probabilistic	Consecutive sampling	Multicenter	Prospetively	Ivory Coast	West and Central	Africa	West Africa	Lower-middle income economies	Apr/1995-Feb/1996	Adults	Hospital-based	Unclear/Not reported	Unclear/Not reported	General population	HBV	Direct ELISA	HBeAg
Combe	2001	Cross sectional	Non probabilistic	Consecutive sampling	Multicenter	Prospetively	Ivory Coast	West and Central	Africa	West Africa	Lower-middle income economies	Apr/1995-Feb/1996	Adults	Hospital-based	Unclear/Not reported	Unclear/Not reported	General population	HBV	Direct ELISA	HBsAg
Combe	2001	Cross sectional	Non probabilistic	Consecutive sampling	Multicenter	Prospetively	Ivory Coast	West and Central	Africa	West Africa	Lower-middle income economies	Apr/1995-Feb/1996	Adults	Hospital-based	Unclear/Not reported	Unclear/Not reported	General population	HCV	Indirect ELISA	Anti-HCV
Croce	2007	Cross sectional	Non probabilistic	Consecutive sampling	Monocenter	Prospetively	Tanzania	East and Southern	Africa	Eastern Africa	Lower-middle income economies	Nov/2002-Dec/2002	Adults	Hospital-based	Unclear/Not reported	Unclear/Not reported	General population	HBV	Enzyme immunoassay (EIA)	HBsAg
Cunha	2007	Cross sectional	Non probabilistic	Consecutive sampling	Monocenter	Prospetively	Mozambique	East and Southern	Africa	Eastern Africa	Low-income economies	Apr/2004-Jul/2004	Adults	Hospital-based	Unclear/Not reported	Unclear/Not reported	Blood donors	HCV	Indirect ELISA	Anti-HCV
Cunha	2007	Cross sectional	Non probabilistic	Consecutive sampling	Monocenter	Prospetively	Mozambique	East and Southern	Africa	Eastern Africa	Low-income economies	Apr/2004-Jul/2004	Adults	Hospital-based	Unclear/Not reported	Unclear/Not reported	Blood donors	HBV	Direct ELISA	HBsAg
Dahoma	2011	Cross sectional	Non probabilistic	Consecutive sampling	Monocenter	Prospetively	Tanzania	East and Southern	Africa	Eastern Africa	Lower-middle income economies	Apr/2007-Jun/2007	Adults	Community-based	Unclear/Not reported	Unclear/Not reported	General population	HCV	Rapid Diagnostic test	Anti-HCV
Dahoma	2011	Cross sectional	Non probabilistic	Consecutive sampling	Monocenter	Prospetively	Tanzania	East and Southern	Africa	Eastern Africa	Lower-middle income economies	Apr/2007-Jun/2007	Adults	Community-based	Unclear/Not reported	Unclear/Not reported	Men who have sex with men	HBV	Rapid Diagnostic test	HBsAg
Day	2013	Cohort (Baseline data)	Non probabilistic	Consecutive sampling	Monocenter	Prospetively	Kenya	East and Southern	Africa	Eastern Africa	Lower-middle income economies	Mar/2004-Mar/2010	Unclear/Not reported	Hospital-based	Unclear/Not reported	On ART	General population	HBV	ADVIA Centaur chemiluminometric immunoassay system	HBsAg
Day	2013	Cohort (Baseline data)	Non probabilistic	Consecutive sampling	Monocenter	Prospetively	Kenya	East and Southern	Africa	Eastern Africa	Lower-middle income economies	Mar/2004-Mar/2010	Unclear/Not reported	Hospital-based	Unclear/Not reported	On ART	General population	CFR	ADVIA Centaur chemiluminometric immunoassay system	HBsAg
Day	2013	Cohort (Baseline data)	Non probabilistic	Consecutive sampling	Monocenter	Prospetively	Kenya	East and Southern	Africa	Eastern Africa	Lower-middle income economies	Mar/2004-Mar/2010	Unclear/Not reported	Hospital-based	Unclear/Not reported	On ART	General population	HBV	Direct ELISA	HBeAg
Demir	2018	Cohort (Baseline data)	Non probabilistic	Consecutive sampling	Monocenter	Prospetively	Malawi	East and Southern	Africa	Eastern Africa	Low-income economies	Unclear/Not reported	Unclear/Not reported	Hospital-based	Unclear/Not reported	On ART	General population	HBV	Enzyme immunoassay (EIA)	HBsAg
Deressa	2017	Cross sectional	Probabilistic	Systematic sampling	Monocenter	Prospetively	Ethiopia	East and Southern	Africa	Eastern Africa	Low-income economies	Mar/2016-Jul/2016	Adults	Hospital-based	Unclear/Not reported	ART naïve/ On ART	General population	HBV	Enzyme immunoassay (EIA)	HBsAg
Diale	2015	Cross sectional	Non probabilistic	Consecutive sampling	Monocenter	Retrospectively	South Africa	East and Southern	Africa	Southern Africa	Upper-middle-income economies	Jan/2008-Dec/2013	All ages	Hospital-based	Unclear/Not reported	Unclear/Not reported	Pregnant women	HBV	Enzyme immunoassay (EIA)	HBeAg
Diale	2015	Cross sectional	Non probabilistic	Consecutive sampling	Monocenter	Retrospectively	South Africa	East and Southern	Africa	Southern Africa	Upper-middle-income economies	Jan/2008-Dec/2013	All ages	Hospital-based	Unclear/Not reported	Unclear/Not reported	Pregnant women	HBV	Enzyme immunoassay (EIA)	HBsAg
Diarra	2006	Case control	Non probabilistic	Consecutive sampling	Monocenter	Prospetively	Mali	West and Central	Africa	West Africa	Low-income economies	Unclear/Not reported	Unclear/Not reported	Hospital-based	Unclear/Not reported	Unclear/Not reported	General population	HCV	Indirect ELISA	Anti-HCV
Diarra	2006	Case control	Non probabilistic	Consecutive sampling	Monocenter	Prospetively	Mali	West and Central	Africa	West Africa	Low-income economies	Unclear/Not reported	Unclear/Not reported	Hospital-based	Unclear/Not reported	Unclear/Not reported	General population	HBV	Direct ELISA	HBsAg
Diop-Ndiaye	2008	Clinical Trial (Baseline data)	Probabilistic	Simple random sampling	Monocenter	Retrospectively	Senegal	West and Central	Africa	West Africa	Lower-middle income economies	Aug/1998-Apr/2002	Adults	Hospital-based	Unclear/Not reported	On ART	General population	HCV	Indirect ELISA	Anti-HCV
Diop-Ndiaye	2008	Clinical Trial (Baseline data)	Probabilistic	Simple random sampling	Monocenter	Retrospectively	Senegal	West and Central	Africa	West Africa	Lower-middle income economies	Aug/1998-Apr/2002	Adults	Hospital-based	Unclear/Not reported	On ART	General population	HBV and HCV	Direct ELISA, Indirect ELISA	Anti-HCV, HBsAg
Diop-Ndiaye	2008	Clinical Trial (Baseline data)	Probabilistic	Simple random sampling	Monocenter	Retrospectively	Senegal	West and Central	Africa	West Africa	Lower-middle income economies	Aug/1998-Apr/2002	Adults	Hospital-based	Unclear/Not reported	On ART	General population	HBV	Direct ELISA	HBsAg
Diro	2008	Cross sectional	Non probabilistic	Consecutive sampling	Monocenter	Prospetively	Ethiopia	East and Southern	Africa	Eastern Africa	Low-income economies	Apr/2004-Jul/2004	Adults	Hospital-based	Unclear/Not reported	Unclear/Not reported	Blood donors	HCV	Indirect ELISA	Anti-HCV
Diro	2008	Cross sectional	Non probabilistic	Consecutive sampling	Monocenter	Prospetively	Ethiopia	East and Southern	Africa	Eastern Africa	Low-income economies	Apr/2004-Jul/2004	Adults	Hospital-based	Unclear/Not reported	Unclear/Not reported	Blood donors	HBV and HCV	Direct ELISA, Indirect ELISA	Anti-HCV, HBsAg
Diro	2008	Cross sectional	Non probabilistic	Consecutive sampling	Monocenter	Prospetively	Ethiopia	East and Southern	Africa	Eastern Africa	Low-income economies	Apr/2004-Jul/2004	Adults	Hospital-based	Unclear/Not reported	Unclear/Not reported	Blood donors	HBV	Direct ELISA	HBsAg
Diwe	2013	Cross sectional	Non probabilistic	Consecutive sampling	Monocenter	Prospetively	Nigeria	West and Central	Africa	West Africa	Lower-middle income economies	May/2012-Jul/2012	Unclear/Not reported	Hospital-based	Unclear/Not reported	On ART	General population	HCV	Rapid Diagnostic test	Anti-HCV
Diwe	2013	Cross sectional	Non probabilistic	Consecutive sampling	Monocenter	Prospetively	Nigeria	West and Central	Africa	West Africa	Lower-middle income economies	May/2012-Jul/2012	Unclear/Not reported	Hospital-based	Unclear/Not reported	On ART	General population	HBV and HCV	Rapid Diagnostic test	Anti-HCV, HBsAg
Diwe	2013	Cross sectional	Non probabilistic	Consecutive sampling	Monocenter	Prospetively	Nigeria	West and Central	Africa	West Africa	Lower-middle income economies	May/2012-Jul/2012	Unclear/Not reported	Hospital-based	Unclear/Not reported	On ART	General population	HBV	Rapid Diagnostic test	HBsAg
Dovonou	2015	Cross sectional	Non probabilistic	Consecutive sampling	Monocenter	Prospetively	Benin	West and Central	Africa	West Africa	Lower-middle income economies	May/2011-Jun/2012	Adults	Hospital-based	Unclear/Not reported	ART naïve/ On ART	General population	HBV	Direct ELISA	HBsAg
du Plessis	1999	Cross sectional	Probabilistic	Simple random sampling	Multicenter	Prospetively	South Africa	East and Southern	Africa	Southern Africa	Upper-middle-income economies	Unclear/Not reported	All ages	Hospital-based	Unclear/Not reported	Unclear/Not reported	Deceased people	HCV	Indirect ELISA	Anti-HCV
du Plessis	1999	Cross sectional	Probabilistic	Simple random sampling	Multicenter	Prospetively	South Africa	East and Southern	Africa	Southern Africa	Upper-middle-income economies	Unclear/Not reported	All ages	Hospital-based	Unclear/Not reported	Unclear/Not reported	Deceased people	HBV	Radioimmunoassay	HBsAg
Dziuban	2013	Cross sectional	Non probabilistic	Consecutive sampling	Monocenter	Prospetively	Swaziland	Unclear	Africa	Southern Africa	Unclear	Jan/2009-May/2011	All ages	Hospital-based	Unclear/Not reported	ART naïve	General population	HBV	Rapid Diagnostic test	HBsAg
Ejele	2004	Cross sectional	Non probabilistic	Consecutive sampling	Monocenter	Prospetively	Nigeria	West and Central	Africa	West Africa	Lower-middle income economies	Jun/1995-Feb/2003	Unclear/Not reported	Hospital-based	Unclear/Not reported	On ART	General population	HBV	Chromatographic immunoassay technique	HBsAg
Ekouevi	2018	Cross sectional	Non probabilistic	Consecutive sampling	Monocenter	Prospetively	Burkina Faso	West and Central	Africa	West Africa	Low-income economies	Mar/2012-Dec/2012	Adults	Hospital-based	Unclear/Not reported	On ART	General population	HCV	Enzyme immunoassay (EIA)	Anti-HCV
Ekouevi	2018	Cross sectional	Non probabilistic	Consecutive sampling	Monocenter	Prospetively	Ivory Coast	West and Central	Africa	West Africa	Lower-middle income economies	Mar/2012-Dec/2012	Adults	Hospital-based	Unclear/Not reported	On ART	General population	HCV	Enzyme immunoassay (EIA)	Anti-HCV
Elsharkawy	2021	Cross sectional	Non probabilistic	Consecutive sampling	Monocenter	Prospetively	Egypt	North Africa and Middle East	Eastern Mediterranean	Northern Africa	Lower-middle income economies	Jul/2018-Mar/2019	All ages	Hospital-based	Unclear/Not reported	Unclear/Not reported	General population	HCV	Indirect ELISA	Anti-HCV
Eze	2014	Cross sectional	Non probabilistic	Consecutive sampling	Monocenter	Prospetively	Nigeria	West and Central	Africa	West Africa	Lower-middle income economies	Jul/2009-Dec/2009	Children	Hospital-based	Unclear/Not reported	Unclear/Not reported	General population	HCV	Rapid Diagnostic test	Anti-HCV
Ezechi	2014	Cross sectional	Non probabilistic	Consecutive sampling	Monocenter	Prospetively	Nigeria	West and Central	Africa	West Africa	Lower-middle income economies	Jan/2006-Dec/2011	Adults	Hospital-based	Unclear/Not reported	Unclear/Not reported	Pregnant women	HCV	Unclear/Not reported	Anti-HCV
Ezechi	2014	Cross sectional	Non probabilistic	Consecutive sampling	Monocenter	Prospetively	Nigeria	West and Central	Africa	West Africa	Lower-middle income economies	Jan/2006-Dec/2011	Adults	Hospital-based	Unclear/Not reported	Unclear/Not reported	Pregnant women	HBV and HCV	Unclear/Not reported	Anti-HCV, HBsAg
Ezechi	2014	Cross sectional	Non probabilistic	Consecutive sampling	Monocenter	Prospetively	Nigeria	West and Central	Africa	West Africa	Lower-middle income economies	Jan/2006-Dec/2011	Adults	Hospital-based	Unclear/Not reported	Unclear/Not reported	Pregnant women	HBV	Unclear/Not reported	HBsAg
Feldt	2013	Cross sectional	Non probabilistic	Consecutive sampling	Multicenter	Retrospectively	Cameroon	West and Central	Africa	Central Africa	Lower-middle income economies	Sep/2009-Oct/2010	Children	Hospital-based	Unclear/Not reported	On ART	General population	HBV	Enzyme immunoassay (EIA)	HBsAg
Feldt	2013	Cross sectional	Non probabilistic	Consecutive sampling	Multicenter	Retrospectively	Cameroon	West and Central	Africa	Central Africa	Lower-middle income economies	Sep/2009-Oct/2010	Children	Hospital-based	Unclear/Not reported	On ART	General population	HCV	Enzyme immunoassay (EIA)	Anti-HCV
Feldt	2013	Cross sectional	Non probabilistic	Consecutive sampling	Multicenter	Retrospectively	Cameroon	West and Central	Africa	Central Africa	Lower-middle income economies	Sep/2009-Oct/2010	Adults	Hospital-based	Unclear/Not reported	On ART	General population	HCV	Enzyme immunoassay (EIA)	Anti-HCV
Feldt	2013	Cross sectional	Non probabilistic	Consecutive sampling	Multicenter	Retrospectively	Cameroon	West and Central	Africa	Central Africa	Lower-middle income economies	Sep/2009-Oct/2010	Adults	Hospital-based	Unclear/Not reported	On ART	General population	HBV	Enzyme immunoassay (EIA)	HBsAg
Firnhaber	2008	Cross sectional	Non probabilistic	Consecutive sampling	Monocenter	Prospetively	South Africa	East and Southern	Africa	Southern Africa	Upper-middle-income economies	Unclear/Not reported	Unclear/Not reported	Hospital-based	Urban	ART naïve	General population	HBV	Microparticle Enzyme Immunoassay (MEIA)	HBsAg
Forbi	2007	Case control	Probabilistic	Simple random sampling	Monocenter	Retrospectively	Nigeria	West and Central	Africa	West Africa	Lower-middle income economies	Jun/2005-Dec/2005	Adults	Hospital-based	Unclear/Not reported	ART naïve/ On ART	General population	HCV	Indirect ELISA	Anti-HCV

Forbi	2007	Case control	Probabilistic	Simple random sampling	Monocenter	Retrospectively	Nigeria	West and Central	Africa	West Africa	Lower-middle income economies	Jun/2005-Dec/2005	Adults	Hospital-based	Unclear/Not reported	ART naïve/ On ART	General population	HBV and HCV	Direct ELISA, Indirect ELISA	Anti-HCV, HBsAg
Forbi	2007	Case control	Probabilistic	Simple random sampling	Monocenter	Retrospectively	Nigeria	West and Central	Africa	West Africa	Lower-middle income economies	Jun/2005-Dec/2005	Adults	Hospital-based	Unclear/Not reported	ART naïve/ On ART	General population	HBV and HCV	Direct ELISA, Indirect ELISA	Anti-HCV, HBsAg
Forbi	2007	Case control	Probabilistic	Simple random sampling	Monocenter	Retrospectively	Nigeria	West and Central	Africa	West Africa	Lower-middle income economies	Jun/2005-Dec/2005	Adults	Hospital-based	Unclear/Not reported	ART naïve/ On ART	General population	HBV	Direct ELISA	HBsAg
Franzeck	2013	Cross sectional	Non probabilistic	Consecutive sampling	Monocenter	Retrospectively	Tanzania	East and Southern	Africa	Eastern Africa	Lower-middle income economies	Nov/2011-Jun/2012	Adults	Hospital-based	Unclear/Not reported	ART naïve	General population	HBV	Enzyme immunoassay (EIA)	HBsAg
Franzeck	2013	Cross sectional	Non probabilistic	Consecutive sampling	Monocenter	Retrospectively	Tanzania	East and Southern	Africa	Eastern Africa	Lower-middle income economies	Nov/2011-Jun/2012	Adults	Hospital-based	Unclear/Not reported	ART naïve	General population	HCV	Enzyme immunoassay (EIA)	Anti-HCV
Franzeck	2013	Cross sectional	Non probabilistic	Consecutive sampling	Monocenter	Retrospectively	Tanzania	East and Southern	Africa	Eastern Africa	Lower-middle income economies	Nov/2011-Jun/2012	Adults	Hospital-based	Unclear/Not reported	ART naïve	General population	HBV and HCV	Enzyme immunoassay (EIA)	Anti-HCV, HBsAg
Frempong	2019	Cross sectional	Non probabilistic	Consecutive sampling	Multicenter	Prospetively	Ghana	West and Central	Africa	West Africa	Lower-middle income economies	May/2012-May/2013	Adults	Hospital-based	Unclear/Not reported	Unclear/Not reported	Pregnant women	HCV	Indirect ELISA	Anti-HCV
Frempong	2019	Cross sectional	Non probabilistic	Consecutive sampling	Multicenter	Prospetively	Ghana	West and Central	Africa	West Africa	Lower-middle income economies	May/2012-May/2013	Adults	Hospital-based	Unclear/Not reported	Unclear/Not reported	Pregnant women	HBV	Direct ELISA	HBeAg
Frempong	2019	Cross sectional	Non probabilistic	Consecutive sampling	Multicenter	Prospetively	Ghana	West and Central	Africa	West Africa	Lower-middle income economies	May/2012-May/2013	Adults	Hospital-based	Unclear/Not reported	Unclear/Not reported	Pregnant women	HBV	Direct ELISA	HBsAg
Gededzha	2010	Cross sectional	Non probabilistic	Consecutive sampling	Monocenter	Prospetively	South Africa	East and Southern	Africa	Southern Africa	Upper-middle-income economies	2004-2006.	Adults	Hospital-based	Unclear/Not reported	On ART	General population	HCV	Classical RT-PCR	Anti-HCV
Gededzha	2018	Cross sectional	Non probabilistic	Convenience sampling	Monocenter	Prospetively	South Africa	East and Southern	Africa	Southern Africa	Upper-middle-income economies	2005-2007	Adults	Hospital-based	Unclear/Not reported	ART naïve	General population	HBV	Immunoassay kit	HBeAg
Gededzha	2018	Cross sectional	Non probabilistic	Convenience sampling	Monocenter	Prospetively	South Africa	East and Southern	Africa	Southern Africa	Upper-middle-income economies	2005-2007	Adults	Hospital-based	Unclear/Not reported	ART naïve	General population	HBV	Immunoassay kit	HBeAg
Gededzha	2018	Cross sectional	Non probabilistic	Convenience sampling	Monocenter	Prospetively	South Africa	East and Southern	Africa	Southern Africa	Upper-middle-income economies	2005-2007	Adults	Hospital-based	Unclear/Not reported	ART naïve	General population	HBV	Immunoassay kit	Viral DNA
Gedefie	2021	Cross sectional	Non probabilistic	Consecutive sampling	Monocenter	Prospetively	Ethiopia	East and Southern	Africa	Eastern Africa	Low-income economies	Jan/2008-Mar/2008	Adults	Hospital-based	Urban/Rural	On ART	General population	HCV	Immunochromatographic test	Anti-HCV
George	2018	Cross sectional	Non probabilistic	Consecutive sampling	Monocenter	Retrospectively	Nigeria	West and Central	Africa	West Africa	Lower-middle income economies	Jan/2009-Dec/2013	Unclear/Not reported	Hospital-based	Unclear/Not reported	On ART	General population	HCV	Unclear/Not reported	Anti-HCV
George	2018	Cross sectional	Non probabilistic	Consecutive sampling	Monocenter	Retrospectively	Nigeria	West and Central	Africa	West Africa	Lower-middle income economies	Jan/2009-Dec/2013	Unclear/Not reported	Hospital-based	Unclear/Not reported	On ART	General population	HBV	Unclear/Not reported	HBsAg
Geretti	2010	Cross sectional	Non probabilistic	Consecutive sampling	Monocenter	Prospetively	Ghana	West and Central	Africa	West Africa	Lower-middle income economies	Unclear/Not reported	Unclear/Not reported	Hospital-based	Unclear/Not reported	Unclear/Not reported	General population	HBV	Immunoassay kit	HBsAg
Geretti	2010	Cross sectional	Non probabilistic	Consecutive sampling	Monocenter	Prospetively	Ghana	West and Central	Africa	West Africa	Lower-middle income economies	Unclear/Not reported	Unclear/Not reported	Hospital-based	Unclear/Not reported	Unclear/Not reported	General population	HBV	Real-time PCR	Viral DNA
Giuliano	2018	Cohort (Baseline data)	Non probabilistic	Consecutive sampling	Monocenter	Prospetively	Malawi	East and Southern	Africa	Eastern Africa	Low-income economies	2008-2011	Unclear/Not reported	Community-based	Unclear/Not reported	Unclear/Not reported	Pregnant women	HBV	Immunoassay kit	HBsAg
Goa	2019	Cross sectional	Probabilistic	Simple random sampling	Monocenter	Prospetively	Ethiopia	East and Southern	Africa	Eastern Africa	Low-income economies	Oct/2017-Dec/2017	Adults	Hospital-based	Urban/Rural	On ART	General population	HBV	Immunoassay kit	HBsAg
Gogela	2018	Cross sectional	Non probabilistic	Consecutive sampling	Monocenter	Prospetively	South Africa	East and Southern	Africa	Southern Africa	Upper-middle-income economies	2011-2014	Adults	Hospital-based	Unclear/Not reported	Unclear/Not reported	General population	HCV	Immunoassay kit	Anti-HCV
Goverwa-Sibanda	2020	Cross sectional	Non probabilistic	Consecutive sampling	Monocenter	Prospetively	Zimbabwe	East and Southern	Africa	Eastern Africa	Lower-middle income economies	Oct/2017-Apr/2019	All ages	Hospital-based	Unclear/Not reported	On ART	General population	HBV	Rapid Diagnostic test	HBsAg
Greer	2017	Clinical Trial (Baseline data)	Probabilistic	Simple random sampling	Multicenter	Prospetively	Botswana	East and Southern	Africa	Southern Africa	Upper-middle-income economies	Unclear/Not reported	Unclear/Not reported	Hospital-based	Unclear/Not reported	On ART	General population	HBV	ADVIA Centaur chemiluminometric immunoassay system	HBsAg
Greer	2017	Clinical Trial (Baseline data)	Probabilistic	Simple random sampling	Multicenter	Prospetively	Kenya	East and Southern	Africa	Eastern Africa	Lower-middle income economies	Unclear/Not reported	Unclear/Not reported	Hospital-based	Unclear/Not reported	On ART	General population	HBV	ADVIA Centaur chemiluminometric immunoassay system	HBsAg
Greer	2017	Clinical Trial (Baseline data)	Probabilistic	Simple random sampling	Multicenter	Prospetively	Malawi	East and Southern	Africa	Eastern Africa	Low-income economies	Unclear/Not reported	Unclear/Not reported	Hospital-based	Unclear/Not reported	On ART	General population	HBV	ADVIA Centaur chemiluminometric immunoassay system	HBsAg
Greer	2017	Clinical Trial (Baseline data)	Probabilistic	Simple random sampling	Multicenter	Prospetively	South Africa	East and Southern	Africa	Southern Africa	Upper-middle-income economies	Unclear/Not reported	Unclear/Not reported	Hospital-based	Unclear/Not reported	On ART	General population	HBV	ADVIA Centaur chemiluminometric immunoassay system	HBsAg
Gudo	2018	Cross sectional	Non probabilistic	Consecutive sampling	Multicenter	Prospetively	Gabon	West and Central	Africa	Central Africa	Upper-middle-income economies	Oct/2015-Apr/2016	Adults	Hospital-based	Unclear/Not reported	ART naïve/ On ART	General population	HBV	Real-time PCR	Viral DNA
Guimarães	2013	Cross sectional	Non probabilistic	Consecutive sampling	Monocenter	Prospetively	Angola	East and Southern	Africa	Central Africa	Lower-middle income economies	Unclear/Not reported	Unclear/Not reported	Hospital-based	Unclear/Not reported	Unclear/Not reported	General population	HCV	Indirect ELISA	Anti-HCV
Guimarães	2013	Cross sectional	Non probabilistic	Consecutive sampling	Monocenter	Prospetively	Angola	East and Southern	Africa	Central Africa	Lower-middle income economies	Unclear/Not reported	Unclear/Not reported	Hospital-based	Unclear/Not reported	Unclear/Not reported	General population	HBV	Direct ELISA	HBsAg
Hadush	2013	Cross sectional	Non probabilistic	Consecutive sampling	Multicenter	Prospetively	Ethiopia	East and Southern	Africa	Eastern Africa	Low-income economies	Dec/2010-Feb/2011	Unclear/Not reported	Hospital-based	Unclear/Not reported	On ART	General population	HCV	Indirect ELISA	Anti-HCV
Harania	2008	Cross sectional	Non probabilistic	Consecutive sampling	Monocenter	Prospetively	Kenya	East and Southern	Africa	Eastern Africa	Lower-middle income economies	Unclear/Not reported	Adults	Hospital-based	Unclear/Not reported	Unclear/Not reported	General population	HCV	Indirect ELISA	Anti-HCV
Harania	2008	Cross sectional	Non probabilistic	Consecutive sampling	Monocenter	Prospetively	Kenya	East and Southern	Africa	Eastern Africa	Lower-middle income economies	Unclear/Not reported	Adults	Hospital-based	Unclear/Not reported	Unclear/Not reported	General population	HBV and HCV	Direct ELISA, Indirect ELISA	Anti-HCV, HBsAg
Harania	2008	Cross sectional	Non probabilistic	Consecutive sampling	Monocenter	Prospetively	Kenya	East and Southern	Africa	Eastern Africa	Lower-middle income economies	Unclear/Not reported	Adults	Hospital-based	Unclear/Not reported	Unclear/Not reported	General population	HBV	Direct ELISA	HBsAg
Harvard	2019	Cross sectional	Non probabilistic	Consecutive sampling	Monocenter	Prospetively	Nigeria	West and Central	Africa	West Africa	Lower-middle income economies	Unclear/Not reported	Adults	Hospital-based	Unclear/Not reported	On ART	General population	HBV	Rapid Diagnostic test	HBeAg
Harvard	2019	Cross sectional	Non probabilistic	Consecutive sampling	Monocenter	Prospetively	Nigeria	West and Central	Africa	West Africa	Lower-middle income economies	Unclear/Not reported	Adults	Hospital-based	Unclear/Not reported	On ART	General population	HBV	Rapid Diagnostic test	HBsAg
Hawkins	2013	Cross sectional	Non probabilistic	Consecutive sampling	Monocenter	Prospetively	Tanzania	East and Southern	Africa	Eastern Africa	Lower-middle income economies	Nov/2004-Sep/2011	Adults	Hospital-based	Unclear/Not reported	On ART	General population	HBV	Rapid Diagnostic test	HBsAg
Hector	2018	Cohort (Baseline data)	Non probabilistic	Consecutive sampling	Multicenter	Prospetively	Mozambique	East and Southern	Africa	Eastern Africa	Low-income economies	Jan/2013	Unclear/Not reported	Community-based	Unclear/Not reported	On ART	General population	HBV	Rapid Diagnostic test	HBsAg
Hoffmann	2012	Case control	Non probabilistic	Consecutive sampling	Multicenter	Prospetively	South Africa	East and Southern	Africa	Southern Africa	Upper-middle-income economies	Unclear/Not reported	Adults	Hospital-based	Unclear/Not reported	ART naïve/ On ART	Pregnant women	HBV	Direct ELISA	HBsAg
Hoffmann	2012	Case control	Non probabilistic	Consecutive sampling	Multicenter	Prospetively	South Africa	East and Southern	Africa	Southern Africa	Upper-middle-income economies	Unclear/Not reported	Adults	Hospital-based	Unclear/Not reported	ART naïve/ On ART	Pregnant women	HCV	Direct ELISA	Anti-HCV
Hoffmann	2014	Cross sectional	Non probabilistic	Consecutive sampling	Monocenter	Prospetively	South Africa	East and Southern	Africa	Southern Africa	Upper-middle-income economies	Unclear/Not reported	Adults	Hospital-based	Unclear/Not reported	On ART	Pregnant women	HBV	Immunoassay kit	HBsAg
Hoffmann	2007	Cohort (Baseline data)	Probabilistic	Simple random sampling	Monocenter	Prospetively	South Africa	East and Southern	Africa	Southern Africa	Upper-middle-income economies	Nov/2002-Dec/2005	Unclear/Not reported	Hospital-based	Unclear/Not reported	ART naïve	General population	HCV	Immunoassay kit	Anti-HCV
Hoffmann	2007	Cohort (Baseline data)	Probabilistic	Simple random sampling	Monocenter	Prospetively	South Africa	East and Southern	Africa	Southern Africa	Upper-middle-income economies	Nov/2002-Dec/2005	Unclear/Not reported	Hospital-based	Unclear/Not reported	ART naïve	General population	HBV	Immunoassay kit	HBsAg
Hoffmann	2008	Cohort (Baseline data)	Non probabilistic	Consecutive sampling	Monocenter	Prospetively	South Africa	East and Southern	Africa	Southern Africa	Upper-middle-income economies	Nov/2002-Nov/2006	Adults	Hospital-based	Unclear/Not reported	ART naïve	General population	HCV	Unclear/Not reported	Anti-HCV
Hoffmann	2008	Cohort (Baseline data)	Non probabilistic	Consecutive sampling	Monocenter	Prospetively	South Africa	East and Southern	Africa	Southern Africa	Upper-middle-income economies	Nov/2002-Nov/2006	Adults	Hospital-based	Unclear/Not reported	ART naïve	General population	HBV	Immunoassay kit	HBsAg
Hønge	2014	Cross sectional	Non probabilistic	Consecutive sampling	Monocenter	Prospetively	Guinea-Bissau	West and Central	Africa	West Africa	Low-income economies	Apr/2011-Sep/2011	Adults	Hospital-based	Unclear/Not reported	Unclear/Not reported	General population	HCV	Rapid Diagnostic test	Anti-HCV
Hønge	2014	Cross sectional	Non probabilistic	Consecutive sampling	Monocenter	Prospetively	Guinea-Bissau	West and Central	Africa	West Africa	Low-income economies	Apr/2011-Sep/2011	Adults	Hospital-based	Unclear/Not reported	Unclear/Not reported	General population	HBV	Rapid Diagnostic test	HBsAg
Hønge	2014	Cross sectional	Non probabilistic	Consecutive sampling	Monocenter	Prospetively	Guinea-Bissau	West and Central	Africa	West Africa	Low-income economies	Apr/2011-Sep/2011	Adults	Hospital-based	Unclear/Not reported	ART naïve/ On ART	General population	HCV	Real-time PCR	Viral RNA
Hønge	2014	Cross sectional	Non probabilistic	Consecutive sampling	Monocenter	Prospetively	Guinea-Bissau	West and Central	Africa	West Africa	Low-income economies	Apr/2011- Sep/2011	Adults	Hospital-based	Unclear/Not reported	ART naïve/ On ART	General population	HBV	Real-time PCR	Viral DNA
Houghtaling	2018	Clinical Trial (Baseline data)	Probabilistic	Simple random sampling	Monocenter	Prospetively	Ivory Coast	West and Central	Africa	West Africa	Lower-middle income economies	Dec/2002-Apr/2004	Adults	Hospital-based	Unclear/Not reported	On ART	General population	HBV	Classical PCR	Viral DNA
Ifeorah	2017	Cohort (Baseline data)	Non probabilistic	Consecutive sampling	Multicenter	Prospetively	Nigeria	West and Central	Africa	West Africa	Lower-middle income economies	Apr/2016-Oct/2016	All ages	Hospital-based	Unclear/Not reported	Unclear/Not reported	General population	HBV	Direct ELISA	HBsAg
Ikomey	2016	Cross sectional	Non probabilistic	Consecutive sampling	Multicenter	Prospetively	Cameroon	West and Central	Africa	Central Africa	Lower-middle income economies	Mar/2014-Aug/2014	Unclear/Not reported	Hospital-based	Unclear/Not reported	Unclear/Not reported	Pregnant women	HCV	Indirect ELISA	Anti-HCV
Ikomey	2016	Cross sectional	Non probabilistic	Consecutive sampling	Multicenter	Prospetively	Cameroon	West and Central	Africa	Central Africa	Lower-middle income economies	Mar/2014-Aug/2014	Unclear/Not reported	Hospital-based	Unclear/Not reported	Unclear/Not reported	Pregnant women	HBV and HCV	Direct ELISA, Indirect ELISA	Anti-HCV, HBsAg
Ikomey	2016	Cross sectional	Non probabilistic	Consecutive sampling	Multicenter	Prospetively	Cameroon	West and Central	Africa	Central Africa	Lower-middle income economies	Mar/2014-Aug/2014	Unclear/Not reported	Hospital-based	Unclear/Not reported	Unclear/Not reported	Pregnant women	HBV	Direct ELISA	HBsAg
Ikpeme	2013	Cross sectional	Non probabilistic	Consecutive sampling	Monocenter	Prospetively	Nigeria	West and Central	Africa	West Africa	Lower-middle income economies	Dec/2010-Aug/2011	Unclear/Not reported	Hospital-based	Unclear/Not reported	On ART	General population	HBV	Rapid Diagnostic test	HBsAg
Ilboudo	2007	Cross sectional	Non probabilistic	Consecutive sampling	Monocenter	Prospetively	Burkina Faso	West and Central	Africa	West Africa	Low-income economies	Feb/2005-Mar/2006	Adults	Hospital-based	Unclear/Not reported	Unclear/Not reported	Pregnant women	HBV	Immunoassay kit	HBsAg
Inyama	2005	Cross sectional	Non probabilistic	Consecutive sampling	Monocenter	Prospetively	Nigeria	West and Central	Africa	West Africa	Lower-middle income economies	Jun/2002-May/2003	Adults	Hospital-based	Unclear/Not reported	Unclear/Not reported	General population	HCV	Enzyme immunoassay (EIA)	Anti-HCV
Ive	2013	Clinical Trial (Baseline data)	Probabilistic	Simple random sampling	Multicenter	Prospetively	South Africa	East and Southern	Africa	Southern Africa	Upper-middle-income economies	Feb/2005-Feb/2007	Adults	Hospital-based	Unclear/Not reported	ART naïve/ On ART	General population	HBV	Enzyme immunoassay (EIA)	HBsAg
Iwalokun	2006	Case control	Non probabilistic	Consecutive sampling	Monocenter	Retrospectively	Nigeria	West and Central	Africa	West Africa	Lower-middle income economies	Unclear/Not reported	Adults	Hospital-based	Unclear/Not reported	Unclear/Not reported	General population	HBV	Agglutination test kits	HBsAg
Jackson	1991	Cross sectional	Non probabilistic	Consecutive sampling	Monocenter	Prospetively	Uganda	East and Southern	Africa	Eastern Africa	Low-income economies	Jul/1990	Adults	Hospital-based	Unclear/Not reported	Unclear/Not reported	Pregnant women	HCV	Enzyme immunoassay (EIA)	Anti-HCV
Jaquet	2017	Cross sectional	Non probabilistic	Consecutive sampling	Multicenter	Prospetively	Ivory Coast	West and Central	Africa	West Africa	Lower-middle income economies	Unclear/Not reported	Adults	Hospital-based	Unclear/Not reported	ART naïve/ On ART	General population	HCV	Rapid Diagnostic test	Anti-HCV
Jaquet	2017	Cross sectional	Non probabilistic	Consecutive sampling	Multicenter	Prospetively	Ivory Coast	West and Central	Africa	West Africa	Lower-middle income economies	Unclear/Not reported	Adults	Hospital-based	Unclear/Not reported	ART naïve/ On ART	General population	HBV	Rapid Diagnostic test	HBsAg
Jaquet	2017	Cross sectional	Non probabilistic	Consecutive sampling	Multicenter	Prospetively	Senegal	West and Central	Africa	West Africa	Lower-middle income economies	Unclear/Not reported	Adults	Hospital-based	Unclear/Not reported	ART naïve/ On ART	General population	HCV	Rapid Diagnostic test	Anti-HCV
Jaquet	2017	Cross sectional	Non probabilistic	Consecutive sampling	Multicenter	Prospetively	Senegal	West and Central	Africa	West Africa	Lower-middle income economies	Unclear/Not reported	Adults	Hospital-based	Unclear/Not reported	ART naïve/ On ART	General population	HBV	Rapid Diagnostic test	HBsAg
Jaquet	2017	Cross sectional	Non probabilistic	Consecutive sampling	Multicenter	Prospetively	Togo	West and Central	Africa	West Africa	Low-income economies	Unclear/Not reported	Adults	Hospital-based	Unclear/Not reported	ART naïve/ On ART	General population	HCV	Rapid Diagnostic test	Anti-HCV
Jaquet	2017	Cross sectional	Non probabilistic	Consecutive sampling	Multicenter	Prospetively	Togo	West and Central	Africa	West Africa	Low-income economies	Unclear/Not reported	Adults	Hospital-based	Unclear/Not reported	ART naïve/ On ART	General population	HBV	Rapid Diagnostic test	HBsAg
Jobarteh	2010	Case control	Non probabilistic	Consecutive sampling	Monocenter	Retrospectively	Gambia	West and Central	Africa	West Africa	Low-income economies	1988-2008	All ages	Hospital-based	Unclear/Not reported	On ART	General population	HBV	Real-time PCR	Viral DNA
Jobarteh	2010	Case control	Non probabilistic	Consecutive sampling	Monocenter	Retrospectively	Gambia	West and Central	Africa	West Africa	Low-income economies	1988-2008	All ages	Hospital-based	Unclear/Not reported	ART naïve/ On ART	General population	HCV	Real-time RT-PCR	Viral RNA
Jooste	2016	Cohort (Baseline data)	Non probabilistic	Consecutive sampling	Monocenter	Prospetively	South Africa	East and Southern	Africa	Southern Africa	Upper-middle-income economies	Mar/2015-May/2016	Children	Hospital-based	Unclear/Not reported	Unclear/Not reported	General population	HBV	Immunoassay kit	HBsAg
Joseph	2019	Cross sectional	Non probabilistic	Consecutive sampling	Monocenter	Prospetively	Cameroon	West and Central	Africa	Central Africa	Lower-middle income economies	Feb/2017-Jun/2017	Children	Hospital-based	Unclear/Not reported	On ART	General population	HBV	Enzyme immunoassay (EIA)	HBsAg
Kaba	2019	Cross sectional	Non probabilistic	Consecutive sampling	Monocenter	Prospetively	Guinea	West and Central	Africa	West Africa	Low-income economies	Feb/2017-Jul/2017	Children	Hospital-based	Unclear/Not reported	On ART	General population	HBV	Rapid Diagnostic test	HBsAg
Kallestrup	2003	Cross sectional	Non probabilistic	Consecutive sampling	Monocenter	Retrospectively	Zimbabwe	East and Southern	Africa	Eastern Africa	Lower-middle income economies	Unclear/Not reported	Adults	Community-based	Unclear/Not reported	Unclear/Not reported	General population	HCV	Enzyme immunoassay (EIA)	Anti-HCV
Kamenya	2017	Cross sectional	Probabilistic	Systematic sampling	Monocenter	Prospetively	Tanzania	East and Southern	Africa	Eastern Africa	Lower-middle income economies	Mar/2015-Jun/2015	Adults	Hospital-based	Unclear/Not reported	Unclear/Not reported	General population	HBV	Rapid Diagnostic test	HBsAg
Kania	2009	Cross sectional	Probabilistic	Simple random sampling	Monocenter	Prospetively	Burkina Faso	West and Central	Africa	West Africa	Low-income economies	May/2002- Aug/2002	Adults	Hospital-based	Unclear/Not reported	Unclear/Not reported	Blood donors	HCV	Indirect ELISA	Anti-HCV
Kania	2009	Cross sectional	Probabilistic	Simple random sampling	Monocenter	Prospetively	Burkina Faso	West and Central	Africa	West Africa	Low-income economies	May/2002- Aug/2002	Adults	Hospital-based	Unclear/Not reported	Unclear/Not reported	Blood donors	HBV	Direct ELISA	HBsAg
Kapembwa	2011	Cross sectional	Non probabilistic	Consecutive sampling	Monocenter	Prospetively	Zambia	East and Southern	Africa	Eastern Africa	Lower-middle income economies	Dec/2007-Jun/2008,	Adults	Hospital-based	Unclear/Not reported	ART naïve	General population	HCV	Indirect ELISA	Anti-HCV
Kapembwa	2011	Cross sectional	Non probabilistic	Consecutive sampling	Monocenter	Prospetively	Zambia	East and Southern	Africa	Eastern Africa	Lower-middle income economies	Dec/2007-Jun/2008,	Adults	Hospital-based	Unclear/Not reported	ART naïve	General population	HBV and HCV	Direct ELISA, Indirect ELISA	Anti-HCV, HBsAg
Kapembwa	2011	Cross sectional	Non probabilistic	Consecutive sampling	Monocenter	Prospetively	Zambia	East and Southern	Africa	Eastern Africa	Lower-middle income economies	Dec/2007-Jun/2008,	Adults	Hospital-based	Unclear/Not reported	ART naïve	General population	HBV	Direct ELISA	HBsAg
Kashala	1994	Cross sectional	Probabilistic	Stratified sampling	Multicenter	Prospetively	Democratic Republic o	West and Central	Africa	Central Africa	Low-income economies	1989-1992	Unclear/Not reported	Hospital-based	Unclear/Not reported	Unclear/Not reported	General population	HBV	Direct ELISA	HBsAg
Kashala	1994	Cross sectional	Probabilistic	Stratified sampling	Multicenter	Prospetively	Democratic Republic o	West and Central	Africa	Central Africa	Low-income economies	1989-1992	Unclear/Not reported	Hospital-based	Unclear/Not reported	Unclear/Not reported	General population	HBV	Indirect ELISA	HBsAg + IgM anti-HBc
Kashala	1994	Cross sectional	Probabilistic	Stratified sampling	Multicenter	Prospetively	Democratic Republic o	West and Central	Africa	Central Africa	Low-income economies	1989-1992	Unclear/Not reported	Hospital-based	Unclear/Not reported	Unclear/Not reported	General population	HBV	Real-time PCR	Viral DNA
Katusiime	2016	Cross sectional	Non probabilistic	Consecutive sampling	Monocenter	Prospetively	Uganda	East and Southern	Africa	Eastern Africa	Low-income economies	Jul/2011-Apr/2012	Adults	Hospital-based	Urban	Unclear/Not reported	General population	HBV	Unclear/Not reported	HBsAg
Kfutwah	2012	Cross sectional	Non probabilistic	Consecutive sampling	Monocenter	Prospetively	Cameroon	West and Central	Africa	Central Africa	Lower-middle income economies	Jan/2000-Apr/2003	Unclear/Not reported	Hospital-based	Unclear/Not reported	Unclear/Not reported	Pregnant women	HBV	Enzyme immunoassay (EIA)	HBsAg
Kibaya	2015	Cross sectional	Non probabilistic	Consecutive sampling	Monocenter	Prospetively	Kenya	East and Southern	Africa	Eastern Africa	Lower-middle income economies	Feb/2010-Mar/2010	Unclear/Not reported	Community-based	Unclear/Not reported	Unclear/Not reported	Injecting drug users	HBV	Enzyme immunoassay (EIA)	HBsAg
Kilani	2007	Cross sectional	Non probabilistic	Consecutive sampling	Multicenter	Retrospectively	Tunisia	North Africa and Middle East	Eastern Mediterranean	Northern Africa	Lower-middle income economies	Unclear/Not reported	Unclear/Not reported	Hospital-based	Unclear/Not reported	Unclear/Not reported	General population	HCV	Indirect ELISA	Anti-HCV
Kilonzo	2017	Cross sectional	Non probabilistic	Consecutive sampling	Monocenter	Retrospectively	Tanzania	East and Southern	Africa	Eastern Africa	Lower-middle income economies	May/2014-Jul/2015	All ages	Hospital-based	Unclear/Not reported	ART naïve	General population	HBV	Unclear/Not reported	HBsAg
King	2015	Cross sectional	Non probabilistic	Consecutive sampling	Monocenter	Prospetively	Ghana	West and Central	Africa	West Africa	Lower-middle income economies	2010-2012	Adults	Hospital-based	Unclear/Not reported	Unclear/Not reported	General population	HCV	Real-time RT-PCR	Viral RNA
King	2016	Cross sectional	Non probabilistic	Consecutive sampling	Monocenter	Retrospectively	South Africa	East and Southern	Africa	Southern Africa	Upper-middle-income economies	2004-2010	Unclear/Not reported	Hospital-based	Unclear/Not reported	ART naïve	General population	HBV	Unclear/Not reported	HBsAg
Kirakoya-Samadoulougou	2014	Cross sectional	Non probabilistic	Consecutive sampling	Multicenter	Prospetively	Burkina Faso	West and Central	Africa	West Africa	Low-income economies	2010	Adults	Community-based	Unclear/Not reported	Unclear/Not reported	Blood donors	HCV	Enzyme immunoassay (EIA)	Anti-HCV
Kirakoya-Samadoulougou	2014	Cross sectional	Non probabilistic	Consecutive sampling	Multicenter	Prospetively	Burkina Faso	West and Central	Africa	West Africa	Low-income economies	2010	Adults	Community-based	Unclear/Not reported	Unclear/Not reported	Blood donors	HBV	Enzyme immunoassay (EIA)	HBsAg
Kouamé	2018	Clinical Trial (Baseline data)	Probabilistic	Simple random sampling	Multicenter	Prospetively	Ivory Coast	West and Central	Africa	West Africa	Lower-middle income economies	Mar/2008-Jul/2012	Adults	Hospital-based	Unclear/Not reported	On ART	General population	HBV	Direct ELISA	HBsAg
Kouamé	2018	Clinical Trial (Baseline data)	Probabilistic	Simple random sampling	Multicenter	Prospetively	Ivory Coast	West and Central	Africa	West Africa	Lower-middle income economies	Mar/2008-Jul/2012	Adults	Hospital-based	Unclear/Not reported	On ART	General population	CFR	Direct ELISA	HBsAg
Kouanfack	2012	Cross sectional	Non probabilistic	Consecutive sampling	Monocenter	Prospetively	Cameroon	West and Central	Africa	Central Africa	Lower-middle income economies	Oct/2006-nov/2007	Adults	Hospital-based	Unclear/Not reported	Unclear/Not reported	General population	HBV	Enzyme immunoassay (EIA)	HBsAg
Kubio	2012	Cross sectional	Non probabilistic	Consecutive sampling	Monocenter	Prospetively	Ghana	West and Central	Africa	West Africa	Lower-middle income economies	2009	Unclear/Not reported	Hospital-based	Unclear/Not reported	Unclear/Not reported	Blood donors	HCV	Unclear/Not reported	Anti-HCV
Kubio	2012	Cross sectional	Non probabilistic	Consecutive sampling	Monocenter	Prospetively	Ghana	West and Central	Africa	West Africa	Lower-middle income economies	2009	Unclear/Not reported	Hospital-based	Unclear/Not reported	Unclear/Not reported	Blood donors	HBV and HCV	Unclear/Not reported	Anti-HCV, HBsAg
Kubio	2012	Cross sectional	Non probabilistic	Consecutive sampling	Monocenter	Prospetively	Ghana	West and Central	Africa	West Africa	Lower-middle income economies	2009	Unclear/Not reported	Hospital-based	Unclear/Not reported	Unclear/Not reported	Blood donors	HBV	Unclear/Not reported	HBsAg
Kwofie	2021	Cohort (Baseline data)	Non probabilistic	Consecutive sampling	Multicenter	Prospetively	Ghana	West and Central	Africa	West Africa	Lower-middle income economies	Jan/2014-Dec/2015	All ages	Hospital-based	Unclear/Not reported	On ART	General population	HBV	Enzyme immunoassay (EIA)	HBsAg
Kwofie	2021	Cohort (Baseline data)	Non probabilistic	Consecutive sampling	Multicenter	Prospetively	Ghana	West and Central	Africa	West Africa	Lower-middle income economies	Jan/2014-Dec/2015	All ages	Hospital-based	Unclear/Not reported	On ART	General population	HCV	Enzyme immunoassay (EIA)	Anti-HCV
Kwofie	2021	Cohort (Baseline data)	Non probabilistic	Consecutive sampling	Multicenter	Prospetively	Ghana	West and Central	Africa	West Africa	Lower-middle income economies	Jan/2014-Dec/2015	All ages	Hospital-based	Unclear/Not reported	On ART	General population	HBV and HCV	Enzyme immunoassay (EIA)	Anti-HCV, HBsAg
Kye-Duodu	2016	Cross sectional	Non probabilistic	Consecutive sampling	Multicenter	Prospetively	Ghana	West and Central	Africa	West Africa	Lower-middle income economies	Mar/2012-Jun/2012	All ages	Hospital-based	Unclear/Not reported	Unclear/Not reported	General population	HBV	Direct ELISA	HBsAg
Ladep	2007	Cohort (Baseline data)	Non probabilistic	Consecutive sampling	Monocenter	Retrospectively	Nigeria	West and Central	Africa	West Africa	Lower-middle income economies	Sep/2002-Feb/2005	Unclear/Not reported	Hospital-based	Unclear/Not reported	On ART	General population	HCV	Indirect ELISA	Anti-HCV
Ladep	2013	Cohort (Baseline data)	Non probabilistic	Consecutive sampling	Monocenter	Prospetively	Nigeria	West and Central	Africa	West Africa	Lower-middle income economies	Jun/2004-Dec/2010	Adults	Hospital-based	Unclear/Not reported	On ART	General population	HBV	Enzyme immunoassay (EIA)	HBsAg
Ladep	2013	Cohort (Baseline data)	Non probabilistic	Consecutive sampling	Monocenter	Prospetively	Nigeria	West and Central	Africa	West Africa	Lower-middle income economies	Jun/2004-Dec/2010	Adults	Hospital-based	Unclear/Not reported	On ART	General population	CFR	Enzyme immunoassay (EIA)	HBsAg
Ladep	2013	Cohort (Baseline data)	Non probabilistic	Consecutive sampling	Monocenter	Retrospectively	Nigeria	West and Central	Africa	West Africa	Lower-middle income economies	Jun/2004-Dec/2010	Unclear/Not reported	Hospital-based	Unclear/Not reported	Unclear/Not reported	General population	HCV	Enzyme immunoassay (EIA)	Anti-HCV
Ladep	2013	Cohort (Baseline data)	Non probabilistic	Consecutive sampling	Monocenter	Retrospectively	Nigeria	West and Central	Africa	West Africa	Lower-middle income economies	Jun/2004-Dec/2010	Unclear/Not reported	Hospital-based	Unclear/Not reported	Unclear/Not reported	General population	HBV and HCV	Enzyme immunoassay (EIA)	Anti-HCV, HBsAg
Ladep	2013	Cohort (Baseline data)	Non probabilistic	Consecutive sampling	Monocenter	Retrospectively	Nigeria	West and Central	Africa	West Africa	Lower-middle income economies	Jun/2004-Dec/2010	Unclear/Not reported	Hospital-based	Unclear/Not reported	Unclear/Not reported	General population	HBV	Enzyme immunoassay (EIA)	HBsAg
Lassey	2004	Cross sectional	Probabilistic	Simple random sampling	Monocenter	Prospetively	Ghana	West and Central	Africa	West Africa	Lower-middle income economies	Mar/2001-Sep/2001	Unclear/Not reported	Hospital-based	Unclear/Not reported	Unclear/Not reported	Pregnant women	HCV	Indirect ELISA	Anti-HCV
Laurent	2010	Cohort (Baseline data)	Non probabilistic	Consecutive sampling	Multicenter	Retrospectively	Cameroon	West and Central	Africa	Central Africa	Lower-middle income economies	Jan/2001-Apr/2003	Unclear/Not reported	Hospital-based	Unclear/Not reported	On ART	General population	HCV	Enzyme immunoassay (EIA)	Anti-HCV
Laurent	2010	Cohort (Baseline data)	Non probabilistic	Consecutive sampling	Multicenter	Retrospectively	Cameroon	West and Central	Africa	Central Africa	Lower-middle income economies	Jan/2001-Apr/2003	Unclear/Not reported	Hospital-based	Unclear/Not reported	On ART	General population	HBV	Enzyme immunoassay (EIA)	HBsAg
Laurent	2010	Cohort (Baseline data)	Non probabilistic	Consecutive sampling	Multicenter	Retrospectively	Cameroon	West and Central	Africa	Central Africa	Lower-middle income economies	Jan/2001-Apr/2003	Unclear/Not reported	Hospital-based	Unclear/Not reported	On ART	General population	HBV and HCV	Real-time PCR, Real-time RT-PCR	Viral DNA, Viral RNA
Laurent	2010	Cohort (Baseline data)	Non probabilistic	Consecutive sampling	Multicenter	Retrospectively	Cameroon	West and Central	Africa	Central Africa	Lower-middle income economies	Jan/2001-Apr/2003	Unclear/Not reported	Hospital-based	Unclear/Not reported	On ART	General population	HCV	Real-time RT-PCR	Viral RNA
Laurent	2007	Cross sectional	Non probabilistic	Consecutive sampling	Multicenter	Prospetively	Cameroon	West and Central	Africa	Central Africa	Lower-middle income economies	Sep/2001	Adults	Community-based	Unclear/Not reported	Unclear/Not reported	General population	HCV	Indirect ELISA	Anti-HCV
Laurent	2001	Cross sectional	Probabilistic	Simple random sampling	Multicenter	Prospetively	Democratic Republic o	West and Central	Africa	Central Africa	Low-income economies	1988; 1990	Unclear/Not reported	Hospital/community based	Unclear/Not reported	Unclear/Not reported	Pregnant women	HCV	Indirect ELISA	Anti-HCV
Laurent	2001	Cross sectional	Probabilistic	Simple random sampling	Multicenter	Prospetively	Democratic Republic o	West and Central	Africa	Central Africa	Low-income economies	1988; 1990	Unclear/Not reported	Hospital/community based	Unclear/Not reported	Unclear/Not reported	Commercial sex workers	HCV	Indirect ELISA	Anti-HCV
Lawal	2020	Cross sectional	Probabilistic	Systematic sampling	Monocenter	Prospetively	Nigeria	West and Central	Africa	West Africa	Lower-middle income economies	Aug/2017-Dec/2017	Children	Hospital-based	Unclear/Not reported	Unclear/Not reported	General population	HCV	Indirect ELISA	Anti-HCV
Lawal	2020	Cross sectional	Probabilistic	Systematic sampling	Monocenter	Prospetively	Nigeria	West and Central	Africa	West Africa	Lower-middle income economies	Aug/2017-Dec/2017	Children	Hospital-based	Unclear/Not reported	Unclear/Not reported	General population	HBV and HCV	Indirect ELISA, Direct ELISA	Anti-HCV, HBsAg
Lawal	2020	Cross sectional	Probabilistic	Systematic sampling	Monocenter	Prospetively	Nigeria	West and Central	Africa	West Africa	Lower-middle income economies	Aug/2017-Dec/2017	Children	Hospital-based	Unclear/Not reported	Unclear/Not reported	General population	HBV	Direct ELISA	HBsAg
Lepretre	2015	Cross sectional	Probabilistic	Snowball sampling	Multicenter	Prospetively	Senegal	West and Central	Africa	West Africa	Lower-middle income economies	Apr/2011-Jul/2011	Unclear/Not reported	Community-based	Unclear/Not reported	Unclear/Not reported	Injecting drug users	HBV	Rapid Diagnostic test	HBsAg
Lepretre	2015	Cross sectional	Probabilistic	Snowball sampling	Multicenter	Prospetively	Senegal	West and Central	Africa	West Africa	Lower-middle income economies	Apr/2011-Jul/2011	Unclear/Not reported	Community-based	Unclear/Not reported	Unclear/Not reported	Injecting drug users	HCV	Rapid Diagnostic test	Anti-HCV
Lesi	2007	Case control	Non probabilistic	Consecutive sampling	Monocenter	Prospetively	Nigeria	West and Central	Africa	West Africa	Lower-middle income economies	Unclear/Not reported	Unclear/Not reported	Hospital-based	Unclear/Not reported	Unclear/Not reported	General population	HCV	Rapid Diagnostic test	Anti-HCV
Lesi	2007	Case control	Non probabilistic	Consecutive sampling	Monocenter	Prospetively	Nigeria	West and Central	Africa	West Africa	Lower-middle income economies	Unclear/Not reported	Unclear/Not reported	Hospital-based	Unclear/Not reported	Unclear/Not reported	General population	HBV	Rapid Diagnostic test	HBsAg
Liégeois	2020	Cross sectional	Non probabilistic	Consecutive sampling	Multicenter	Prospetively	Cameroon	West and Central	Africa	Central Africa	Lower-middle income economies	Mar/2014-Dec/2014	Adults	Hospital-based	Unclear/Not reported	Unclear/Not reported	General population	HBV	Direct ELISA	HBsAg
Lô	2016	Cross sectional	Non probabilistic	Consecutive sampling	Monocenter	Retrospectively	Senegal	West and Central	Africa	West Africa	Lower-middle income economies	2006-2010	Adults	Hospital-based	Unclear/Not reported	Unclear/Not reported	General population	HBV	Rapid Diagnostic test	HBsAg
Loaarec	2019	Cross sectional	Non probabilistic	Consecutive sampling	Multicenter	Retrospectively	Kenya	East and Southern	Africa	Eastern Africa	Lower-middle income economies	Unclear/Not reported	Unclear/Not reported	Hospital-based	Unclear/Not reported	On ART	General population	HCV	Immunochromatographic test	Anti-HCV
Loaarec	2019	Cross sectional	Non probabilistic	Consecutive sampling	Multicenter	Retrospectively	Malawi	East and Southern	Africa	Eastern Africa	Low-income economies	Unclear/Not reported	Unclear/Not reported	Hospital-based	Unclear/Not reported	On ART	General population	HCV	Immunochromatographic test	Anti-HCV
Loaarec	2019	Cross sectional	Non probabilistic	Consecutive sampling	Multicenter	Retrospectively	Mozambique	East and Southern	Africa	Eastern Africa	Low-income economies	Unclear/Not reported	Unclear/Not reported	Hospital-based	Unclear/Not reported	On ART	General population	HCV	Immunochromatographic test	Anti-HCV
Loaarec	2019	Cross sectional	Non probabilistic	Consecutive sampling	Multicenter	Retrospectively	Uganda	East and Southern	Africa	Eastern Africa	Low-income economies	Unclear/Not reported	Unclear/Not reported	Hospital-based	Unclear/Not reported	On ART	General population	HCV	Immunochromatographic test	Anti-HCV
Lodenyo	2000	Cross sectional	Non probabilistic	Consecutive sampling	Monocenter	Prospetively	South Africa	East and Southern	Africa	Southern Africa	Upper-middle-income economies	May/1999	Adults	Hospital-based	Unclear/Not reported	Unclear/Not reported	General population	HCV	Indirect ELISA	Anti-HCV
Lodenyo	2000	Cross sectional	Non probabilistic	Consecutive sampling	Monocenter	Prospetively	South Africa	East and Southern	Africa	Southern Africa	Upper-middle-income economies	May/1999	Adults	Hospital-based	Unclear/Not reported	Unclear/Not reported	General population	HBV	Direct ELISA	HBeAg
Lodenyo	2000	Cross sectional	Non probabilistic	Consecutive sampling	Monocenter	Prospetively	South Africa	East and Southern	Africa	Southern Africa	Upper-middle-income economies	May/1999	Adults	Hospital-based	Unclear/Not reported	Unclear/Not reported	General population	HBV	Direct ELISA	HBsAg
Lukhwareni	2009	Cross sectional	Non probabilistic	Consecutive sampling	Monocenter	Retrospectively	South Africa	East and Southern	Africa	Southern Africa	Upper-middle-income economies	Aug/2004-Mar/2007	All ages	Hospital-based	Unclear/Not reported	On ART	General population	HBV	Enzyme immunoassay (EIA)	HBsAg
Maaref	2011	Cross sectional	Non probabilistic	Consecutive sampling	Monocenter	Prospetively	Tunisia	North Africa and Middle East	Eastern Mediterranean	Northern Africa	Lower-middle income economies	Jan/2006-Dec/2006	Unclear/Not reported	Hospital-based	Unclear/Not reported	Unclear/Not reported	General population	HCV	Enzyme immunoassay (EIA)	Anti-HCV
Maaref	2011	Cross sectional	Non probabilistic	Consecutive sampling	Monocenter	Prospetively	Tunisia	North Africa and Middle East	Eastern Mediterranean	Northern Africa	Lower-middle income economies	Jan/2006-Dec/2006	Unclear/Not reported	Hospital-based	Unclear/Not reported	Unclear/Not reported	General population	HBV	Enzyme immunoassay (EIA)	HBsAg
Mabayoje	2007	Cross sectional	Non probabilistic	Consecutive sampling	Monocenter	Prospetively	Nigeria	West and Central	Africa	West Africa	Lower-middle income economies	Jul/2004-Dec/2005	Adults	Hospital-based	Unclear/Not reported	Unclear/Not reported	Blood donors	HCV	Rapid Diagnostic test	Anti-HCV
Mabayoje	2007	Cross sectional	Non probabilistic	Consecutive sampling	Monocenter	Prospetively	Nigeria	West and Central	Africa	West Africa	Lower-middle income economies	Jul/2004-Dec/2005	Adults	Hospital-based	Unclear/Not reported	Unclear/Not reported	Blood donors	HBV	Rapid Diagnostic test	HBsAg
Mabayoje	2013	Cross sectional	Non probabilistic	Consecutive sampling	Monocenter	Prospetively	Nigeria	West and Central	Africa	West Africa	Lower-middle income economies	Sep/2012-Jan/2013	All ages	Hospital-based	Unclear/Not reported	Unclear/Not reported	General population	HCV	Indirect ELISA	Anti-HCV
Mabeya	2016	Cross sectional	Probabilistic	Stratified sampling	Multicenter	Prospetively	Kenya	East and Southern	Africa	Eastern Africa	Lower-middle income economies	Oct/2009-Dec/2009	All ages	Hospital-based	Unclear/Not reported	Unclear/Not reported	General population	HCV	Indirect ELISA	Anti-HCV
Mabeya	2016	Cross sectional	Probabilistic	Stratified sampling	Multicenter	Prospetively	Kenya	East and Southern	Africa	Eastern Africa	Lower-middle income economies	Oct/2009-Dec/2009	All ages	Hospital-based	Unclear/Not reported	Unclear/Not reported	General population	HBV	Direct ELISA	HBsAg
Magaji	2021	Cross sectional	Non probabilistic	Consecutive sampling	Multicenter	Prospetively	Nigeria	West and Central	Africa	West Africa	Lower-middle income economies	Nov/2017-Apr/2018	Adults	Hospital-based	Unclear/Not reported	Unclear/Not reported	Pregnant women	HBV	Rapid Diagnostic test	HBsAg
Magoro	2016	Cross sectional	Non probabilistic	Consecutive sampling	Monocenter	Retrospectively	Cameroon	West and Central	Africa	Central Africa	Lower-middle income economies	Feb/2013-Dec/2013	All ages	Hospital-based	Unclear/Not reported	Unclear/Not reported	General population	HBV	Direct ELISA	HBsAg
Makanera	2019	Cross sectional	Probabilistic	Systematic sampling	Monocenter	Prospetively	Guinea	West and Central	Africa	West Africa	Low-income economies	Jul/2016-Apr/2017	All ages	Hospital-based	Unclear/Not reported	Unclear/Not reported	General population	HBV	Rapid Diagnostic test	HBsAg
Manyazewal	2014	Cross sectional	Probabilistic	Simple random sampling	Multicenter	Prospetively	Ethiopia	East and Southern	Africa	Eastern Africa	Low-income economies	Unclear/Not reported	Adults	Hospital-based	Unclear/Not reported	On ART	General population	HCV	Rapid Diagnostic test	Anti-HCV
Manyazewal	2014	Cross sectional	Probabilistic	Simple random sampling	Multicenter	Prospetively	Ethiopia	East and Southern	Africa	Eastern Africa	Low-income economies	Unclear/Not reported	Adults	Hospital-based	Unclear/Not reported	On ART	General population	HBV	Rapid Diagnostic test	HBeAg
Manyazewal	2014	Cross sectional	Probabilistic	Simple random sampling	Multicenter	Prospetively	Ethiopia	East and Southern	Africa	Eastern Africa	Low-income economies	Unclear/Not reported	Adults	Hospital-based	Unclear/Not reported	On ART	General population	HBV	Rapid Diagnostic test	HBsAg
Matee	2006	Cross sectional	Non probabilistic	Consecutive sampling	Monocenter	Prospetively	Tanzania	East and Southern	Africa	Eastern Africa	Lower-middle income economies	Apr/2004-May/2005	Adults	Hospital-based	Unclear/Not reported	Unclear/Not reported	Blood donors	HBV	Direct ELISA	HBsAg
Matthews	2015	Cross sectional	Non probabilistic	Consecutive sampling	Monocenter	Retrospectively	Botswana	East and Southern	Africa	Southern Africa	Upper-middle-income economies	2004-2013	Unclear/Not reported	Hospital-based	Unclear/Not reported	Unclear/Not reported	General population	HBV	Enzyme immunoassay (EIA)	HBsAg
Matthews	2016	Cross sectional	Non probabilistic	Convenience sampling	Monocenter	Retrospectively	Botswana	East and Southern	Africa	Southern Africa	Upper-middle-income economies	Unclear/Not reported	Adults	Hospital-based	Unclear/Not reported	ART naïve	General population	HBV	Enzyme immunoassay (EIA)	HBsAg
Matthews	2015	Cross sectional	Non probabilistic	Consecutive sampling	Monocenter	Retrospectively	South Africa	East and Southern	Africa	Southern Africa	Upper-middle-income economies	2004-2013	Unclear/Not reported	Hospital-based	Unclear/Not reported	Unclear/Not reported	General population	HBV	Enzyme immunoassay (EIA)	HBsAg
Matthews	2016	Cross sectional	Non probabilistic	Convenience sampling	Monocenter	Retrospectively	South Africa	East and Southern	Africa	Southern Africa	Upper-middle-income economies	Unclear/Not reported	Adults	Hospital-based	Unclear/Not reported	ART naïve	General population	HBV	Enzyme immunoassay (EIA)	HBsAg
Mavenyengwa	2014	Cross sectional	Non probabilistic	Consecutive sampling	Multicenter	Prospetively	Namibia	East and Southern	Africa	Southern Africa	Upper-middle-income economies	Jan/2012-Dec/2012	Adults	Hospital-based	Unclear/Not reported	Unclear/Not reported	Blood donors	HCV	Chemiluminescent enzyme immunoassay (CLEIA)	Anti-HCV
Mavenyengwa	2014	Cross sectional	Non probabilistic	Consecutive sampling	Multicenter	Prospetively	Namibia	East and Southern	Africa	Southern Africa	Upper-middle-income economies	Jan/2012-Dec/2012	Adults	Hospital-based	Unclear/Not reported	Unclear/Not reported	Blood donors	HBV	Electro-chemiluminescence immunoassay (ECLIA)	HBsAg
Mayaki	2013	Cross sectional	Non probabilistic	Consecutive sampling	Monocenter	Prospetively	Niger	West and Central	Africa	West Africa	Low-income economies	2010	Adults	Hospital-based	Unclear/Not reported	Unclear/Not reported	Blood donors	HBV and HCV	Rapid Diagnostic test	Anti-HCV, HBsAg
Mayaki	2013	Cross sectional	Non probabilistic	Consecutive sampling	Monocenter	Prospetively	Niger	West and Central	Africa	West Africa	Low-income economies	2010	Adults	Hospital-based	Unclear/Not reported	Unclear/Not reported	Blood donors	HBV	Rapid Diagnostic test	HBsAg
Mayaphi	2012	Cross sectional	Non probabilistic	Consecutive sampling	Monocenter	Prospetively	South Africa	East and Southern	Africa	Southern Africa	Upper-middle-income economies	Jun/2007-Jan/2009	Adults	Hospital-based	Unclear/Not reported	Unclear/Not reported	General population	HBV	Enzyme immunoassay (EIA)	HBeAg
Mayaphi	2012	Cross sectional	Non probabilistic	Consecutive sampling	Monocenter	Prospetively	South Africa	East and Southern	Africa	Southern Africa	Upper-middle-income economies	Jun/2007-Jan/2009	Adults	Hospital-based	Unclear/Not reported	Unclear/Not reported	General population	HBV	Enzyme immunoassay (EIA)	HBsAg

Mbanya	2003	Cross sectional	Non probabilistic	Consecutive sampling	Monocenter	Prospetively	Cameroon	West and Central	Africa	Central Africa	Lower-middle income economies	Jul/2001-Oct/2001	Adults	Hospital-based	Unclear/Not reported	Unclear/Not reported	Blood donors	HCV	Rapid Diagnostic test	Anti-HCV
Mbanya	2003	Cross sectional	Non probabilistic	Consecutive sampling	Monocenter	Prospetively	Cameroon	West and Central	Africa	Central Africa	Lower-middle income economies	Jul/2001-Oct/2001	Adults	Hospital-based	Unclear/Not reported	Unclear/Not reported	Blood donors	HBV	Rapid Diagnostic test	HBsAg
Mboto	2010	Cross sectional	Non probabilistic	Consecutive sampling	Monocenter	Prospetively	Gambia	West and Central	Africa	West Africa	Low-income economies	Jul/2022-Dec/2002	All ages	Hospital-based	Unclear/Not reported	Unclear/Not reported	General population	HCV	Enzyme immunoassay (EIA)	Anti-HCV
Mboto	2009	Cross sectional	Non probabilistic	Consecutive sampling	Monocenter	Prospetively	Gambia	West and Central	Africa	West Africa	Low-income economies	Jul/2002-Dec/2002	All ages	Hospital-based	Unclear/Not reported	Unclear/Not reported	General population	HCV	Enzyme immunoassay (EIA)	Anti-HCV
Mdlalose	2016	Cross sectional	Probabilistic	Simple random sampling	Monocenter	Retrospectively	South Africa	East and Southern	Africa	Southern Africa	Upper-middle-income economies	Jun/2011- Dec/2014	Children	Hospital-based	Unclear/Not reported	Unclear/Not reported	General population	HBV	Classical PCR	Viral DNA
Mirzoyan	1999	Cross sectional	Probabilistic	Respondent-driven sampling	Monocenter	Prospetively	Libya	North Africa and Middle East	Eastern Mediterranean	Northern Africa	Upper-middle-income economies	Jul/2010-Sep/2010	Unclear/Not reported	Community-based	Unclear/Not reported	Unclear/Not reported	Injecting drug users	HCV	Unclear/Not reported	Anti-HCV
Mirzoyan	1999	Cross sectional	Probabilistic	Respondent-driven sampling	Monocenter	Prospetively	Libya	North Africa and Middle East	Eastern Mediterranean	Northern Africa	Upper-middle-income economies	Jul/2010-Sep/2010	Unclear/Not reported	Community-based	Unclear/Not reported	Unclear/Not reported	Injecting drug users	HBV	Unclear/Not reported	HBsAg
Molu	2018	Cross sectional	Non probabilistic	Consecutive sampling	Monocenter	Retrospectively	Cameroon	West and Central	Africa	Central Africa	Lower-middle income economies	Jan/2008-Apr/2014	All ages	Hospital-based	Rural	ART naïve	General population	HBV	Immunochromatographic test	HBsAg
Moore	2010	Cohort (Baseline data)	Non probabilistic	Consecutive sampling	Monocenter	Prospetively	Malawi	East and Southern	Africa	Eastern Africa	Low-income economies	2005	Unclear/Not reported	Hospital-based	Unclear/Not reported	ART naïve	General population	HCV	Indirect ELISA	Anti-HCV
Moore	2010	Cohort (Baseline data)	Non probabilistic	Consecutive sampling	Monocenter	Prospetively	Malawi	East and Southern	Africa	Eastern Africa	Low-income economies	2005	Unclear/Not reported	Hospital-based	Unclear/Not reported	ART naïve	General population	HBV and HCV	Direct ELISA, Indirect ELISA	Anti-HCV, HBsAg
Moore	2010	Cohort (Baseline data)	Non probabilistic	Consecutive sampling	Monocenter	Prospetively	Malawi	East and Southern	Africa	Eastern Africa	Low-income economies	2005	Unclear/Not reported	Hospital-based	Unclear/Not reported	ART naïve	General population	HBV	Direct ELISA	HBsAg
Mphahlele	2006	Case control	Non probabilistic	Consecutive sampling	Monocenter	Retrospectively	South Africa	East and Southern	Africa	Southern Africa	Upper-middle-income economies	jan/1998-Mar/2001	Adults	Hospital-based	Unclear/Not reported	Unclear/Not reported	General population	HCV	Enzyme immunoassay (EIA)	Anti-HCV
Mphahlele	2006	Case control	Non probabilistic	Consecutive sampling	Monocenter	Retrospectively	South Africa	East and Southern	Africa	Southern Africa	Upper-middle-income economies	jan/1998-Mar/2001	Adults	Hospital-based	Unclear/Not reported	Unclear/Not reported	General population	HBV	Chemiluminescent enzyme immunoassay (CLEIA)	HBsAg
Mphahlele	2006	Case control	Non probabilistic	Consecutive sampling	Monocenter	Retrospectively	South Africa	East and Southern	Africa	Southern Africa	Upper-middle-income economies	jan/1998-Mar/2001	Adults	Hospital-based	Unclear/Not reported	Unclear/Not reported	General population	HBV	Classical PCR	Viral DNA
Mpody	2019	Clinical Trial (Baseline data)	Probabilistic	Simple random sampling	Multicenter	Prospetively	Democratic Republic o	West and Central	Africa	Central Africa	Low-income economies	Nov/2016-Jun/2018	Adults	Hospital-based	Unclear/Not reported	On ART	Pregnant women	HBV	Rapid Diagnostic test	HBsAg
Mpody	2019	Clinical Trial (Baseline data)	Probabilistic	Simple random sampling	Multicenter	Prospetively	Democratic Republic o	West and Central	Africa	Central Africa	Low-income economies	Nov/2016-Jun/2018	Adults	Hospital-based	Unclear/Not reported	ART naïve/ On ART	Pregnant women	HBV	Rapid Diagnostic test	HBsAg
Mpody	2019	Clinical Trial (Baseline data)	Probabilistic	Simple random sampling	Multicenter	Prospetively	Democratic Republic o	West and Central	Africa	Central Africa	Low-income economies	Nov/2016-Jun/2018	Adults	Hospital-based	Unclear/Not reported	ART naïve/ On ART	Pregnant women	HBV	Rapid Diagnostic test	HBsAg
Msomi	2020	Cross sectional	Probabilistic	Simple random sampling	Monocenter	Retrospectively	South Africa	East and Southern	Africa	Southern Africa	Upper-middle-income economies	2018	Adults	Hospital-based	Urban/Rural	On ART	General population	HBV	Immunoassay kit	HBsAg
Msuya	2006	Cross sectional	Non probabilistic	Consecutive sampling	Monocenter	Prospetively	Tanzania	East and Southern	Africa	Eastern Africa	Lower-middle income economies	Sept/1999-Dec/1999	Adults	Hospital-based	Unclear/Not reported	Unclear/Not reported	General population	HCV	Enzyme immunoassay (EIA)	Anti-HCV
Msuya	2006	Cross sectional	Non probabilistic	Consecutive sampling	Monocenter	Prospetively	Tanzania	East and Southern	Africa	Eastern Africa	Lower-middle income economies	Sept/1999-Dec/1999	Adults	Hospital-based	Unclear/Not reported	Unclear/Not reported	General population	HBV	Enzyme immunoassay (EIA)	HBsAg
Mudawi	2014	Cross sectional	Non probabilistic	Consecutive sampling	Multicenter	Prospetively	Sudan	North Africa and Middle East	Eastern Mediterranean	Northern Africa	Low-income economies	Dec/2010-Jun/2012	Adults	Hospital-based	Unclear/Not reported	ART naïve	General population	HCV	Immunoassay kit	Anti-HCV
Mudawi	2014	Cross sectional	Non probabilistic	Consecutive sampling	Multicenter	Prospetively	Sudan	North Africa and Middle East	Eastern Mediterranean	Northern Africa	Low-income economies	Dec/2010-Jun/2012	Adults	Hospital-based	Unclear/Not reported	ART naïve	General population	HBV	Immunoassay kit	HBsAg
Mudawi	2014	Cross sectional	Non probabilistic	Consecutive sampling	Multicenter	Prospetively	Sudan	North Africa and Middle East	Eastern Mediterranean	Northern Africa	Low-income economies	Dec/2010-Jun/2012	Adults	Hospital-based	Unclear/Not reported	ART naïve	General population	HBV	Immunoassay kit	Viral DNA
Mugusi S	2012	Cohort (Baseline data)	Non probabilistic	Consecutive sampling	Multicenter	Prospetively	Tanzania	East and Southern	Africa	Eastern Africa	Lower-middle income economies	Sept/2007-Jun/2010	Unclear/Not reported	Hospital-based	Unclear/Not reported	Unclear/Not reported	General population	HCV	Unclear/Not reported	Anti-HCV
Mugusi S	2012	Cohort (Baseline data)	Non probabilistic	Consecutive sampling	Multicenter	Prospetively	Tanzania	East and Southern	Africa	Eastern Africa	Lower-middle income economies	Sept/2007-Jun/2010	Unclear/Not reported	Hospital-based	Unclear/Not reported	Unclear/Not reported	General population	HBV	Unclear/Not reported	HBsAg
Mullis	2013	Cross sectional	Non probabilistic	Consecutive sampling	Monocenter	Prospetively	Uganda	East and Southern	Africa	Eastern Africa	Low-income economies	Unclear/Not reported	Unclear/Not reported	Hospital-based	Unclear/Not reported	Unclear/Not reported	General population	HCV	Direct ELISA	Anti-HCV
Mulu	2013	Cross sectional	Non probabilistic	Convenience sampling	Monocenter	Prospetively	Ethiopia	East and Southern	Africa	Eastern Africa	Low-income economies	Jul/2012-Dec/2012	Adults	Hospital-based	Unclear/Not reported	On ART	General population	HCV	Indirect ELISA	Anti-HCV
Mulu	2013	Cross sectional	Non probabilistic	Convenience sampling	Monocenter	Prospetively	Ethiopia	East and Southern	Africa	Eastern Africa	Low-income economies	Jul/2012-Dec/2012	Adults	Hospital-based	Unclear/Not reported	On ART	General population	HBV and HCV	Direct ELISA, Indirect ELISA	Anti-HCV, HBsAg
Mulu	2013	Cross sectional	Non probabilistic	Convenience sampling	Monocenter	Prospetively	Ethiopia	East and Southern	Africa	Eastern Africa	Low-income economies	Jul/2012-Dec/2012	Adults	Hospital-based	Unclear/Not reported	On ART	General population	HBV	Direct ELISA	HBsAg
Munyemana	2021	Cross sectional	Non probabilistic	Consecutive sampling	Monocenter	Prospetively	Rwanda	East and Southern	Africa	Eastern Africa	Low-income economies	Jan/2019-Jun/2019	All ages	Hospital-based	Unclear/Not reported	Unclear/Not reported	General population	HCV	Rapid Diagnostic test	Anti-HCV
Muriuki	2013	Cross sectional	Probabilistic	Stratified sampling	Multicenter	Prospetively	Kenya	East and Southern	Africa	Eastern Africa	Lower-middle income economies	Oct/2009-Dec/2009	All ages	Hospital-based	Unclear/Not reported	Unclear/Not reported	General population	HCV	Indirect ELISA	Anti-HCV
Muriuki	2013	Cross sectional	Probabilistic	Stratified sampling	Multicenter	Prospetively	Kenya	East and Southern	Africa	Eastern Africa	Lower-middle income economies	Oct/2009-Dec/2009	All ages	Hospital-based	Unclear/Not reported	Unclear/Not reported	General population	HBV and HCV	Direct ELISA, Indirect ELISA	Anti-HCV, HBsAg
Muriuki	2013	Cross sectional	Probabilistic	Stratified sampling	Multicenter	Prospetively	Kenya	East and Southern	Africa	Eastern Africa	Lower-middle income economies	Oct/2009-Dec/2009	All ages	Hospital-based	Unclear/Not reported	Unclear/Not reported	General population	HBV	Direct ELISA	HBsAg
Muro	2013	Cross sectional	Non probabilistic	Consecutive sampling	Monocenter	Retrospectively	Tanzania	East and Southern	Africa	Eastern Africa	Lower-middle income economies	Dec/2006-Mar/2008	Children	Hospital-based	Unclear/Not reported	Unclear/Not reported	General population	HCV	Indirect ELISA	Anti-HCV
Muro	2013	Cross sectional	Non probabilistic	Consecutive sampling	Monocenter	Retrospectively	Tanzania	East and Southern	Africa	Eastern Africa	Lower-middle income economies	Dec/2006-Mar/2008	Children	Hospital-based	Unclear/Not reported	Unclear/Not reported	General population	HBV	Direct ELISA	HBsAg
Musa	2015	Cross sectional	Non probabilistic	Consecutive sampling	Monocenter	Prospetively	Nigeria	West and Central	Africa	West Africa	Lower-middle income economies	Nov/2008-Aug/2010	Children	Hospital-based	Unclear/Not reported	Unclear/Not reported	General population	HCV	Immunoassay kit	Anti-HCV
Musyoki	2015	Cross sectional	Non probabilistic	Consecutive sampling	Monocenter	Prospetively	South Africa	East and Southern	Africa	Southern Africa	Upper-middle-income economies	Jul/2010-Jun/2012	Adults	Hospital-based	Unclear/Not reported	Unclear/Not reported	Patients with cancer	HCV	Indirect ELISA	Anti-HCV
Musyoki	2015	Cross sectional	Non probabilistic	Consecutive sampling	Monocenter	Prospetively	South Africa	East and Southern	Africa	Southern Africa	Upper-middle-income economies	Jul/2010-Jun/2012	Adults	Hospital-based	Unclear/Not reported	Unclear/Not reported	Patients with cancer	HBV	Immunoassay kit	HBsAg
Musyoki	2015	Cross sectional	Non probabilistic	Consecutive sampling	Monocenter	Prospetively	South Africa	East and Southern	Africa	Southern Africa	Upper-middle-income economies	Jul/2010-Jun/2012	Adults	Hospital-based	Unclear/Not reported	Unclear/Not reported	Patients with cancer	HBV	Classical PCR	Viral DNA
Musyoki	2015	Cross sectional	Non probabilistic	Consecutive sampling	Monocenter	Prospetively	South Africa	East and Southern	Africa	Southern Africa	Upper-middle-income economies	Jul/2010-Jun/2012	Adults	Hospital-based	Unclear/Not reported	Unclear/Not reported	Patients with cancer	HCV	Classical PCR	Viral RNA
Mutwa	2013	Cross sectional	Non probabilistic	Consecutive sampling	Monocenter	Prospetively	Rwanda	East and Southern	Africa	Eastern Africa	Low-income economies	Feb/2010-Oct/2010	Children	Hospital-based	Unclear/Not reported	On ART	General population	HBV	Enzyme immunoassay (EIA)	HBsAg
Mwatelah	2015	Cross sectional	Non probabilistic	Consecutive sampling	Monocenter	Prospetively	Kenya	East and Southern	Africa	Eastern Africa	Lower-middle income economies	Unclear/Not reported	Unclear/Not reported	Hospital-based	Unclear/Not reported	Unclear/Not reported	Injecting drug users	HCV	Rapid Diagnostic test	Anti-HCV
Mweemba	2014	Cross sectional	Non probabilistic	Consecutive sampling	Monocenter	Prospetively	Zambia	East and Southern	Africa	Eastern Africa	Lower-middle income economies	Jan/2011-Dec/2013	Adults	Hospital-based	Unclear/Not reported	Unclear/Not reported	General population	HBV	Direct ELISA	HBsAg
Nagalo	2011	Cross sectional	Non probabilistic	Consecutive sampling	Monocenter	Prospetively	Burkina Faso	West and Central	Africa	West Africa	Low-income economies	Jan/2009-Dec/2009	Adults	Hospital-based	Unclear/Not reported	Unclear/Not reported	Blood donors	HCV	Indirect ELISA	Anti-HCV
Nagalo	2011	Cross sectional	Non probabilistic	Consecutive sampling	Monocenter	Prospetively	Burkina Faso	West and Central	Africa	West Africa	Low-income economies	Jan/2009-Dec/2009	Adults	Hospital-based	Unclear/Not reported	Unclear/Not reported	Blood donors	HBV and HCV	Direct ELISA, Indirect ELISA	Anti-HCV, HBsAg
Nagalo	2011	Cross sectional	Non probabilistic	Consecutive sampling	Monocenter	Prospetively	Burkina Faso	West and Central	Africa	West Africa	Low-income economies	Jan/2009-Dec/2009	Adults	Hospital-based	Unclear/Not reported	Unclear/Not reported	Blood donors	HBV	Direct ELISA	HBsAg
Nagalo	2012	Cross sectional	Non probabilistic	Consecutive sampling	Multicenter	Prospetively	Burkina Faso	West and Central	Africa	West Africa	Low-income economies	Jan/2009-Dec/2009	Unclear/Not reported	Hospital-based	Unclear/Not reported	Unclear/Not reported	Blood donors	HCV	Indirect ELISA	Anti-HCV
Nagalo	2012	Cross sectional	Non probabilistic	Consecutive sampling	Multicenter	Prospetively	Burkina Faso	West and Central	Africa	West Africa	Low-income economies	Jan/2009-Dec/2009	Unclear/Not reported	Hospital-based	Unclear/Not reported	Unclear/Not reported	Blood donors	HBV	Direct ELISA	HBsAg
Nagu	2008	Cross sectional	Non probabilistic	Consecutive sampling	Monocenter	Prospetively	Tanzania	East and Southern	Africa	Eastern Africa	Lower-middle income economies	Apr/2006-Sep/2006	Adults	Hospital-based	Unclear/Not reported	ART naïve	General population	HCV	Indirect ELISA	Anti-HCV
Nagu	2008	Cross sectional	Non probabilistic	Consecutive sampling	Monocenter	Prospetively	Tanzania	East and Southern	Africa	Eastern Africa	Lower-middle income economies	Apr/2006-Sep/2006	Adults	Hospital-based	Unclear/Not reported	ART naïve	General population	HBV and HCV	Direct ELISA, Indirect ELISA	Anti-HCV, HBsAg
Nagu	2008	Cross sectional	Non probabilistic	Consecutive sampling	Monocenter	Prospetively	Tanzania	East and Southern	Africa	Eastern Africa	Lower-middle income economies	Apr/2006-Sep/2006	Adults	Hospital-based	Unclear/Not reported	ART naïve	General population	HBV	Direct ELISA	HBsAg
Nagu	2008	Cross sectional	Non probabilistic	Consecutive sampling	Monocenter	Prospetively	Tanzania	East and Southern	Africa	Eastern Africa	Lower-middle income economies	Apr/2006-Sep/2006	Adults	Hospital-based	Unclear/Not reported	ART naïve	General population	HBV	Direct ELISA, Indirect ELISA	HBsAg + IgM anti-HBc
Nakwagala	2002	Cross sectional	Non probabilistic	Consecutive sampling	Monocenter	Prospetively	Uganda	East and Southern	Africa	Eastern Africa	Low-income economies	Dec/1999-Jan/2000	Adults	Hospital-based	Unclear/Not reported	Unclear/Not reported	General population	HBV	Direct ELISA	HBeAg
Nakwagala	2002	Cross sectional	Non probabilistic	Consecutive sampling	Monocenter	Prospetively	Uganda	East and Southern	Africa	Eastern Africa	Low-income economies	Dec/1999-Jan/2000	Adults	Hospital-based	Unclear/Not reported	Unclear/Not reported	General population	HBV	Direct ELISA	HBsAg
Naniche	2011	Cross sectional	Non probabilistic	Consecutive sampling	Monocenter	Prospetively	Mozambique	East and Southern	Africa	Eastern Africa	Low-income economies	Apr/2006–Nov/2008	Unclear/Not reported	Hospital-based	Unclear/Not reported	On ART	General population	HCV	Indirect ELISA	Anti-HCV
Naniche	2011	Cross sectional	Non probabilistic	Consecutive sampling	Monocenter	Prospetively	Mozambique	East and Southern	Africa	Eastern Africa	Low-income economies	Apr/2006–Nov/2008	Unclear/Not reported	Hospital-based	Unclear/Not reported	On ART	General population	HBV	Rapid Diagnostic test	HBsAg
Ndjomou	2002	Cross sectional	Non probabilistic	Consecutive sampling	Multicenter	Prospetively	Cameroon	West and Central	Africa	Central Africa	Lower-middle income economies	1998	Unclear/Not reported	Hospital-based	Unclear/Not reported	Unclear/Not reported	General population	HCV	Classical RT-PCR	Viral RNA
Ndjoyi-Mbiguino	2018	Cross sectional	Non probabilistic	Consecutive sampling	Monocenter	Retrospectively	Gabon	West and Central	Africa	Central Africa	Upper-middle-income economies	Nov/2015-Apr/2016	Unclear/Not reported	Hospital-based	Unclear/Not reported	ART naïve/ On ART	General population	HCV	Indirect ELISA	Anti-HCV
Ndow	2017	Cohort (Baseline data)	Non probabilistic	Consecutive sampling	Monocenter	Prospetively	Gambia	West and Central	Africa	West Africa	Low-income economies	Sep/2015-Feb/2016	Adults	Hospital-based	Unclear/Not reported	Unclear/Not reported	General population	HBV	Rapid Diagnostic test	HBsAg
Ndow	2017	Cohort (Baseline data)	Non probabilistic	Consecutive sampling	Monocenter	Prospetively	Gambia	West and Central	Africa	West Africa	Low-income economies	Sep/2015-Feb/2016	Adults	Hospital-based	Unclear/Not reported	Unclear/Not reported	General population	CFR	Rapid Diagnostic test	HBsAg
N'Dri-Yoman	2010	Cross sectional	Non probabilistic	Consecutive sampling	Multicenter	Prospetively	Ivory Coast	West and Central	Africa	West Africa	Lower-middle income economies	Jun/2006-Sep/2006	Adults	Hospital-based	Unclear/Not reported	ART naïve	General population	HBV	Enzyme immunoassay (EIA)	HBeAg
N'Dri-Yoman	2010	Cross sectional	Non probabilistic	Consecutive sampling	Multicenter	Prospetively	Ivory Coast	West and Central	Africa	West Africa	Lower-middle income economies	Jun/2006-Sep/2006	Adults	Hospital-based	Unclear/Not reported	ART naïve	General population	HBV	Enzyme immunoassay (EIA)	HBsAg
Newton	2015	Cross sectional	Non probabilistic	Consecutive sampling	Monocenter	Prospetively	Nigeria	West and Central	Africa	West Africa	Lower-middle income economies	Jun/2012-Feb/2013	All ages	Hospital-based	Unclear/Not reported	Unclear/Not reported	General population	HCV	Rapid Diagnostic test	Anti-HCV
Nkengasong	1994	Cross sectional	Probabilistic	Systematic sampling	Monocenter	Prospetively	Cameroon	West and Central	Africa	Central Africa	Lower-middle income economies	Jan/1992-Feb/1993	Adults	Hospital-based	Unclear/Not reported	Unclear/Not reported	General population	HCV	Indirect ELISA	Anti-HCV
Nnakenyi	2020	Cross sectional	Non probabilistic	Consecutive sampling	Monocenter	Retrospectively	Nigeria	West and Central	Africa	West Africa	Lower-middle income economies	2008-2013	Unclear/Not reported	Hospital-based	Unclear/Not reported	Unclear/Not reported	General population	HCV	Indirect ELISA	Anti-HCV
Nnakenyi	2020	Cross sectional	Non probabilistic	Consecutive sampling	Monocenter	Retrospectively	Nigeria	West and Central	Africa	West Africa	Lower-middle income economies	2008-2013	Unclear/Not reported	Hospital-based	Unclear/Not reported	Unclear/Not reported	General population	HBV and HCV	Direct ELISA, Indirect ELISA	Anti-HCV, HBsAg
Nnakenyi	2020	Cross sectional	Non probabilistic	Consecutive sampling	Monocenter	Retrospectively	Nigeria	West and Central	Africa	West Africa	Lower-middle income economies	2008-2013	Unclear/Not reported	Hospital-based	Unclear/Not reported	Unclear/Not reported	General population	HBV	Direct ELISA	HBsAg
Noubiap	2015	Cohort (Baseline data)	Non probabilistic	Convenience sampling	Monocenter	Prospetively	Cameroon	West and Central	Africa	Central Africa	Lower-middle income economies	2000-2013	Unclear/Not reported	Hospital-based	Unclear/Not reported	ART naïve/ On ART	General population	HCV	Indirect ELISA	Anti-HCV
Noubiap	2015	Cohort (Baseline data)	Non probabilistic	Convenience sampling	Monocenter	Prospetively	Cameroon	West and Central	Africa	Central Africa	Lower-middle income economies	2000-2013	Unclear/Not reported	Hospital-based	Unclear/Not reported	ART naïve/ On ART	General population	HBV	Direct ELISA	HBeAg
Noubiap	2015	Cohort (Baseline data)	Non probabilistic	Convenience sampling	Monocenter	Prospetively	Cameroon	West and Central	Africa	Central Africa	Lower-middle income economies	2000-2013	Unclear/Not reported	Hospital-based	Unclear/Not reported	ART naïve/ On ART	General population	HBV and HCV	Direct ELISA, Indirect ELISA	Anti-HCV, HBsAg
Noubiap	2015	Cohort (Baseline data)	Non probabilistic	Convenience sampling	Monocenter	Prospetively	Cameroon	West and Central	Africa	Central Africa	Lower-middle income economies	2000-2013	Unclear/Not reported	Hospital-based	Unclear/Not reported	ART naïve/ On ART	General population	HBV	Direct ELISA	HBsAg
Noubiap	2013	Cross sectional	Non probabilistic	Consecutive sampling	Monocenter	Retrospectively	Cameroon	West and Central	Africa	Central Africa	Lower-middle income economies	Dec/2011-May/2012	Adults	Hospital-based	Unclear/Not reported	Unclear/Not reported	Blood donors	HCV	Immunochromatographic test	Anti-HCV
Noubiap	2013	Cross sectional	Non probabilistic	Consecutive sampling	Monocenter	Retrospectively	Cameroon	West and Central	Africa	Central Africa	Lower-middle income economies	Dec/2011-May/2012	Adults	Hospital-based	Unclear/Not reported	Unclear/Not reported	Blood donors	HBV	Immunoassay kit	HBsAg
Ntagirabiri	2012	Cross sectional	Non probabilistic	Consecutive sampling	Monocenter	Retrospectively	Burundi	West and Central	Africa	Eastern Africa	Low-income economies	2004-2009	Unclear/Not reported	Hospital-based	Unclear/Not reported	Unclear/Not reported	General population	HCV	Rapid Diagnostic test	Anti-HCV
Nwokedi	2006	Cross sectional	Non probabilistic	Consecutive sampling	Monocenter	Prospetively	Nigeria	West and Central	Africa	West Africa	Lower-middle income economies	Feb/2002-Mar/2003	Adults	Hospital-based	Unclear/Not reported	Unclear/Not reported	General population	HBV	Rapid Diagnostic test	HBsAg
Nwolisa	2013	Cross sectional	Non probabilistic	Consecutive sampling	Monocenter	Prospetively	Nigeria	West and Central	Africa	West Africa	Lower-middle income economies	Feb/2010-Jul/2010	Children	Hospital-based	Unclear/Not reported	Unclear/Not reported	General population	HBV	Rapid Diagnostic test	HBsAg
Nyirenda	2008	Cross sectional	Non probabilistic	Consecutive sampling	Monocenter	Prospetively	Malawi	East and Southern	Africa	Eastern Africa	Low-income economies	2004	Unclear/Not reported	Hospital-based	Unclear/Not reported	Unclear/Not reported	General population	HBV	Immunoassay kit	HBsAg
Nyirenda	2008	Cross sectional	Non probabilistic	Consecutive sampling	Monocenter	Prospetively	Malawi	East and Southern	Africa	Eastern Africa	Low-income economies	2004	Unclear/Not reported	Hospital-based	Unclear/Not reported	Unclear/Not reported	General population	HBV and HCV	Immunoassay kit	Anti-HCV, HBsAg
Nyirenda	2008	Cross sectional	Non probabilistic	Consecutive sampling	Monocenter	Prospetively	Malawi	East and Southern	Africa	Eastern Africa	Low-income economies	2004	Unclear/Not reported	Hospital-based	Unclear/Not reported	Unclear/Not reported	General population	HCV	Immunoassay kit	Anti-HCV
Obienu	2011	Cross sectional	Non probabilistic	Consecutive sampling	Monocenter	Prospetively	Nigeria	West and Central	Africa	West Africa	Lower-middle income economies	May/2007-Apr/2009.	Unclear/Not reported	Hospital-based	Unclear/Not reported	ART naïve	General population	HCV	Indirect ELISA	Anti-HCV
Ocama	2010	Cohort (Baseline data)	Non probabilistic	Consecutive sampling	Monocenter	Retrospectively	Uganda	East and Southern	Africa	Eastern Africa	Low-income economies	Apr/2004-Apr/2005	Unclear/Not reported	Hospital-based	Unclear/Not reported	On ART	General population	HBV	Direct ELISA	HBsAg
Ocama	2008	Cross sectional	Non probabilistic	Consecutive sampling	Monocenter	Prospetively	Uganda	East and Southern	Africa	Eastern Africa	Low-income economies	May/2004-Mar/2005	Unclear/Not reported	Hospital-based	Unclear/Not reported	ART naïve/ On ART	General population	HCV	Unclear/Not reported	Anti-HCV
Ocama	2008	Cross sectional	Non probabilistic	Consecutive sampling	Monocenter	Prospetively	Uganda	East and Southern	Africa	Eastern Africa	Low-income economies	May/2004-Mar/2005	Unclear/Not reported	Hospital-based	Unclear/Not reported	ART naïve/ On ART	General population	HBV	Unclear/Not reported	HBsAg
Odama	2004	Cross sectional	Non probabilistic	Consecutive sampling	Multicenter	Retrospectively	Nigeria	West and Central	Africa	West Africa	Lower-middle income economies	Unclear/Not reported	Unclear/Not reported	Hospital-based	Unclear/Not reported	Unclear/Not reported	General population	HCV	Indirect ELISA	Anti-HCV
Ogutu	1990	Cross sectional	Non probabilistic	Consecutive sampling	Monocenter	Prospetively	Kenya	East and Southern	Africa	Eastern Africa	Lower-middle income economies	Unclear/Not reported	Unclear/Not reported	Hospital-based	Unclear/Not reported	Unclear/Not reported	General population	HBV	Reverse passive hemagglutination assay	HBsAg
Ogwu-Richard	2015	Cross sectional	Non probabilistic	Consecutive sampling	Multicenter	Prospetively	Nigeria	West and Central	Africa	West Africa	Lower-middle income economies	Unclear/Not reported	Adults	Hospital-based	Unclear/Not reported	On ART	General population	HCV	Immunochromatographic test	Anti-HCV
Ogwu-Richard	2015	Cross sectional	Non probabilistic	Consecutive sampling	Multicenter	Prospetively	Nigeria	West and Central	Africa	West Africa	Lower-middle income economies	Unclear/Not reported	Adults	Hospital-based	Unclear/Not reported	On ART	General population	HBV and HCV	Immunochromatographic test	Anti-HCV, HBsAg
Ogwu-Richard	2015	Cross sectional	Non probabilistic	Consecutive sampling	Multicenter	Prospetively	Nigeria	West and Central	Africa	West Africa	Lower-middle income economies	Unclear/Not reported	Adults	Hospital-based	Unclear/Not reported	On ART	General population	HBV	Immunochromatographic test	HBsAg
Ojide	2015	Cross sectional	Non probabilistic	Consecutive sampling	Monocenter	Prospetively	Nigeria	West and Central	Africa	West Africa	Lower-middle income economies	Apr/2011-Sep/2011	Adults	Hospital-based	Unclear/Not reported	Unclear/Not reported	General population	HCV	Immunochromatographic test	Anti-HCV
Ojide	2015	Cross sectional	Non probabilistic	Consecutive sampling	Monocenter	Prospetively	Nigeria	West and Central	Africa	West Africa	Lower-middle income economies	Apr/2011-Sep/2011	Adults	Hospital-based	Unclear/Not reported	Unclear/Not reported	General population	HBV and HCV	Immunochromatographic test	Anti-HCV, HBsAg
Ojide	2015	Cross sectional	Non probabilistic	Consecutive sampling	Monocenter	Prospetively	Nigeria	West and Central	Africa	West Africa	Lower-middle income economies	Apr/2011-Sep/2011	Adults	Hospital-based	Unclear/Not reported	Unclear/Not reported	General population	HBV	Immunochromatographic test	HBsAg
Okeke	2012	Cross sectional	Non probabilistic	Consecutive sampling	Monocenter	Retrospectively	Nigeria	West and Central	Africa	West Africa	Lower-middle income economies	Jan/2007-Dec/2009	Adults	Hospital-based	Rural	ART naïve/ On ART	Pregnant women	HCV	Indirect ELISA	Anti-HCV
Okeke	2012	Cross sectional	Non probabilistic	Consecutive sampling	Monocenter	Retrospectively	Nigeria	West and Central	Africa	West Africa	Lower-middle income economies	Jan/2007-Dec/2009	Adults	Hospital-based	Unclear/Not reported	ART naïve/ On ART	Pregnant women	HBV and HCV	Direct ELISA, Indirect ELISA	Anti-HCV, HBsAg
Okeke	2012	Cross sectional	Non probabilistic	Consecutive sampling	Monocenter	Retrospectively	Nigeria	West and Central	Africa	West Africa	Lower-middle income economies	Jan/2007-Dec/2009	Adults	Hospital-based	Unclear/Not reported	On ART	Pregnant women	HBV	Direct ELISA	HBsAg
Okocha	2012	Cross sectional	Non probabilistic	Consecutive sampling	Monocenter	Retrospectively	Nigeria	West and Central	Africa	West Africa	Lower-middle income economies	2005-2009	All ages	Hospital-based	Unclear/Not reported	Unclear/Not reported	General population	HBV	Immunochromatographic test	HBsAg
Okonko	2017	Cross sectional	Non probabilistic	Consecutive sampling	Monocenter	Prospetively	Nigeria	West and Central	Africa	West Africa	Lower-middle income economies	Sep/2012-Jun/2013	Adults	Hospital-based	Unclear/Not reported	Unclear/Not reported	Blood donors	HBV	Direct ELISA	HBsAg
Okoth	2017	Cross sectional	Non probabilistic	Convenience sampling	Monocenter	Prospetively	Kenya	East and Southern	Africa	Eastern Africa	Lower-middle income economies	Jul/2009-Nov/2009	Unclear/Not reported	Hospital-based	Unclear/Not reported	ART naïve/ On ART	General population	HCV	Indirect ELISA	Anti-HCV
Okoth	2017	Cross sectional	Non probabilistic	Convenience sampling	Monocenter	Prospetively	Kenya	East and Southern	Africa	Eastern Africa	Lower-middle income economies	Jul/2009-Nov/2009	Unclear/Not reported	Hospital-based	Unclear/Not reported	ART naïve/ On ART	General population	HBV	Direct ELISA	HBsAg
Okwuraiwe	2012	Cohort (Baseline data)	Non probabilistic	Consecutive sampling	Monocenter	Prospetively	Nigeria	West and Central	Africa	West Africa	Lower-middle income economies	Jan/2006-Dec/2009	Unclear/Not reported	Hospital-based	Unclear/Not reported	ART naïve/ On ART	General population	HCV	Indirect ELISA	Anti-HCV
Okwuraiwe	2012	Cohort (Baseline data)	Non probabilistic	Consecutive sampling	Monocenter	Prospetively	Nigeria	West and Central	Africa	West Africa	Lower-middle income economies	Jan/2006-Dec/2009	Unclear/Not reported	Hospital-based	Unclear/Not reported	ART naïve/ On ART	General population	HBV and HCV	Enzyme immunoassay (EIA), Indirect ELISA	Anti-HCV, HBsAg
Okwuraiwe	2012	Cohort (Baseline data)	Non probabilistic	Consecutive sampling	Monocenter	Prospetively	Nigeria	West and Central	Africa	West Africa	Lower-middle income economies	Jan/2006-Dec/2009	Unclear/Not reported	Hospital-based	Unclear/Not reported	On ART	General population	HBV	Enzyme immunoassay (EIA)	HBsAg
Olatunji	2008	Cross sectional	Non probabilistic	Consecutive sampling	Monocenter	Prospetively	Nigeria	West and Central	Africa	West Africa	Lower-middle income economies	Unclear/Not reported	Unclear/Not reported	Hospital-based	Unclear/Not reported	Unclear/Not reported	General population	HCV	Indirect ELISA	Anti-HCV
Olatunji	2008	Cross sectional	Non probabilistic	Consecutive sampling	Monocenter	Prospetively	Nigeria	West and Central	Africa	West Africa	Lower-middle income economies	Unclear/Not reported	Unclear/Not reported	Hospital-based	Unclear/Not reported	Unclear/Not reported	General population	HBV	Agglutination test kits	HBsAg
Olawumi	2014	Cross sectional	Non probabilistic	Consecutive sampling	Monocenter	Prospetively	Nigeria	West and Central	Africa	West Africa	Lower-middle income economies	Jan/2010-Dec/2010	Unclear/Not reported	Hospital-based	Unclear/Not reported	ART naïve	General population	HBV	Indirect ELISA	HBsAg
Olokoba	2008	Cross sectional	Non probabilistic	Consecutive sampling	Monocenter	Prospetively	Nigeria	West and Central	Africa	West Africa	Lower-middle income economies	Dec/2006-Dec/2007	Unclear/Not reported	Hospital-based	Unclear/Not reported	Unclear/Not reported	General population	HCV	Indirect ELISA	Anti-HCV
Omatola	2019	Cross sectional	Non probabilistic	Convenience sampling	Multicenter	Prospetively	Nigeria	West and Central	Africa	West Africa	Lower-middle income economies	Jan/2017-Apr/2017	Unclear/Not reported	Hospital-based	Unclear/Not reported	On ART	General population	HBV	Immunochromatographic test	HBsAg
Omatola	2020	Cross sectional	Non probabilistic	Consecutive sampling	Monocenter	Prospetively	Nigeria	West and Central	Africa	West Africa	Lower-middle income economies	Unclear/Not reported	Unclear/Not reported	Hospital-based	Unclear/Not reported	On ART	General population	HBV	Immunochromatographic test	HBsAg
Omatola	2017	Cross sectional	Probabilistic	Simple random sampling	Monocenter	Prospetively	Nigeria	West and Central	Africa	West Africa	Lower-middle income economies	Mar/2016-Jun/2016	Unclear/Not reported	Hospital-based	Unclear/Not reported	Unclear/Not reported	General population	HBV	Immunochromatographic test	HBsAg
Omosigho	2011	Cross sectional	Non probabilistic	Consecutive sampling	Monocenter	Prospetively	Nigeria	West and Central	Africa	West Africa	Lower-middle income economies	Jan/2009-Jul/2010	Unclear/Not reported	Hospital-based	Unclear/Not reported	Unclear/Not reported	General population	HCV	Enzyme immunoassay (EIA)	Anti-HCV
Opaleye	2021	Cross sectional	Probabilistic	Simple random sampling	Multicenter	Prospetively	Nigeria	West and Central	Africa	West Africa	Lower-middle income economies	Jun/2017-Aug/2017	All ages	Hospital-based	Unclear/Not reported	ART naïve/ On ART	General population	HBV	Direct ELISA	HBsAg
O'Reilly	2011	Cross sectional	Probabilistic	Simple random sampling	Monocenter	Prospetively	Uganda	East and Southern	Africa	Eastern Africa	Low-income economies	Jan/2008-Feb/2008	Adults	Hospital-based	Unclear/Not reported	Unclear/Not reported	General population	HCV	Rapid Diagnostic test	Anti-HCV
Oronsaye	2004	Cross sectional	Non probabilistic	Consecutive sampling	Monocenter	Prospetively	Nigeria	West and Central	Africa	West Africa	Lower-middle income economies	Jan/2000-Dec/2001	Adults	Hospital-based	Unclear/Not reported	Unclear/Not reported	Blood donors	HBV	Rapid Diagnostic test	HBsAg
Oshitani	1996	Cross sectional	Non probabilistic	Consecutive sampling	Multicenter	Prospetively	Zambia	East and Southern	Africa	Eastern Africa	Lower-middle income economies	Apr/1992-Oct/1992	Unclear/Not reported	Hospital-based	Unclear/Not reported	Unclear/Not reported	Pregnant women	HBV	Reverse passive hemagglutination assay	HBeAg
Oshitani	1996	Cross sectional	Non probabilistic	Consecutive sampling	Multicenter	Prospetively	Zambia	East and Southern	Africa	Eastern Africa	Lower-middle income economies	Apr/1992-Oct/1992	Unclear/Not reported	Hospital-based	Unclear/Not reported	Unclear/Not reported	Pregnant women	HBV	Direct ELISA	HBsAg
Oshitani	1995	Cross sectional	Non probabilistic	Consecutive sampling	Monocenter	Prospetively	Zambia	East and Southern	Africa	Eastern Africa	Lower-middle income economies	Unclear/Not reported	All ages	Hospital-based	Unclear/Not reported	Unclear/Not reported	General population	HCV	Passive haemagglutination test	Anti-HCV
Otegbayo	2008	Cohort (Baseline data)	Non probabilistic	Consecutive sampling	Monocenter	Retrospectively	Nigeria	West and Central	Africa	West Africa	Lower-middle income economies	Aug/2004-Feb/2007	Adults	Hospital-based	Unclear/Not reported	ART naïve	General population	HCV	Indirect ELISA	Anti-HCV
Otegbayo	2008	Cohort (Baseline data)	Non probabilistic	Consecutive sampling	Monocenter	Retrospectively	Nigeria	West and Central	Africa	West Africa	Lower-middle income economies	Aug/2004-Feb/2007	Adults	Hospital-based	Unclear/Not reported	ART naïve	General population	HBV	Direct ELISA	HBsAg
Otuonye	2002	Cross sectional	Non probabilistic	Consecutive sampling	Multicenter	Prospetively	Nigeria	West and Central	Africa	West Africa	Lower-middle income economies	Nov/1997-Dec/1999	Unclear/Not reported	Hospital-based	Unclear/Not reported	Unclear/Not reported	General population	HBV	Unclear/Not reported	HBsAg
Ouattara	1990	Cross sectional	Non probabilistic	Consecutive sampling	Multicenter	Prospetively	Ivory Coast	West and Central	Africa	West Africa	Lower-middle income economies	Jan/1987-Dec/1987	All ages	Hospital-based	Unclear/Not reported	Unclear/Not reported	General population	HBV	Direct ELISA	HBeAg
Ouattara	1990	Cross sectional	Non probabilistic	Consecutive sampling	Multicenter	Prospetively	Ivory Coast	West and Central	Africa	West Africa	Lower-middle income economies	Jan/1987-Dec/1987	All ages	Hospital-based	Unclear/Not reported	Unclear/Not reported	General population	HBV	Direct ELISA	HBeAg
Ouattara	1990	Cross sectional	Non probabilistic	Consecutive sampling	Multicenter	Prospetively	Ivory Coast	West and Central	Africa	West Africa	Lower-middle income economies	Jan/1987-Dec/1987	All ages	Hospital-based	Unclear/Not reported	Unclear/Not reported	General population	HBV	Direct ELISA	HBeAg
Ouattara	1990	Cross sectional	Non probabilistic	Consecutive sampling	Multicenter	Prospetively	Ivory Coast	West and Central	Africa	West Africa	Lower-middle income economies	Jan/1987-Dec/1987	All ages	Hospital-based	Unclear/Not reported	Unclear/Not reported	General population	HBV	Direct ELISA	HBsAg
Ouattara	1990	Cross sectional	Non probabilistic	Consecutive sampling	Multicenter	Prospetively	Ivory Coast	West and Central	Africa	West Africa	Lower-middle income economies	Jan/1987-Dec/1987	All ages	Hospital-based	Unclear/Not reported	Unclear/Not reported	General population	HBV	Direct ELISA	HBsAg
Ouattara	1990	Cross sectional	Non probabilistic	Consecutive sampling	Multicenter	Prospetively	Ivory Coast	West and Central	Africa	West Africa	Lower-middle income economies	Jan/1987-Dec/1987	All ages	Hospital-based	Unclear/Not reported	Unclear/Not reported	General population	HBV	Direct ELISA	HBsAg
Ouermi	2009	Cross sectional	Non probabilistic	Consecutive sampling	Monocenter	Prospetively	Burkina Faso	West and Central	Africa	West Africa	Low-income economies	Jan/2009-Jun/2009	Unclear/Not reported	Hospital-based	Unclear/Not reported	Unclear/Not reported	Pregnant women	HBV	Rapid Diagnostic test	HBsAg
Pappoe	2019	Cross sectional	Non probabilistic	Consecutive sampling	Multicenter	Prospetively	Ghana	West and Central	Africa	West Africa	Lower-middle income economies	May/2015-Aug/2015	All ages	Hospital-based	Unclear/Not reported	ART naïve/ On ART	General population	HCV	Rapid Diagnostic test	Anti-HCV
Pappoe	2019	Cross sectional	Non probabilistic	Consecutive sampling	Multicenter	Prospetively	Ghana	West and Central	Africa	West Africa	Lower-middle income economies	May/2015-Aug/2015	All ages	Hospital-based	Unclear/Not reported	ART naïve/ On ART	General population	HBV	Rapid Diagnostic test	HBsAg
Parboosing	2008	Cross sectional	Non probabilistic	Consecutive sampling	Multicenter	Prospetively	South Africa	East and Southern	Africa	Southern Africa	Upper-middle-income economies	Jan/2004-Jun/2005	All ages	Hospital-based	Unclear/Not reported	ART naïve	General population	HBV	Direct ELISA	HBsAg
Patassi	2016	Cross sectional	Non probabilistic	Consecutive sampling	Multicenter	Prospetively	Togo	West and Central	Africa	West Africa	Low-income economies	Jan/2010-Dec/2011	Adults	Hospital-based	Unclear/Not reported	On ART	General population	HBV	Direct ELISA	HBeAg
Patassi	2016	Cross sectional	Non probabilistic	Consecutive sampling	Multicenter	Prospetively	Togo	West and Central	Africa	West Africa	Low-income economies	Jan/2010-Dec/2011	Adults	Hospital-based	Unclear/Not reported	On ART	General population	HBV	Direct ELISA	HBsAg
Patel	2011	Cross sectional	Non probabilistic	Consecutive sampling	Monocenter	Retrospectively	Botswana	East and Southern	Africa	Southern Africa	Upper-middle-income economies	Jan/2005-Dec/2009	Adults	Hospital-based	Unclear/Not reported	ART naïve/ On ART	General population	HCV	Indirect ELISA	Anti-HCV
Patel	2011	Cross sectional	Non probabilistic	Consecutive sampling	Monocenter	Retrospectively	Botswana	East and Southern	Africa	Southern Africa	Upper-middle-income economies	Jan/2005-Dec/2009	Adults	Hospital-based	Unclear/Not reported	ART naïve/ On ART	General population	HBV	Direct ELISA	HBsAg
Pawlotsky	1995	Cross sectional	Non probabilistic	Consecutive sampling	Monocenter	Prospetively	Central African Repub	West and Central	Africa	Central Africa	Low-income economies	Unclear/Not reported	Adults	Hospital-based	Unclear/Not reported	Unclear/Not reported	General population	HCV	Enzyme immunoassay (EIA)	Anti-HCV
Pawlotsky	1995	Cross sectional	Non probabilistic	Consecutive sampling	Monocenter	Prospetively	Central African Repub	West and Central	Africa	Central Africa	Low-income economies	Unclear/Not reported	Adults	Hospital-based	Unclear/Not reported	Unclear/Not reported	General population	HBV	Enzyme immunoassay (EIA)	HBsAg
Peter	2015	Cross sectional	Non probabilistic	Consecutive sampling	Monocenter	Prospetively	Nigeria	West and Central	Africa	West Africa	Lower-middle income economies	Nov/2012-Apr/2013	Adults	Hospital-based	Unclear/Not reported	ART naïve	General population	HCV	Indirect ELISA	Anti-HCV
Peter	2015	Cross sectional	Non probabilistic	Consecutive sampling	Monocenter	Prospetively	Nigeria	West and Central	Africa	West Africa	Lower-middle income economies	Nov/2012-Apr/2013	Adults	Hospital-based	Unclear/Not reported	ART naïve	General population	HBV	Direct ELISA	HBsAg
Pirillo	2015	Cohort (Baseline data)	Non probabilistic	Consecutive sampling	Monocenter	Prospetively	Malawi	East and Southern	Africa	Eastern Africa	Low-income economies	2008-2011	Unclear/Not reported	Hospital-based	Unclear/Not reported	ART naïve/ On ART	Pregnant women	HBV	Chemiluminescent enzyme immunoassay (CLEIA)	HBeAg
Pirillo	2015	Cohort (Baseline data)	Non probabilistic	Consecutive sampling	Monocenter	Prospetively	Malawi	East and Southern	Africa	Eastern Africa	Low-income economies	2008-2011	Unclear/Not reported	Hospital-based	Unclear/Not reported	ART naïve/ On ART	Pregnant women	HBV	Direct ELISA	HBsAg
Pirillo	2007	Cross sectional	Non probabilistic	Consecutive sampling	Multicenter	Retrospectively	Rwanda	East and Southern	Africa	Eastern Africa	Low-income economies	Jul/2001-Feb/2004	Unclear/Not reported	Hospital-based	Unclear/Not reported	ART naïve/ On ART	Pregnant women	HCV	Indirect ELISA	Anti-HCV
Pirillo	2007	Cross sectional	Non probabilistic	Consecutive sampling	Multicenter	Retrospectively	Rwanda	East and Southern	Africa	Eastern Africa	Low-income economies	Jul/2001-Feb/2004	Unclear/Not reported	Hospital-based	Unclear/Not reported	ART naïve/ On ART	Pregnant women	HBV	Direct ELISA	HBsAg
Pirillo	2007	Cross sectional	Non probabilistic	Consecutive sampling	Multicenter	Retrospectively	Uganda	East and Southern	Africa	Eastern Africa	Low-income economies	Jul/2001-Feb/2004	Unclear/Not reported	Hospital-based	Unclear/Not reported	ART naïve/ On ART	Pregnant women	HBV	Direct ELISA	HBsAg
Pirillo	2007	Cross sectional	Non probabilistic	Consecutive sampling	Multicenter	Retrospectively	Uganda	East and Southern	Africa	Eastern Africa	Low-income economies	Jul/2001-Feb/2004	Unclear/Not reported	Hospital-based	Unclear/Not reported	ART naïve/ On ART	Pregnant women	HCV	Indirect ELISA	Anti-HCV
Plamondon	2007	Cross sectional	Non probabilistic	Consecutive sampling	Multicenter	Prospetively	Guinea-Bissau	West and Central	Africa	West Africa	Low-income economies	Jan/2005-Mar/2005	Adults	Hospital-based	Unclear/Not reported	Unclear/Not reported	General population	HCV	Indirect ELISA	Anti-HCV
Powell	2015	Cross sectional	Non probabilistic	Consecutive sampling	Monocenter	Prospetively	South Africa	East and Southern	Africa	Southern Africa	Upper-middle-income economies	Sep/2004-Dec/2009	Unclear/Not reported	Hospital-based	Unclear/Not reported	ART naïve/ On ART	General population	HBV	Electro-chemiluminescence immunoassay (ECLIA)	HBsAg
Price	2017	Clinical Trial (Baseline data)	Probabilistic	Simple random sampling	Multicenter	Retrospectively	Uganda	East and Southern	Africa	Eastern Africa	Low-income economies	Jan/2003-Oct/2004	Adults	Hospital-based	Unclear/Not reported	Unclear/Not reported	General population	HBV	Enzyme immunoassay (EIA)	HBsAg

Price	2017	Clinical Trial (Baseline data)	Probabilistic	Simple random sampling	Multicenter	Retrospectively	Zimbabwe	East and Southern	Africa	Eastern Africa	Lower-middle income economies	Jan/2003-Oct/2004	Adults	Hospital-based	Unclear/Not reported	Unclear/Not reported	General population	HBV	Enzyme immunoassay (EIA)	HBsAg
Rabenau	2010	Cross sectional	Non probabilistic	Consecutive sampling	Monocenter	Prospetively	Lesotho	East and Southern	Africa	Southern Africa	Lower-middle income economies	Jan/2007-Mar/2007	Adults	Hospital-based	Unclear/Not reported	On ART	General population	HCV	Indirect ELISA	Anti-HCV
Rabenau	2010	Cross sectional	Non probabilistic	Consecutive sampling	Monocenter	Prospetively	Lesotho	East and Southern	Africa	Southern Africa	Lower-middle income economies	Jan/2007-Mar/2007	Adults	Hospital-based	Unclear/Not reported	On ART	General population	HBV	Direct ELISA	HBsAg
Rahlenbeck	1997	Cross sectional	Non probabilistic	Consecutive sampling	Monocenter	Prospetively	Ethiopia	East and Southern	Africa	Eastern Africa	Low-income economies	Nov/1994-Mar/1995	Adults	Hospital-based	Unclear/Not reported	Unclear/Not reported	Blood donors	HBV	Rapid Diagnostic test	HBsAg
Ramírez Mena	2022	Cross sectional	Non probabilistic	Consecutive sampling	Monocenter	Retrospectively	Senegal	West and Central	Africa	West Africa	Lower-middle income economies	Jan/2019-Mar/2019	Adults	Hospital-based	Urban	Unclear/Not reported	General population	HBV	Rapid Diagnostic test	HBsAg
Ramírez Mena	2022	Cross sectional	Non probabilistic	Consecutive sampling	Monocenter	Prospetively	Senegal	West and Central	Africa	West Africa	Lower-middle income economies	Jan/2019-Mar/2019	Adults	Hospital-based	Urban	Unclear/Not reported	General population	HBV	Rapid Diagnostic test	HBsAg
Rebbani	2013	Cohort (Baseline data)	Non probabilistic	Consecutive sampling	Monocenter	Prospetively	Morocco	North Africa and Middle East	Eastern Mediterranean	Northern Africa	Lower-middle income economies	Jan/2006-Jun/2010	Unclear/Not reported	Hospital-based	Unclear/Not reported	ART naïve/ On ART	General population	HBV	Direct ELISA	HBeAg
Rebbani	2013	Cohort (Baseline data)	Non probabilistic	Consecutive sampling	Monocenter	Prospetively	Morocco	North Africa and Middle East	Eastern Mediterranean	Northern Africa	Lower-middle income economies	Jan/2006-Jun/2010	Unclear/Not reported	Hospital-based	Unclear/Not reported	ART naïve/ On ART	General population	HCV	Indirect ELISA	Anti-HCV
Rebbani	2013	Cohort (Baseline data)	Non probabilistic	Consecutive sampling	Monocenter	Prospetively	Morocco	North Africa and Middle East	Eastern Mediterranean	Northern Africa	Lower-middle income economies	Jan/2006-Jun/2010	Unclear/Not reported	Hospital-based	Unclear/Not reported	ART naïve/ On ART	General population	HBV	Direct ELISA	HBsAg
Rouet	2015	Cross sectional	Non probabilistic	Consecutive sampling	Monocenter	Retrospectively	Gabon	West and Central	Africa	Central Africa	Upper-middle-income economies	Mar/2010-Jan/2013	Unclear/Not reported	Hospital-based	Unclear/Not reported	ART naïve/ On ART	General population	HCV	Indirect ELISA	Anti-HCV
Rouet	2008	Cohort (Baseline data)	Non probabilistic	Consecutive sampling	Monocenter	Retrospectively	Ivory Coast	West and Central	Africa	West Africa	Lower-middle income economies	Oct/2000-Dec/2003	Children	Hospital-based	Unclear/Not reported	ART naïve/ On ART	General population	CFR	Direct ELISA	HBsAg
Rouet	2008	Cohort (Baseline data)	Non probabilistic	Consecutive sampling	Monocenter	Retrospectively	Ivory Coast	West and Central	Africa	West Africa	Lower-middle income economies	Oct/2000-Dec/2003	Children	Hospital-based	Unclear/Not reported	ART naïve/ On ART	General population	HCV	Direct ELISA	Anti-HCV
Rouet	2008	Cohort (Baseline data)	Non probabilistic	Consecutive sampling	Monocenter	Retrospectively	Ivory Coast	West and Central	Africa	West Africa	Lower-middle income economies	Oct/2000-Dec/2003	Children	Hospital-based	Unclear/Not reported	ART naïve/ On ART	General population	HBV	Direct ELISA	HBeAg
Rouet	2008	Cohort (Baseline data)	Non probabilistic	Consecutive sampling	Monocenter	Retrospectively	Ivory Coast	West and Central	Africa	West Africa	Lower-middle income economies	Oct/2000-Dec/2003	Children	Hospital-based	Unclear/Not reported	ART naïve/ On ART	General population	HBV	Direct ELISA	HBsAg
Rouet	2004	Cross sectional	Non probabilistic	Consecutive sampling	Multicenter	Retrospectively	Ivory Coast	West and Central	Africa	West Africa	Lower-middle income economies	Sep/1995-Nov/1998	Unclear/Not reported	Hospital-based	Unclear/Not reported	ART naïve/ On ART	Pregnant women	HBV	Direct ELISA	HBsAg
Rouet	2004	Cross sectional	Non probabilistic	Consecutive sampling	Multicenter	Retrospectively	Ivory Coast	West and Central	Africa	West Africa	Lower-middle income economies	Sep/1995-Nov/1998	Unclear/Not reported	Hospital-based	Unclear/Not reported	ART naïve/ On ART	Pregnant women	HCV	Indirect ELISA	Anti-HCV
Rusine	2013	Cohort (Baseline data)	Non probabilistic	Consecutive sampling	Monocenter	Prospetively	Rwanda	East and Southern	Africa	Eastern Africa	Low-income economies	Nov/2007-Jan/2010	Unclear/Not reported	Hospital-based	Unclear/Not reported	ART naïve	General population	HCV	Indirect ELISA	Anti-HCV
Rusine	2013	Cohort (Baseline data)	Non probabilistic	Consecutive sampling	Monocenter	Prospetively	Rwanda	East and Southern	Africa	Eastern Africa	Low-income economies	Nov/2007-Jan/2010	Unclear/Not reported	Hospital-based	Unclear/Not reported	ART naïve	General population	HBV	Direct ELISA	HBsAg
Sadoh	2011	Cross sectional	Non probabilistic	Consecutive sampling	Monocenter	Prospetively	Nigeria	West and Central	Africa	West Africa	Lower-middle income economies	Oct/2009-Dec/2009	Children	Hospital-based	Unclear/Not reported	On ART	General population	HCV	Indirect ELISA	Anti-HCV
Sadoh	2011	Cross sectional	Non probabilistic	Consecutive sampling	Monocenter	Prospetively	Nigeria	West and Central	Africa	West Africa	Lower-middle income economies	Oct/2009-Dec/2009	Children	Hospital-based	Unclear/Not reported	On ART	General population	HBV	Direct ELISA	HBsAg
Sagoe	2012	Cross sectional	Non probabilistic	Consecutive sampling	Monocenter	Prospetively	Ghana	West and Central	Africa	West Africa	Lower-middle income economies	Jun/2007-Nov/2007	Unclear/Not reported	Hospital-based	Unclear/Not reported	ART naïve	General population	HBV	Direct ELISA	HBeAg
Sagoe	2012	Cross sectional	Non probabilistic	Consecutive sampling	Monocenter	Prospetively	Ghana	West and Central	Africa	West Africa	Lower-middle income economies	Jun/2007-Nov/2007	Unclear/Not reported	Hospital-based	Unclear/Not reported	ART naïve	General population	HCV	Indirect ELISA	Anti-HCV
Sagoe	2012	Cross sectional	Non probabilistic	Consecutive sampling	Monocenter	Prospetively	Ghana	West and Central	Africa	West Africa	Lower-middle income economies	Jun/2007-Nov/2007	Unclear/Not reported	Hospital-based	Unclear/Not reported	ART naïve	General population	HBV	Direct ELISA	HBsAg
Salpini	2016	Cross sectional	Non probabilistic	Consecutive sampling	Monocenter	Prospetively	Cameroon	West and Central	Africa	Central Africa	Lower-middle income economies	Apr/2010-Aug/2010	Unclear/Not reported	Hospital-based	Unclear/Not reported	ART naïve/ On ART	General population	HCV	Indirect ELISA	Anti-HCV
Salpini	2016	Cross sectional	Non probabilistic	Consecutive sampling	Monocenter	Prospetively	Cameroon	West and Central	Africa	Central Africa	Lower-middle income economies	Apr/2010-Aug/2010	Unclear/Not reported	Hospital-based	Unclear/Not reported	ART naïve/ On ART	General population	HBV	Direct ELISA	HBsAg
Salu	2018	Cross sectional	Non probabilistic	Consecutive sampling	Multicenter	Prospetively	Nigeria	West and Central	Africa	West Africa	Lower-middle income economies	Dec/2008-Jan/2014	Children	Hospital-based	Unclear/Not reported	ART naïve/ On ART	General population	HCV	Indirect ELISA	Anti-HCV
Salu	2018	Cross sectional	Non probabilistic	Consecutive sampling	Multicenter	Prospetively	Nigeria	West and Central	Africa	West Africa	Lower-middle income economies	Dec/2008-Jan/2014	Children	Hospital-based	Unclear/Not reported	ART naïve/ On ART	General population	HBV	Direct ELISA	HBsAg
Salyani	2021	Cross sectional	Non probabilistic	Consecutive sampling	Multicenter	Prospetively	Kenya	East and Southern	Africa	Eastern Africa	Lower-middle income economies	Mar/2019-Apr/2020	Unclear/Not reported	Hospital-based	Unclear/Not reported	ART naïve	General population	HBV	Electro-chemiluminescence immunoassay (ECLIA)	HBsAg
Seremba	2010	Cross sectional	Non probabilistic	Consecutive sampling	Monocenter	Prospetively	Uganda	East and Southern	Africa	Eastern Africa	Low-income economies	Jan/2006	All ages	Hospital-based	Unclear/Not reported	Unclear/Not reported	General population	HCV	Rapid Diagnostic test	Anti-HCV
Shimelis	2017	Cross sectional	Probabilistic	Simple random sampling	Monocenter	Retrospectively	Ethiopia	East and Southern	Africa	Eastern Africa	Low-income economies	Jan/2014-May/2014	Adults	Hospital-based	Urban/Rural	On ART	General population	HCV	Rapid Diagnostic test	Anti-HCV
Shimelis	2017	Cross sectional	Probabilistic	Simple random sampling	Monocenter	Retrospectively	Ethiopia	East and Southern	Africa	Eastern Africa	Low-income economies	Jan/2014-May/2014	Adults	Hospital-based	Urban/Rural	On ART	General population	HBV and HCV	Rapid Diagnostic test	Anti-HCV, HBsAg
Shimelis	2017	Cross sectional	Probabilistic	Simple random sampling	Monocenter	Retrospectively	Ethiopia	East and Southern	Africa	Eastern Africa	Low-income economies	Jan/2014-May/2014	Adults	Hospital-based	Urban/Rural	On ART	General population	HBV	Rapid Diagnostic test	HBeAg
Shimelis	2017	Cross sectional	Probabilistic	Simple random sampling	Monocenter	Retrospectively	Ethiopia	East and Southern	Africa	Eastern Africa	Low-income economies	Jan/2014-May/2014	Adults	Hospital-based	Urban/Rural	On ART	General population	HBV	Rapid Diagnostic test	HBsAg
Shimelis	2008	Cross sectional	Non probabilistic	Consecutive sampling	Monocenter	Prospetively	Ethiopia	East and Southern	Africa	Eastern Africa	Low-income economies	Jan/2007-Feb/2007	All ages	Hospital-based	Unclear/Not reported	Unclear/Not reported	General population	HBV	Direct ELISA	HBsAg
Simani	2009	Cross sectional	Non probabilistic	Consecutive sampling	Multicenter	Prospetively	South Africa	East and Southern	Africa	Southern Africa	Upper-middle-income economies	Jan/2003-Dec/2004	Children	Hospital-based	Unclear/Not reported	ART naïve/ On ART	General population	HBV	Auszyme Assay	HBsAg
Simpore	2006	Cross sectional	Non probabilistic	Consecutive sampling	Monocenter	Prospetively	Burkina Faso	West and Central	Africa	West Africa	Low-income economies	May/2004-Aug/2005	Adults	Hospital-based	Unclear/Not reported	ART naïve/ On ART	Pregnant women	HCV	Inter Second Antibody Immunoassay (ISAI)	Anti-HCV
Simpore	2006	Cross sectional	Non probabilistic	Consecutive sampling	Monocenter	Prospetively	Burkina Faso	West and Central	Africa	West Africa	Low-income economies	May/2004-Aug/2005	Adults	Hospital-based	Unclear/Not reported	ART naïve/ On ART	Pregnant women	HBV and HCV	Inter Second Antibody Immunoassay (ISAI)	Anti-HCV, HBsAg
Simpore	2006	Cross sectional	Non probabilistic	Consecutive sampling	Monocenter	Prospetively	Burkina Faso	West and Central	Africa	West Africa	Low-income economies	May/2004-Aug/2005	Adults	Hospital-based	Unclear/Not reported	ART naïve/ On ART	Pregnant women	HBV	Inter Second Antibody Immunoassay (ISAI)	HBsAg
Simpore	2004	Cross sectional	Non probabilistic	Consecutive sampling	Multicenter	Prospetively	Burkina Faso	West and Central	Africa	West Africa	Low-income economies	Unclear/Not reported	Adults	Hospital-based	Unclear/Not reported	Unclear/Not reported	General population	HBV	Immunoassay kit	HBsAg
Simpore	2004	Cross sectional	Non probabilistic	Consecutive sampling	Multicenter	Prospetively	Burkina Faso	West and Central	Africa	West Africa	Low-income economies	Unclear/Not reported	Adults	Hospital-based	Unclear/Not reported	Unclear/Not reported	Pregnant women	HBV	Immunoassay kit	HBsAg
Simpore	2005	Cross sectional	Probabilistic	Simple random sampling	Monocenter	Prospetively	Burkina Faso	West and Central	Africa	West Africa	Low-income economies	Dec/2001-Jul/2002	Adults	Hospital-based	Unclear/Not reported	Unclear/Not reported	Pregnant women	HCV	Classical RT-PCR	Viral RNA
Simpore	2005	Cross sectional	Probabilistic	Simple random sampling	Monocenter	Prospetively	Burkina Faso	West and Central	Africa	West Africa	Low-income economies	Dec/2001-Jul/2002	Adults	Hospital-based	Unclear/Not reported	Unclear/Not reported	Pregnant women	HCV	Enzyme immunoassay (EIA)	Anti-HCV
Simpore	2006	Cross sectional	Non probabilistic	Consecutive sampling	Monocenter	Prospetively	Burkina Faso	West and Central	Africa	West Africa	Low-income economies	Apr/2003-May/2005	Adults	Hospital-based	Unclear/Not reported	Unclear/Not reported	Pregnant women	HCV	Immunoassay kit	Anti-HCV
Simpore	2006	Cross sectional	Non probabilistic	Consecutive sampling	Monocenter	Prospetively	Burkina Faso	West and Central	Africa	West Africa	Low-income economies	Apr/2003-May/2005	Adults	Hospital-based	Unclear/Not reported	Unclear/Not reported	Pregnant women	HBV	Immunoassay kit	HBsAg
Sonderup	2015	Cross sectional	Non probabilistic	Consecutive sampling	Multicenter	Retroprospectively	South Africa	East and Southern	Africa	Southern Africa	Upper-middle-income economies	Jan/2000-Jul/2013	Unclear/Not reported	Hospital-based	Unclear/Not reported	ART naïve/ On ART	General population	HCV	Enzyme immunoassay (EIA)	Anti-HCV
Sonderup	2015	Cross sectional	Non probabilistic	Consecutive sampling	Multicenter	Retroprospectively	South Africa	East and Southern	Africa	Southern Africa	Upper-middle-income economies	Jan/2000-Jul/2013	Unclear/Not reported	Hospital-based	Unclear/Not reported	ART naïve/ On ART	General population	HBV	Enzyme immunoassay (EIA)	HBsAg
Sonderup	2015	Cross sectional	Non probabilistic	Consecutive sampling	Multicenter	Retroprospectively	South Africa	East and Southern	Africa	Southern Africa	Upper-middle-income economies	Jan/2000-Jul/2013	Unclear/Not reported	Hospital-based	Unclear/Not reported	ART naïve/ On ART	General population	HCV	Classical PCR	Viral RNA
Soni	1993	Cross sectional	Non probabilistic	Consecutive sampling	Multicenter	Prospetively	South Africa	East and Southern	Africa	Southern Africa	Upper-middle-income economies	Sep/1991-Nov/1991	Adults	Hospital-based	Unclear/Not reported	Unclear/Not reported	Men who have sex with men	HCV	Enzyme immunoassay (EIA)	Anti-HCV
Stabinski	2011	Cross sectional	Non probabilistic	Consecutive sampling	Multicenter	Prospetively	Uganda	East and Southern	Africa	Eastern Africa	Low-income economies	Unclear/Not reported	Unclear/Not reported	Hospital-based	Rural	ART naïve/ On ART	General population	HBV	Direct ELISA	HBsAg
Stokx	2011	Cross sectional	Non probabilistic	Consecutive sampling	Monocenter	Prospetively	Mozambique	East and Southern	Africa	Eastern Africa	Low-income economies	Feb/2009-May/2009	Adults	Hospital-based	Unclear/Not reported	Unclear/Not reported	Blood donors	HBV	Rapid Diagnostic test	HBsAg
Sutcliffe	2002	Case control	Non probabilistic	Consecutive sampling	Monocenter	Prospetively	Malawi	East and Southern	Africa	Eastern Africa	Low-income economies	1998	Adults	Hospital-based	Unclear/Not reported	Unclear/Not reported	General population	HBV	Enzyme immunoassay (EIA)	HBsAg
Sutcliffe	2002	Case control	Non probabilistic	Consecutive sampling	Monocenter	Prospetively	Malawi	East and Southern	Africa	Eastern Africa	Low-income economies	1998	Adults	Hospital-based	Unclear/Not reported	Unclear/Not reported	General population	HCV	Indirect ELISA	Anti-HCV
Tamo	2017	Cross sectional	Non probabilistic	Consecutive sampling	Monocenter	Prospetively	Cameroon	West and Central	Africa	Central Africa	Lower-middle income economies	Nov/2015-Feb/2016	Adults	Hospital-based	Unclear/Not reported	Unclear/Not reported	General population	HCV	Unclear/Not reported	Anti-HCV
Taye	2013	Cohort (Baseline data)	Non probabilistic	Convenience sampling	Monocenter	Prospetively	Ethiopia	East and Southern	Africa	Eastern Africa	Low-income economies	Sep/2006	Unclear/Not reported	Hospital-based	Unclear/Not reported	Unclear/Not reported	General population	HCV	Rapid Diagnostic test	Anti-HCV
Telatela	2007	Cross sectional	Non probabilistic	Consecutive sampling	Monocenter	Prospetively	Tanzania	East and Southern	Africa	Eastern Africa	Lower-middle income economies	Apr/2006-Aug/2006	Children	Hospital-based	Unclear/Not reported	ART naïve/ On ART	General population	HCV	Indirect ELISA	Anti-HCV
Telatela	2007	Cross sectional	Non probabilistic	Consecutive sampling	Monocenter	Prospetively	Tanzania	East and Southern	Africa	Eastern Africa	Lower-middle income economies	Apr/2006-Aug/2006	Children	Hospital-based	Unclear/Not reported	ART naïve/ On ART	General population	HBV	Direct ELISA	HBsAg
Tess	2000	Cross sectional	Probabilistic	Simple random sampling	Monocenter	Prospetively	Tanzania	East and Southern	Africa	Eastern Africa	Lower-middle income economies	1989-1990	Adults	Hospital-based	Unclear/Not reported	Unclear/Not reported	General population	HCV	Enzyme immunoassay (EIA)	Anti-HCV
Tessema	2010	Cross sectional	Non probabilistic	Consecutive sampling	Monocenter	Retrospectively	Ethiopia	East and Southern	Africa	Eastern Africa	Low-income economies	Jan/2003-Dec/2007	Adults	Hospital-based	Unclear/Not reported	Unclear/Not reported	Blood donors	HCV	Indirect ELISA	Anti-HCV
Tessema	2010	Cross sectional	Non probabilistic	Consecutive sampling	Monocenter	Retrospectively	Ethiopia	East and Southern	Africa	Eastern Africa	Low-income economies	Jan/2003-Dec/2007	Adults	Hospital-based	Unclear/Not reported	Unclear/Not reported	Blood donors	HBV	Direct ELISA	HBsAg
Torpey	2021	Cross sectional	Non probabilistic	Consecutive sampling	Monocenter	Prospetively	Ghana	West and Central	Africa	West Africa	Lower-middle income economies	Nov/2018-Aug/2019	Adults	Hospital-based	Unclear/Not reported	On ART	General population	HCV	Rapid Diagnostic test	Anti-HCV
Torpey	2021	Cross sectional	Non probabilistic	Consecutive sampling	Monocenter	Prospetively	Ghana	West and Central	Africa	West Africa	Lower-middle income economies	Nov/2018-Aug/2019	Adults	Hospital-based	Unclear/Not reported	On ART	General population	HCV	Classical RT-PCR	Viral RNA
Tounkara	2009	Cross sectional	Non probabilistic	Consecutive sampling	Monocenter	Prospetively	Mali	West and Central	Africa	West Africa	Low-income economies	Nov/2001-Jul/2002	Adults	Hospital-based	Unclear/Not reported	Unclear/Not reported	Blood donors	HBV	Direct ELISA	HBsAg
Toyé	2020	Cross sectional	Non probabilistic	Consecutive sampling	Multicenter	Retrospectively	Senegal	West and Central	Africa	West Africa	Lower-middle income economies	Mar/2015-Jun/2015	Unclear/Not reported	Hospital-based	Unclear/Not reported	On ART	General population	HBV	Rapid Diagnostic test	HBsAg
Tremeau-Bravard	2012	Cross sectional	Non probabilistic	Consecutive sampling	Monocenter	Retrospectively	Nigeria	West and Central	Africa	West Africa	Lower-middle income economies	Sep/2010-May/2011	Unclear/Not reported	Hospital-based	Unclear/Not reported	On ART	General population	HCV	Enzyme immunoassay (EIA)	Anti-HCV
Tremeau-Bravard	2012	Cross sectional	Non probabilistic	Consecutive sampling	Monocenter	Retrospectively	Nigeria	West and Central	Africa	West Africa	Lower-middle income economies	Sep/2010-May/2011	Unclear/Not reported	Hospital-based	Unclear/Not reported	On ART	General population	HBV and HCV	Enzyme immunoassay (EIA)	Anti-HCV, HBsAg
Tremeau-Bravard	2012	Cross sectional	Non probabilistic	Consecutive sampling	Monocenter	Retrospectively	Nigeria	West and Central	Africa	West Africa	Lower-middle income economies	Sep/2010-May/2011	Unclear/Not reported	Hospital-based	Unclear/Not reported	On ART	General population	HBV	Enzyme immunoassay (EIA)	HBsAg
Umutesi	2017	Cross sectional	Non probabilistic	Consecutive sampling	Nationally representative	Prospetively	Rwanda	East and Southern	Africa	Eastern Africa	Low-income economies	Jan/2016-Jun/2016	All ages	Hospital-based	Urban/Rural	ART naïve/ On ART	General population	HBV and HCV	Direct ELISA, Indirect ELISA	Anti-HCV, HBsAg
Umutesi	2017	Cross sectional	Non probabilistic	Consecutive sampling	Nationally representative	Prospetively	Rwanda	East and Southern	Africa	Eastern Africa	Low-income economies	Jan/2016-Jun/2016	All ages	Hospital-based	Urban/Rural	ART naïve/ On ART	General population	HBV	Direct ELISA	HBsAg
Umutesi	2017	Cross sectional	Non probabilistic	Consecutive sampling	Nationally representative	Prospetively	Rwanda	East and Southern	Africa	Eastern Africa	Low-income economies	Jan/2016-Jun/2016	All ages	Hospital-based	Urban/Rural	ART naïve/ On ART	General population	HCV	Indirect ELISA	Anti-HCV
Uneke	2005	Cross sectional	Non probabilistic	Consecutive sampling	Monocenter	Prospetively	Nigeria	West and Central	Africa	West Africa	Lower-middle income economies	Dec/1999-May/2002	Adults	Hospital-based	Unclear/Not reported	ART naïve/ On ART	Blood donors	HBV	Direct ELISA	HBsAg
Utoo	2012	Cross sectional	Non probabilistic	Consecutive sampling	Monocenter	Prospetively	Nigeria	West and Central	Africa	West Africa	Lower-middle income economies	Jan/2010-Jun/2010	Adults	Hospital-based	Unclear/Not reported	ART naïve/ On ART	General population	HBV	Direct ELISA	HBsAg
Varo	2016	Cross sectional	Probabilistic	Simple random sampling	Monocenter	Prospetively	Malawi	East and Southern	Africa	Eastern Africa	Low-income economies	May/2008-Dec/2010	Children	Hospital-based	Unclear/Not reported	On ART	General population	HBV	Enzyme immunoassay (EIA)	HBsAg
Velen	2016	Cohort (Baseline data)	Non probabilistic	Consecutive sampling	Monocenter	Retrospectively	South Africa	East and Southern	Africa	Southern Africa	Upper-middle-income economies	2002-2010	Unclear/Not reported	Hospital-based	Unclear/Not reported	On ART	General population	HBV	ADVIA Centaur chemiluminometric immunoassay system	HBsAg
Waddell	2006	Cross sectional	Non probabilistic	Consecutive sampling	Monocenter	Prospetively	Tanzania	East and Southern	Africa	Eastern Africa	Lower-middle income economies	Unclear/Not reported	Adults	Hospital-based	Unclear/Not reported	Unclear/Not reported	Blood donors	HCV	VERSANT HCV RNA Qualitative Assay	Viral RNA
Walusansa	2009	Cross sectional	Non probabilistic	Consecutive sampling	Monocenter	Prospetively	Uganda	East and Southern	Africa	Eastern Africa	Low-income economies	Oct/2003-Feb/2004	Adults	Hospital-based	Unclear/Not reported	ART naïve/ On ART	General population	HCV	Rapid Diagnostic test	Anti-HCV
Wandeler	2016	Cross sectional	Non probabilistic	Consecutive sampling	Multicenter	Prospetively	Mozambique	East and Southern	Africa	Eastern Africa	Low-income economies	May/2013-Nov/2014	Adults	Hospital-based	Unclear/Not reported	On ART	General population	HCV	Rapid Diagnostic test	Anti-HCV
Wandeler	2016	Cross sectional	Non probabilistic	Consecutive sampling	Multicenter	Prospetively	Mozambique	East and Southern	Africa	Eastern Africa	Low-income economies	May/2013-Aug/2014	Adults	Hospital-based	Unclear/Not reported	On ART	General population	HBV	Rapid Diagnostic test	HBsAg
Wandeler	2016	Cross sectional	Non probabilistic	Consecutive sampling	Multicenter	Prospetively	Zambia	East and Southern	Africa	Eastern Africa	Lower-middle income economies	May/2013-Nov/2014	Adults	Hospital-based	Unclear/Not reported	On ART	General population	HCV	Rapid Diagnostic test	Anti-HCV
Wandeler	2016	Cross sectional	Non probabilistic	Consecutive sampling	Multicenter	Prospetively	Zambia	East and Southern	Africa	Eastern Africa	Lower-middle income economies	Unclear/Not reported	Adults	Hospital-based	Unclear/Not reported	On ART	General population	HBV	Rapid Diagnostic test	HBsAg
Wandeler	2016	Cross sectional	Non probabilistic	Consecutive sampling	Multicenter	Prospetively	Zambia	East and Southern	Africa	Eastern Africa	Lower-middle income economies	May/2013-Aug/2014	Adults	Hospital-based	Unclear/Not reported	On ART	General population	HBV	Rapid Diagnostic test	HBsAg
Webale	2015	Cross sectional	Non probabilistic	Consecutive sampling	Monocenter	Prospetively	Kenya	East and Southern	Africa	Eastern Africa	Lower-middle income economies	Unclear/Not reported	Unclear/Not reported	Hospital-based	Unclear/Not reported	ART naïve/ On ART	General population	HBV	Rapid Diagnostic test	HBeAg
Webale	2015	Cross sectional	Non probabilistic	Consecutive sampling	Monocenter	Prospetively	Kenya	East and Southern	Africa	Eastern Africa	Lower-middle income economies	Unclear/Not reported	Unclear/Not reported	Hospital-based	Unclear/Not reported	ART naïve/ On ART	Injecting drug users	HBV	Rapid Diagnostic test	HBeAg
Webale	2015	Cross sectional	Non probabilistic	Consecutive sampling	Monocenter	Prospetively	Kenya	East and Southern	Africa	Eastern Africa	Lower-middle income economies	Unclear/Not reported	Unclear/Not reported	Hospital-based	Unclear/Not reported	ART naïve/ On ART	General population	HBV	Rapid Diagnostic test	HBsAg
Webale	2015	Cross sectional	Non probabilistic	Consecutive sampling	Monocenter	Prospetively	Kenya	East and Southern	Africa	Eastern Africa	Lower-middle income economies	Unclear/Not reported	Unclear/Not reported	Hospital-based	Unclear/Not reported	ART naïve/ On ART	Injecting drug users	HBV	Rapid Diagnostic test	HBsAg
Wekesa	2020	Cross sectional	Non probabilistic	Consecutive sampling	Multicenter	Prospetively	Uganda	East and Southern	Africa	Eastern Africa	Low-income economies	Jan/2015-Dec/2017	Adults	Hospital-based	Urban/Rural	ART naïve/ On ART	General population	HBV	Enzyme immunoassay (EIA)	HBsAg
Wekesa	2020	Cross sectional	Non probabilistic	Consecutive sampling	Multicenter	Prospetively	Uganda	East and Southern	Africa	Eastern Africa	Low-income economies	Jan/2015-Dec/2017	Adults	Hospital-based	Rural	ART naïve/ On ART	General population	HCV	Indirect ELISA	Anti-HCV
Weldemhret	2016	Cross sectional	Non probabilistic	Consecutive sampling	Monocenter	Prospetively	Ethiopia	East and Southern	Africa	Eastern Africa	Low-income economies	Aug/2014-Oct/2014	Unclear/Not reported	Hospital-based	Urban/Rural	On ART	General population	HBV	Direct ELISA	HBsAg
Wester	2006	Cross sectional	Non probabilistic	Consecutive sampling	Monocenter	Prospetively	Botswana	East and Southern	Africa	Southern Africa	Upper-middle-income economies	May/2001-Jan/2002	Adults	Hospital-based	Unclear/Not reported	ART naïve	General population	HCV	Enzyme immunoassay (EIA)	Anti-HCV
Wester	2006	Cross sectional	Non probabilistic	Consecutive sampling	Monocenter	Prospetively	Botswana	East and Southern	Africa	Southern Africa	Upper-middle-income economies	May/2001-Jan/2002	Adults	Hospital-based	Unclear/Not reported	ART naïve	General population	HBV	Enzyme immunoassay (EIA)	HBeAg
Wester	2006	Cross sectional	Non probabilistic	Consecutive sampling	Monocenter	Prospetively	Botswana	East and Southern	Africa	Southern Africa	Upper-middle-income economies	May/2001-Jan/2002	Adults	Hospital-based	Unclear/Not reported	ART naïve	General population	HBV	Enzyme immunoassay (EIA)	HBsAg
Wondimeneh	2013	Cross sectional	Non probabilistic	Consecutive sampling	Monocenter	Prospetively	Ethiopia	East and Southern	Africa	Eastern Africa	Low-income economies	Mar/2011-May/2011	Adults	Hospital-based	Urban/Rural	ART naïve	General population	HCV	Immunochromatographic test	Anti-HCV
Wondimeneh	2013	Cross sectional	Non probabilistic	Consecutive sampling	Monocenter	Prospetively	Ethiopia	East and Southern	Africa	Eastern Africa	Low-income economies	Mar/2011-May/2011	Adults	Hospital-based	Urban/Rural	ART naïve	General population	HBV and HCV	Immunochromatographic test	Anti-HCV, HBsAg
Wondimeneh	2013	Cross sectional	Non probabilistic	Consecutive sampling	Monocenter	Prospetively	Ethiopia	East and Southern	Africa	Eastern Africa	Low-income economies	Mar/2011-May/2011	Adults	Hospital-based	Urban/Rural	ART naïve	General population	HBV	Immunochromatographic test	HBsAg
Xie	2015	Cross sectional	Non probabilistic	Consecutive sampling	Monocenter	Retrospectively	Equatorial Guinea	West and Central	Africa	Central Africa	Upper-middle-income economies	Jan/2011-Apr/2013	Adults	Hospital-based	Unclear/Not reported	Unclear/Not reported	Blood donors	HCV	Rapid Diagnostic test	Anti-HCV
Xie	2015	Cross sectional	Non probabilistic	Consecutive sampling	Monocenter	Retrospectively	Equatorial Guinea	West and Central	Africa	Central Africa	Upper-middle-income economies	Jan/2011-Apr/2013	Adults	Hospital-based	Unclear/Not reported	Unclear/Not reported	Blood donors	HBV	Rapid Diagnostic test	HBsAg
Ya’aba	2015	Cross sectional	Probabilistic	Simple random sampling	Multicenter	Prospetively	Nigeria	West and Central	Africa	West Africa	Lower-middle income economies	Aug/2011-Oct/2011	Unclear/Not reported	Hospital-based	Unclear/Not reported	ART naïve/ On ART	General population	HCV	Rapid Diagnostic test	Anti-HCV
Ya’aba	2017	Cross sectional	Non probabilistic	Consecutive sampling	Monocenter	Prospetively	Nigeria	West and Central	Africa	West Africa	Lower-middle income economies	May/2013-Nov/2013	Adults	Hospital-based	Unclear/Not reported	On ART	General population	HCV	Indirect ELISA	Anti-HCV
Ya'Aba	2009	Cross sectional	Non probabilistic	Consecutive sampling	Monocenter	Prospetively	Nigeria	West and Central	Africa	West Africa	Lower-middle income economies	Jan/2005-May/2006	Adults	Hospital-based	Unclear/Not reported	Unclear/Not reported	Pregnant women	HCV	Rapid Diagnostic test	Anti-HCV
Yakubu	2021	Cross sectional	Non probabilistic	Consecutive sampling	Monocenter	Prospetively	Nigeria	West and Central	Africa	West Africa	Lower-middle income economies	Mar/2014-Oct/2015	Adults	Hospital-based	Unclear/Not reported	ART naïve	General population	HCV	Indirect ELISA	Anti-HCV
Yendewa	2021	Cross sectional	Non probabilistic	Convenience sampling	Multicenter	Prospetively	Sierra Leone	West and Central	Africa	West Africa	Low-income economies	Feb/2019-May/2019	Adults	Hospital-based	Unclear/Not reported	ART naïve/ On ART	Pregnant women	HCV	Chemiluminescent enzyme immunoassay (CLEIA)	Anti-HCV
Yendewa	2021	Cross sectional	Non probabilistic	Convenience sampling	Multicenter	Prospetively	Sierra Leone	West and Central	Africa	West Africa	Low-income economies	Feb/2019-May/2019	Adults	Hospital-based	Unclear/Not reported	ART naïve/ On ART	Pregnant women	HBV	Chemiluminescent enzyme immunoassay (CLEIA)	HBeAg
Yendewa	2021	Cross sectional	Non probabilistic	Convenience sampling	Multicenter	Prospetively	Sierra Leone	West and Central	Africa	West Africa	Low-income economies	Feb/2019-May/2019	Adults	Hospital-based	Unclear/Not reported	ART naïve/ On ART	Pregnant women	HBV	Chemiluminescent enzyme immunoassay (CLEIA)	HBsAg
Yendewa	2021	Cross sectional	Non probabilistic	Convenience sampling	Multicenter	Prospetively	Sierra Leone	West and Central	Africa	West Africa	Low-income economies	Feb/2019-May/2019	Adults	Hospital-based	Unclear/Not reported	ART naïve/ On ART	Pregnant women	HCV	Classical RT-PCR	Viral RNA
Yendewa	2021	Cross sectional	Non probabilistic	Convenience sampling	Multicenter	Prospetively	Sierra Leone	West and Central	Africa	West Africa	Low-income economies	Feb/2019-May/2019	Adults	Hospital-based	Unclear/Not reported	ART naïve/ On ART	Pregnant women	HCV	Chemiluminescent enzyme immunoassay (CLEIA)	Anti-HCV
Yendewa	2021	Cross sectional	Non probabilistic	Convenience sampling	Multicenter	Prospetively	Sierra Leone	West and Central	Africa	West Africa	Low-income economies	Feb/2019-May/2019	Adults	Hospital-based	Unclear/Not reported	ART naïve/ On ART	Pregnant women	HBV	Chemiluminescent enzyme immunoassay (CLEIA)	HBeAg
Yendewa	2021	Cross sectional	Non probabilistic	Convenience sampling	Multicenter	Prospetively	Sierra Leone	West and Central	Africa	West Africa	Low-income economies	Feb/2019-May/2019	Unclear/Not reported	Hospital-based	Unclear/Not reported	ART naïve/ On ART	Pregnant women	HBV	Chemiluminescent enzyme immunoassay (CLEIA)	HBsAg
Yendewa	2021	Cross sectional	Non probabilistic	Convenience sampling	Multicenter	Prospetively	Sierra Leone	West and Central	Africa	West Africa	Low-income economies	Feb/2019-May/2019	Adults	Hospital-based	Unclear/Not reported	ART naïve/ On ART	Pregnant women	HCV	Classical RT-PCR	Viral RNA
Yendewa	2021	Cross sectional	Non probabilistic	Convenience sampling	Multicenter	Prospetively	Sierra Leone	West and Central	Africa	West Africa	Low-income economies	Feb/2019-May/2019	Children	Hospital-based	Unclear/Not reported	ART naïve/ On ART	Pregnant women	HCV	Chemiluminescent enzyme immunoassay (CLEIA)	Anti-HCV
Yendewa	2021	Cross sectional	Non probabilistic	Convenience sampling	Multicenter	Prospetively	Sierra Leone	West and Central	Africa	West Africa	Low-income economies	Feb/2019-May/2019	Children	Hospital-based	Unclear/Not reported	ART naïve/ On ART	Pregnant women	HBV	Chemiluminescent enzyme immunoassay (CLEIA)	HBeAg
Yendewa	2021	Cross sectional	Non probabilistic	Convenience sampling	Multicenter	Prospetively	Sierra Leone	West and Central	Africa	West Africa	Low-income economies	Feb/2019-May/2019	Children	Hospital-based	Unclear/Not reported	ART naïve/ On ART	Pregnant women	HBV	Chemiluminescent enzyme immunoassay (CLEIA)	HBsAg
Yendewa	2021	Cross sectional	Non probabilistic	Convenience sampling	Multicenter	Prospetively	Sierra Leone	West and Central	Africa	West Africa	Low-income economies	Feb/2019-May/2019	Children	Hospital-based	Unclear/Not reported	ART naïve/ On ART	Pregnant women	HCV	Classical RT-PCR	Viral RNA
Yendewa	2019	Cross sectional	Non probabilistic	Consecutive sampling	Monocenter	Prospetively	Sierra Leone	West and Central	Africa	West Africa	Low-income economies	Nov/2017	Adults	Hospital-based	Unclear/Not reported	ART naïve/ On ART	General population	HCV	Chemiluminescent enzyme immunoassay (CLEIA)	Anti-HCV
Yendewa	2019	Cross sectional	Non probabilistic	Consecutive sampling	Monocenter	Prospetively	Sierra Leone	West and Central	Africa	West Africa	Low-income economies	Nov/2017	Adults	Hospital-based	Unclear/Not reported	ART naïve/ On ART	General population	HBV	Chemiluminescent enzyme immunoassay (CLEIA)	HBsAg
Yimer	2014	Cohort (Baseline data)	Non probabilistic	Consecutive sampling	Monocenter	Prospetively	Ethiopia	East and Southern	Africa	Eastern Africa	Low-income economies	Jun/2007-Jun/2011	Adults	Hospital-based	Unclear/Not reported	ART naïve	General population	HCV	Unclear/Not reported	Anti-HCV
Yimer	2014	Cohort (Baseline data)	Non probabilistic	Consecutive sampling	Monocenter	Prospetively	Ethiopia	East and Southern	Africa	Eastern Africa	Low-income economies	Jun/2007-Jun/2011	Adults	Hospital-based	Unclear/Not reported	ART naïve	General population	HBV	Unclear/Not reported	HBsAg
Zeba	2011	Cross sectional	Non probabilistic	Consecutive sampling	Monocenter	Prospetively	Burkina Faso	West and Central	Africa	West Africa	Low-income economies	Mar/2009-Sep/2009	Adults	Hospital-based	Unclear/Not reported	Unclear/Not reported	Pregnant women	HCV	Real-time RT-PCR	Viral RNA
Zeba	2011	Cross sectional	Non probabilistic	Consecutive sampling	Monocenter	Prospetively	Burkina Faso	West and Central	Africa	West Africa	Low-income economies	Mar/2009-Sep/2009	Adults	Hospital-based	Unclear/Not reported	Unclear/Not reported	Pregnant women	HCV	Rapid Diagnostic test	Anti-HCV
Zoufaly	2012	Cross sectional	Non probabilistic	Consecutive sampling	Monocenter	Retrospectively	Cameroon	West and Central	Africa	Central Africa	Lower-middle income economies	Jan/2005-Mar/2010	Adults	Hospital-based	Unclear/Not reported	On ART	General population	HBV	Rapid Diagnostic test	HBsAg
