# Supplementary material for: Epidemiology of hepatitis B virus and/or hepatitis C virus infections among people living with human immunodeficiency virus in Africa: A systematic review and meta-analysis
Source: PLoS One. 2022 May 31;17(5):e0269250. doi: 10.1371/journal.pone.0269250 (PMC9154112; doi:10.1371/journal.pone.0269250)
Supplement: S7 Table — (PDF) [file pone.0269250.s008.pdf]

S7 Table: Risk of bias assessment

Author	Year (Yea	Was the study's target populat	Was the sampling frame a true	Was some form of random sel	Were data collected directly fr	Was an acceptable inclusion c	Was the response rate ≥ 70 %	Was the HBV and/or HCV in P	Was the same mode of data c	Was the length of the study pe	Were the numerator(s) and de	Risk of bias	PLHIV Study population	HBV and/or HCV
Abera	2014	No	Yes	No	Yes	Yes	Yes	Yes	Yes	No	Yes	Low risk of bias	General population	HCV
Abera	2014	No	Yes	No	Yes	Yes	Yes	Yes	Yes	No	Yes	Low risk of bias	General population	HBV
Abroug	2020	No	Yes	No	Yes	Yes	Yes	Yes	Yes	No	Yes	Low risk of bias	Pregnant women	HBV
Adekunle	2011	No	Yes	No	Yes	Yes	Unclear	Yes	Yes	No	Yes	Moderate risk of bias	General population	HCV
Adekunle	2011	No	Yes	No	Yes	Yes	Unclear	Yes	Yes	No	Yes	Moderate risk of bias	General population	HBV
Adesina	2016	No	Yes	No	Yes	Yes	Unclear	Yes	Yes	Yes	Yes	Low risk of bias	Pregnant women	HCV
Adesina	2016	No	Yes	No	Yes	Yes	Unclear	Yes	Yes	Yes	Yes	Low risk of bias	Pregnant women	HBV and HCV
Adesina	2016	No	Yes	No	Yes	Yes	Unclear	Yes	Yes	Yes	Yes	Low risk of bias	Pregnant women	HBV
Adewole	2009	No	Yes	No	Yes	Yes	Yes	Yes	Yes	Unclear	Yes	Low risk of bias	General population	HCV
Adewole	2009	No	Yes	No	Yes	Yes	Yes	Yes	Yes	Unclear	Yes	Low risk of bias	General population	HBV
Adewumi	2015	No	Yes	No	Yes	Yes	Yes	Yes	Yes	No	Yes	Low risk of bias	General population	HBV
Adeyemi	2020	No	Yes	No	Yes	Yes	Yes	Yes	Yes	No	Yes	Low risk of bias	General population	HBV
Agbaji	2013	No	Yes	No	Yes	Yes	Yes	Yes	Yes	Yes	Yes	Low risk of bias	General population	HCV
Agbaji	2013	No	Yes	No	Yes	Yes	Yes	Yes	Yes	Yes	Yes	Low risk of bias	General population	HCV
Agwale	2004	No	Yes	No	Yes	Yes	Yes	Yes	Yes	Yes	Yes	Low risk of bias	General population	HCV
Ajayi	2011	No	Yes	No	Yes	Yes	Yes	Yes	Yes	No	Yes	Low risk of bias	General population	HCV
Ajayi	2011	No	Yes	No	Yes	Yes	Yes	Yes	Yes	No	Yes	Low risk of bias	General population	HBV
Akinbami	2020	No	Yes	No	Yes	No	Unclear	Yes	Yes	No	Yes	Moderate risk of bias	General population	HCV
Akinniyi	2021	No	Yes	No	Yes	Yes	No	Yes	Yes	Unclear	Yes	Moderate risk of bias	General population	HBV
Akinniyi	2021	No	Yes	No	Yes	Yes	No	Yes	Yes	Unclear	Yes	Moderate risk of bias	General population	HBV
Alemayehu	2011	No	Yes	No	Yes	Yes	Unclear	Yes	Yes	No	Yes	Moderate risk of bias	General population	HCV
Ambassa	2016	No	Yes	No	Yes	Yes	Unclear	Yes	Yes	No	Yes	Moderate risk of bias	General population	HBV
Amstutz	2020	No	Yes	No	Yes	Yes	Yes	Yes	Yes	No	Yes	Low risk of bias	General population	HCV
Amstutz	2020	No	Yes	No	Yes	Yes	Yes	Yes	Yes	No	Yes	Low risk of bias	General population	HBV
Amstutz	2020	No	Yes	No	Yes	Yes	Yes	Yes	Yes	No	Yes	Low risk of bias	General population	HCV
Amstutz	2020	No	Yes	No	Yes	Yes	Yes	Yes	Yes	No	Yes	Low risk of bias	General population	HBV
Andersson	2013	No	Yes	No	Yes	Yes	Yes	Yes	Yes	Unclear	Yes	Low risk of bias	Pregnant women	HBV
Andersson	2012	No	Yes	No	Yes	Yes	Yes	Yes	Yes	Yes	Yes	Low risk of bias	Pregnant women	HBV
Andreotti	2014	No	Yes	No	Yes	Yes	Yes	Yes	Yes	Yes	Yes	Low risk of bias	Pregnant women	HCV
Andreotti	2014	No	Yes	No	Yes	Yes	Yes	Yes	Yes	Yes	Yes	Low risk of bias	Pregnant women	HBV
Andreotti	2014	No	Yes	No	Yes	Yes	Yes	Yes	Yes	Yes	Yes	Low risk of bias	Pregnant women	HCV
Anyanwu	2020	No	Yes	Yes	Yes	Yes	Yes	Yes	Yes	No	Yes	Low risk of bias	General population	HCV
Anyanwu	2020	No	Yes	Yes	Yes	Yes	Yes	Yes	Yes	No	Yes	Low risk of bias	General population	HBV and HCV
Anyanwu	2020	No	Yes	Yes	Yes	Yes	Yes	Yes	Yes	No	Yes	Low risk of bias	General population	HBV
Archampon	2016	No	Yes	No	Yes	Yes	Yes	Yes	Yes	Yes	Yes	Low risk of bias	General population	HBV
Ashir	2009	No	Yes	No	Yes	Yes	Unclear	Yes	Yes	No	Yes	Moderate risk of bias	General population	HBV
Attia	2012	No	Yes	No	Yes	Yes	Yes	Yes	Yes	No	Yes	Low risk of bias	General population	HBV
Augusto	2019	No	Yes	Yes	Yes	Yes	Yes	Yes	Yes	Yes	Yes	Low risk of bias	Prisoners	HBV
Ayana	2019	No	Yes	No	Yes	Yes	Yes	Yes	Yes	No	Yes	Low risk of bias	General population	HBV
Ayele	2002	No	Yes	No	Yes	Yes	Yes	Yes	Yes	Unclear	Yes	Low risk of bias	General population	HCV
Ayelign	2021	No	Yes	No	Yes	Yes	No	Yes	Yes	Unclear	Yes	Moderate risk of bias	General population	HBV
Ayelign	2021	No	Yes	No	Yes	Yes	No	Yes	Yes	Unclear	Yes	Moderate risk of bias	General population	HCV
Ayuk	2013	No	Yes	No	Yes	Yes	Unclear	Yes	Yes	Unclear	Yes	Moderate risk of bias	General population	HBV
Bado	2013	No	Yes	No	Yes	Yes	Yes	Yes	Yes	No	Yes	Low risk of bias	General population	HBV
Balogun	2010	No	Yes	No	Yes	Yes	Unclear	Yes	Yes	No	Yes	Moderate risk of bias	General population	HBV
Balogun	2012	No	Yes	No	Yes	Yes	Unclear	Yes	Yes	No	Yes	Moderate risk of bias	General population	HCV
Balogun	2012	No	Yes	No	Yes	Yes	Unclear	Yes	Yes	No	Yes	Moderate risk of bias	General population	HBV and HCV
Balogun	2012	No	Yes	No	Yes	Yes	Unclear	Yes	Yes	No	Yes	Moderate risk of bias	General population	HBV
Barros	1996	No	Yes	No	Yes	Yes	Unclear	Yes	Yes	Unclear	Yes	Moderate risk of bias	General population	HBV
Barth	2011	No	Yes	No	Yes	Yes	Unclear	Yes	Yes	No	Yes	Moderate risk of bias	General population	HCV
Barth	2011	No	Yes	No	Yes	Yes	Unclear	Yes	Yes	No	Yes	Moderate risk of bias	General population	HBV
Baseke	2015	No	Yes	Yes	Yes	Yes	Yes	Yes	Yes	Unclear	Yes	Low risk of bias	General population	HCV
Baseke	2015	No	Yes	Yes	Yes	Yes	Yes	Yes	Yes	Unclear	Yes	Low risk of bias	General population	HBV and HCV
Baseke	2015	No	Yes	Yes	Yes	Yes	Yes	Yes	Yes	Unclear	Yes	Low risk of bias	General population	HBV
Beghin	2017	No	Yes	No	Yes	Yes	Yes	Yes	Yes	No	Yes	Low risk of bias	General population	HBV
Bell	2012	No	Yes	No	Yes	Yes	Yes	Yes	Yes	No	Yes	Low risk of bias	General population	HBV
Bell	2012	No	Yes	No	Yes	Yes	Yes	Yes	Yes	No	Yes	Low risk of bias	General population	HBV
Bessimbaye	2014	No	Yes	No	Yes	Yes	Unclear	Yes	Yes	Unclear	Yes	Moderate risk of bias	General population	HCV
Bessimbaye	2014	No	Yes	No	Yes	Yes	Unclear	Yes	Yes	Unclear	Yes	Moderate risk of bias	General population	HBV and HCV
Bessimbaye	2014	No	Yes	No	Yes	Yes	Unclear	Yes	Yes	Unclear	Yes	Moderate risk of bias	General population	HBV
Bhattachary	2021	No	Yes	Yes	Yes	Yes	Unclear	Yes	Yes	Unclear	Yes	Low risk of bias	Pregnant women	HBV
Bhattachary	2021	No	Yes	Yes	Yes	Yes	Unclear	Yes	Yes	Unclear	Yes	Low risk of bias	Pregnant women	HBV
Bivigou-Mb	2016	No	Yes	No	Yes	Yes	Unclear	Yes	Yes	Yes	Yes	Low risk of bias	General population	HCV
Boateng	2019	No	Yes	Yes	Yes	Yes	Unclear	Yes	Yes	No	Yes	Low risk of bias	General population	HCV
Boateng	2019	No	Yes	Yes	Yes	Yes	Unclear	Yes	Yes	No	Yes	Low risk of bias	General population	HBV
Burnett	2007	No	Yes	No	Yes	Yes	Unclear	Yes	Yes	Yes	Yes	Low risk of bias	Pregnant women	HBV
Burnett	2007	No	Yes	No	Yes	Yes	Unclear	Yes	Yes	Yes	Yes	Low risk of bias	Pregnant women	HBV
Calisti	2015	No	Yes	No	Yes	Yes	Yes	Yes	Yes	Yes	Yes	Low risk of bias	General population	HBV
Carimo	2018	No	Yes	No	Yes	Yes	Unclear	Yes	Yes	No	Yes	Moderate risk of bias	General population	HBV
Chambal	2017	No	Yes	No	Yes	Yes	Unclear	Yes	Yes	No	Yes	Moderate risk of bias	General population	HBV
Chasela	2012	No	Yes	No	Yes	Yes	Unclear	Yes	Yes	Unclear	Yes	Moderate risk of bias	Pregnant women	HBV
Cherry	2010	No	Yes	No	Yes	Yes	Unclear	Yes	Yes	Unclear	Yes	Moderate risk of bias	General population	HCV
Chiesa	2020	No	Yes	Yes	Yes	Yes	Unclear	Yes	Yes	No	Yes	Low risk of bias	General population	HBV
Chisenga	2018	No	Yes	No	Yes	Yes	Unclear	Yes	Yes	Unclear	Yes	Moderate risk of bias	General population	HBV
Chisenga	2018	No	Yes	No	Yes	Yes	Unclear	Yes	Yes	Unclear	Yes	Moderate risk of bias	General population	HBV
Coffie	2017	No	Yes	Yes	Yes	Yes	Unclear	No	No	Unclear	Yes	Moderate risk of bias	General population	HBV
Coffie	2017	No	Yes	No	Yes	Yes	Unclear	Yes	Yes	No	Yes	Moderate risk of bias	General population	HBV
Coffie	2017	No	Yes	No	Yes	Yes	Unclear	Yes	Yes	No	Yes	Moderate risk of bias	General population	HBV
Coffie	2017	No	Yes	No	Yes	Yes	Unclear	Yes	Yes	No	Yes	Moderate risk of bias	General population	HBV
Combe	2001	No	Yes	No	Yes	Yes	Yes	Yes	Yes	No	Yes	Low risk of bias	General population	HBV
Combe	2001	No	Yes	No	Yes	Yes	Yes	Yes	Yes	No	Yes	Low risk of bias	General population	HBV
Combe	2001	No	Yes	No	Yes	Yes	Yes	Yes	Yes	No	Yes	Low risk of bias	General population	HCV
Day	2013	No	Yes	No	Yes	Yes	Unclear	Yes	Yes	Yes	Yes	Low risk of bias	General population	HBV
Day	2013	No	Yes	No	Yes	Yes	Unclear	Yes	Yes	Yes	Yes	Low risk of bias	General population	CFR
Day	2013	No	Yes	No	Yes	Yes	Unclear	Yes	Yes	Yes	Yes	Low risk of bias	General population	HBV
Demir	2018	No	Yes	No	Yes	Yes	Unclear	Yes	Yes	Unclear	Yes	Moderate risk of bias	General population	HBV
Deressa	2017	No	Yes	Yes	Yes	Yes	Unclear	Yes	Yes	No	Yes	Low risk of bias	General population	HBV
Diale	2015	No	Yes	No	Yes	Yes	Unclear	Yes	Yes	Yes	Yes	Low risk of bias	Pregnant women	HBV
Diale	2015	No	Yes	No	Yes	Yes	Unclear	Yes	Yes	Yes	Yes	Low risk of bias	Pregnant women	HBV
Diarra	2006	No	Yes	No	Yes	Yes	Unclear	Yes	Yes	Unclear	Yes	Moderate risk of bias	General population	HCV
Diarra	2006	No	Yes	No	Yes	Yes	Unclear	Yes	Yes	Unclear	Yes	Moderate risk of bias	General population	HBV
Diwe	2013	No	Yes	No	Yes	Yes	Unclear	Yes	Yes	No	Yes	Moderate risk of bias	General population	HCV
Diwe	2013	No	Yes	No	Yes	Yes	Unclear	Yes	Yes	No	Yes	Moderate risk of bias	General population	HBV and HCV
Diwe	2013	No	Yes	No	Yes	Yes	Unclear	Yes	Yes	No	Yes	Moderate risk of bias	General population	HBV
Dovonou	2015	No	Yes	No	Yes	Yes	Yes	Yes	Yes	Yes	Yes	Low risk of bias	General population	HBV
Dziuban	2013	No	Yes	No	Yes	Yes	Unclear	Yes	Yes	Yes	Yes	Low risk of bias	General population	HBV
Ejele	2004	No	Yes	No	Yes	Yes	Unclear	Yes	Yes	Yes	Yes	Low risk of bias	General population	HBV
Ekouevi	2018	No	Yes	No	Yes	Yes	Unclear	Yes	Yes	No	Yes	Moderate risk of bias	General population	HCV
Ekouevi	2018	No	Yes	No	Yes	Yes	Unclear	Yes	Yes	No	Yes	Moderate risk of bias	General population	HCV
Elsharkawy	2021	No	Yes	No	Yes	Yes	No	Yes	Yes	No	Yes	Moderate risk of bias	General population	HCV
Eze	2014	No	Yes	No	Yes	Yes	Unclear	Yes	Yes	No	Yes	Moderate risk of bias	General population	HCV
Feldt	2013	No	Yes	No	Yes	Yes	Unclear	Yes	Yes	Yes	Yes	Low risk of bias	General population	HBV
Feldt	2013	No	Yes	No	Yes	Yes	Unclear	Yes	Yes	Yes	Yes	Low risk of bias	General population	HCV

Feldt	2013	No	Yes	No	Yes	Yes	Unclear	Yes	Yes	Yes	Yes	Low risk of bias	General population	HCV
Feldt	2013	No	Yes	No	Yes	Yes	Unclear	Yes	Yes	Yes	Yes	Low risk of bias	General population	HBV
Firnhaber	2008	No	Yes	No	Yes	Yes	Unclear	Yes	Yes	Unclear	Yes	Moderate risk of bias	General population	HBV
Forbi	2007	No	Yes	Yes	Yes	Yes	Unclear	Yes	Yes	No	Yes	Low risk of bias	General population	HCV
Forbi	2007	No	Yes	Yes	Yes	Yes	Unclear	Yes	Yes	No	Yes	Low risk of bias	General population	HBV and HCV
Forbi	2007	No	Yes	Yes	Yes	Yes	Unclear	Yes	Yes	No	Yes	Low risk of bias	General population	HBV and HCV
Forbi	2007	No	Yes	Yes	Yes	Yes	Unclear	Yes	Yes	No	Yes	Low risk of bias	General population	HBV
Franzeck	2013	No	Yes	No	Yes	Yes	Yes	Yes	Yes	No	Yes	Low risk of bias	General population	HBV
Franzeck	2013	No	Yes	No	Yes	Yes	Yes	Yes	Yes	No	Yes	Low risk of bias	General population	HCV
Franzeck	2013	No	Yes	No	Yes	Yes	Yes	Yes	Yes	No	Yes	Low risk of bias	General population	HBV and HCV
Frempong	2019	No	Yes	No	Yes	Yes	Unclear	Yes	Yes	Yes	Yes	Low risk of bias	Pregnant women	HCV
Frempong	2019	No	Yes	No	Yes	Yes	Unclear	Yes	Yes	Yes	Yes	Low risk of bias	Pregnant women	HBV
Frempong	2019	No	Yes	No	Yes	Yes	Unclear	Yes	Yes	Yes	Yes	Low risk of bias	Pregnant women	HBV
Gededzha	2010	No	Yes	No	Yes	Yes	Unclear	Yes	Yes	Yes	Yes	Low risk of bias	General population	HCV
Gededzha	2018	No	Yes	No	Yes	Yes	Unclear	Yes	Yes	Yes	Yes	Low risk of bias	General population	HBV
Gededzha	2018	No	Yes	No	Yes	Yes	Unclear	Yes	Yes	Yes	Yes	Low risk of bias	General population	HBV
Gededzha	2018	No	Yes	No	Yes	Yes	Unclear	Yes	Yes	Yes	Yes	Low risk of bias	General population	HBV
Gedefie	2021	No	Yes	No	Yes	Yes	Yes	Yes	Yes	No	Yes	Low risk of bias	General population	HCV
George	2018	No	Yes	No	Yes	Yes	Unclear	Yes	Yes	Yes	Yes	Low risk of bias	General population	HCV
George	2018	No	Yes	No	Yes	Yes	Unclear	Yes	Yes	Yes	Yes	Low risk of bias	General population	HBV
Geretti	2010	No	Yes	No	Yes	Yes	Unclear	Yes	Yes	Unclear	Yes	Moderate risk of bias	General population	HBV
Geretti	2010	No	Yes	No	Yes	Yes	Unclear	Yes	Yes	Unclear	Yes	Moderate risk of bias	General population	HBV
Giuliano	2018	No	Yes	No	Yes	Yes	Unclear	Yes	Yes	Yes	Yes	Low risk of bias	Pregnant women	HBV
Goa	2019	No	Yes	Yes	Yes	Yes	Unclear	Yes	Yes	No	Yes	Low risk of bias	General population	HBV
Gogela	2018	No	Yes	No	Yes	Yes	Unclear	Yes	Yes	Yes	Yes	Low risk of bias	General population	HCV
Goverwa-S	2020	No	Yes	No	Yes	Yes	Yes	Yes	Yes	Yes	Yes	Low risk of bias	General population	HBV
Greer	2017	No	Yes	Yes	Yes	Yes	Unclear	Unclear	Yes	Unclear	Yes	Moderate risk of bias	General population	HBV
Greer	2017	No	Yes	Yes	Yes	Yes	Unclear	Unclear	Yes	Unclear	Yes	Moderate risk of bias	General population	HBV
Greer	2017	No	Yes	Yes	Yes	Yes	Unclear	Unclear	Yes	Unclear	Yes	Moderate risk of bias	General population	HBV
Greer	2017	No	Yes	Yes	Yes	Yes	Unclear	Unclear	Yes	Unclear	Yes	Moderate risk of bias	General population	HBV
Gudo	2018	No	Yes	No	Yes	Yes	Unclear	Yes	Yes	No	Yes	Moderate risk of bias	General population	HBV
Guimarães	2013	No	Yes	No	Yes	Yes	Unclear	Yes	Yes	Unclear	Yes	Moderate risk of bias	General population	HCV
Guimarães	2013	No	Yes	No	Yes	Yes	Unclear	Yes	Yes	Unclear	Yes	Moderate risk of bias	General population	HBV
Hadush	2013	No	Yes	No	Yes	Yes	Unclear	Yes	Yes	No	Yes	Moderate risk of bias	General population	HCV
Harvard	2019	No	Yes	No	Yes	Yes	Unclear	Yes	Yes	Unclear	Yes	Moderate risk of bias	General population	HBV
Harvard	2019	No	Yes	No	Yes	Yes	Unclear	Yes	Yes	Unclear	Yes	Moderate risk of bias	General population	HBV
Hawkins	2013	No	Yes	No	Yes	Yes	Unclear	Yes	Yes	Yes	Yes	Low risk of bias	General population	HBV
Hector	2018	No	Yes	No	Yes	Yes	Unclear	Yes	Yes	No	Yes	Moderate risk of bias	General population	HBV
Hoffmann	2012	No	Yes	No	Yes	Yes	Unclear	Yes	Yes	Unclear	Yes	Moderate risk of bias	Pregnant women	HBV
Hoffmann	2012	No	Yes	No	Yes	Yes	Unclear	Yes	Yes	Unclear	Yes	Moderate risk of bias	Pregnant women	HCV
Hoffmann	2014	No	Yes	No	Yes	Yes	Unclear	Yes	Yes	Unclear	Yes	Moderate risk of bias	Pregnant women	HBV
Hønge	2014	No	Yes	No	Yes	Yes	Yes	Yes	Yes	No	Yes	Low risk of bias	General population	HCV
Hønge	2014	No	Yes	No	Yes	Yes	Yes	Yes	Yes	No	Yes	Low risk of bias	General population	HBV
Hønge	2014	No	Yes	No	Yes	Yes	Yes	Yes	Yes	No	Yes	Low risk of bias	General population	HCV
Hønge	2014	No	Yes	No	Yes	Yes	Unclear	Yes	Yes	No	Yes	Moderate risk of bias	General population	HBV
Houghtaling	2018	No	Yes	Yes	Yes	Yes	Yes	Yes	Yes	Yes	Yes	Low risk of bias	General population	HBV
Ifeorah	2017	No	Yes	No	Yes	Yes	Unclear	Yes	Yes	No	Yes	Moderate risk of bias	General population	HBV
Ikomey	2016	No	Yes	No	Yes	Yes	Unclear	Yes	Yes	No	Yes	Moderate risk of bias	Pregnant women	HCV
Ikomey	2016	No	Yes	No	Yes	Yes	Unclear	Yes	Yes	No	Yes	Moderate risk of bias	Pregnant women	HBV and HCV
Ikomey	2016	No	Yes	No	Yes	Yes	Unclear	Yes	Yes	No	Yes	Moderate risk of bias	Pregnant women	HBV
Ikpeme	2013	No	Yes	No	Yes	Yes	Unclear	Yes	Yes	No	Yes	Moderate risk of bias	General population	HBV
Ilboudo	2007	No	Yes	No	Yes	Yes	No	Yes	Yes	Yes	Yes	Low risk of bias	Pregnant women	HBV
Ive	2013	No	Yes	Yes	Yes	Yes	Unclear	Yes	Yes	Yes	Yes	Low risk of bias	General population	HBV
Iwalokun	2006	No	Yes	No	Yes	Yes	Unclear	Yes	Yes	Unclear	Yes	Moderate risk of bias	General population	HBV
Jackson	1991	No	Yes	No	Yes	Yes	No	Yes	Yes	No	Yes	Moderate risk of bias	Pregnant women	HCV
Jaquet	2017	No	Yes	No	Yes	Yes	Unclear	Yes	Yes	Unclear	Yes	Moderate risk of bias	General population	HCV
Jaquet	2017	No	Yes	No	Yes	Yes	Unclear	Yes	Yes	Unclear	Yes	Moderate risk of bias	General population	HBV
Jaquet	2017	No	Yes	No	Yes	Yes	Unclear	Yes	Yes	Unclear	Yes	Moderate risk of bias	General population	HCV
Jaquet	2017	No	Yes	No	Yes	Yes	Unclear	Yes	Yes	Unclear	Yes	Moderate risk of bias	General population	HBV
Jaquet	2017	No	Yes	No	Yes	Yes	Unclear	Yes	Yes	Unclear	Yes	Moderate risk of bias	General population	HCV
Jaquet	2017	No	Yes	No	Yes	Yes	Unclear	Yes	Yes	Unclear	Yes	Moderate risk of bias	General population	HBV
Jobarteh	2010	No	Yes	No	Yes	Yes	Unclear	Yes	Yes	Yes	Yes	Low risk of bias	General population	HBV
Jobarteh	2010	No	Yes	No	Yes	Yes	Unclear	Yes	Yes	Yes	Yes	Low risk of bias	General population	HCV
Jooste	2016	No	Yes	No	Yes	Yes	Unclear	Yes	Yes	No	Yes	Moderate risk of bias	General population	HBV
Joseph	2019	No	Yes	No	Yes	Yes	Unclear	Yes	Yes	No	Yes	Moderate risk of bias	General population	HBV
Kaba	2019	No	Yes	No	Yes	Yes	Unclear	Yes	Yes	No	Yes	Moderate risk of bias	General population	HBV
Kamenya	2017	No	Yes	Yes	Yes	Yes	Yes	Yes	Yes	No	Yes	Low risk of bias	General population	HBV
Kashala	1994	No	Yes	Yes	Yes	Yes	Yes	Yes	Yes	Yes	Yes	Low risk of bias	General population	HBV
Kashala	1994	No	Yes	Yes	Yes	Yes	Yes	Yes	Yes	Yes	Yes	Low risk of bias	General population	HBV
Kashala	1994	No	Yes	Yes	Yes	Yes	Yes	Yes	Yes	Yes	Yes	Low risk of bias	General population	HBV
Katusiime	2016	No	Yes	No	Yes	Yes	Yes	Yes	Yes	No	Yes	Low risk of bias	General population	HBV
Kfutwah	2012	No	Yes	No	Yes	Yes	Yes	Yes	Yes	Yes	Yes	Low risk of bias	Pregnant women	HBV
Kibaya	2015	No	Yes	No	Yes	Yes	Yes	Yes	Yes	No	Yes	Low risk of bias	Injecting drug users	HBV
Kilonzo	2017	No	Yes	No	Yes	Yes	No	No	Unclear	Yes	Yes	Moderate risk of bias	General population	HBV
King	2015	No	Yes	No	Yes	Yes	No	Yes	Yes	Yes	Yes	Low risk of bias	General population	HCV
King	2016	No	Yes	No	Yes	Yes	No	No	No	Yes	Yes	Moderate risk of bias	General population	HBV
Kirakoya-Sa	2014	No	Yes	No	Yes	Yes	No	Yes	Yes	Unclear	Yes	Moderate risk of bias	Blood donors	HCV
Kirakoya-Sa	2014	No	Yes	No	Yes	Yes	No	Yes	Yes	Unclear	Yes	Moderate risk of bias	Blood donors	HBV
Kouamé	2018	No	Yes	Yes	Yes	Yes	No	Yes	Yes	Yes	Yes	Low risk of bias	General population	HBV
Kouamé	2018	No	Yes	Yes	Yes	Yes	No	Yes	Yes	Yes	Yes	Low risk of bias	General population	CFR
Kouanfack	2012	No	Yes	No	Yes	Yes	Yes	Yes	Yes	Yes	Yes	Low risk of bias	General population	HBV
Kwofie	2021	No	Yes	No	Yes	Yes	No	Yes	Yes	Yes	Yes	Low risk of bias	General population	HBV
Kwofie	2021	No	Yes	No	Yes	Yes	No	Yes	Yes	Yes	Yes	Low risk of bias	General population	HCV
Kwofie	2021	No	Yes	No	Yes	Yes	No	Yes	Yes	Yes	Yes	Low risk of bias	General population	HBV and HCV
Kye-Duodu	2016	No	Yes	No	Yes	Yes	Yes	Yes	Yes	No	Yes	Low risk of bias	General population	HBV
Ladep	2007	No	Yes	No	Yes	Yes	Yes	Yes	Yes	Yes	Yes	Low risk of bias	General population	HCV
Ladep	2013	No	Yes	No	Yes	Yes	No	Yes	Yes	Yes	Yes	Low risk of bias	General population	HBV
Ladep	2013	No	Yes	No	Yes	Yes	No	Yes	Yes	Yes	Yes	Low risk of bias	General population	CFR
Laurent	2010	No	Yes	No	Yes	Yes	Yes	Yes	Yes	Yes	Yes	Low risk of bias	General population	HCV
Laurent	2010	No	Yes	No	Yes	Yes	Yes	Yes	Yes	Yes	Yes	Low risk of bias	General population	HBV
Laurent	2010	No	Yes	No	Yes	Yes	Yes	Yes	Yes	Yes	Yes	Low risk of bias	General population	HBV and HCV
Laurent	2010	No	Yes	No	Yes	Yes	Yes	Yes	Yes	Yes	Yes	Low risk of bias	General population	HCV
Lawal	2020	No	Yes	Yes	Yes	Yes	Yes	Yes	Yes	No	Yes	Low risk of bias	General population	HCV
Lawal	2020	No	Yes	Yes	Yes	Yes	Yes	Yes	Yes	No	Yes	Low risk of bias	General population	HBV and HCV
Lawal	2020	No	Yes	Yes	Yes	Yes	Yes	Yes	Yes	No	Yes	Low risk of bias	General population	HBV
Lesi	2007	No	Yes	No	Yes	Yes	Yes	Yes	Yes	Unclear	Yes	Low risk of bias	General population	HCV
Lesi	2007	No	Yes	No	Yes	Yes	Yes	Yes	Yes	Unclear	Yes	Low risk of bias	General population	HBV
Liégeois	2020	No	Yes	No	Yes	Yes	Yes	Yes	Yes	No	Yes	Low risk of bias	General population	HBV
Lô	2016	No	Yes	No	Yes	Yes	Unclear	Yes	Yes	Yes	Yes	Low risk of bias	General population	HBV
Loaarec	2019	No	Yes	No	Yes	Yes	Yes	Yes	Yes	Unclear	Yes	Low risk of bias	General population	HCV
Loaarec	2019	No	Yes	No	Yes	Yes	Yes	Yes	Yes	Unclear	Yes	Low risk of bias	General population	HCV
Loaarec	2019	No	Yes	No	Yes	Yes	Yes	Yes	Yes	Unclear	Yes	Low risk of bias	General population	HCV
Loaarec	2019	No	Yes	No	Yes	Yes	Yes	Yes	Yes	Unclear	Yes	Low risk of bias	General population	HCV
Lodenyo	2000	No	Yes	No	Yes	Yes	Unclear	Yes	Yes	No	Yes	Moderate risk of bias	General population	HCV
Lodenyo	2000	No	Yes	No	Yes	Yes	Unclear	Yes	Yes	No	Yes	Moderate risk of bias	General population	HBV
Lodenyo	2000	No	Yes	No	Yes	Yes	Unclear	Yes	Yes	No	Yes	Moderate risk of bias	General population	HBV

Lukhwareni	2009	No	Yes	No	Yes	Yes	Unclear	Yes	Yes	Yes	Yes	Low risk of bias	General population	HBV
Maaref	2011	No	Yes	No	Yes	Yes	Unclear	Yes	Yes	Yes	No	Moderate risk of bias	General population	HCV
Maaref	2011	No	Yes	No	Yes	Yes	Unclear	Yes	Yes	Yes	No	Moderate risk of bias	General population	HBV
Mabeya	2016	No	Yes	Yes	Yes	Yes	No	Yes	Yes	No	Yes	Low risk of bias	General population	HCV
Mabeya	2016	No	Yes	Yes	Yes	Yes	No	Yes	Yes	No	Yes	Low risk of bias	General population	HBV
Magaji	2021	No	Yes	No	Yes	Yes	No	Yes	Yes	No	Yes	Moderate risk of bias	Pregnant women	HBV
Magoro	2016	No	Yes	No	Yes	Yes	Unclear	Yes	Yes	No	Yes	Moderate risk of bias	General population	HBV
Makanera	2019	No	Yes	Yes	Yes	Yes	Yes	Yes	Yes	No	Yes	Low risk of bias	General population	HBV
Manyazewa	2014	No	Yes	Yes	Yes	Yes	Unclear	Yes	Yes	Unclear	Yes	Low risk of bias	General population	HCV
Manyazewa	2014	No	Yes	Yes	Yes	Yes	Unclear	Yes	Yes	Unclear	Yes	Low risk of bias	General population	HBV
Manyazewa	2014	No	Yes	Yes	Yes	Yes	Unclear	Yes	Yes	Unclear	Yes	Low risk of bias	General population	HBV
Matthews	2015	No	Yes	No	Yes	Yes	No	Yes	Yes	Yes	Yes	Low risk of bias	General population	HBV
Matthews	2016	No	Yes	No	Yes	Yes	No	Yes	Yes	Unclear	Yes	Moderate risk of bias	General population	HBV
Matthews	2015	No	Yes	No	Yes	Yes	No	Yes	Yes	Yes	Yes	Low risk of bias	General population	HBV
Matthews	2016	No	Yes	No	Yes	Yes	No	Yes	Yes	Unclear	Yes	Moderate risk of bias	General population	HBV
Mayaphi	2012	No	Yes	No	Yes	Yes	Yes	Yes	Yes	Yes	Yes	Low risk of bias	General population	HBV
Mayaphi	2012	No	Yes	No	Yes	Yes	Yes	Yes	Yes	Yes	Yes	Low risk of bias	General population	HBV
Mboto	2010	No	Yes	No	Yes	Yes	No	Yes	Yes	No	Yes	Moderate risk of bias	General population	HCV
Mdlalose	2016	No	Yes	Yes	Yes	Yes	Yes	Yes	Yes	Yes	Yes	Low risk of bias	General population	HBV
Molu	2018	No	Yes	No	Yes	Yes	Yes	Yes	Yes	Yes	Yes	Low risk of bias	General population	HBV
Mphahlele	2006	No	Yes	No	Yes	Yes	Yes	Yes	Yes	Yes	Yes	Low risk of bias	General population	HCV
Mphahlele	2006	No	Yes	No	Yes	Yes	Yes	Yes	Yes	Yes	Yes	Low risk of bias	General population	HBV
Mphahlele	2006	No	Yes	No	Yes	Yes	Yes	Yes	Yes	Yes	Yes	Low risk of bias	General population	HBV
Mpody	2019	No	Yes	Yes	Yes	Yes	Yes	Yes	Yes	Yes	Yes	Low risk of bias	Pregnant women	HBV
Mpody	2019	No	Yes	Yes	Yes	Yes	Yes	Yes	Yes	Yes	Yes	Low risk of bias	Pregnant women	HBV
Mpody	2019	No	Yes	Yes	Yes	Yes	Yes	Yes	Yes	Yes	Yes	Low risk of bias	Pregnant women	HBV
Msomi	2020	No	Yes	Yes	Yes	Yes	Unclear	Yes	Yes	Unclear	Yes	Low risk of bias	General population	HBV
Mudawi	2014	No	Yes	No	Yes	Yes	Unclear	Yes	Yes	Yes	Yes	Low risk of bias	General population	HCV
Mudawi	2014	No	Yes	No	Yes	Yes	Unclear	Yes	Yes	Yes	Yes	Low risk of bias	General population	HBV
Mudawi	2014	No	Yes	No	Yes	Yes	Unclear	Yes	Yes	Yes	Yes	Low risk of bias	General population	HBV
Munyemana	2021	No	Yes	No	Yes	Yes	No	Yes	Yes	No	Yes	Moderate risk of bias	General population	HCV
Muriuki	2013	No	Yes	Yes	Yes	Yes	Unclear	Yes	Yes	No	Yes	Low risk of bias	General population	HCV
Muriuki	2013	No	Yes	Yes	Yes	Yes	Unclear	Yes	Yes	No	Yes	Low risk of bias	General population	HBV and HCV
Muriuki	2013	No	Yes	Yes	Yes	Yes	Unclear	Yes	Yes	No	Yes	Low risk of bias	General population	HBV
Musa	2015	No	Yes	No	Yes	Yes	Unclear	Yes	Yes	Yes	Yes	Low risk of bias	General population	HCV
Musyoki	2015	No	Yes	No	Yes	Yes	Unclear	Yes	Yes	Yes	Yes	Low risk of bias	Patients with cancer	HCV
Musyoki	2015	No	Yes	No	Yes	Yes	Unclear	Yes	Yes	Yes	Yes	Low risk of bias	Patients with cancer	HBV
Musyoki	2015	No	Yes	No	Yes	Yes	Unclear	Yes	Yes	Yes	Yes	Low risk of bias	Patients with cancer	HBV
Musyoki	2015	No	Yes	No	Yes	Yes	Unclear	Yes	Yes	Yes	Yes	Low risk of bias	Patients with cancer	HCV
Mutwa	2013	No	Yes	No	Yes	Yes	Yes	Yes	Yes	No	Yes	Low risk of bias	General population	HBV
Mweemba	2014	No	Yes	No	Yes	Yes	No	Yes	Yes	Yes	Yes	Low risk of bias	General population	HBV
Nagu	2008	No	Yes	No	Yes	Yes	Unclear	Yes	Yes	No	Yes	Moderate risk of bias	General population	HCV
Nagu	2008	No	Yes	No	Yes	Yes	Unclear	Yes	Yes	No	Yes	Moderate risk of bias	General population	HBV and HCV
Nagu	2008	No	Yes	No	Yes	Yes	Unclear	Yes	Yes	No	Yes	Moderate risk of bias	General population	HBV
Nagu	2008	No	Yes	No	Yes	Yes	Unclear	Yes	Yes	No	Yes	Moderate risk of bias	General population	HBV
Naniche	2011	No	Yes	No	Yes	Yes	Unclear	Yes	Yes	Yes	Yes	Low risk of bias	General population	HCV
Naniche	2011	No	Yes	No	Yes	Yes	Unclear	Yes	Yes	Yes	Yes	Low risk of bias	General population	HBV
Ndjomou	2002	No	Yes	No	Yes	Yes	Unclear	Yes	Yes	Unclear	Yes	Moderate risk of bias	General population	HCV
Ndjoyi-Mbig	2018	No	Yes	No	Yes	Yes	Unclear	Yes	Yes	No	Yes	Moderate risk of bias	General population	HCV
Ndow	2017	No	Yes	No	Yes	Yes	Unclear	Yes	Yes	No	Yes	Moderate risk of bias	General population	HBV
Ndow	2017	No	Yes	No	Yes	Yes	Unclear	Yes	Yes	No	Yes	Moderate risk of bias	General population	CFR
N'Dri-Yoma	2010	No	Yes	No	Yes	Yes	Unclear	Yes	Yes	No	Yes	Moderate risk of bias	General population	HBV
N'Dri-Yoma	2010	No	Yes	No	Yes	Yes	Unclear	Yes	Yes	No	Yes	Moderate risk of bias	General population	HBV
Newton	2015	No	Yes	No	Yes	Yes	Unclear	Yes	Yes	No	Yes	Moderate risk of bias	General population	HCV
Nkengason	1994	No	Yes	Yes	Yes	Yes	Yes	Yes	Yes	No	Yes	Low risk of bias	General population	HCV
Nnakenyi	2020	No	Yes	No	Yes	Yes	Yes	Yes	Yes	Yes	Yes	Low risk of bias	General population	HCV
Nnakenyi	2020	No	Yes	No	Yes	Yes	Yes	Yes	Yes	Yes	Yes	Low risk of bias	General population	HBV and HCV
Nnakenyi	2020	No	Yes	No	Yes	Yes	Yes	Yes	Yes	Yes	Yes	Low risk of bias	General population	HBV
Noubiap	2015	No	Yes	No	Yes	Yes	Yes	Yes	Yes	Yes	Yes	Low risk of bias	General population	HCV
Noubiap	2015	No	Yes	No	Yes	Yes	Yes	Yes	Yes	Yes	Yes	Low risk of bias	General population	HBV
Noubiap	2015	No	Yes	No	Yes	Yes	Yes	Yes	Yes	Yes	Yes	Low risk of bias	General population	HBV and HCV
Noubiap	2015	No	Yes	No	Yes	Yes	Yes	Yes	Yes	Yes	Yes	Low risk of bias	General population	HBV
Nwokedi	2006	No	Yes	No	Yes	Yes	Unclear	Yes	Yes	No	Yes	Moderate risk of bias	General population	HBV
Nwolisa	2013	No	Yes	No	Yes	Yes	Yes	Yes	Yes	No	Yes	Low risk of bias	General population	HBV
Ocama	2010	No	Yes	No	Yes	Yes	Yes	Yes	Yes	Yes	Yes	Low risk of bias	General population	HBV
Ocama	2008	No	Yes	No	Yes	Yes	Yes	Yes	Yes	No	Yes	Low risk of bias	General population	HCV
Ocama	2008	No	Yes	No	Yes	Yes	Yes	Yes	Yes	No	Yes	Low risk of bias	General population	HBV
Ogutu	1990	No	Yes	No	Yes	Yes	Unclear	Yes	Yes	Unclear	Yes	Moderate risk of bias	General population	HBV
Ogwu-Richa	2015	No	Yes	No	Yes	Yes	Unclear	Yes	Yes	Unclear	Yes	Moderate risk of bias	General population	HCV
Ogwu-Richa	2015	No	Yes	No	Yes	Yes	Unclear	Yes	Yes	Unclear	Yes	Moderate risk of bias	General population	HBV and HCV
Ogwu-Richa	2015	No	Yes	No	Yes	Yes	Unclear	Yes	Yes	Unclear	Yes	Moderate risk of bias	General population	HBV
Ojide	2015	No	Yes	No	Yes	Yes	Unclear	Yes	Yes	No	Yes	Moderate risk of bias	General population	HCV
Ojide	2015	No	Yes	No	Yes	Yes	Unclear	Yes	Yes	No	Yes	Moderate risk of bias	General population	HBV and HCV
Ojide	2015	No	Yes	No	Yes	Yes	Unclear	Yes	Yes	No	Yes	Moderate risk of bias	General population	HBV
Okeke	2012	No	Yes	No	Yes	Yes	Yes	Yes	Yes	Yes	Yes	Low risk of bias	Pregnant women	HCV
Okeke	2012	No	Yes	No	Yes	Yes	Yes	Yes	Yes	Yes	Yes	Low risk of bias	Pregnant women	HBV and HCV
Okeke	2012	No	Yes	No	Yes	Yes	Yes	Yes	Yes	Yes	Yes	Low risk of bias	Pregnant women	HBV
Okocha	2012	No	Yes	No	Yes	Yes	Unclear	Yes	Yes	Yes	Yes	Low risk of bias	General population	HBV
Okonko	2017	No	Yes	No	Yes	Yes	Unclear	Yes	Yes	No	Yes	Moderate risk of bias	Blood donors	HBV
Okoth	2017	No	Yes	No	Yes	Yes	Unclear	Yes	Yes	No	Yes	Moderate risk of bias	General population	HCV
Okoth	2017	No	Yes	No	Yes	Yes	Unclear	Yes	Yes	No	Yes	Moderate risk of bias	General population	HBV
Okwuraiwe	2012	No	Yes	No	Yes	Yes	No	Yes	Yes	Yes	Yes	Low risk of bias	General population	HCV
Okwuraiwe	2012	No	Yes	No	Yes	Yes	No	Yes	Yes	Yes	Yes	Low risk of bias	General population	HBV and HCV
Okwuraiwe	2012	No	Yes	No	Yes	Yes	No	Yes	Yes	Yes	Yes	Low risk of bias	General population	HBV
Olatunji	2008	No	Yes	No	Yes	Yes	Unclear	Yes	Yes	Unclear	Yes	Moderate risk of bias	General population	HCV
Olatunji	2008	No	Yes	No	Yes	Yes	Unclear	Yes	Yes	Unclear	Yes	Moderate risk of bias	General population	HBV
Olawumi	2014	No	Yes	No	Yes	Yes	Yes	Yes	Yes	No	Yes	Low risk of bias	General population	HBV
Omatola	2019	No	Yes	No	Yes	Yes	Unclear	Yes	Yes	No	Yes	Moderate risk of bias	General population	HBV
Omatola	2020	No	Yes	No	Yes	Yes	Unclear	Yes	Yes	Unclear	Yes	Moderate risk of bias	General population	HBV
Omatola	2017	No	Yes	Yes	Yes	Yes	Unclear	Yes	Yes	No	Yes	Low risk of bias	General population	HBV
Opaleye	2021	No	Yes	Yes	Yes	Yes	Unclear	Yes	Yes	No	Yes	Low risk of bias	General population	HBV
Oshitani	1996	No	Yes	No	Yes	Yes	Unclear	Yes	Yes	No	Yes	Moderate risk of bias	Pregnant women	HBV
Oshitani	1996	No	Yes	No	Yes	Yes	Unclear	Yes	Yes	No	Yes	Moderate risk of bias	Pregnant women	HBV
Otegbayo	2008	No	Yes	No	Yes	Yes	Yes	Yes	Yes	Yes	Yes	Low risk of bias	General population	HCV
Otegbayo	2008	No	Yes	No	Yes	Yes	Yes	Yes	Yes	Yes	Yes	Low risk of bias	General population	HBV
Ouattara	1990	No	Yes	No	Yes	Yes	Unclear	Yes	Yes	Yes	Yes	Low risk of bias	General population	HBV
Ouattara	1990	No	Yes	No	Yes	Yes	Unclear	Yes	Yes	Yes	Yes	Low risk of bias	General population	HBV
Ouattara	1990	No	Yes	No	Yes	Yes	Unclear	Yes	Yes	Yes	Yes	Low risk of bias	General population	HBV
Ouattara	1990	No	Yes	No	Yes	Yes	Unclear	Yes	Yes	Yes	Yes	Low risk of bias	General population	HBV
Ouattara	1990	No	Yes	No	Yes	Yes	Unclear	Yes	Yes	Yes	Yes	Low risk of bias	General population	HBV
Ouattara	1990	No	Yes	No	Yes	Yes	Unclear	Yes	Yes	Yes	Yes	Low risk of bias	General population	HBV
Ouermi	2009	No	Yes	No	Yes	Yes	Unclear	Yes	Yes	No	Yes	Moderate risk of bias	Pregnant women	HBV
Pappoe	2019	No	Yes	No	Yes	Yes	Unclear	Yes	Yes	No	Yes	Moderate risk of bias	General population	HCV
Pappoe	2019	No	Yes	No	Yes	Yes	Unclear	Yes	Yes	No	Yes	Moderate risk of bias	General population	HBV
Parboosing	2008	No	Yes	No	Yes	Yes	Unclear	Yes	Yes	No	Yes	Moderate risk of bias	General population	HBV

Patassi	2016	No	Yes	No	Yes	Yes	Unclear	Yes	Yes	Yes	Yes	Low risk of bias	General population	HBV
Patassi	2016	No	Yes	No	Yes	Yes	Unclear	Yes	Yes	Yes	Yes	Low risk of bias	General population	HBV
Patel	2011	No	Yes	No	Yes	Yes	Yes	Yes	Yes	Yes	Yes	Low risk of bias	General population	HCV
Patel	2011	No	Yes	No	Yes	Yes	Yes	Yes	Yes	Yes	Yes	Low risk of bias	General population	HBV
Peter	2015	No	Yes	No	Yes	Yes	Yes	Yes	Yes	No	Yes	Low risk of bias	General population	HCV
Peter	2015	No	Yes	No	Yes	Yes	Yes	Yes	Yes	No	Yes	Low risk of bias	General population	HBV
Pirillo	2015	No	Yes	No	Yes	Yes	Yes	Yes	Yes	Yes	Yes	Low risk of bias	Pregnant women	HBV
Pirillo	2015	No	Yes	No	Yes	Yes	Yes	Yes	Yes	Yes	Yes	Low risk of bias	Pregnant women	HBV
Pirillo	2007	No	Yes	No	Yes	Yes	Yes	Yes	Yes	Yes	Yes	Low risk of bias	Pregnant women	HCV
Pirillo	2007	No	Yes	No	Yes	Yes	Yes	Yes	Yes	Yes	Yes	Low risk of bias	Pregnant women	HBV
Pirillo	2007	No	Yes	No	Yes	Yes	Yes	Yes	Yes	Yes	Yes	Low risk of bias	Pregnant women	HBV
Pirillo	2007	No	Yes	No	Yes	Yes	Yes	Yes	Yes	Yes	Yes	Low risk of bias	Pregnant women	HCV
Powell	2015	No	Yes	No	Yes	Yes	Yes	Yes	Yes	Yes	Yes	Low risk of bias	General population	HBV
Price	2017	No	Yes	No	Yes	Yes	No	Yes	Yes	Yes	Yes	Low risk of bias	General population	HBV
Price	2017	No	Yes	No	Yes	Yes	No	Yes	Yes	Yes	Yes	Low risk of bias	General population	HBV
Ramírez Me	2022	No	Yes	No	Yes	No	No	Yes	Yes	No	Yes	Moderate risk of bias	General population	HBV
Ramírez Me	2022	No	Yes	No	Yes	No	No	Yes	Yes	No	Yes	Moderate risk of bias	General population	HBV
Rebbani	2013	No	Yes	No	Yes	Yes	Yes	Yes	Yes	Yes	Yes	Low risk of bias	General population	HBV
Rebbani	2013	No	Yes	No	Yes	Yes	Yes	Yes	Yes	Yes	Yes	Low risk of bias	General population	HCV
Rebbani	2013	No	Yes	No	Yes	Yes	Yes	Yes	Yes	Yes	Yes	Low risk of bias	General population	HBV
Rouet	2015	No	Yes	No	Yes	Yes	Yes	Yes	Yes	Yes	Yes	Low risk of bias	General population	HCV
Rouet	2008	No	Yes	No	Yes	Yes	Yes	Yes	Yes	Yes	Yes	Low risk of bias	General population	CFR
Rouet	2008	No	Yes	No	Yes	Yes	Yes	Yes	Yes	Yes	Yes	Low risk of bias	General population	HCV
Rouet	2008	No	Yes	No	Yes	Yes	Yes	Yes	Yes	Yes	Yes	Low risk of bias	General population	HBV
Rouet	2008	No	Yes	No	Yes	Yes	Yes	Yes	Yes	Yes	Yes	Low risk of bias	General population	HBV
Rouet	2004	No	Yes	No	Yes	Yes	Yes	Yes	Yes	Yes	Yes	Low risk of bias	Pregnant women	HBV
Rouet	2004	No	Yes	No	Yes	Yes	Yes	Yes	Yes	Yes	Yes	Low risk of bias	Pregnant women	HCV
Rusine	2013	No	Yes	No	Yes	Yes	Yes	Yes	Yes	Yes	Yes	Low risk of bias	General population	HCV
Rusine	2013	No	Yes	No	Yes	Yes	Yes	Yes	Yes	Yes	Yes	Low risk of bias	General population	HBV
Sadoh	2011	No	Yes	No	Yes	Yes	Unclear	Yes	Yes	No	Yes	Moderate risk of bias	General population	HCV
Sadoh	2011	No	Yes	No	Yes	Yes	Unclear	Yes	Yes	No	Yes	Moderate risk of bias	General population	HBV
Sagoe	2012	No	Yes	No	Yes	Yes	Yes	Yes	Yes	No	Yes	Low risk of bias	General population	HBV
Sagoe	2012	No	Yes	No	Yes	Yes	Yes	Yes	Yes	No	Yes	Low risk of bias	General population	HCV
Sagoe	2012	No	Yes	No	Yes	Yes	Yes	Yes	Yes	No	Yes	Low risk of bias	General population	HBV
Salpini	2016	No	Yes	No	Yes	Yes	Yes	Yes	Yes	No	Yes	Low risk of bias	General population	HCV
Salpini	2016	No	Yes	No	Yes	Yes	Yes	Yes	Yes	No	Yes	Low risk of bias	General population	HBV
Salu	2018	No	Yes	No	Yes	Yes	Yes	Yes	Yes	Yes	Yes	Low risk of bias	General population	HCV
Salu	2018	No	Yes	No	Yes	Yes	Yes	Yes	Yes	Yes	Yes	Low risk of bias	General population	HBV
Salyani	2021	No	Yes	No	Yes	Yes	Yes	Yes	Yes	No	Yes	Low risk of bias	General population	HBV
Shimelis	2017	No	Yes	Yes	Yes	Yes	Unclear	Yes	Yes	No	Yes	Low risk of bias	General population	HCV
Shimelis	2017	No	Yes	Yes	Yes	Yes	Unclear	Yes	Yes	No	Yes	Low risk of bias	General population	HBV and HCV
Shimelis	2017	No	Yes	Yes	Yes	Yes	Unclear	Yes	Yes	No	Yes	Low risk of bias	General population	HBV
Shimelis	2017	No	Yes	Yes	Yes	Yes	Unclear	Yes	Yes	No	Yes	Low risk of bias	General population	HBV
Simani	2009	No	Yes	No	Yes	Yes	Unclear	Yes	Yes	Yes	Yes	Low risk of bias	General population	HBV
Simpore	2006	No	Yes	No	Yes	Yes	Unclear	Yes	Yes	Yes	Yes	Low risk of bias	Pregnant women	HCV
Simpore	2006	No	Yes	No	Yes	Yes	Unclear	Yes	Yes	Yes	Yes	Low risk of bias	Pregnant women	HBV and HCV
Simpore	2006	No	Yes	No	Yes	Yes	Unclear	Yes	Yes	Yes	Yes	Low risk of bias	Pregnant women	HBV
Sonderup	2015	No	Yes	No	Yes	Yes	Unclear	Yes	Yes	Yes	Yes	Low risk of bias	General population	HCV
Sonderup	2015	No	Yes	No	Yes	Yes	Unclear	Yes	Yes	Yes	Yes	Low risk of bias	General population	HBV
Sonderup	2015	No	Yes	No	Yes	Yes	Unclear	Yes	Yes	Yes	Yes	Low risk of bias	General population	HCV
Stabinski	2011	No	Yes	No	Yes	Yes	Unclear	Yes	Yes	Unclear	Yes	Moderate risk of bias	General population	HBV
Tamo	2017	No	Yes	No	Yes	Yes	No	Yes	Yes	No	Yes	Moderate risk of bias	General population	HCV
Telatela	2007	No	Yes	No	Yes	Yes	Unclear	Yes	Yes	No	Yes	Moderate risk of bias	General population	HCV
Telatela	2007	No	Yes	No	Yes	Yes	Unclear	Yes	Yes	No	Yes	Moderate risk of bias	General population	HBV
Torpey	2021	No	Yes	No	Yes	Yes	No	Yes	Yes	No	Yes	Moderate risk of bias	General population	HCV
Torpey	2021	No	Yes	No	Yes	Yes	No	Yes	Yes	No	Yes	Moderate risk of bias	General population	HCV
Toyé	2020	No	Yes	No	Yes	Yes	Unclear	Yes	Yes	No	Yes	Moderate risk of bias	General population	HBV
Tremeau-B	2012	No	Yes	No	Yes	Yes	Unclear	Yes	Yes	No	Yes	Moderate risk of bias	General population	HCV
Tremeau-B	2012	No	Yes	No	Yes	Yes	Unclear	Yes	Yes	No	Yes	Moderate risk of bias	General population	HBV and HCV
Tremeau-B	2012	No	Yes	No	Yes	Yes	Unclear	Yes	Yes	No	Yes	Moderate risk of bias	General population	HBV
Umutesi	2017	Yes	Yes	No	Yes	Yes	No	Yes	Yes	No	Yes	Low risk of bias	General population	HBV and HCV
Umutesi	2017	Yes	Yes	No	Yes	Yes	No	Yes	Yes	No	Yes	Low risk of bias	General population	HBV
Umutesi	2017	Yes	Yes	No	Yes	Yes	No	Yes	Yes	No	Yes	Low risk of bias	General population	HCV
Uneke	2005	No	Yes	No	Yes	Yes	Unclear	Yes	Yes	Yes	Yes	Low risk of bias	Blood donors	HBV
Utoo	2012	No	Yes	No	Yes	Yes	Unclear	Yes	Yes	No	Yes	Moderate risk of bias	General population	HBV
Varo	2016	No	Yes	Yes	Yes	Yes	Unclear	Yes	Yes	Yes	Yes	Low risk of bias	General population	HBV
Velen	2016	No	Yes	No	Yes	Yes	Unclear	Yes	Yes	Yes	Yes	Low risk of bias	General population	HBV
Walusansa	2009	No	Yes	No	Yes	Yes	Unclear	Yes	Yes	No	Yes	Moderate risk of bias	General population	HCV
Wandeler	2016	No	Yes	No	Yes	Yes	Unclear	Yes	Yes	Yes	Yes	Low risk of bias	General population	HCV
Wandeler	2016	No	Yes	No	Yes	Yes	Unclear	Yes	Yes	Yes	Yes	Low risk of bias	General population	HBV
Wandeler	2016	No	Yes	No	Yes	Yes	Unclear	Yes	Yes	Yes	Yes	Low risk of bias	General population	HCV
Wandeler	2016	No	Yes	No	Yes	Yes	Unclear	Yes	Yes	Unclear	Yes	Moderate risk of bias	General population	HBV
Wandeler	2016	No	Yes	No	Yes	Yes	Unclear	Yes	Yes	Yes	Yes	Low risk of bias	General population	HBV
Webale	2015	No	Yes	No	Yes	Yes	Unclear	Yes	Yes	Unclear	Yes	Moderate risk of bias	General population	HBV
Webale	2015	No	Yes	No	Yes	Yes	Unclear	Yes	Yes	Unclear	Yes	Moderate risk of bias	Injecting drug users	HBV
Webale	2015	No	Yes	No	Yes	Yes	Unclear	Yes	Yes	Unclear	Yes	Moderate risk of bias	General population	HBV
Webale	2015	No	Yes	No	Yes	Yes	Unclear	Yes	Yes	Unclear	Yes	Moderate risk of bias	Injecting drug users	HBV
Wekesa	2020	No	Yes	No	Yes	Yes	Unclear	Yes	Yes	Yes	Yes	Low risk of bias	General population	HBV
Wekesa	2020	No	Yes	No	Yes	Yes	Unclear	Yes	Yes	Yes	Yes	Low risk of bias	General population	HCV
Weldemhre	2016	No	Yes	No	Yes	Yes	Yes	Yes	Yes	No	Yes	Low risk of bias	General population	HBV
Wondimene	2013	No	Yes	No	Yes	Yes	Unclear	Yes	Yes	No	Yes	Moderate risk of bias	General population	HCV
Wondimene	2013	No	Yes	No	Yes	Yes	Unclear	Yes	Yes	No	Yes	Moderate risk of bias	General population	HBV and HCV
Wondimene	2013	No	Yes	No	Yes	Yes	Unclear	Yes	Yes	No	Yes	Moderate risk of bias	General population	HBV
Ya'aba	2015	No	Yes	Yes	Yes	Yes	Unclear	Yes	Yes	No	Yes	Low risk of bias	General population	HCV
Ya'aba	2017	No	Yes	No	Yes	Yes	Unclear	Yes	Yes	No	Yes	Moderate risk of bias	General population	HCV
Yakubu	2021	No	Yes	No	Yes	Yes	No	Yes	Yes	Yes	Yes	Low risk of bias	General population	HCV
Yendewa	2021	No	Yes	No	Yes	Yes	Unclear	Yes	Yes	No	Yes	Moderate risk of bias	Pregnant women	HCV
Yendewa	2021	No	Yes	No	Yes	Yes	Unclear	Yes	Yes	No	Yes	Moderate risk of bias	Pregnant women	HBV
Yendewa	2021	No	Yes	No	Yes	Yes	Unclear	Yes	Yes	No	Yes	Moderate risk of bias	Pregnant women	HBV
Yendewa	2021	No	Yes	No	Yes	Yes	Unclear	Yes	Yes	No	Yes	Moderate risk of bias	Pregnant women	HCV
Yendewa	2021	No	Yes	No	Yes	Yes	Unclear	Yes	Yes	No	Yes	Moderate risk of bias	Pregnant women	HCV
Yendewa	2021	No	Yes	No	Yes	Yes	Unclear	Yes	Yes	No	Yes	Moderate risk of bias	Pregnant women	HBV
Yendewa	2021	No	Yes	No	Yes	Yes	Unclear	Yes	Yes	No	Yes	Moderate risk of bias	Pregnant women	HBV
Yendewa	2021	No	Yes	No	Yes	Yes	Unclear	Yes	Yes	No	Yes	Moderate risk of bias	Pregnant women	HCV
Yendewa	2021	No	Yes	No	Yes	Yes	Unclear	Yes	Yes	No	Yes	Moderate risk of bias	Pregnant women	HCV
Yendewa	2021	No	Yes	No	Yes	Yes	Unclear	Yes	Yes	No	Yes	Moderate risk of bias	Pregnant women	HBV
Yendewa	2021	No	Yes	No	Yes	Yes	Unclear	Yes	Yes	No	Yes	Moderate risk of bias	Pregnant women	HBV
Yendewa	2021	No	Yes	No	Yes	Yes	Unclear	Yes	Yes	No	Yes	Moderate risk of bias	Pregnant women	HCV
Yendewa	2019	No	Yes	No	Yes	Yes	Unclear	Yes	Yes	No	Yes	Moderate risk of bias	General population	HCV
Yendewa	2019	No	Yes	No	Yes	Yes	Unclear	Yes	Yes	No	Yes	Moderate risk of bias	General population	HBV
Zoufaly	2012	No	Yes	No	Yes	Yes	Unclear	Yes	Yes	Yes	Yes	Low risk of bias	General population	HBV
Abreha	2011	No	Yes	No	Yes	Yes	No	Yes	Yes	No	Yes	Moderate risk of bias	General population	HCV
Adoga	2009	No	Yes	No	Yes	Yes	Yes	Yes	Yes	No	Yes	Low risk of bias	Prisoners	HBV
Adoga	2009	No	Yes	No	Yes	Yes	Yes	Yes	Yes	No	Yes	Low risk of bias	Prisoners	HCV
Agaba	2014	No	Yes	No	Yes	Yes	No	Yes	Yes	Yes	Yes	Low risk of bias	General population	HBV

Agaba	2014	No	Yes	No	Yes	Yes	No	Yes	Yes	Yes	Yes	Low risk of bias	General population	HCV
Ahmed	1998	No	Yes	No	Yes	Yes	No	Yes	Yes	Yes	Yes	Low risk of bias	Pregnant women	HBV
Ahmed	1998	No	Yes	No	Yes	Yes	No	Yes	Yes	Yes	Yes	Low risk of bias	Pregnant women	HBV
Ahmed	1998	No	Yes	No	Yes	Yes	No	Yes	Yes	Yes	Yes	Low risk of bias	Pregnant women	HCV
Alidjinou	2014	No	Yes	No	Yes	Yes	No	Yes	Yes	Unclear	Yes	Moderate risk of bias	Blood donors	HCV
Anigilaje	2013	No	Yes	No	Yes	Yes	No	Yes	Yes	Yes	Yes	Low risk of bias	General population	HBV
Anigilaje	2013	No	Yes	No	Yes	Yes	No	Yes	Yes	Yes	Yes	Low risk of bias	General population	HCV
Anigilaje	2013	No	Yes	No	Yes	Yes	No	Yes	Yes	Yes	Yes	Low risk of bias	General population	HBV and HCV
Atina	2004	No	Yes	No	Yes	Yes	No	Yes	Yes	Unclear	Yes	Moderate risk of bias	General population	HCV
Balew	2014	No	Yes	Yes	Yes	Yes	Yes	Yes	Yes	No	Yes	Low risk of bias	General population	HBV
Balew	2014	No	Yes	Yes	Yes	Yes	Yes	Yes	Yes	No	Yes	Low risk of bias	General population	HCV
Balew	2014	No	Yes	Yes	Yes	Yes	Yes	Yes	Yes	No	Yes	Low risk of bias	General population	HBV and HCV
Benjelloun	1996	No	Yes	No	Yes	Yes	No	Yes	Yes	Unclear	Yes	Moderate risk of bias	General population	HCV
Bouare	2012	No	Yes	No	Yes	Yes	No	Yes	Yes	Unclear	Yes	Moderate risk of bias	General population	HCV
Bouare	2012	No	Yes	No	Yes	Yes	No	Yes	Yes	Unclear	Yes	Moderate risk of bias	Pregnant women	HCV
Bowring	2013	No	Yes	Yes	Yes	Yes	No	Yes	Yes	No	Yes	Low risk of bias	General population	HCV
Bowring	2013	No	Yes	Yes	Yes	Yes	No	Yes	Yes	No	Yes	Low risk of bias	Injecting drug users	HCV
Brandful	1999	No	Yes	No	Yes	Yes	No	Unclear	Yes	Yes	Yes	Moderate risk of bias	General population	HCV
Brandful	1999	No	Yes	No	Yes	Yes	No	Unclear	Yes	Yes	Yes	Moderate risk of bias	General population	HBV
Buseri	2009	No	Yes	No	Yes	Yes	No	Yes	Yes	No	Yes	Moderate risk of bias	Blood donors	HCV
Buseri	2009	No	Yes	No	Yes	Yes	No	Yes	Yes	No	Yes	Moderate risk of bias	Blood donors	HBV
Chakraborty	2003	No	Yes	No	Yes	Yes	No	Yes	Yes	Unclear	Yes	Moderate risk of bias	General population	HBV
Chakraborty	2003	No	Yes	No	Yes	Yes	No	Yes	Yes	Unclear	Yes	Moderate risk of bias	General population	HCV
Collenberg	2006	No	Yes	No	Yes	Yes	No	Yes	Yes	No	Yes	Moderate risk of bias	General population	HBV
Croce	2007	No	Yes	No	Yes	Yes	No	Yes	Yes	No	Yes	Moderate risk of bias	General population	HBV
Cunha	2007	No	Yes	No	Yes	Yes	No	Yes	Yes	No	Yes	Moderate risk of bias	Blood donors	HCV
Cunha	2007	No	Yes	No	Yes	Yes	No	Yes	Yes	No	Yes	Moderate risk of bias	Blood donors	HBV
Dahoma	2011	No	Yes	No	Yes	Yes	No	Yes	Yes	No	Yes	Moderate risk of bias	General population	HCV
Dahoma	2011	No	Yes	No	Yes	Yes	No	Yes	Yes	No	Yes	Moderate risk of bias	Men who have sex with men	HBV
Diop-Ndiaye	2008	No	Yes	Yes	Yes	Yes	No	Yes	Yes	Yes	Yes	Low risk of bias	General population	HCV
Diop-Ndiaye	2008	No	Yes	Yes	Yes	Yes	No	Yes	Yes	Yes	Yes	Low risk of bias	General population	HBV and HCV
Diop-Ndiaye	2008	No	Yes	Yes	Yes	Yes	No	Yes	Yes	Yes	Yes	Low risk of bias	General population	HBV
Diro	2008	No	Yes	No	Yes	Yes	No	Yes	Yes	No	Yes	Moderate risk of bias	Blood donors	HCV
Diro	2008	No	Yes	No	Yes	Yes	No	Yes	Yes	No	Yes	Moderate risk of bias	Blood donors	HBV and HCV
Diro	2008	No	Yes	No	Yes	Yes	No	Yes	Yes	No	Yes	Moderate risk of bias	Blood donors	HBV
du Plessis	1999	No	Yes	Yes	No	Yes	No	Yes	Yes	Unclear	Yes	Moderate risk of bias	Deceased people	HCV
du Plessis	1999	No	Yes	Yes	No	Yes	No	Yes	Yes	Unclear	Yes	Moderate risk of bias	Deceased people	HBV
Ezechi	2014	No	Yes	No	Yes	Yes	Yes	Unclear	Yes	Yes	Yes	Low risk of bias	Pregnant women	HCV
Ezechi	2014	No	Yes	No	Yes	Yes	Yes	Unclear	Yes	Yes	Yes	Low risk of bias	Pregnant women	HBV and HCV
Ezechi	2014	No	Yes	No	Yes	Yes	Yes	Unclear	Yes	Yes	Yes	Low risk of bias	Pregnant women	HBV
Ladep	2013	No	Yes	No	Yes	Yes	No	Yes	Yes	Yes	Yes	Low risk of bias	General population	HCV
Ladep	2013	No	Yes	No	Yes	Yes	No	Yes	Yes	Yes	Yes	Low risk of bias	General population	HBV and HCV
Ladep	2013	No	Yes	No	Yes	Yes	No	Yes	Yes	Yes	Yes	Low risk of bias	General population	HBV
Harania	2008	No	Yes	No	Yes	Yes	No	Yes	Yes	Unclear	Yes	Moderate risk of bias	General population	HCV
Harania	2008	No	Yes	No	Yes	Yes	No	Yes	Yes	Unclear	Yes	Moderate risk of bias	General population	HBV and HCV
Harania	2008	No	Yes	No	Yes	Yes	No	Yes	Yes	Unclear	Yes	Moderate risk of bias	General population	HBV
Hoffmann	2007	No	Yes	Yes	Yes	Yes	No	Yes	Yes	Unclear	Yes	Low risk of bias	General population	HCV
Hoffmann	2007	No	Yes	Yes	Yes	Yes	No	Yes	Yes	Unclear	Yes	Low risk of bias	General population	HBV
Hoffmann	2008	No	Yes	No	Yes	Yes	Yes	Unclear	Yes	Yes	Yes	Low risk of bias	General population	HCV
Hoffmann	2008	No	Yes	No	Yes	Yes	Yes	Yes	Yes	Yes	Yes	Low risk of bias	General population	HBV
Inyama	2005	No	Yes	No	Yes	Yes	No	Yes	Yes	Yes	Yes	Low risk of bias	General population	HCV
Kallestrup	2003	No	Yes	No	Yes	Yes	Unclear	Yes	Yes	Unclear	Yes	Moderate risk of bias	General population	HCV
Kania	2009	No	Yes	Yes	Yes	Yes	Yes	Yes	Yes	No	Yes	Low risk of bias	Blood donors	HCV
Kania	2009	No	Yes	Yes	Yes	Yes	Yes	Yes	Yes	No	Yes	Low risk of bias	Blood donors	HBV
Kapembwa	2011	No	Yes	No	Yes	Yes	Yes	Yes	Yes	No	Yes	Low risk of bias	General population	HCV
Kapembwa	2011	No	Yes	No	Yes	Yes	Yes	Yes	Yes	No	Yes	Low risk of bias	General population	HBV and HCV
Kapembwa	2011	No	Yes	No	Yes	Yes	Yes	Yes	Yes	No	Yes	Low risk of bias	General population	HBV
Kilani	2007	No	Yes	No	Yes	Yes	No	Yes	Yes	Unclear	Yes	Moderate risk of bias	General population	HCV
Kubio	2012	No	Yes	No	Yes	Yes	No	Unclear	Yes	Unclear	Yes	Moderate risk of bias	Blood donors	HCV
Kubio	2012	No	Yes	No	Yes	Yes	No	Unclear	Yes	Unclear	Yes	Moderate risk of bias	Blood donors	HBV and HCV
Kubio	2012	No	Yes	No	Yes	Yes	No	Unclear	Yes	Unclear	Yes	Moderate risk of bias	Blood donors	HBV
Lassey	2004	No	Yes	Yes	Yes	Yes	Unclear	Yes	Yes	No	Yes	Low risk of bias	Pregnant women	HCV
Laurent	2007	No	Yes	No	Yes	Yes	No	Yes	Yes	No	Yes	Moderate risk of bias	General population	HCV
Laurent	2001	No	Yes	Yes	Yes	Yes	No	Yes	Yes	Unclear	Yes	Low risk of bias	Pregnant women	HCV
Laurent	2001	No	Yes	Yes	Yes	Yes	No	Yes	Yes	Unclear	Yes	Low risk of bias	Commercial sex workers	HCV
Lepretre	2015	No	Yes	Yes	Yes	Yes	Unclear	Yes	Yes	No	Yes	Low risk of bias	Injecting drug users	HBV
Lepretre	2015	No	Yes	Yes	Yes	Yes	Unclear	Yes	Yes	No	Yes	Low risk of bias	Injecting drug users	HCV
Mabayoje	2007	No	Yes	No	Yes	Yes	No	Yes	Yes	Yes	Yes	Low risk of bias	Blood donors	HCV
Mabayoje	2007	No	Yes	No	Yes	Yes	No	Yes	Yes	Yes	Yes	Low risk of bias	Blood donors	HBV
Mabayoje	2013	No	Yes	No	Yes	Yes	No	Yes	Yes	No	Yes	Moderate risk of bias	General population	HCV
Matee	2006	No	Yes	No	Yes	Yes	No	Yes	Yes	Yes	Yes	Low risk of bias	Blood donors	HBV
Mavenyeng	2014	No	Yes	No	Yes	Yes	No	Yes	Yes	Yes	Yes	Low risk of bias	Blood donors	HCV
Mavenyeng	2014	No	Yes	No	Yes	Yes	No	Yes	Yes	Yes	Yes	Low risk of bias	Blood donors	HBV
Mayaki	2013	No	Yes	No	Yes	Yes	No	Yes	Yes	Unclear	Yes	Moderate risk of bias	Blood donors	HBV and HCV
Mayaki	2013	No	Yes	No	Yes	Yes	No	Yes	Yes	Unclear	Yes	Moderate risk of bias	Blood donors	HBV
Mbanya	2003	No	Yes	No	Yes	Yes	No	Yes	Yes	No	Yes	Moderate risk of bias	Blood donors	HCV
Mbanya	2003	No	Yes	No	Yes	Yes	No	Yes	Yes	No	Yes	Moderate risk of bias	Blood donors	HBV
Mboto	2009	No	Yes	No	Yes	Yes	No	Yes	Yes	No	Yes	Moderate risk of bias	General population	HCV
Mirzoyan	1999	No	Yes	Yes	Yes	Yes	Unclear	Unclear	Yes	No	Yes	Moderate risk of bias	Injecting drug users	HCV
Mirzoyan	1999	No	Yes	Yes	Yes	Yes	Unclear	Unclear	Yes	No	Yes	Moderate risk of bias	Injecting drug users	HBV
Moore	2010	No	Yes	No	Yes	Yes	No	Yes	Yes	Unclear	Yes	Moderate risk of bias	General population	HCV
Moore	2010	No	Yes	No	Yes	Yes	No	Yes	Yes	Unclear	Yes	Moderate risk of bias	General population	HBV and HCV
Moore	2010	No	Yes	No	Yes	Yes	No	Yes	Yes	Unclear	Yes	Moderate risk of bias	General population	HBV
Msuya	2006	No	Yes	No	Yes	Yes	No	Yes	Yes	No	Yes	Moderate risk of bias	General population	HCV
Msuya	2006	No	Yes	No	Yes	Yes	No	Yes	Yes	No	Yes	Moderate risk of bias	General population	HBV
Mugusi S	2012	No	Yes	No	Yes	Yes	No	Unclear	Yes	Yes	Yes	Moderate risk of bias	General population	HCV
Mugusi S	2012	No	Yes	No	Yes	Yes	No	Unclear	Yes	Yes	Yes	Moderate risk of bias	General population	HBV
Mullis	2013	No	Yes	No	Yes	Yes	No	Yes	Yes	Unclear	Yes	Moderate risk of bias	General population	HCV
Mulu	2013	No	Yes	No	Yes	Yes	No	Yes	Yes	No	Yes	Moderate risk of bias	General population	HCV
Mulu	2013	No	Yes	No	Yes	Yes	No	Yes	Yes	No	Yes	Moderate risk of bias	General population	HBV and HCV
Mulu	2013	No	Yes	No	Yes	Yes	No	Yes	Yes	No	Yes	Moderate risk of bias	General population	HBV
Muro	2013	No	Yes	No	Yes	Yes	No	Yes	Yes	Yes	Yes	Low risk of bias	General population	HCV

Muro	2013	No	Yes	No	Yes	Yes	No	Yes	Yes	Yes	Yes	Low risk of bias	General population	HBV
Mwatelah	2015	No	Yes	No	Yes	Yes	No	Yes	Yes	Unclear	Yes	Moderate risk of bias	Injecting drug users	HCV
Nagalo	2011	No	Yes	No	Yes	Yes	No	Yes	Yes	Yes	Yes	Low risk of bias	Blood donors	HCV
Nagalo	2011	No	Yes	No	Yes	Yes	No	Yes	Yes	Yes	Yes	Low risk of bias	Blood donors	HBV and HCV
Nagalo	2011	No	Yes	No	Yes	Yes	No	Yes	Yes	Yes	Yes	Low risk of bias	Blood donors	HBV
Nagalo	2012	No	Yes	No	Yes	Yes	No	Yes	Yes	Yes	Yes	Low risk of bias	Blood donors	HCV
Nagalo	2012	No	Yes	No	Yes	Yes	No	Yes	Yes	Yes	Yes	Low risk of bias	Blood donors	HBV
Nakwagala	2002	No	Yes	No	Yes	Yes	No	Yes	Yes	No	Yes	Moderate risk of bias	General population	HBV
Nakwagala	2002	No	Yes	No	Yes	Yes	No	Yes	Yes	No	Yes	Moderate risk of bias	General population	HBV
Noubiap	2013	No	Yes	No	Yes	Yes	No	Yes	Yes	No	Yes	Moderate risk of bias	Blood donors	HCV
Noubiap	2013	No	Yes	No	Yes	Yes	No	Yes	Yes	No	Yes	Moderate risk of bias	Blood donors	HBV
Ntagirabiri	2012	No	Yes	No	Yes	Yes	No	Yes	Yes	Yes	Yes	Low risk of bias	General population	HCV
Nyirenda	2008	No	Yes	No	Yes	Yes	No	Yes	Yes	Unclear	Yes	Moderate risk of bias	General population	HBV
Nyirenda	2008	No	Yes	No	Yes	Yes	No	Yes	Yes	Unclear	Yes	Moderate risk of bias	General population	HBV and HCV
Nyirenda	2008	No	Yes	No	Yes	Yes	No	Yes	Yes	Unclear	Yes	Moderate risk of bias	General population	HCV
Obienu	2011	No	Yes	No	Yes	Yes	No	Yes	Yes	Yes	Yes	Low risk of bias	General population	HCV
Odama	2004	No	Yes	No	Yes	Yes	No	Yes	Yes	Unclear	Yes	Moderate risk of bias	General population	HCV
Olokoba	2008	No	Yes	No	Yes	Yes	No	Yes	Yes	Yes	Yes	Low risk of bias	General population	HCV
Omosigho	2011	No	Yes	No	Yes	Yes	No	Yes	Yes	Yes	Yes	Low risk of bias	General population	HCV
O'Reilly	2011	No	Yes	Yes	Yes	Yes	No	Yes	Yes	No	Yes	Low risk of bias	General population	HCV
Oronsaye	2004	No	Yes	No	Yes	Yes	No	Yes	Yes	Yes	Yes	Low risk of bias	Blood donors	HBV
Oshitani	1995	No	Yes	No	Yes	Yes	No	Yes	Yes	Unclear	Yes	Moderate risk of bias	General population	HCV
Otuonye	2002	No	Yes	No	Yes	Yes	No	Unclear	Yes	No	Yes	Moderate risk of bias	General population	HBV
Pawlotsky	1995	No	Yes	No	Yes	Yes	No	Yes	Yes	Unclear	Yes	Moderate risk of bias	General population	HCV
Pawlotsky	1995	No	Yes	No	Yes	Yes	No	Yes	Yes	Unclear	Yes	Moderate risk of bias	General population	HBV
Plamondon	2007	No	Yes	No	Yes	Yes	No	Yes	Yes	No	Yes	Moderate risk of bias	General population	HCV
Rabenau	2010	No	Yes	No	Yes	Yes	No	Yes	Yes	No	No	Moderate risk of bias	General population	HCV
Rabenau	2010	No	Yes	No	Yes	Yes	No	Yes	Yes	No	No	Moderate risk of bias	General population	HBV
Rahlenbeck	1997	No	Yes	No	Yes	Yes	No	Yes	Yes	No	Yes	Moderate risk of bias	Blood donors	HBV
Seremba	2010	No	Yes	No	Yes	Yes	No	Yes	Yes	No	Yes	Moderate risk of bias	General population	HCV
Shimelis	2008	No	Yes	No	Yes	Yes	No	Yes	Yes	No	Yes	Moderate risk of bias	General population	HBV
Simpore	2004	No	Yes	No	Yes	Yes	No	Yes	Yes	Unclear	Yes	Moderate risk of bias	General population	HBV
Simpore	2004	No	Yes	No	Yes	Yes	No	Yes	Yes	Unclear	Yes	Moderate risk of bias	Pregnant women	HBV
Simpore	2005	No	Yes	Yes	Yes	Yes	No	Yes	Yes	No	Yes	Low risk of bias	Pregnant women	HCV
Simpore	2005	No	Yes	Yes	Yes	Yes	No	Yes	Yes	No	Yes	Low risk of bias	Pregnant women	HCV
Simpore	2006	No	Yes	No	Yes	Yes	No	Yes	Yes	Yes	Yes	Low risk of bias	Pregnant women	HCV
Simpore	2006	No	Yes	No	Yes	Yes	No	Yes	Yes	Yes	Yes	Low risk of bias	Pregnant women	HBV
Soni	1993	No	Yes	No	Yes	Yes	No	Yes	Yes	No	Yes	Moderate risk of bias	Men who have sex with men	HCV
Stokx	2011	No	Yes	No	Yes	Yes	No	Yes	Yes	No	Yes	Moderate risk of bias	Blood donors	HBV
Sutcliffe	2002	No	Yes	No	Yes	Yes	No	Yes	Yes	Unclear	No	Moderate risk of bias	General population	HBV
Sutcliffe	2002	No	Yes	No	Yes	Yes	No	Yes	Yes	Unclear	No	Moderate risk of bias	General population	HCV
Taye	2013	No	Yes	No	Yes	Yes	No	Yes	Yes	No	No	Moderate risk of bias	General population	HCV
Tess	2000	No	Yes	Yes	Yes	Yes	No	Yes	Yes	Unclear	Yes	Low risk of bias	General population	HCV
Tessema	2010	No	Yes	No	Yes	Yes	No	Yes	Yes	Yes	Yes	Low risk of bias	Blood donors	HCV
Tessema	2010	No	Yes	No	Yes	Yes	No	Yes	Yes	Yes	Yes	Low risk of bias	Blood donors	HBV
Tounkara	2009	No	Yes	No	Yes	Yes	No	Yes	Yes	No	Yes	Moderate risk of bias	Blood donors	HBV
Waddell	2006	No	Yes	No	Yes	Yes	No	Yes	Yes	Unclear	Yes	Moderate risk of bias	Blood donors	HCV
Wester	2006	No	Yes	No	Yes	Yes	No	Yes	Yes	No	Yes	Moderate risk of bias	General population	HCV
Wester	2006	No	Yes	No	Yes	Yes	No	Yes	Yes	No	Yes	Moderate risk of bias	General population	HBV
Wester	2006	No	Yes	No	Yes	Yes	No	Yes	Yes	No	Yes	Moderate risk of bias	General population	HBV
Xie	2015	No	Yes	No	Yes	Yes	No	Yes	Yes	Yes	Yes	Low risk of bias	Blood donors	HCV
Xie	2015	No	Yes	No	Yes	Yes	No	Yes	Yes	Yes	Yes	Low risk of bias	Blood donors	HBV
Ya'Aba	2009	No	Yes	No	Yes	Yes	No	Yes	Yes	Yes	Yes	Low risk of bias	Pregnant women	HCV
Yimer	2014	No	Yes	No	Yes	Yes	No	Unclear	Yes	Yes	Yes	Moderate risk of bias	General population	HCV
Yimer	2014	No	Yes	No	Yes	Yes	No	Unclear	Yes	Yes	Yes	Moderate risk of bias	General population	HBV
Zeba	2011	No	Yes	No	Yes	Yes	No	Yes	Yes	No	Yes	Moderate risk of bias	Pregnant women	HCV
Zeba	2011	No	Yes	No	Yes	Yes	No	Yes	Yes	No	Yes	Moderate risk of bias	Pregnant women	HCV
