# Supplementary material for: Epidemiology of hepatitis B virus and/or hepatitis C virus infections among people living with human immunodeficiency virus in Africa: A systematic review and meta-analysis
Source: PLoS One. 2022 May 31;17(5):e0269250. doi: 10.1371/journal.pone.0269250 (PMC9154112; doi:10.1371/journal.pone.0269250)
Supplement: S8 Table — (PDF) [file pone.0269250.s009.pdf]

S8 Table. Subgroup analyses of case fatality rate and prevalence of hepatitis B and C virus in people living with HIV in Africa.

	**Prevalence. % (95%CI)**	**95% Prediction interval**	**N Studies**	**N Participants**	**H (95%CI)**	**I² (95%CI)**	**P heterogeneity**	**P difference subtypes**
**HBV case fatality rate in PLHIV**								
**Study Design**								0.451
Clinical Trial (Baseline data)	7.7 [4-12.5]	NA	1	155	NA	NA	1	
Cohort (Baseline data)	3.7 [0-11.2]	[0-51.4]	4	2071	2.2 [1.4-3.7]	80.2 [47.8-92.5]	0.002	
**Sampling**								0.451
Non probabilistic	3.7 [0-11.2]	[0-51.4]	4	2071	2.2 [1.4-3.7]	80.2 [47.8-92.5]	0.002	
Probabilistic	7.7 [4-12.5]	NA	1	155	NA	NA	1	
**Timing of samples collection**								0.001
Prospetively	2.4 [0.3-5.9]	[0-22.9]	4	2195	1.8 [1.1-3.1]	69.6 [12.5-89.4]	0.02	
Retrospectively	22.6 [9.3-39.2]	NA	1	31	NA	NA	1	
**Countries**								0.22
Gambia	1.3 [0-5.4]	NA	1	79	NA	NA	1	
Ivory Coast	13.2 [2-30.9]	NA	2	186	2.2 [1.1-4.6]	80 [13.8-95.3]	0.026	
Kenya	0 [0-15.1]	NA	1	11	NA	NA	1	
Nigeria	2.5 [1.8-3.2]	NA	1	1950	NA	NA	1	
**UNAIDS Region**								0.513
East and Southern	0 [0-15.1]	NA	1	11	NA	NA	1	
West and Central	5.6 [1.3-12.2]	[0-47.7]	4	2215	2.8 [1.8-4.4]	87.5 [70.3-94.8]	< 0.001	
**UNSD Region**								0.513
Eastern Africa	0 [0-15.1]	NA	1	11	NA	NA	1	
West Africa	5.6 [1.3-12.2]	[0-47.7]	4	2215	2.8 [1.8-4.4]	87.5 [70.3-94.8]	< 0.001	
**Country income level**								0.125
Low-income economies	1.3 [0-5.4]	NA	1	79	NA	NA	1	
Lower-middle income economies	5.9 [0.6-14.7]	[0-58.1]	4	2147	2.8 [1.8-4.3]	87.3 [69.7-94.7]	< 0.001	
**Age range**								0.002
Adults	3.4 [0.8-7.4]	[0-88.5]	3	2184	2.2 [1.2-4]	79.7 [35.6-93.6]	0.007	
Children	22.6 [9.3-39.2]	NA	1	31	NA	NA	1	
**HBV and/or HCV_diagnostic_method**								0.22
ADVIA Centaur chemiluminometric immunoassay system	0 [0-15.1]	NA	1	11	NA	NA	1	

	Prevalence. % (95%CI)	95% Prediction interval	N Studies	N Participants	H (95%CI)	I² (95%CI)	P heterogeneity	P difference subtypes
Direct ELISA	13.2 [2-30.9]	NA	2	186	2.2 [1.1-4.6]	80 [13.8-95.3]	0.026	
Enzyme immunoassay (EIA)	2.5 [1.8-3.2]	NA	1	1950	NA	NA	1	
Rapid Diagnostic test	1.3 [0-5.4]	NA	1	79	NA	NA	1	
**HBV prevalence in PLHIV**								
**Study Design**								0.017
Case control	13.3 [9.4-17.8]	[1.4-34]	13	5625	4.4 [3.7-5.2]	94.8 [92.7-96.4]	< 0.001	
Clinical Trial (Baseline data)	6.7 [4.4-9.4]	[0.2-20.4]	15	13400	5.4 [4.7-6.2]	96.6 [95.5-97.4]	< 0.001	
Cohort (Baseline data)	11.3 [9.6-13.2]	[3.3-23.3]	35	71253	6.5 [6-7]	97.6 [97.2-98]	< 0.001	
Cross sectional	10.4 [9.5-11.4]	[0.7-28.5]	252	252313	6.7 [6.5-6.8]	97.7 [97.6-97.9]	< 0.001	
**Sampling**								< 0.001
Non probabilistic	11 [10.1-12]	[0.8-29.9]	268	315895	7.7 [7.5-7.9]	98.3 [98.2-98.4]	< 0.001	
Probabilistic	7.5 [6.2-9]	[0.8-19.5]	47	26696	4.2 [3.8-4.6]	94.2 [93.1-95.2]	< 0.001	
**Timing of samples collection**								0.448
Prospetively	10.5 [9.6-11.5]	[0.6-29]	254	264160	6.9 [6.7-7.1]	97.9 [97.8-98]	< 0.001	
Retrospectively	10 [8.4-11.8]	[1-26.2]	59	77870	7.3 [6.9-7.8]	98.1 [97.9-98.3]	< 0.001	
**Countries**								< 0.001
Angola	26.3 [13.4-41.6]	NA	1	38	NA	NA	1	
Benin	16.9 [14.3-19.7]	NA	1	744	NA	NA	1	
Botswana	4.9 [3.4-6.7]	[1.4-10.2]	6	1436	1.3 [1-2.1]	44.9 [0-78.2]	0.106	
Burkina Faso	14.7 [12.1-17.4]	[7.2-24]	13	3201	1.8 [1.3-2.4]	68.3 [43.6-82.2]	< 0.001	
Cameroon	12.6 [9.9-15.7]	[3.2-26.6]	17	6120	3.1 [2.5-3.7]	89.3 [84.4-92.6]	< 0.001	
Central African Republic	16.7 [5.1-32.4]	NA	1	30	NA	NA	1	
Chad	16.1 [13.5-18.9]	NA	1	725	NA	NA	1	
Democratic Republic of the Congo	6.1 [3.7-9]	[0.3-17.4]	6	1713	2.1 [1.4-3.1]	76.9 [48.4-89.6]	0.001	
Equatorial Guinea	12.6 [8.6-17.2]	NA	1	230	NA	NA	1	
Ethiopia	6 [4.2-8.1]	[0.4-17.1]	18	7016	3.3 [2.8-3.9]	90.8 [86.9-93.5]	< 0.001	
Gabon	6.2 [4.2-8.5]	NA	1	487	NA	NA	1	
Gambia	7.3 [2.3-14.8]	NA	2	1442	4.6 [2.7-7.9]	95.2 [85.8-98.4]	< 0.001	
Ghana	12.5 [9.6-15.8]	[3.3-26.2]	13	6665	3.2 [2.6-3.9]	90.2 [85.1-93.6]	< 0.001	
Guinea	13.7 [3-29.8]	NA	2	204	2.5 [1.3-5.1]	84.4 [36.2-96.2]	0.011	
Guinea-Bissau	10 [6.5-14.2]	[0-71.8]	3	1052	1.7 [1-3.2]	67.1 [0-90.5]	0.048	
Ivory Coast	13.1 [10.2-16.3]	[2.9-28.8]	18	6801	3.4 [2.9-4.1]	91.5 [88-93.9]	< 0.001	
Kenya	7 [4.2-10.4]	[0-24.1]	15	2562	3 [2.4-3.6]	88.6 [82.9-92.4]	< 0.001	
Lesotho	3.9 [2.5-5.6]	[0-30.5]	3	1685	1.3 [1-2.3]	38.2 [0-80.6]	0.198	

	Prevalence. % (95%CI)	95% Prediction interval	N Studies	N Participants	H (95%CI)	I² (95%CI)	P heterogeneity	P difference subtypes
Libya	5.4 [3.1-8.4]	NA	1	294	NA	NA	1	
Malawi	9.1 [6.6-11.9]	[1.8-20.8]	13	4529	2.6 [2.1-3.3]	85.2 [76.2-90.7]	< 0.001	
Mali	17.4 [6.4-32.1]	[0-100]	3	1153	5.5 [3.8-7.9]	96.7 [93.2-98.4]	< 0.001	
Morocco	13 [0-40.9]	NA	2	529	3.2 [1.7-6.2]	90.5 [65.7-97.4]	0.001	
Mozambique	10.7 [8.5-13.1]	[4.3-19.3]	8	4871	2.3 [1.7-3.2]	81.5 [64.6-90.3]	< 0.001	
Namibia	2.7 [0-7.9]	NA	1	75	NA	NA	1	
Niger	15.4 [6.7-26.6]	NA	1	52	NA	NA	1	
Nigeria	13.3 [11.6-15]	[3.3-28.3]	59	101365	7.4 [7-7.8]	98.2 [97.9-98.4]	< 0.001	
Rwanda	4 [3.9-4.1]	[3.7-4.3]	4	114612	1 [1-2.6]	0 [0-84.7]	0.41	
Senegal	10.6 [7-14.8]	[1.2-26.9]	7	2559	3 [2.2-4.1]	88.9 [79.7-94]	< 0.001	
Sierra Leone	7.6 [2-16]	[0-44.9]	7	579	3 [2.2-4.1]	88.8 [79.3-93.9]	< 0.001	
South Africa	9.5 [7.4-11.9]	[0.3-27.6]	42	18618	4.8 [4.4-5.2]	95.6 [94.8-96.4]	< 0.001	
Sudan	18.7 [6.4-35.4]	NA	2	716	5.2 [3.1-8.6]	96.3 [89.7-98.7]	< 0.001	
Swaziland	46 [44.1-47.8]	NA	1	2788	NA	NA	1	
Tanzania	6 [4.2-8.1]	[1-14.3]	13	20351	2.6 [2.1-3.3]	85.3 [76.5-90.8]	< 0.001	
Togo	8.9 [5.4-13.2]	[0-78.9]	3	1577	2 [1.1-3.7]	75.9 [20.8-92.7]	0.016	
Tunisia	5.6 [2.1-10.4]	NA	1	125	NA	NA	1	
Uganda	8.1 [6-10.5]	[1.7-18.3]	12	9757	3.6 [3-4.4]	92.4 [88.6-94.9]	< 0.001	
Zambia	12.1 [10.3-14.1]	[7-18.4]	8	9740	2 [1.4-2.8]	74.3 [47.9-87.3]	< 0.001	
Zimbabwe	13.7 [8.2-20.2]	NA	2	1359	2.9 [1.5-5.7]	88.3 [55.2-96.9]	0.003	
**UNAIDS Region**								< 0.001
East and Southern	7.8 [7.1-8.6]	[1.5-18.1]	148	200681	4.8 [4.5-5]	95.6 [95.2-96]	< 0.001	
North Africa and Middle East	11.5 [5-20.1]	[0-48.6]	6	1664	4.6 [3.5-5.9]	95.2 [92-97.2]	< 0.001	
West and Central	12.5 [11.5-13.5]	[3-26.9]	159	136699	5.3 [5.1-5.5]	96.4 [96.1-96.7]	< 0.001	
**WHO Region**								0.767
Africa	10.4 [9.6-11.3]	[0.7-28.5]	309	340927	7.4 [7.2-7.5]	98.2 [98.1-98.2]	< 0.001	
Eastern Mediterranean	11.5 [5-20.1]	[0-48.6]	6	1664	4.6 [3.5-5.9]	95.2 [92-97.2]	< 0.001	
**UNSD Region**								< 0.001
Central Africa	11.2 [8.9-13.6]	[2.1-25.6]	28	9343	3.3 [2.9-3.8]	91 [88.1-93.1]	< 0.001	
Eastern Africa	7.8 [6.9-8.7]	[1.9-17]	93	174797	4.7 [4.4-5]	95.4 [94.8-96]	< 0.001	
Northern Africa	11.5 [5-20.1]	[0-48.6]	6	1664	4.6 [3.5-5.9]	95.2 [92-97.2]	< 0.001	
Southern Africa	9.4 [6.5-12.6]	[0-40.5]	53	24602	7.8 [7.4-8.3]	98.4 [98.2-98.5]	< 0.001	
West Africa	12.8 [11.7-14]	[3.2-27.3]	132	127394	5.6 [5.3-5.8]	96.8 [96.5-97.1]	< 0.001	
**Country income level**								< 0.001

	Prevalence. % (95%CI)	95% Prediction interval	N Studies	N Participants	H (95%CI)	I² (95%CI)	P heterogeneity	P difference subtypes
Low-income economies	9.3 [8.2-10.4]	[1.7-21.5]	99	153229	4.6 [4.3-4.9]	95.3 [94.7-95.8]	< 0.001	
Lower-middle income economies	11.5 [10.5-12.6]	[2.2-26.4]	160	160643	6 [5.8-6.2]	97.2 [97-97.4]	< 0.001	
Upper-middle-income economies	8.6 [6.9-10.6]	[0.3-25]	52	21140	4.4 [4-4.8]	94.8 [93.9-95.6]	< 0.001	
**Age range**								< 0.001
Adults	11.3 [10.2-12.5]	[1.7-27.4]	159	136281	6 [5.7-6.2]	97.2 [97-97.4]	< 0.001	
Children	5.4 [3.5-7.7]	[0-20]	24	4732	3.1 [2.7-3.6]	89.7 [85.9-92.4]	< 0.001	
**Setting**								0.216
Community-based	11.9 [9.4-14.7]	[4.7-21.6]	12	21763	2.9 [2.3-3.6]	87.8 [80.6-92.4]	< 0.001	
Hospital-based	10.4 [9.6-11.3]	[0.7-28.3]	303	320828	7.1 [6.9-7.3]	98 [97.9-98.1]	< 0.001	
**Rural/Urban**								0.539
Rural	6.4 [2.9-10.9]	[0-25.2]	7	1880	3.1 [2.3-4.1]	89.3 [80.4-94.1]	< 0.001	
Urban	8.3 [5.6-11.5]	[1.1-21]	7	2441	2.6 [1.9-3.6]	85.3 [71.6-92.4]	< 0.001	
**ART status**								0.033
ART naïve	12.2 [9.5-15.3]	[0.1-38]	48	36928	7.7 [7.2-8.1]	98.3 [98.1-98.5]	< 0.001	
On ART	9 [7.8-10.4]	[1.2-22.6]	75	100404	6.7 [6.3-7]	97.7 [97.5-98]	< 0.001	
**PLHIV Study population**								0.001
Blood donors	15 [11.9-18.4]	[3.7-31.6]	22	4150	2.6 [2.2-3.1]	85.5 [79.4-89.9]	< 0.001	
Deceased people	10 [1.3-23.8]	NA	1	30	NA	NA	1	
General population	10.7 [9.8-11.7]	[0.8-29]	237	316865	8 [7.8-8.2]	98.4 [98.4-98.5]	< 0.001	
Injecting drug users	5.8 [1.2-13]	[0-41.7]	5	702	3 [2.1-4.3]	88.9 [76.9-94.7]	< 0.001	
Men who have sex with men	9.2 [3.2-17.7]	NA	1	65	NA	NA	1	
Patients with cancer	22.9 [0-95.8]	NA	2	68	7 [4.6-10.8]	98 [95.2-99.1]	< 0.001	
Pregnant women	7.3 [5.8-9.1]	[0.3-20.9]	45	20209	4.1 [3.7-4.5]	94.1 [92.9-95.1]	< 0.001	
Prisoners	11.5 [8.8-14.5]	NA	2	502	1	0	0.416	
**HBV and/or HCV_diagnostic_method**								< 0.001
ADVIA Centaur chemiluminometric immunoassay system	7.5 [3.1-13.6]	[0-34.2]	6	2284	3.7 [2.7-4.9]	92.5 [86.5-95.9]	< 0.001	
Agglutination test kits	40.4 [20.7-61.8]	NA	2	156	2.6 [1.3-5.2]	85.2 [39.8-96.3]	0.009	
Auszyme Assay	5.6 [2.1-10.3]	NA	2	505	1.5 [1-3]	54.5 [0-88.9]	0.138	
Chemiluminescent enzyme immunoassay (CLEIA)	9.5 [5.6-14.2]	[0-29.6]	11	3332	3.2 [2.5-4]	90.1 [84.3-93.7]	< 0.001	
Chromatographic immunoassay technique	9.6 [6.7-13]	NA	1	342	NA	NA	1	
Classical PCR	19.7 [8.5-33.9]	[0-78.1]	5	1357	5.1 [3.9-6.7]	96.2 [93.4-97.8]	< 0.001	

	Prevalence. % (95%CI)	95% Prediction interval	N Studies	N Participants	H (95%CI)	I² (95%CI)	P heterogeneity	P difference subtypes
Direct ELISA	11.5 [10.3-12.9]	[2-26.9]	107	169597	5.9 [5.6-6.2]	97.1 [96.8-97.4]	< 0.001	
Direct ELISA, Indirect ELISA	1.5 [0.3-3.5]	NA	1	260	NA	NA	1	
Electro-chemiluminescence immunoassay (ECLIA)	3 [0.4-7.7]	[0-29.5]	5	1102	3.4 [2.4-4.8]	91.2 [82.5-95.6]	< 0.001	
Enzyme immunoassay (EIA)	9 [7.4-10.8]	[0.9-23.7]	50	90499	7.9 [7.5-8.4]	98.4 [98.2-98.6]	< 0.001	
Immunoassay kit	14.7 [11-18.9]	[1.6-36.5]	21	8952	4.3 [3.8-5]	94.7 [93-96]	< 0.001	
Immunochromatographic test	8.8 [5.9-12.3]	[0.5-25.2]	13	4025	3.5 [2.9-4.2]	91.7 [87.7-94.5]	< 0.001	
Indirect ELISA	19.9 [0.5-55.1]	NA	2	212	5.5 [3.4-9]	96.7 [91.1-98.8]	< 0.001	
Inter Second Antibody Immunoassay (ISAI)	11.6 [7.6-16.3]	NA	1	207	NA	NA	1	
Microparticle Enzyme Immunoassay (MEIA)	4.8 [3.1-6.8]	NA	1	502	NA	NA	1	
Neutralization test	4.2 [2.1-7]	NA	2	1745	1.7 [1-3.6]	65.3 [0-92.1]	0.089	
Radioimmunoassay	10 [1.3-23.8]	NA	1	30	NA	NA	1	
Rapid Diagnostic test	9.1 [6.9-11.5]	[0-34.2]	64	45845	7.8 [7.4-8.2]	98.3 [98.2-98.5]	< 0.001	
Real-time PCR	8.5 [4.9-12.8]	[0-28.5]	5	2585	3.5 [2.5-4.9]	91.8 [83.9-95.8]	< 0.001	
Reverse passive hemagglutination assay	42.1 [12.7-74.8]	NA	2	65	2.6 [1.3-5.3]	85.5 [41.4-96.4]	0.009	
Serological tests	11.6 [9.4-14]	NA	1	759	NA	NA	1	
**Target detected**								0.003
HBeAg	7.1 [4.7-9.7]	[0-26.2]	34	7639	3.7 [3.3-4.2]	92.9 [91-94.3]	< 0.001	
HBsAg	10.7 [9.8-11.6]	[0.9-28.6]	267	330256	7.6 [7.4-7.8]	98.3 [98.2-98.4]	< 0.001	
HBsAg + IgM anti-HBc	3.7 [0.1-11.1]	NA	2	372	2.6 [1.3-5.2]	85.1 [39.6-96.3]	0.01	
Viral DNA	17.2 [11.5-23.8]	[0.9-46.1]	12	4324	5.1 [4.3-6]	96.2 [94.7-97.2]	< 0.001	
**HCV prevalence in PLHIV**								
**Study Design**								0.337
Case control	4.9 [1.7-9.6]	[0-27.6]	8	3967	5.3 [4.3-6.4]	96.4 [94.6-97.6]	< 0.001	
Clinical Trial (Baseline data)	8 [5.4-11]	NA	1	362	NA	NA	1	
Cohort (Baseline data)	6.2 [4.5-8.2]	[0.3-18]	22	56004	7.8 [7.1-8.6]	98.4 [98-98.6]	< 0.001	
Cross sectional	5.3 [4.4-6.2]	[0-21.1]	181	213096	7 [6.8-7.2]	97.9 [97.8-98.1]	< 0.001	
**Sampling**								0.042
Non probabilistic	4.8 [4.1-5.5]	[0-17.8]	185	267622	7.3 [7.1-7.5]	98.1 [98-98.2]	< 0.001	
Probabilistic	10.9 [4.7-19]	[0-66.8]	27	5807	8.4 [7.7-9]	98.6 [98.3-98.8]	< 0.001	
**Timing of samples collection**								0.097
Prospetively	5.7 [4.8-6.7]	[0-21.4]	169	196698	6.5 [6.3-6.8]	97.6 [97.5-97.8]	< 0.001	

	Prevalence. % (95%CI)	95% Prediction interval	N Studies	N Participants	H (95%CI)	I² (95%CI)	P heterogeneity	P difference subtypes
Retrospectively	4.3 [2.7-6.1]	[0-21.1]	40	75869	10.7 [10.1-11.3]	99.1 [99-99.2]	< 0.001	
**Countries**								< 0.001
Angola	10.5 [2.4-22.6]	NA	1	38	NA	NA	1	
Botswana	0.4 [0-1.7]	NA	2	302	1	0	0.708	
Burkina Faso	6.2 [3.9-9.1]	[0.2-18.5]	11	2762	2.6 [2-3.4]	85.3 [75.3-91.2]	< 0.001	
Burundi	10.6 [8.8-12.6]	NA	1	1053	NA	NA	1	
Cameroon	13.9 [7.1-22.4]	[0-53.5]	13	1468	4 [3.4-4.8]	93.8 [91.1-95.7]	< 0.001	
Central African Republic	3.3 [0-13.8]	NA	1	30	NA	NA	1	
Chad	1 [0.4-1.8]	NA	1	725	NA	NA	1	
Democratic Republic of the Congo	6.8 [4.5-9.6]	NA	2	421	1	0	0.968	
Egypt	34.8 [31.8-37.7]	NA	1	1004	NA	NA	1	
Equatorial Guinea	3.5 [1.4-6.3]	NA	1	230	NA	NA	1	
Ethiopia	5.6 [3.6-7.8]	[0.1-17.5]	16	10027	4 [3.4-4.7]	93.8 [91.3-95.5]	< 0.001	
Gabon	6.1 [3.1-10]	[0-83.1]	3	2015	3.3 [2-5.3]	90.6 [75.1-96.4]	< 0.001	
Gambia	4.8 [0-15.4]	[0-100]	3	774	4 [2.6-6.2]	93.8 [85.3-97.4]	< 0.001	
Ghana	3.4 [1.6-5.7]	[0-13.7]	11	9606	4 [3.3-4.9]	93.7 [90.6-95.8]	< 0.001	
Guinea-Bissau	1.3 [0.7-2]	[0-8.7]	3	1221	1 [1-3.1]	0 [0-89.6]	0.795	
Ivory Coast	1.3 [0.5-2.4]	[0-5.9]	5	1887	1.7 [1-2.7]	64 [5.2-86.3]	0.025	
Kenya	3.4 [0.4-8.7]	[0-30.7]	8	6295	5.8 [4.8-7]	97 [95.6-98]	< 0.001	
Lesotho	1.6 [0.5-3.2]	[0-40]	3	1685	1.6 [1-3]	59.7 [0-88.5]	0.083	
Libya	96.3 [93.7-98.2]	NA	1	294	NA	NA	1	
Malawi	4.3 [1.3-8.8]	[0-25.9]	7	1784	3.8 [2.9-4.9]	93 [88.1-95.9]	< 0.001	
Mali	4.5 [0-27.8]	[0-100]	3	155	3.5 [2.2-5.6]	91.9 [79.3-96.8]	< 0.001	
Morocco	11.3 [1.2-29.1]	NA	2	619	4.5 [2.6-7.7]	95 [84.8-98.3]	< 0.001	
Mozambique	1.2 [0.1-3.5]	[0-19.6]	4	4006	3.8 [2.6-5.5]	93 [85.3-96.7]	< 0.001	
Namibia	1.3 [0-5.6]	NA	1	75	NA	NA	1	
Nigeria	5.7 [4.4-7]	[0.2-17.3]	49	87019	7.3 [6.9-7.8]	98.1 [97.9-98.4]	< 0.001	
Republic of the Congo	7.7 [5.2-10.5]	NA	1	392	NA	NA	1	
Rwanda	6.7 [3.5-10.8]	[0-31.5]	4	117769	3.4 [2.3-5.1]	91.6 [81.7-96.2]	< 0.001	
Senegal	14.5 [1.9-34.9]	[0-100]	3	595	5 [3.4-7.3]	96 [91.3-98.1]	< 0.001	
Sierra Leone	0.7 [0-2.3]	[0-6.5]	7	579	1.4 [1-2.1]	45.7 [0-77.1]	0.087	
South Africa	3.1 [1-6.1]	[0-19.4]	15	3759	3.7 [3.1-4.5]	92.9 [89.8-95]	< 0.001	
Sudan	1.7 [0.6-3.3]	NA	1	358	NA	NA	1	
Tanzania	9.2 [2.9-18.3]	[0-52.9]	11	1586	5 [4.2-5.9]	95.9 [94.2-97.1]	< 0.001	

	Prevalence. % (95%CI)	95% Prediction interval	N Studies	N Participants	H (95%CI)	I² (95%CI)	P heterogeneity	P difference subtypes
Togo	2 [0.6-4.2]	NA	1	248	NA	NA	1	
Tunisia	33.4 [21.1-46.9]	NA	2	487	2.7 [1.4-5.4]	86.5 [46.5-96.6]	0.006	
Uganda	3.2 [1.2-6.1]	[0-17.4]	10	10777	4.9 [4.1-6]	95.9 [94.1-97.2]	< 0.001	
Zambia	0.5 [0-1.5]	[0-33.8]	3	1260	1.6 [1-3]	62.3 [0-89.2]	0.071	
Zimbabwe	0.8 [0-3.4]	NA	1	124	NA	NA	1	
**UNAIDS Region**								0.006
East and Southern	3.9 [3.1-4.9]	[0-14.9]	86	159487	5.7 [5.4-6]	96.9 [96.6-97.2]	< 0.001	
North Africa and Middle East	30 [8.2-58.1]	[0-100]	7	2762	14.7 [13.1-16.4]	99.5 [99.4-99.6]	< 0.001	
West and Central	5.4 [4.4-6.4]	[0-19.6]	119	111180	6.6 [6.3-6.8]	97.7 [97.5-97.9]	< 0.001	
**WHO Region**								0.016
Africa	4.7 [4.1-5.4]	[0-17]	205	270667	6.7 [6.5-6.9]	97.7 [97.6-97.9]	< 0.001	
Eastern Mediterranean	30 [8.2-58.1]	[0-100]	7	2762	14.7 [13.1-16.4]	99.5 [99.4-99.6]	< 0.001	
**UNSD Region**								< 0.001
Central Africa	9.4 [6.2-13.1]	[0-30.9]	23	5319	3.9 [3.4-4.5]	93.5 [91.4-95]	< 0.001	
Eastern Africa	4.5 [3.5-5.7]	[0-16.2]	65	154681	6.3 [5.9-6.7]	97.5 [97.1-97.7]	< 0.001	
Northern Africa	30 [8.2-58.1]	[0-100]	7	2762	14.7 [13.1-16.4]	99.5 [99.4-99.6]	< 0.001	
Southern Africa	2.2 [1-3.9]	[0-12.7]	21	5821	3.2 [2.7-3.7]	90.1 [86.3-92.9]	< 0.001	
West Africa	4.7 [3.7-5.7]	[0-18.3]	96	104846	7 [6.7-7.4]	98 [97.8-98.2]	< 0.001	
**Country income level**								0.008
Low-income economies	4 [3.2-4.9]	[0-13.5]	75	152689	5 [4.7-5.4]	96.1 [95.5-96.5]	< 0.001	
Lower-middle income economies	6.4 [5.2-7.7]	[0-24.3]	114	114065	7.9 [7.6-8.2]	98.4 [98.3-98.5]	< 0.001	
Upper-middle-income economies	5.7 [1.3-12.6]	[0-56.3]	23	6675	9.4 [8.7-10.2]	98.9 [98.7-99]	< 0.001	
**Age range**								0.118
Adults	4.5 [3.4-5.7]	[0-22]	112	72408	6.6 [6.3-6.9]	97.7 [97.5-97.9]	< 0.001	
Children	2.9 [1.5-4.8]	[0-12.7]	15	2835	2.5 [2-3.1]	83.8 [74.6-89.6]	< 0.001	
**Setting**								0.001
Community-based	19.2 [9.4-31.3]	[0-71.5]	12	24000	14 [12.8-15.2]	99.5 [99.4-99.6]	< 0.001	
Hospital-based	4.8 [4.1-5.5]	[0-17.8]	198	249008	6.7 [6.4-6.9]	97.7 [97.6-97.9]	< 0.001	
**Rural/Urban**								< 0.001
Rural	1.7 [0.4-3.8]	[0-14.8]	4	2477	2.2 [1.3-3.6]	79.3 [44.8-92.2]	0.002	
Urban	55 [39.3-70.2]	NA	1	40	NA	NA	1	
**ART status**								0.868
ART naïve	4.3 [2.2-7.1]	[0-23]	23	21607	6.3 [5.6-6.9]	97.4 [96.9-97.9]	< 0.001	
On ART	4.2 [2.6-6.2]	[0-22.5]	41	58263	9.9 [9.4-10.5]	99 [98.9-99.1]	< 0.001	

	Prevalence. % (95%CI)	95% Prediction interval	N Studies	N Participants	H (95%CI)	I² (95%CI)	P heterogeneity	P difference subtypes
**PLHIV Study population**								< 0.001
Blood donors	6.4 [3.9-9.3]	[0-20.2]	16	2822	2.5 [2-3.1]	84.3 [75.9-89.8]	< 0.001	
Commercial sex workers	7.4 [5-10.3]	NA	1	390	NA	NA	1	
Deceased people	3.3 [0-13.8]	NA	1	30	NA	NA	1	
General population	5.1 [4.3-5.9]	[0-18.2]	157	260616	7.7 [7.4-8]	98.3 [98.2-98.4]	< 0.001	
Injecting drug users	58 [11.9-96.6]	[0-100]	4	542	10.8 [8.9-13.1]	99.1 [98.7-99.4]	< 0.001	
Men who have sex with men	0 [0-8.4]	NA	1	20	NA	NA	1	
Patients with cancer	50 [5.2-94.8]	NA	2	68	4.5 [2.6-7.8]	95 [85.1-98.3]	< 0.001	
Pregnant women	1.8 [1-2.7]	[0-6.8]	29	8887	2.1 [1.8-2.5]	78.2 [69.1-84.6]	< 0.001	
Prisoners	3.7 [0.1-10.8]	NA	1	54	NA	NA	1	
**HBV and/or HCV_diagnostic_method**								< 0.001
Chemiluminescent enzyme immunoassay (CLEIA)	1.7 [0.4-3.7]	[0-7.8]	5	470	1.2 [1-1.9]	28 [0-71.7]	0.235	
Classical PCR	33.3 [0-99.6]	NA	2	335	9.7 [6.8-13.7]	98.9 [97.8-99.5]	< 0.001	
Classical RT-PCR	0.5 [0-1.6]	[0-4.4]	8	5102	1.7 [1.2-2.5]	67.3 [31.1-84.5]	0.003	
Direct ELISA	3.4 [1.2-6.7]	[0-20.3]	9	9957	6.8 [5.8-8]	97.9 [97.1-98.4]	< 0.001	
Electro-chemiluminescence immunoassay (ECLIA)	0.8 [0-2.5]	NA	1	242	NA	NA	1	
Enzyme immunoassay (EIA)	4.6 [3.5-5.9]	[0.3-12.6]	32	56448	5 [4.5-5.5]	96 [95.1-96.7]	< 0.001	
Immunoassay kit	3.9 [1.7-6.7]	[0-15.3]	6	1323	2.1 [1.4-3.1]	76.7 [48-89.6]	0.001	
Immunochromatographic test	3 [1.4-5.2]	[0-13.5]	10	16916	5.9 [5-7]	97.1 [96-97.9]	< 0.001	
Indirect ELISA	7 [5.9-8.2]	[0.6-18.7]	79	156058	5.2 [4.9-5.6]	96.4 [95.9-96.8]	< 0.001	
Inter Second Antibody Immunoassay (ISAI)	4.8 [2.3-8.2]	NA	1	207	NA	NA	1	
Passive haemagglutination test	0.5 [0-2.3]	NA	1	182	NA	NA	1	
Rapid Diagnostic test	5.4 [3.6-7.4]	[0-22.5]	41	17276	5 [4.6-5.5]	96.1 [95.3-96.7]	< 0.001	
Real-time PCR	1.4 [0.6-2.5]	NA	1	576	NA	NA	1	
Real-time RT-PCR	2.8 [0.4-7.1]	[0-36.4]	4	1527	3.9 [2.7-5.6]	93.4 [86.2-96.8]	< 0.001	
Transcription-mediated amplification assay	8.2 [4.2-13.3]	NA	1	146	NA	NA	1	
VERSANT HCV RNA Qualitative Assay	0 [0-1.7]	NA	1	100	NA	NA	1	
**Target detected**								0.01
Anti-HCV	5.6 [4.8-6.5]	[0-20.4]	195	265987	7.5 [7.3-7.8]	98.2 [98.1-98.3]	< 0.001	

	Prevalence. % (95%CI)	95% Prediction interval	N Studies	N Participants	H (95%CI)	I² (95%CI)	P heterogeneity	P difference subtypes
Viral RNA	2.5 [1-4.6]	[0-15.1]	17	7442	3.9 [3.3-4.6]	93.4 [90.9-95.2]	< 0.001	
**HBV and HCV prevalence in PLHIV**								
**Study Design**								0.926
Case control	1.2 [0-6.1]	[0-100]	3	1935	4.2 [2.7-6.4]	94.3 [86.6-97.5]	< 0.001	
Cohort (Baseline data)	0.7 [0-2.2]	[0-9.9]	5	28996	7.9 [6.5-9.7]	98.4 [97.6-98.9]	< 0.001	
Cross sectional	0.8 [0.3-1.3]	[0-4.1]	19	123479	3.4 [2.9-4]	91.3 [87.9-93.8]	< 0.001	
**Sampling**								0.868
Non probabilistic	0.9 [0.3-1.6]	[0-5.5]	20	152686	7.5 [6.8-8.2]	98.2 [97.8-98.5]	< 0.001	
Probabilistic	0.7 [0-2.3]	[0-9]	7	1724	2.5 [1.8-3.5]	84.4 [69.7-92]	< 0.001	
**Timing of samples collection**								0.473
Prospetively	0.9 [0.4-1.4]	[0-3.8]	17	128168	3.7 [3.1-4.3]	92.5 [89.6-94.7]	< 0.001	
Retrospectively	0.6 [0.1-1.5]	[0-5.7]	10	26242	5 [4.1-6]	96 [94.2-97.2]	< 0.001	
**Countries**								< 0.001
Burkina Faso	0.5 [0-2.1]	NA	1	207	NA	NA	1	
Cameroon	1 [0-4.3]	[0-88.6]	3	732	2 [1.1-3.7]	75.5 [19-92.6]	0.017	
Chad	0 [0-0.2]	NA	1	725	NA	NA	1	
Ethiopia	0.3 [0-2]	NA	2	877	2.5 [1.2-5]	83.4 [30.9-96]	0.014	
Ghana	0 [0-0.9]	NA	1	200	NA	NA	1	
Kenya	1 [0.1-2.5]	NA	1	300	NA	NA	1	
Nigeria	1.1 [0.4-2.1]	[0-6.5]	14	37067	5.8 [5-6.6]	97 [96-97.7]	< 0.001	
Rwanda	0.2 [0.1-0.2]	NA	1	113681	NA	NA	1	
Tanzania	1.7 [0-6.6]	NA	2	532	3 [1.5-5.8]	88.7 [57.1-97]	0.003	
Uganda	1.1 [0-4.8]	NA	1	89	NA	NA	1	
**UNAIDS Region**								0.626
East and Southern	0.7 [0.1-1.6]	[0-5.3]	7	115479	2.7 [2-3.7]	86.3 [74-92.8]	< 0.001	
West and Central	0.9 [0.3-1.6]	[0-5.6]	20	38931	5 [4.4-5.7]	96 [94.8-96.9]	< 0.001	
**UNSD Region**								0.831
Central Africa	0.4 [0-2.6]	[0-19.6]	4	1457	2.7 [1.8-4.3]	86.8 [68-94.5]	< 0.001	
Eastern Africa	0.7 [0.1-1.6]	[0-5.3]	7	115479	2.7 [2-3.7]	86.3 [74-92.8]	< 0.001	
West Africa	1 [0.4-1.9]	[0-6]	16	37474	5.4 [4.7-6.2]	96.6 [95.5-97.4]	< 0.001	
**Country income level**								0.002
Low-income economies	0.1 [0-0.4]	[0-1.4]	6	115579	1.6 [1-2.5]	62.1 [7.6-84.4]	0.022	
Lower-middle income economies	1.1 [0.5-1.8]	[0-5.9]	21	38831	4.8 [4.2-5.4]	95.6 [94.3-96.6]	< 0.001	

	Prevalence. % (95%CI)	95% Prediction interval	N Studies	N Participants	H (95%CI)	I² (95%CI)	P heterogeneity	P difference subtypes
**Age range**								0.019
Adults	1.7 [0.7-3.1]	[0-9.4]	13	21197	3.6 [3-4.4]	92.3 [88.5-94.8]	< 0.001	
Children	0 [0-0.9]	NA	1	187	NA	NA	1	
**Setting**								< 0.001
Community-based	2.5 [2.3-2.8]	NA	1	17882	NA	NA	1	
Hospital-based	0.7 [0.3-1]	[0-2.9]	26	136528	3.3 [2.9-3.8]	91 [88-93.2]	< 0.001	
**ART status**								0.956
ART naïve	1.4 [0.2-3.7]	[0-68.6]	3	932	2.2 [1.2-3.9]	79.3 [34-93.5]	0.008	
On ART	1.3 [0.1-3.3]	[0-12.2]	9	19949	4.3 [3.4-5.3]	94.5 [91.6-96.4]	< 0.001	
**PLHIV Study population**								0.26
General population	0.9 [0.4-1.6]	[0-5.6]	23	152195	7.1 [6.4-7.8]	98 [97.6-98.3]	< 0.001	
Pregnant women	0.2 [0-1]	[0-5.6]	4	2215	1.6 [1-2.7]	60.3 [0-86.7]	0.056	
**HBV and/or HCV_diagnostic_method**								0.001
Direct ELISA, Indirect ELISA	0.9 [0.4-1.7]	[0-4.2]	13	122305	3.4 [2.8-4.2]	91.3 [87-94.2]	< 0.001	
Enzyme immunoassay (EIA)	0.8 [0-2.3]	[0-14.1]	4	18797	3.1 [2-4.7]	89.4 [75.6-95.4]	< 0.001	
Enzyme immunoassay (EIA), Indirect ELISA	0.4 [0.3-0.5]	NA	1	10214	NA	NA	1	
Enzyme immunoassay (EIA), Rapid Diagnostic test	0 [0-0.2]	NA	1	725	NA	NA	1	
Immunochromatographic test	3.9 [0-14.4]	[0-100]	3	925	5.7 [4-8.1]	96.9 [93.7-98.5]	< 0.001	
Indirect ELISA, Direct ELISA	0 [0-0.9]	NA	1	187	NA	NA	1	
Inter Second Antibody Immunoassay (ISAI)	0.5 [0-2.1]	NA	1	207	NA	NA	1	
Rapid Diagnostic test	0 [0-0.2]	NA	2	881	1	0	0.953	
Real-time PCR, Real-time RT-PCR	0 [0-1]	NA	1	169	NA	NA	1	
**Target detected**								0.108
Anti-HCV, HBsAg	0.9 [0.4-1.5]	[0-5.3]	26	154241	6.6 [6.1-7.3]	97.7 [97.3-98.1]	< 0.001	
Viral DNA, Viral RNA	0 [0-1]	NA	1	169	NA	NA	1	
