# Supplementary material for: Epidemiology of hepatitis B virus and/or hepatitis C virus infections among people living with human immunodeficiency virus in Africa: A systematic review and meta-analysis
Source: PLoS One. 2022 May 31;17(5):e0269250. doi: 10.1371/journal.pone.0269250 (PMC9154112; doi:10.1371/journal.pone.0269250)
Supplement: S6 Fig — (PDF) [file pone.0269250.s015.pdf]

S6 Fig. Funnel chart for publications of the hepatitis B/C prevalence in PLHIV in Africa.

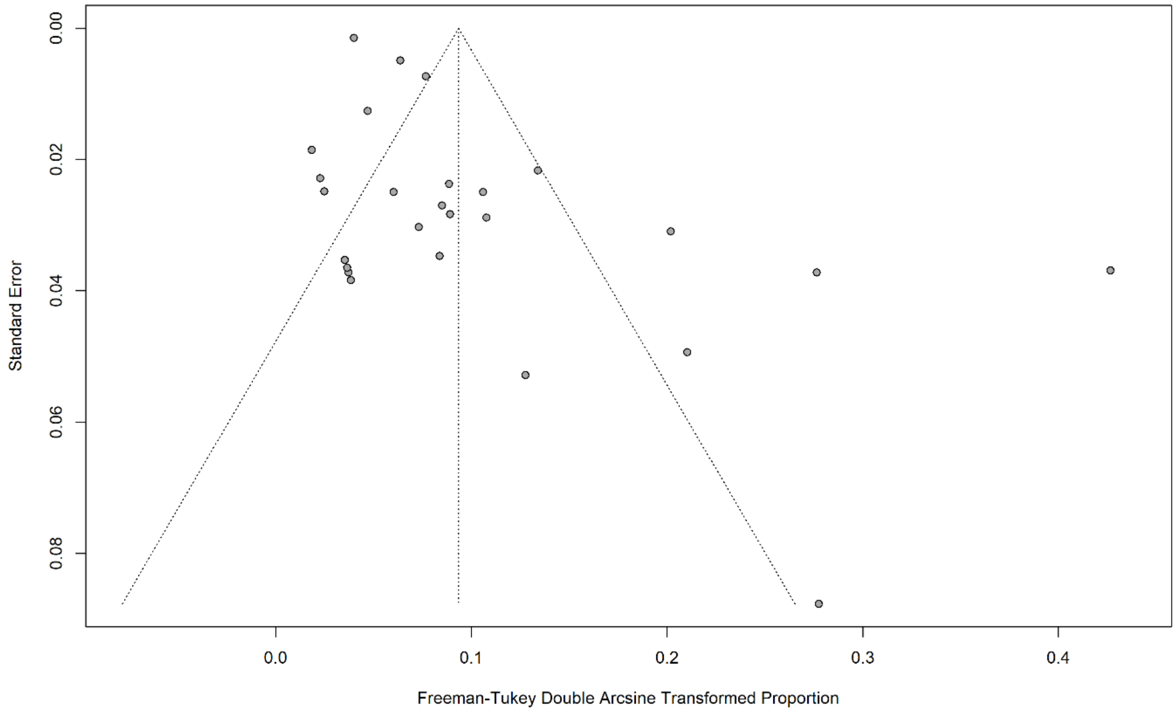

P Egger test= 0.004
