# Supplementary material for: Epidemiology of hepatitis B virus and/or hepatitis C virus infections among people living with human immunodeficiency virus in Africa: A systematic review and meta-analysis
Source: PLoS One. 2022 May 31;17(5):e0269250. doi: 10.1371/journal.pone.0269250 (PMC9154112; doi:10.1371/journal.pone.0269250)
Supplement: S1 Text — (PDF) [file pone.0269250.s016.pdf]

S1 Text: Reference list of included studies on prevalence of HBV and/or HCV in PLHIV in Africa.

1. King J, Hagemeister DT. Hepatitis B co-infection in HIV-infected patients receiving antiretroviral therapy at the TC Newman Anti Retroviral Treatment Clinic in Paarl, Western Cape. South Afr J HIV Med. 2016;17(1):336. PubMed PMID: rayyan-131263173.
2. King S, Adjei-Asante K, Appiah L, Adinku D, Beloukas A, Atkins M, et al. Antibody screening tests variably overestimate the prevalence of hepatitis C virus infection among HIV-infected adults in Ghana. J Viral Hepat. 2015;22(5):461-8. PubMed PMID: rayyan-131261995.
3. Kirakoya-Samadoulougou F, Sanou M, Samadoulougou S, Bakiono F, Kiénou K, Koumaré A, et al. High seroprevalence of hepatitis B virus and hepatitis C virus among human immunodeficiency virus carriers in blood donors of Burkina Faso: a need for their screening before HARRT therapy. J Viral Hepat. 2014;21(7):e52-3. PubMed PMID: rayyan-131263482.
4. Kouamé GM, Boyd A, Moh R, Badje A, Gabillard D, Ouattara E, et al. Higher Mortality Despite Early Antiretroviral Therapy in Human Immunodeficiency Virus and Hepatitis B Virus (HBV)-Coinfected Patients With High HBV Replication. Clin Infect Dis. 2018;66(1):112-20. PubMed PMID: rayyan-131263486.
5. Kouanfack C, Aghokeng AF, Mondain AM, Bourgeois A, Kenfack A, Mpoudi-Ngolé E, et al. Lamivudine-resistant HBV infection in HIV-positive patients receiving antiretroviral therapy in a public routine clinic in Cameroon. Antivir Ther. 2012;17(2):321-6. PubMed PMID: rayyan-131263983.
6. Kubio C, Tierney G, Quaye T, Nabilisi JW, Ziemah C, Zagbeeb SM, et al. Blood transfusion practice in a rural hospital in Northern Ghana, Damongo, West Gonja District. Transfusion. 2012;52(10):2161-6. Epub 2012/05/23. doi: 10.1111/j.1537-2995.2012.03709.x. PubMed PMID: 22612858.
7. Kwofie TB, Adigbli D, Osei-Yeboah J, Ativi E, Lokpo SY. Hepatitis B and C infections in HIV-1 patients on combination antiretroviral therapy (cART) in Ghana: implications for immunologic recovery, clinical response to treatment, and hepatotoxicity. Heliyon. 2021;7(6):e07172. Epub 2021/06/19. doi: 10.1016/j.heliyon.2021.e07172. PubMed PMID: 34141932; PubMed Central PMCID: PMCPMC8188365.
8. Kye-Duodu G, Nortey P, Malm K, Nyarko KM, Sackey SO, Ofori S, et al. Prevalence of hepatitis B virus co-infection among HIV-seropositive persons attending antiretroviral clinics in the Eastern Region of Ghana. Pan Afr Med J. 2016;25:7. PubMed PMID: rayyan-131264705.
9. Ladep NG, Agaba PA, Agbaji O, Muazu A, Ugoagwu P, Imade G, et al. Rates and impact of hepatitis on human immunodeficiency virus infection in a large African cohort. World J Gastroenterol. 2013;19(10):1602-10. PubMed PMID: rayyan-131264921.
10. Ladep NG, Agbaji O, Agaba P, Badung P, Imade G, Sankalé J, et al. Does immunological status affect the prevalence of Hepatitis C virus infection among HIV/AIDS patients? Niger J Med. 2007;16(3):231-4. PubMed PMID: rayyan-131262614.
11. Ladep NG, Agbaji OO, Agaba PA, Muazu A, Ugoagwu P, Imade G, et al. Hepatitis B Co-Infection is Associated with Poorer Survival of HIV-Infected Patients on Highly Active Antiretroviral Therapy in West Africa. J AIDS Clin Res. 2013. PubMed PMID: rayyan-131263174.
12. Lassey AT, Damale NK, Bekoe V, Klufio CA. Hepatitis C virus seroprevalence among mothers delivering at the Korle-Bu Teaching Hospital, Ghana. East Afr Med J.

2004;81(4):198-201. Epub 2005/05/12. doi: 10.4314/eamj.v81i4.9155. PubMed PMID: 15884286.
13. Laurent C, Bourgeois A, Mpoudi M, Butel C, Mpoudi-Ngolé E, Delaporte E. HIV and hepatitis C virus coinfection, Cameroon. Emerging infectious diseases. 2007;13(3):514-6. Epub 2007/06/08. doi: 10.3201/eid1303.061069. PubMed PMID: 17552122; PubMed Central PMCID: PMCPMC2725885.
14. Laurent C, Bourgeois A, Mpoudi-Ngolé E, Kouanfack C, Ciaffi L, Nkoué N, et al. High rates of active hepatitis B and C co-infections in HIV-1 infected Cameroonian adults initiating antiretroviral therapy. HIV Med. 2010;11(1):85-9. PubMed PMID: rayyan-131263474.
15. Laurent C, Henzel D, Mulanga-Kabeya C, Maertens G, Larouzé B, Delaporte E. Seroepidemiological survey of hepatitis C virus among commercial sex workers and pregnant women in Kinshasa, Democratic Republic of Congo. Int J Epidemiol. 2001;30(4):872-7. Epub 2001/08/21. doi: 10.1093/ije/30.4.872. PubMed PMID: 11511619.
16. Lawal MA. Prevalence of and risk factors for hepatitis B and C viral co-infections in HIV infected children in Lagos, Nigeria. Wellcome Open Res. 2020;15(12):e0243656. PubMed PMID: rayyan-131264641.
17. Leprêtre A, Ba I, Lacombe K, Maynart M, Toufik A, Ndiaye O, et al. Prevalence and behavioural risks for HIV and HCV infections in a population of drug users of Dakar, Senegal: the ANRS 12243 UDSEN study. J Int AIDS Soc. 2015;18(1):19888. Epub 2015/05/26. doi: 10.7448/ias.18.1.19888

19888. PubMed PMID: 26004637; PubMed Central PMCID: PMCPMC4442125.
18. Lesi OA, Kehinde MO, Oguh DN, Amira CO. Hepatitis B and C virus infection in Nigerian patients with HIV/AIDS. Niger Postgrad Med J. 2007;14(2):129-33. PubMed PMID: rayyan-131263152.
19. Liégeois F, Boyer S, Eymard-Duvernay S, Carrieri P, Kouanfack C, Domyeum J, et al. Hepatitis B testing, treatment, and virologic suppression in HIV-infected patients in Cameroon (ANRS 12288 EVOLCAM). 2020;20(1):49. PubMed PMID: rayyan-131263204.
20. Lô G, Sow-Sall A, Diop-Ndiaye H, Mandiouba NC, Thiam M, Diop F, et al. Prevalence of hepatitis B markers in Senegalese HIV-1-infected patients. J Med Virol. 2016;88(3):461-5. PubMed PMID: rayyan-131264694.
21. Loarec A, Carnimeo V, Molfino L, Kizito W, Muyindike W, Andrieux-Meyer I, et al. Extremely low hepatitis C prevalence among HIV co-infected individuals in four countries in sub-Saharan Africa. AIDS (London, England). 2019;33(2):353-5. doi: 10.1097/QAD.0000000000002070.
22. Lodenyo H, Schoub B, Ally R, Kairu S, Segal I. Hepatitis B and C virus infections and liver function in AIDS patients at Chris Hani Baragwanath Hospital, Johannesburg. East Afr Med J. 2000;77(1):13-5. PubMed PMID: rayyan-131263154.
23. Lukhwareni A, Burnett RJ, Selabe SG, Mzileni MO, Mphahlele MJ. Increased detection of HBV DNA in HBsAg-positive and HBsAg-negative South African HIV/AIDS patients enrolling for highly active antiretroviral therapy at a Tertiary Hospital. J Med Virol. 2009;81(3):406-12. PubMed PMID: rayyan-131263804.
24. Maaref F, Kilani B, Ammari L, Ben Othman A, Zribi M, Fendri C, et al. [Prevalence of hepatitis G, B and C virus infections among positive HIV population in a Tunisian Hospital, La Rabta, Tunis]. Pathol Biol (Paris). 2011;59(4):213-6. PubMed PMID: rayyan-131264743.
25. Mabayoje VO, Muhibi MA, Akindele RA, Akinleye CA, Mabayoje PS, Babatunde OS. Hepatitis C virus co-infection among people living with HIV/AIDS in a Nigerian Teaching Hospital. HIV & AIDS Review. 2013;4(12):102-5. doi: 10.1016/j.hivar.2013.09.003.

26. Mabayoje VO, Oparinde DP, Akanni EO, Taiwo SS, Muhibi MA, Adebayo TO. Seroprevalence of hepatitis B and C and of human immunodeficiency virus among blood donors in south-west Nigeria. Br J Biomed Sci. 2007;64(4):177-9. Epub 2008/02/02. doi: 10.1080/09674845.2007.11978104. PubMed PMID: 18236741.
27. Mabeya SN, Ngugi C, Nyamache AK, Lihana R. Prevalence of HepatitisB virus infections among HIV infected individuals in Nairobi, Kenya. East African Medical Journal. 2016;93(6):221-5. doi: 10.4314/eamj.v93i6.
28. Magaji FA, Okolo MO, Yiltok ES, Golit W, Anzaku SA, Ogwuche J, et al. Prevalence of hepatitis B virus infection in pregnant women with and without HIV in Jos, Nigeria. Int J Infect Dis. 2021;104:276-81. Epub 2020/12/29. doi: 10.1016/j.ijid.2020.12.058. PubMed PMID: 33359947.
29. Magoro T, Gachara G, Mavhandu L, Lum E, Kimbi HK, Ndip RN, et al. Serologic and genotypic characterization of hepatitis B virus in HIV-1 infected patients from South West and Littoral Regions of Cameroon. Virol J. 2016;13(1):178. PubMed PMID: rayyan-131265158.
30. Makanera A, Dramou I, Sidibe S, Conde M, Sy O, Camara LB, et al. Séroprévalence de la co-infection VIH /virus de l'hépatite B à l'Hôpital de l'Amitié sino-guinéenne (HASIGUI) Kipé/Conakry (Guinée). Journal of Applied Biosciences. 2019;135:13798-807. doi: 10.4314/jab.v135i0.6.
31. Manyazewal T, Sisay Z, Biadgilign S, Abegaz WE. Hepatitis B and hepatitis C virus infections among antiretroviral-naive and -experienced HIV co-infected adults. J Med Microbiol. 2014;63:742-7. PubMed PMID: rayyan-131263162.
32. Matee MI, Magesa PM, Lyamuya EF. Seroprevalence of human immunodeficiency virus, hepatitis B and C viruses and syphilis infections among blood donors at the Muhimbili National Hospital in Dar es Salaam, Tanzania. BMC Public Health. 2006;6:21. Epub 2006/02/01. doi: 10.1186/1471-2458-6-21. PubMed PMID: 16445860; PubMed Central PMCID: PMCPMC1373616.
33. Matthews PC, Beloukas A, Malik A, Carlson JM, Jooste P, Ogwu A, et al. Prevalence and Characteristics of Hepatitis B Virus (HBV) Coinfection among HIV-Positive Women in South Africa and Botswana. PLoS One. 2015;10(7):e0134037. PubMed PMID: rayyan-131264585.
34. Matthews PC, Carlson JM, Beloukas A, Malik A, Jooste P, Ogwu A, et al. HLA-A is a Predictor of Hepatitis B e Antigen Status in HIV-Positive African Adults. J Infect Dis. 2016;213(8):1248-52. PubMed PMID: rayyan-131263621.
35. Mavenyengwa RT, Mukesi M, Chipare I, Shoombe E. Prevalence of human immunodeficiency virus, syphilis, hepatitis B and C in blood donations in Namibia. BMC Public Health. 2014;14:424. Epub 2014/06/03. doi: 10.1186/1471-2458-14-424. PubMed PMID: 24884633; PubMed Central PMCID: PMCPMC4012713.
36. Mayaki Z, Dardenne N, Kabo R, Moutschen M, Sondag D, Albert A, et al. [Seroprevalence of infectious markers among blood donors in Niamey (Niger)]. Rev Epidemiol Sante Publique. 2013;61(3):233-40. Epub 2013/05/07. doi: 10.1016/j.respe.2012.12.018. PubMed PMID: 23642899.
37. Mayaphi SH, Roussow TM, Masemola DP, Olorunju SA, Mphahlele MJ, Martin DJ. HBV/HIV co-infection: the dynamics of HBV in South African patients with AIDS. S Afr Med J. 2012;102(3):157-62. PubMed PMID: rayyan-131263085.
38. Mbanya DN, Takam D, Ndumbe PM. Serological findings amongst first-time blood donors in Yaoundé, Cameroon: is safe donation a reality or a myth? Transfusion medicine (Oxford, England). 2003;13(5):267-73. Epub 2003/11/18. doi: 10.1046/j.1365-3148.2003.00453.x. PubMed PMID: 14617337.

39. Mboto CI, Davies-Russell A, Fielder M, Jewell AP. CD4+ lymphocyte values and trends in individuals infected with human immunodeficiency virus and/or co-infected with hepatitis C virus in the Gambia. Afr Health Sci. 2009;9(3):130-6. Epub 2010/07/01. PubMed PMID: 20589139; PubMed Central PMCID: PMCPMC2887036.
40. Mboto CI, Fielder M, Davies-Russell A, Jewell AP. Hepatitis C virus prevalence and serotypes associated with HIV in The Gambia. Br J Biomed Sci. 2010;67(3):140-4. PubMed PMID: rayyan-131263369.
41. Mdlalose N, Parboosing R, Moodley P. The prevalence of hepatitis B virus infection in HIV-positive and HIV-negative infants: KwaZulu-Natal, South Africa. Afr J Lab Med. 2016;5(1):283. PubMed PMID: rayyan-131264710.
42. Mirzoyan L, Berendes S, Jeffery C, Thomson J, Ben Othman H, Danon L, et al. New evidence on the HIV epidemic in Libya: why countries must implement prevention programs among people who inject drugs. J Acquir Immune Defic Syndr. 2013;62(5):577-83. Epub 2013/01/23. doi: 10.1097/QAI.0b013e318284714a. PubMed PMID: 23337363.
43. Molu JP, Essome MCN, Monamele CG, Njouom R. Sero-prevalence of HBsAg in naive HIV-infected patients in a rural locality of Cameroon. 2018;11(1):39. PubMed PMID: rayyan-131265213.
44. Moore E, Beadsworth MB, Chaponda M, Mhango B, Faragher B, Njala J, et al. Favourable one-year ART outcomes in adult Malawians with hepatitis B and C co-infection. The Journal of infection. 2010;61(2):155-63. Epub 2010/05/18. doi: 10.1016/j.jinf.2010.04.009. PubMed PMID: 20470823.
45. Mphahlele MJ, Lukhwareni A, Burnett RJ, Moropeng LM, Ngobeni JM. High risk of occult hepatitis B virus infection in HIV-positive patients from South Africa. J Clin Virol. 2006;35(1):14-20. PubMed PMID: rayyan-131263479.
46. Mpody C, Thompson P, Tabala M, Ravelomanana NLR, Malongo F, Kawende B, et al. Hepatitis B infection among pregnant and post-partum women living with HIV and on antiretroviral therapy in Kinshasa, DR Congo: A cross-sectional study. PLoS One. 2019;14(5):e0216293. PubMed PMID: rayyan-131263181.
47. Msomi N, Naidoo K, Yende-Zuma N, Padayatchi N, Govender K, Singh JA, et al. High incidence and persistence of hepatitis B virus infection in individuals receiving HIV care in KwaZulu-Natal, South Africa. BMC Infect Dis. 2020;20(1):847. PubMed PMID: rayyan-131263449.
48. Msuya SE, Mbizvo EM, Hussain A, Sam NE, Stray-Pedersen B. Seroprevalence of hepatitis B and C viruses among women of childbearing age in Moshi Urban, Tanzania. East Afr Med J. 2006;83(2):91-4. Epub 2006/05/20. doi: 10.4314/eamj.v83i2.9394. PubMed PMID: 16708880.
49. Mudawi H, Hussein W, Mukhtar M, Yousif M, Nemeri O, Glebe D, et al. Overt and occult hepatitis B virus infection in adult Sudanese HIV patients. Int J Infect Dis. 2014;29:65-70. PubMed PMID: rayyan-131264378.
50. Mugusi S, Ngaimisi E, Janabi M, Minzi O, Bakari M, Riedel KD, et al. Liver enzyme abnormalities and associated risk factors in HIV patients on efavirenz-based HAART with or without tuberculosis co-infection in Tanzania. PLoS One. 2012;7(7):e40180. Epub 2012/07/19. doi: 10.1371/journal.pone.0040180. PubMed PMID: 22808112; PubMed Central PMCID: PMCPMC3394799.
51. Mullis CE, Laeyendecker O, Reynolds SJ, Ocama P, Quinn J, Boaz I, et al. High frequency of false-positive hepatitis C virus enzyme-linked immunosorbent assay in Rakai, Uganda. Clin Infect Dis. 2013;57(12):1747-50. Epub 2013/09/21. doi: 10.1093/cid/cit602. PubMed PMID: 24051866; PubMed Central PMCID: PMCPMC3840403.
52. Mulu W, Gidey B, Chernet A, Alem G, Abera B. Hepatotoxicity and associated risk factors in HIV-infected patients receiving antiretroviral therapy at Felege Hiwot Referral

Hospital, Bahirdar, Ethiopia. Ethiopian journal of health sciences. 2013;23(3):217-26. Epub 2013/12/07. doi: 10.4314/ejhs.v23i3.4. PubMed PMID: 24307821; PubMed Central PMCID: PMCPMC3847531.
53. Munyemana JB, Mukanoheli E, Nsabimana T, Niringiyumukiza JD. HCV Seroprevalence among HIV Patients and Associated Comorbidities at One Primary Health Facility in Rwanda. Am J Trop Med Hyg. 2021;104(5):1747-50. Epub 2021/03/16. doi: 10.4269/ajtmh.20-0500. PubMed PMID: 33720846; PubMed Central PMCID: PMCPMC8103456.
54. Muriuki BM, Gicheru MM, Wachira D, Nyamache AK, Khamadi SA. Prevalence of hepatitis B and C viral co-infections among HIV-1 infected individuals in Nairobi, Kenya. BMC Res Notes. 2013;6:363. PubMed PMID: rayyan-131264679.
55. Muro FJ, Fiorillo SP, Sakasaka P, Odhiambo C, Reddy EA, Cunningham CK, et al. Seroprevalence of Hepatitis B and C Viruses Among Children in Kilimanjaro Region, Tanzania. Journal of the Pediatric Infectious Diseases Society. 2013;2(4):320-6. Epub 2013/12/24. doi: 10.1093/jpids/pit018. PubMed PMID: 24363930; PubMed Central PMCID: PMCPMC3869471.
56. Musa S, Yakubu AM, Muktar HM. Prevalence of hepatitis C Antibody in Human Immunodeficiency Virus infected children. Nigerian Journal of Paediatrics. 2016;43(1):34-9. doi: 10.4314/njp.v43i1.7.
57. Musyoki AM, Msibi TL, Motswaledi MH, Selabe SG, Monokoane TS, Mphahlele MJ. Active co-infection with HBV and/or HCV in South African HIV positive patients due for cancer therapy. J Med Virol. 2015;87(2):213-21. PubMed PMID: rayyan-131261914.
58. Mutwa PR, Boer KR, Rusine JB, Muganga N, Tuyishimire D, Reiss P, et al. Hepatitis B virus prevalence and vaccine response in HIV-infected children and adolescents on combination antiretroviral therapy in Kigali, Rwanda. Pediatr Infect Dis J. 2013;32(3):246-51. PubMed PMID: rayyan-131263267.
59. Mwatelah RS, Lwembe RM, Osman S, Ogutu BR, Aman R, Kitawi RC, et al. Co-Infection Burden of Hepatitis C Virus and Human Immunodeficiency Virus among Injecting Heroin Users at the Kenyan Coast. PLoS One. 2015;10(7):e0132287. Epub 2015/07/25. doi: 10.1371/journal.pone.0132287. PubMed PMID: 26208212; PubMed Central PMCID: PMCPMC4514798.
60. Mweemba A, Zanolini A, Mulenga L, Emge D, Chi BH, Wandeler G, et al. Chronic hepatitis B virus coinfection is associated with renal impairment among Zambian HIV-infected adults. Clin Infect Dis. 2014;59(12):1757-60. PubMed PMID: rayyan-131262301.
61. Nagalo BM, Bisseye C, Sanou M, Kienou K, Nebié YK, Kiba A, et al. Seroprevalence and incidence of transfusion-transmitted infectious diseases among blood donors from regional blood transfusion centres in Burkina Faso, West Africa. Tropical medicine & international health : TM & IH. 2012;17(2):247-53. Epub 2011/10/13. doi: 10.1111/j.1365-3156.2011.02902.x. PubMed PMID: 21988100.
62. Nagalo MB, Sanou M, Bisseye C, Kaboré MI, Nebie YK, Kienou K, et al. Seroprevalence of human immunodeficiency virus, hepatitis B and C viruses and syphilis among blood donors in Koudougou (Burkina Faso) in 2009. Blood transfusion = Trasfusione del sangue. 2011;9(4):419-24. Epub 2011/08/16. doi: 10.2450/2011.0112-10. PubMed PMID: 21839011; PubMed Central PMCID: PMCPMC3200412.
63. Nagu TJ, Bakari M, Matee M. Hepatitis A, B and C viral co-infections among HIV-infected adults presenting for care and treatment at Muhimbili National Hospital in Dar es Salaam, Tanzania. BMC Public Health. 2008;8:416. PubMed PMID: rayyan-131263137.
64. Nakwagala FN, Kagimu MM. Hepatitis B virus and hiv infections among patients in Mulago hospital. East Afr Med J. 2002;79(2):68-72. Epub 2002/10/17. doi: 10.4314/eamj.v79i2.8903. PubMed PMID: 12380879.

65. Naniche D, Letang E, Nhampossa T, David C, Menendez C, Alonso P. Alterations in T cell subsets in human immunodeficiency virus-infected adults with co-infections in southern Mozambique. Am J Trop Med Hyg. 2011;85(4):776-81. PubMed PMID: rayyan-131261973.
66. Ndjomou J, Kupfer B, Kochan B, Zekeng L, Kaptue L, Matz B. Hepatitis C virus infection and genotypes among human immunodeficiency virus high-risk groups in Cameroon. J Med Virol. 2002;66(2):179-86. PubMed PMID: rayyan-131263349.
67. Ndjoyi-Mbiguino A, Kombe Kombe AJ, Bivigou-Mboumba B, Zoa-Assoumou S, Akombi FL, Nzengui Nzengui F, et al. Low prevalence of HCV infection with predominance of genotype 4 among HIV patients living in Libreville, Gabon. 2018;13(1):e0190529. PubMed PMID: rayyan-131264052.
68. Ndow G, Gore ML, Shimakawa Y, Suso P, Jatta A, Tamba S, et al. Hepatitis B testing and treatment in HIV patients in The Gambia-Compliance with international guidelines and clinical outcomes. PLoS One. 2017;12(6):e0179025. PubMed PMID: rayyan-131263203.
69. N'Dri-Yoman T, Anglaret X, Messou E, Attia A, Polneau S, Toni T, et al. Occult HBV infection in untreated HIV-infected adults in Cote d'Ivoire. Antiviral Therapy. 2010;15(7):1029-34. PubMed PMID: rayyan-131264332.
70. Newton OE, Oghene OA, Okonko IO. Anti-HCV antibody among newly diagnosed HIV patients in Ughelli, a suburban area of Delta State Nigeria. Afr Health Sci. 2015;15(3):728-36. PubMed PMID: rayyan-131262000.
71. Nkengasong JN, Claeys H, De Beenhouwer H, Heyndrickxs L, Ayuk J, Lobe V, et al. Hepatitis C virus antibody, viraemia and genotypes in individuals infected with HIV-1 in Cameroon. Ann Soc Belg Med Trop. 1994;74(3):249-52. PubMed PMID: rayyan-131263340.
72. Nnakenyi ID, Uchechukwu C, Nto-Ezimah U. Prevalence of hepatitis B and C virus co-infection in HIV positive patients attending a health institution in southeast Nigeria. Afr Health Sci. 2020;20(2):579-86. PubMed PMID: rayyan-131264680.
73. Noubiap JJ, Aka PV, Nanfack AJ, Agyingi LA, Ngai JN, Nyambi PN. Hepatitis B and C Co-Infections in Some HIV-Positive Populations in Cameroon, West Central Africa: Analysis of Samples Collected Over More Than a Decade. PLoS One. 2015;10(9):e0137375. PubMed PMID: rayyan-131263141.
74. Noubiap JJ, Joko WY, Nansseu JR, Tene UG, Siaka C. Sero-epidemiology of human immunodeficiency virus, hepatitis B and C viruses, and syphilis infections among first-time blood donors in Edéa, Cameroon. Int J Infect Dis. 2013;17(10):e832-7. Epub 2013/01/16. doi: 10.1016/j.ijid.2012.12.007. PubMed PMID: 23317526.
75. Ntagirabiri R, Ngendakumana F, Niyongabo T. Co-infection par le virus de l’immunodéficience humaine et le virus de l’hépatite C au Burundi. J Afr Hepato Gastroenterol. 2012;6(2):128-31. doi: 10.1007/s12157-012-0387-2.
76. Nwokedi EE, Emokpae MA, Dutse AI. Human immunodeficiency virus and hepatitis B virus co-infection among patients in Kano Nigeria. Niger J Med. 2006;15(3):227-9. PubMed PMID: rayyan-131263661.
77. Nwolisa E, Mbanefo F, Ezeogu J, Amadi P. Prevalence of hepatitis B co-infection amongst HIV infected children attending a care and treatment centre in Owerri, South-eastern Nigeria. Pan Afr Med J. 2013;14:89. PubMed PMID: rayyan-131264687.
78. Nyirenda M, Beadsworth MB, Stephany P, Hart CA, Hart IJ, Munthali C, et al. Prevalence of infection with hepatitis B and C virus and coinfection with HIV in medical inpatients in Malawi. The Journal of infection. 2008;57(1):72-7. Epub 2008/06/17. doi: 10.1016/j.jinf.2008.05.004. PubMed PMID: 18555534.
79. Obienu O, Nwokediuko S. Selected biochemical and hematological abnormalities in Nigerians with human immunodeficiency virus and hepatitis C virus coinfection. Hepat Med.

2011;3:63-8. Epub 2011/01/01. doi: 10.2147/hmer.S21735. PubMed PMID: 24367222; PubMed Central PMCID: PMCPMC3846592.
80. Ocama P, Castelnuovo B, Kamya MR, Kirk GD, Reynolds SJ, Kiragga A, et al. Low frequency of liver enzyme elevation in HIV-infected patients attending a large urban treatment centre in Uganda. Int J STD AIDS. 2010;21(8):553-7. PubMed PMID: rayyan-131264048.
81. Ocama P, Katwere M, Piloya T, Feld J, Opio KC, Kambugu A, et al. The spectrum of liver diseases in HIV infected individuals at an HIV treatment clinic in Kampala, Uganda. Afr Health Sci. 2008;8(1):8-12. PubMed PMID: rayyan-131265397.
82. Odama LE, Momoh-Negedu OR, Ya'aba SB, Balogun M, Inyang US, Agwale SM. Prevalence of hepatitis C virus among human immunodeficiency virus infected patients and blood donors in Nigeria. Journal of Phytomedicine and Therapeutics. 2004;9. doi: 10.4314/jopat.v9i1.48587.
83. Ogutu EO, Amayo EO, Okoth F, Lule GN. The prevalence of hepatitis B surface antigen (HBsAg), anti-hepatitis B surface (anti-HBs) and anti-hepatitis B core (anti-HBc) in patients with acquired immuno-deficiency syndrome (AIDS). East Afr Med J. 1990;67(5):355-8. PubMed PMID: rayyan-131264698.
84. Ogwu-Richard SO, Ojo DA, Akingbade OA, Okonko IO. Triple positivity of HBsAg, anti-HCV antibody, and HIV and their influence on CD4+ lymphocyte levels in the highly HIV infected population of Abeokuta, Nigeria. Afr Health Sci. 2015;15(3):719-27. PubMed PMID: rayyan-131265579.
85. Ojide CK, Kalu EI, Ogbaini-Emevon E, Nwadike VU. Co-infections of hepatitis B and C with human immunodeficiency virus among adult patients attending human immunodeficiency virus outpatients clinic in Benin City, Nigeria. Niger J Clin Pract. 2015;18(4):516-21. PubMed PMID: rayyan-131262369.
86. Okeke TC, Obi SN, Okezie OA, Ugwu EO, Akogu SP, Ocheni S, et al. Coinfection with hepatitis B and C viruses among HIV positive pregnant women in Enugu south east, Nigeria. Niger J Med. 2012;21(1):57-60. PubMed PMID: rayyan-131262364.
87. Okocha EC, Oguejiofor OC, Odenigbo CU, Okonkwo UC, Asomugha L. Prevalence of hepatitis B surface antigen seropositivity among HIV-infected and non-infected individuals in Nnewi, Nigeria. Niger Med J. 2012;53(4):249-53. PubMed PMID: rayyan-131264700.
88. Okonko IO, Horsefall SJ, Okerentugba PO, Frank-Peterside N. HBV and HIV coinfections among intending blood donors in Port Harcourt, Nigeria. J Immunoassay Immunochem. 2015;36(4):359-67. PubMed PMID: rayyan-131263073.
89. Okoth SB, Rehmani OF, Karoney MJ, Diero L, Ayuo PO. Sero prevalence of hepatitis B and C viruses among HIV infected patients in a HIV care program in Kenya: a cross sectional study. East African Medical Journal. 2017;94(4):266-73. doi: 10.4314/eamj.v94i4.
90. Okwuraiwe AP, Audu RA, Salu OB, Onwuamah CK, Amoo OS, Ige FA, et al. Immunological and virological response to haart in HIV-1 patients co-infected with hepatitis B and C viruses. West African Journal of Medicine. 2012;31(2):124-8.
91. Olatunji PO, Iseniyi JO. Hepatitis B and C viruses co-infection with Human Immodeficiency Virus (HIV) in infected patients at UITH, Ilorin. Nigerian Medical Practitioner. 2008;54(1):8-10. doi: 10.4314/nmp.v54i1.28940.
92. Olawumi HO, Olanrewaju DO, Shittu AO, Durotoye IA, Akande AA, Nyamngee A. Effect of hepatitis-B virus co-infection on CD4 cell count and liver function of HIV infected patients. Ghana Med J. 2014;48(2):96-100. PubMed PMID: rayyan-131262667.
93. Olokoba AB, Olokoba LB, Midala J, Aderibigbe S. Hepatitis C Virus and Human Immunodeficiency Virus Co-Infection in North-Eastern Nigeria. 2008:3.

94. Omatola CA, Idofe J, Okolo MO, Adejo PO, Maina MM, Oyiguh JA. Seroprevalence of HBV among people living with HIV in Anyigba, Kogi State, Nigeria. Afr Health Sci. 2019;19(2):1938-46. PubMed PMID: rayyan-131265215.
95. Omatola CA, Okolo MO, Adaji DM, Mofolorunsho CK, Abraham Oyiguh J, Zige DV, et al. Coinfection of Human Immunodeficiency Virus-Infected Patients with Hepatitis B Virus in Lokoja, North Central Nigeria. Viral Immunol. 2020;33(5):391-5. PubMed PMID: rayyan-131262361.
96. Omatola CA, Onoja BA, Thomas T. High Rate of Hepatitis B Virus Surface Antigenemia Among People Living with HIV/AIDS in Kakuri, Kaduna State, North West Nigeria. F1000Res. 2017;30(7):516-21. PubMed PMID: rayyan-131263471.
97. Omosigho PO, Inyinbor HE, Emumwen EG, Mohammed SK, Ledogo G, Njab J, et al. Hepatitis C virus co-infection in human immuno deficiency virus positive population in Bida, North Central Nigeria. Internet Journal of Infectious Diseases. 2011;9.
98. Opaleye OO, Akanbi OA, Osundare FA, Wang B, Adesina O, Oluremi AS, et al. Prevalence and characteristics of hepatitis B and D virus infections among HIV-positive individuals in Southwestern Nigeria. Virol J. 2021;18(1):20. PubMed PMID: rayyan-131264584.
99. O'Reilly JI, Ocama P, Opio CK, Alfred A, Paintsil E, Seremba E, et al. Risk Factors and Seroprevalence of Hepatitis C among Patients Hospitalized at Mulago Hospital, Uganda. J Trop Med. 2011;2011:598341. Epub 2011/08/17. doi: 10.1155/2011/598341. PubMed PMID: 21845196; PubMed Central PMCID: PMCPMC3153916.
100. Oronsaye FE, Oronsaye JI. Prevalence of HIV-positives and hepatitis B surface antigen-positives among donors in the University of Benin Teaching Hospital, Nigeria. Tropical doctor. 2004;34(3):159-60. Epub 2004/07/23. doi: 10.1177/004947550403400312. PubMed PMID: 15267047.
101. Oshitani H, Kasolo F, Luo NP, Mpabalwani M, Mizuta K, Numata N, et al. Low prevalence of hepatitis C virus infection in Lusaka, Zambia. Trans R Soc Trop Med Hyg. 1995;89(4):380. Epub 1995/07/01. doi: 10.1016/0035-9203(95)90017-9. PubMed PMID: 7570869.
102. Oshitani H, Kasolo FC, Mpabalwani M, Mizuta K, Luo NP, Suzuki H, et al. Prevalence of hepatitis B antigens in human immunodeficiency virus type 1 seropositive and seronegative pregnant women in Zambia. Trans R Soc Trop Med Hyg. 1996;90(3):235-6. PubMed PMID: rayyan-131264686.
103. Otegbayo JA, Taiwo BO, Akingbola TS, Odaibo GN, Adedapo KS, Penugonda S, et al. Prevalence of hepatitis B and C seropositivity in a Nigerian cohort of HIV-infected patients. Ann Hepatol. 2008;7(2):152-6. PubMed PMID: rayyan-131264678.
104. Otuonye NM, Olukoya DK, Odunukwe NN, Idigbe EO, Udeaja MN, Bamidele M, et al. HIV association with conventional STDS (sexual transmitted diseases) in Lagos State, Nigeria. West Afr J Med. 2002;21(2):153-6. Epub 2002/10/31. PubMed PMID: 12403041.
105. Ouattara SA, Meite M, Aron Y, Akran V, Gody M, Manlan LK, et al. Increase of the prevalence of hepatitis B virus surface antigen related to immunodeficiency inherent in acquired immune deficiency syndrome (AIDS). J Acquir Immune Defic Syndr (1988). 1990;3(3):282-6. PubMed PMID: rayyan-131263802.
106. Ouermi D, Simpore J, Belem AM, Sanou DS, Karou DS, Ilboudo D, et al. Co-infection of Toxoplasma gondii with HBV in HIV-infected and uninfected pregnant women in Burkina Faso. Pak J Biol Sci. 2009;12(17):1188-93. PubMed PMID: rayyan-131262363.
107. Pappoe F, Hagan CKO, Obiri-Yeboah D, Nsiah P. Sero-prevalence of hepatitis B and C viral infections in Ghanaian HIV positive cohort: a consideration for their health care. BMC Infect Dis. 2019;19(1):380. PubMed PMID: rayyan-131265228.

108. Parboosing R, Paruk I, Lalloo UG. Hepatitis C virus seropositivity in a South African cohort of HIV co-infected ARV naive patients is associated with renal insufficiency and increased mortality. Journal of Medical Virology. 2008;80(9):1530-6. PubMed PMID: rayyan-131263371.
109. Patassi A, Benaboud S, Landoh DE, Salou M, Dagnra AC, Saka B, et al. Hepatitis B infection in HIV-1-infected patients receiving highly active antiretroviral therapy in Lome, Togo: Prevalence and molecular consequences. South African medical journal = Suid-Afrikaanse tydskrif vir geneeskunde. 2016;106(6). PubMed PMID: rayyan-131263185.
110. Patel P, Davis S, Tolle M, Mabikwa V, Anabwani G. Prevalence of hepatitis B and hepatitis C coinfections in an adult HIV centre population in Gaborone, Botswana. Am J Trop Med Hyg. 2011;85(2):390-4. PubMed PMID: rayyan-131264683.
111. Pawlotsky JM, Bélec L, Grésenguet G, Deforges L, Bouvier M, Duval J, et al. High prevalence of hepatitis B, C, and E markers in young sexually active adults from the Central African Republic. J Med Virol. 1995;46(3):269-72. Epub 1995/07/01. doi: 10.1002/jmv.1890460318. PubMed PMID: 7561802.
112. Peter YJ, Olayinka AT, Agbaji OO, Ogunsola FT. Epidemiology of Hepatitis B and Hepatitis C Virus infections among HIV counseling and testing clients in Jos, North central Nigeria. African Journal of Clinical and Experimental Microbiology. 2015;16(3):92-6. doi: 10.4314/ajcem.v16i3.2.
113. Pirillo MF, Bassani L, Germinario EA, Mancini MG, Vyankandondera J, Okong P, et al. Seroprevalence of hepatitis B and C viruses among HIV-infected pregnant women in Uganda and Rwanda. J Med Virol. 2007;79(12):1797-801. PubMed PMID: rayyan-131265231.
114. Pirillo MF, Scarcella P, Andreotti M, Jere H, Buonomo E, Sagno JB, et al. Hepatitis B virus mother-to-child transmission among HIV-infected women receiving lamivudine-containing antiretroviral regimens during pregnancy and breastfeeding. J Viral Hepat. 2015;22(3):289-96. PubMed PMID: rayyan-131263263.
115. Plamondon M, Labbé AC, Frost E, Deslandes S, Alves AC, Bastien N, et al. Hepatitis C virus infection in Guinea-Bissau: a sexually transmitted genotype 2 with parenteral amplification? PLoS One. 2007;2(4):e372. Epub 2007/04/19. doi: 10.1371/journal.pone.0000372. PubMed PMID: 17440608; PubMed Central PMCID: PMCPMC1847532.
116. Powell EA, Gededzha MP, Rentz M, Rakgole NJ, Selabe SG, Seleise TA, et al. Mutations associated with occult hepatitis B in HIV-positive South Africans. J Med Virol. 2015;87(3):388-400. PubMed PMID: rayyan-131264233.
117. Price H, Dunn D, Zachary T, Vudriko T, Chirara M, Kityo C, et al. Hepatitis B serological markers and plasma DNA concentrations. AIDS (London, England). 2017;31(8):1109-17. doi: 10.1097/QAD.0000000000001454.
118. Rabenau HF, Lennemann T, Kircher C, Gürtler L, Staszewski S, Preiser W, et al. Prevalence- and gender-specific immune response to opportunistic infections in HIV-infected patients in Lesotho. Sex Transm Dis. 2010;37(7):454-9. Epub 2010/06/10. doi: 10.1097/OLQ.0b013e3181cfcc2b. PubMed PMID: 20531031.
119. Rahlenbeck SI, Yohannes G, Molla K, Reifen R, Assefa A. Infection with HIV, syphilis and hepatitis B in Ethiopia: a survey in blood donors. Int J STD AIDS. 1997;8(4):261-4. Epub 1997/04/01. doi: 10.1258/0956462971919886. PubMed PMID: 9147161.
120. Ramírez Mena A, Tine JM, Fortes L, Ndiaye O, Ka D, Ngom NF, et al. Hepatitis B screening practices and viral control among persons living with HIV in urban Senegal. J Viral Hepat. 2022;29(1):60-8. Epub 2021/10/06. doi: 10.1111/jvh.13615. PubMed PMID: 34610183.

121. Rebbani K, Ouladlahsen A, Bensghir A, Akil A, Lamdini H, Issouf H, et al. Co-infections with hepatitis B and C viruses in human immunodeficiency virus-infected patients in Morocco. Clin Microbiol Infect. 2013;19(10):E454-7. PubMed PMID: rayyan-131262371.
122. Rouet F, Chaix ML, Inwoley A, Anaky MF, Fassinou P, Kpozehouen A, et al. Frequent occurrence of chronic hepatitis B virus infection among West African HIV type-1-infected children. Clin Infect Dis. 2008;46(3):361-6. PubMed PMID: rayyan-131262970.
123. Rouet F, Chaix ML, Inwoley A, Msellati P, Viho I, Combe P, et al. HBV and HCV prevalence and viraemia in HIV-positive and HIV-negative pregnant women in Abidjan, Côte d'Ivoire: the ANRS 1236 study. J Med Virol. 2004;74(1):34-40. PubMed PMID: rayyan-131263070.
124. Rouet F, Deleplancque L, Mboumba BB, Sica J, Mouinga-Ondémé A, Liégeois F, et al. Usefulness of a fourth generation ELISA assay for the reliable identification of HCV infection in HIV-positive adults from Gabon (Central Africa). PLoS One. 2015;10(1):e0116975. PubMed PMID: rayyan-131265644.
125. Rusine J, Ondoa P, Asiimwe-Kateera B, Boer KR, Uwimana JM, Mukabayire O, et al. High seroprevalence of HBV and HCV infection in HIV-infected adults in Kigali, Rwanda. PLoS One. 2013;8(5):e63303. PubMed PMID: rayyan-131263481.
126. Sadoh AE, Sadoh WE, Iduoriyekemwen NJ. HIV co-infection with hepatitis B and C viruses among Nigerian children in an antiretroviral treatment programme. South African Journal of Child Health. 2011;5(1):7-10.
127. Sagoe KW, Agyei AA, Ziga F, Lartey M, Adiku TK, Seshi M, et al. Prevalence and impact of hepatitis B and C virus co-infections in antiretroviral treatment naïve patients with HIV infection at a major treatment center in Ghana. J Med Virol. 2012;84(1):6-10. PubMed PMID: rayyan-131264605.
128. Salpini R, Fokam J, Ceccarelli L, Santoro M-M, Nanfack A, Sosso SM, et al. High Burden of HBV-Infection and Atypical HBV Strains among HIV-infected Cameroonians. Current HIV research. 2016;14(2):165-71. doi: 10.2174/1570162x13666150930114742.
129. Salu OB, Oyefolu AOB, Gbadegesin A, James AB, Oke BO, Ashaka OS, et al. Co–infection of hepatitis B and C viruses among human immunodeficiency virus infected children in Lagos, Nigeria. African Journal of Clinical and Experimental Microbiology. 2018;19(2):125-32. doi: 10.4314/ajcem.v19i2.8.
130. Salyani A. Occult hepatitis B virus infection in a Kenyan cohort of HIV infected anti-retroviral therapy naïve adults. PLoS One. 2021;16(1):e0244947. PubMed PMID: rayyan-131264345.
131. Seremba E, Ocama P, Opio CK, Kagimu M, Thomas DL, Yuan HJ, et al. Poor performance of hepatitis C antibody tests in hospital patients in Uganda. J Med Virol. 2010;82(8):1371-8. Epub 2010/06/24. doi: 10.1002/jmv.21817. PubMed PMID: 20572078.
132. Shimelis T, Tassachew Y, Tadewos A, Hordofa MW, Amsalu A, Tadesse BT, et al. Coinfections with hepatitis B and C virus and syphilis among HIV-infected clients in Southern Ethiopia: a cross-sectional study. HIV AIDS (Auckl). 2017;9:203-10. PubMed PMID: rayyan-131262370.
133. Shimelis T, Torben W, Medhin G, Tebeje M, Andualm A, Demessie F, et al. Hepatitis B virus infection among people attending the voluntary counselling and testing centre and anti-retroviral therapy clinic of St Paul's General Specialised Hospital, Addis Ababa, Ethiopia. Sexually transmitted infections. 2008;84(1):37-41. Epub 2007/09/07. doi: 10.1136/sti.2007.027326. PubMed PMID: 17804606.
134. Simani OE, Leroux-Roels G, François G, Burnett RJ, Meheus A, Mphahlele MJ. Reduced detection and levels of protective antibodies to hepatitis B vaccine in under 2-year-old HIV positive South African children at a paediatric outpatient clinic. Vaccine. 2009;27(1):146-51. PubMed PMID: rayyan-131264942.

135. Simpore J, Granato M, Santarelli R, Nsme RA, Coluzzi M, Pietra V, et al. Prevalence of infection by HHV-8, HIV, HCV and HBV among pregnant women in Burkina Faso. J Clin Virol. 2004;31(1):78-80. Epub 2004/08/04. doi: 10.1016/j.jcv.2004.06.001. PubMed PMID: 15288619.
136. Simpore J, Ilboudo D, Karou D, Pietra V, Granato M, Esposito M, et al. Prevalence of HHV-8 Infections Associated with HIV, HBV and HCV in Pregnant Women in Burkina Faso. J of Medical Sciences. 2005;6(1):93-8. doi: 10.3923/jms.2006.93.98.
137. Simpore J, Ilboudo D, Samandoulougou A, Guardo P, Castronovo P, Musumeci S. HCV and HIV co-infection in pregnant women attending St. Camille Medical Centre in Ouagadougou (Burkina Faso). J Med Virol. 2005;75(2):209-12. Epub 2004/12/17. doi: 10.1002/jmv.20258. PubMed PMID: 15602740.
138. Simpore J, Savadogo A, Ilboudo D, Nadambega MC, Esposito M, Yara J, et al. Toxoplasma gondii, HCV, and HBV seroprevalence and co-infection among HIV-positive and -negative pregnant women in Burkina Faso. J Med Virol. 2006;78(6):730-3. PubMed PMID: rayyan-131265523.
139. Sonderup MW, Wainwright H, Hall P, Hairwadzi H, Spearman CW. A clinicopathological cohort study of liver pathology in 301 patients with human immunodeficiency virus/acquired immune deficiency syndrome. Hepatology. 2015;61(5):1721-9. PubMed PMID: rayyan-131262351.
140. Soni PN, Tait DR, Kenoyer DG, Fernandes-Costa F, Naicker S, Gopaul W, et al. Hepatitis C virus antibodies among risk groups in a South African area endemic for hepatitis B virus. J Med Virol. 1993;40(1):65-8. Epub 1993/05/01. doi: 10.1002/jmv.1890400113. PubMed PMID: 7685810.
141. Stabinski L, Reynolds SJ, Ocama P, Laeyendecker O, Ndyanabo A, Kiggundu V, et al. High prevalence of liver fibrosis associated with HIV infection: a study in rural Rakai, Uganda. Antivir Ther. 2011;16(3):405-11. PubMed PMID: rayyan-131263469.
142. Stokx J, Gillet P, De Weggheleire A, Casas EC, Maendaenda R, Beulane AJ, et al. Seroprevalence of transfusion-transmissible infections and evaluation of the pre-donation screening performance at the Provincial Hospital of Tete, Mozambique. BMC Infect Dis. 2011;11:141. Epub 2011/05/25. doi: 10.1186/1471-2334-11-141. PubMed PMID: 21605363; PubMed Central PMCID: PMCPMC3120673.
143. Sutcliffe S, Taha TE, Kumwenda NI, Taylor E, Liomba GN. HIV-1 prevalence and herpes simplex virus 2, hepatitis C virus, and hepatitis B virus infections among male workers at a sugar estate in Malawi. J Acquir Immune Defic Syndr. 2002;31(1):90-7. Epub 2002/09/28. doi: 10.1097/00126334-200209010-00012. PubMed PMID: 12352155.
144. Tamo IMG, Ngounoue MD, Ambassa AC, Fewou PM. Importance of biochemical exploration of the liver in the control of disease progression in people living with HIV/AIDS and coinfected by HIV and Hepatitis C virus in Cameroon. Journal of the Cameroon Academy of Sciences. 2016;13(3):81-8. doi: 10.4314/jcas.v13i3.
145. Taye S, Lakew M. Impact of hepatitis C virus co-infection on HIV patients before and after highly active antiretroviral therapy: an immunological and clinical chemistry observation, Addis Ababa, Ethiopia. BMC immunology. 2013;14:23. Epub 2013/05/18. doi: 10.1186/1471-2172-14-23. PubMed PMID: 23679118; PubMed Central PMCID: PMCPMC3663769.
146. Telatela SP, Matee MI, Munubhi EK. Seroprevalence of hepatitis B and C viral co-infections among children infected with human immunodeficiency virus attending the paediatric HIV care and treatment center at Muhimbili National Hospital in Dar-es-Salaam, Tanzania. BMC Public Health. 2007;7:338. PubMed PMID: rayyan-131265225.
147. Tess BH, Levin A, Brubaker G, Shao J, Drummond JE, Alter HJ, et al. Seroprevalence of hepatitis C virus in the general population of northwest Tanzania. Am J Trop Med Hyg.

2000;62(1):138-41. Epub 2000/04/13. doi: 10.4269/ajtmh.2000.62.138. PubMed PMID: 10761739.
148. Tessema B, Yismaw G, Kassu A, Amsalu A, Mulu A, Emmrich F, et al. Seroprevalence of HIV, HBV, HCV and syphilis infections among blood donors at Gondar University Teaching Hospital, Northwest Ethiopia: declining trends over a period of five years. BMC Infect Dis. 2010;10:111. Epub 2010/05/13. doi: 10.1186/1471-2334-10-111. PubMed PMID: 20459703; PubMed Central PMCID: PMCPMC2881920.
149. Torpey K, Ogyiri L, Cuylaerts V, Agyeman S, Agyei-Nkansah A, Buyze J, et al. Hepatitis C Prevalence and Validation of a Clinical Prediction Score for Targeted Screening among People Living with HIV in Ghana. Journal of the International Association of Providers of AIDS Care. 2021;20:23259582211022469-. doi: 10.1177/23259582211022469. PubMed PMID: MEDLINE:34060369.
150. Tounkara A, Sarro YS, Kristensen S, Dao S, Diallo H, Diarra B, et al. Seroprevalence of HIV/HBV coinfection in Malian blood donors. Journal of the International Association of Physicians in AIDS Care (Chicago, Ill : 2002). 2009;8(1):47-51. Epub 2009/02/03. doi: 10.1177/1545109708330118. PubMed PMID: 19182212.
151. Toyé RM, Lô G, Diop-Ndiaye H, Cissé AM, Ndiaye AJS, Kébé-Fall K, et al. Prevalence and molecular characterization of hepatitis B virus infection in HIV-infected children in Senegal. Clin Res Hepatol Gastroenterol. 2021;45(2):101502. Epub 2020/08/24. doi: 10.1016/j.clinre.2020.07.007. PubMed PMID: 32828748.
152. Tremeau-Bravard A, Ogbukagu IC, Ticao CJ, Abubakar JJ. Seroprevalence of hepatitis B and C infection among the HIV-positive population in Abuja, Nigeria. Afr Health Sci. 2012;12(3):312-7. PubMed PMID: rayyan-131265224.
153. Umutesi J, Simmons B, Makuza JD, Dushimiyimana D, Mbituyumuremyi A, Uwimana JM, et al. Prevalence of hepatitis B and C infection in persons living with HIV enrolled in care in Rwanda. JMIR Res Protoc. 2017;17(1):315. PubMed PMID: rayyan-131264677.
154. Uneke CJ, Ogbu O, Inyama PU, Anyanwu GI, Njoku MO, Idoko JH. Prevalence of hepatitis-B surface antigen among blood donors and human immunodeficiency virus-infected patients in Jos, Nigeria. Mem Inst Oswaldo Cruz. 2005;100(1):13-6. PubMed PMID: rayyan-131264744.
155. Utoo BT, Mutihir JT, Ifenne DI. Sero-prevalence rate of Hepatitis B among asymptomatic HIV Seropositive antenatal attendees in a Mission Hospital in Nigeria. Tropical Journal of Obstetrics and Gynaecology. 2012;29(1):22-6. doi: 10.4314/tjog.v29i1.
156. Varo R, Buck WC, Kazembe PN, Phiri S, Andrianarimanana D, Weigel R. Seroprevalence of CMV, HSV-2 and HBV among HIV-Infected Malawian Children: A Cross-sectional Survey. J Trop Pediatr. 2016;62(3):220-6. PubMed PMID: rayyan-131265210.
157. Velen K, Charalambous S, Innes C, Churchyard GJ, Hoffmann CJ. Chronic hepatitis B increases mortality and complexity among HIV-coinfected patients in South Africa: a cohort study. HIV Med. 2016;17(9):702-7. PubMed PMID: rayyan-131262299.
158. Waddell RD, Magesa PM, Pallangyo KJ, Matee M, Bakari M, von Reyn F, et al. Coinfection with HIV and HCV in a blood bank population in Dar es Salaam, Tanzania. J Clin Virol. 2006;36(3):237-8. Epub 2006/05/26. doi: 10.1016/j.jcv.2006.04.002. PubMed PMID: 16723273.
159. Walusansa V, Kagimu M. Screening for hepatitis C among HIV positive patients at Mulago hospital in Uganda. Afr Health Sci. 2009;9(3):143-6. PubMed PMID: rayyan-131265103.
160. Wandeler G, Mulenga L, Hobbins M, Joao C, Sinkala E, Hector J, et al. Absence of Active Hepatitis C Virus Infection in Human Immunodeficiency Virus Clinics in Zambia and

Mozambique. Open Forum Infect Dis. 2016;3(2):ofw049. PubMed PMID: rayyan-131261887.
161. Wandeler G, Mulenga L, Vinikoor MJ, Kovari H, Battegay M, Calmy A, et al. Liver fibrosis in treatment-naive HIV-infected and HIV/HBV co-infected patients: Zambia and Switzerland compared. International Journal of Infectious Diseases. 2016;51:97-102. PubMed PMID: rayyan-131264024.
162. Wandeler G, Musukuma K, Zürcher S, Vinikoor MJ, Llenas-García J, Aly MM, et al. Hepatitis B Infection, Viral Load and Resistance in HIV-Infected Patients in Mozambique and Zambia. PLoS One. 2016;11(3):e0152043. PubMed PMID: rayyan-131263189.
163. Webale MK, Budambula V, Lihana R, Musumba FO, Nyamache AK, Budambula NL, et al. Hepatitis B virus sero-profiles and genotypes in HIV-1 infected and uninfected injection and Non-injection drug users from coastal Kenya. BMC Infect Dis. 2015;15:299. PubMed PMID: rayyan-131263273.
164. Wekesa C, Kirk GD, Aizire J, Benson EM, Karabarinde A, Parkes-Ratanshi R, et al. Prevalence and Factors Associated With Liver Fibrosis Among Adult HIV-Infected Patients Attending Urban and Rural Care Clinics in Uganda. Open Forum Infect Dis. 2020;7(11):ofaa483. PubMed PMID: rayyan-131264597.
165. Weldemhret L, Asmelash T, Belodu R, Gebreegziabiher D. Sero-prevalence of HBV and associated risk factors among HIV positive individuals attending ART clinic at Mekelle hospital, Tigray, Northern Ethiopia. AIDS Res Ther. 2016;13:6. PubMed PMID: rayyan-131265216.
166. Wester CW, Bussmann H, Moyo S, Avalos A, Gaolathe T, Ndwapi N, et al. Serological evidence of HIV-associated infection among HIV-1-infected adults in Botswana. Clin Infect Dis. 2006;43(12):1612-5. Epub 2006/11/17. doi: 10.1086/508865. PubMed PMID: 17109297.
167. Wondimeneh Y, Alem M, Asfaw F, Belyhun Y. HBV and HCV seroprevalence and their correlation with CD4 cells and liver enzymes among HIV positive individuals at University of Gondar Teaching Hospital, Northwest Ethiopia. Virol J. 2013;10:171. PubMed PMID: rayyan-131263071.
168. Xie DD, Li J, Chen JT, Eyi UM, Matesa RA, Obono MM, et al. Seroprevalence of Human Immunodeficiency Virus, Hepatitis B Virus, Hepatitis C Virus, and Treponema pallidum Infections among Blood Donors on Bioko Island, Equatorial Guinea. PLoS One. 2015;10(10):e0139947. Epub 2015/10/09. doi: 10.1371/journal.pone.0139947. PubMed PMID: 26448460; PubMed Central PMCID: PMCPMC4598168.
169. Ya'aba Y, Isu NR, Onoja AJ, Ibrahim K, Mohammed SB, Oladosu P. Prevalence of Hepatitis C Virus (HCV) in healthy adults and Human Immunodeficiency Virus (HIV) infected persons in Abuja, Nigeria. Journal of Phytomedicine and Therapeutics. 2015;15(1):11-23. doi: 10.4314/jopat.v15i1.
170. Ya'aba Y, Mohammed SB, Uba A, Ibrahim K, Oladosu OP. Survey of hepatitis C virus antibodies in HIV patients attending general hospital Suleja, Niger State, Nigeria. Journal of Phytomedicine and Therapeutics. 2017;16(2):120-6. doi: 10.4314/jopat.v16i2.
171. Ya'aba Y, Isu NR, Mohammed SB, Oladepo DK, Ibrahim K, Oladusu P, et al. Prevalence of Hepatitis C Virus (HCV) and Human Immunodeficiency Virus (HIV) Co-Infection Among Pregnant Women Attending Antenatal Clinics in Abuja, Nigeria. Journal of Phytomedicine and Therapeutics. 2009;14. doi: 10.4314/jopat.v14i1.69409.
172. Yakubu A, Hali B, Maiyaki AS. Prevalence and risk factors for hepatitis c virus co-infection among human immunodeficiency virus-infected patients and effect of hepatitis c virus infection on acquired immunodeficiency syndrome cases at baseline. Annals of African medicine. 2021;20(4):297-301. Epub 2021/12/12. doi: 10.4103/aam.aam_65_20. PubMed PMID: 34893569; PubMed Central PMCID: PMCPMC8693737.

173. Yendewa GA, Lakoh S, Yendewa SA, Bangura K, Lawrence H, Patino L, et al. Prevalence of hepatitis B surface antigen and serological markers of other endemic infections in HIV-infected children, adolescents and pregnant women in Sierra Leone: A cross-sectional study. International Journal of Infectious Diseases. 2021;102:45-52. PubMed PMID: CCC:000604702000010.
174. Yendewa GA, Sahr F, Aguilera A, Lakoh S, Sesay M, Deen GF, et al. Seroprevalence of Hepatitis B, Hepatitis C, and Human T-Cell Lymphotropic Virus Infections in HIV-Infected Patients in Sierra Leone. PLoS One. 2019;100(6):1521-4. PubMed PMID: rayyan-131265251.
175. Yimer G, Gry M, Amogne W, Makonnen E, Habtewold A, Petros Z, et al. Evaluation of patterns of liver toxicity in patients on antiretroviral and anti-tuberculosis drugs: a prospective four arm observational study in ethiopian patients. PLoS One. 2014;9(4):e94271. Epub 2014/04/10. doi: 10.1371/journal.pone.0094271. PubMed PMID: 24714066; PubMed Central PMCID: PMCPMC3979833 following conflicts. IS-K is an employee of AstraZeneca Innovative Medicines Personalised Healthcare & Biomarkers, Science for Life Laboratory, which had no influence in the conception, design or analysis of this work, and no role in manuscript preparation or publication. MG is an employee of Former AstraZeneca R&D, Global Safety Assessment, Molecular Toxicology, Södertälje, Sweden, which had no influence in the conception, design or analysis of this work, and no role in manuscript preparation or publication. This does not alter the authors' adherence to all the PLOS ONE policies on sharing data and materials.
176. Zeba MT, Karou SD, Sagna T, Djigma F, Bisseye C, Ouermi D, et al. HCV prevalence and co-infection with HIV among pregnant women in Saint Camille Medical Centre, Ouagadougou. Tropical medicine & international health : TM & IH. 2011;16(11):1392-6. Epub 2011/07/19. doi: 10.1111/j.1365-3156.2011.02845.x. PubMed PMID: 21762293.
177. Zoufaly A, Onyoh EF, Tih PM, Awasom CN, Feldt T. High prevalence of hepatitis B and syphilis co-infections among HIV patients initiating antiretroviral therapy in the north-west region of Cameroon. Int J STD AIDS. 2012;23(6):435-8. PubMed PMID: rayyan-131263461.
178. Abera B, Zenebe Y, Mulu W, Kibret M, Kahsu G. Seroprevalence of hepatitis B and C viruses and risk factors in HIV infected children at the Felgehiwot referral hospital, Ethiopia. BMC Res Notes. 2014;7:838. PubMed PMID: rayyan-131265233.
179. Abreha T, Woldeamanuel Y, Pietsch C, Maier M, Asrat D, Abebe A, et al. Genotypes and viral load of hepatitis C virus among persons attending a voluntary counseling and testing center in Ethiopia. J Med Virol. 2011;83(5):776-82. Epub 2011/02/26. doi: 10.1002/jmv.21788. PubMed PMID: 21351106.
180. Abroug H, Zemni I, Chelly S, Maalel I, Samia GS, Belguith Sriha A, et al. Prevalence of hepatitis B virus infection among pregnant women in Jos, Nigeria. Libyan J Med. 2020;19(3):176-81. PubMed PMID: rayyan-131264707.
181. Adekunle AE, Oladimeji AA, Temi AP, Adeseye AI, Akinyeye OA, Taiwo RH. Baseline CD4+ T lymphocyte cell counts, hepatitis B and C viruses seropositivity in adults with Human Immunodeficiency Virus infection at a tertiary hospital in Nigeria. Pan Afr Med J. 2011;9:6. PubMed PMID: rayyan-131262123.
182. Adesina O, Oladokun A, Akinyemi O, Adedokun B, Awolude O, Odaibo G, et al. Human immuno-deficiency virus and hepatitis B virus coinfection in pregnancy at the University College Hospital, Ibadan. Afr J Med Med Sci. 2010;39(4):305-10. PubMed PMID: rayyan-131263662.
183. Adewole OO, Anteyi E, Ajuwon Z, Wada I, Elegba F, Ahmed P, et al. Hepatitis B and C virus co-infection in Nigerian patients with HIV infection. J Infect Dev Ctries. 2009;3(5):369-75. PubMed PMID: rayyan-131263150.

184. Adewumi MO, Donbraye E, Sule WF, Olarinde O. HBV Infection Among HIV-Infected Cohort and HIV-Negative Hospital Attendees in South Western Nigeria. Afr J Infect Dis. 2015;9(1):14-7. PubMed PMID: rayyan-131263077.
185. Adeyemi OA, Itanyi IU. Sero-prevalence and determinants of Hepatitis B among a cohort of HIV-infected women of reproductive age in Nigeria. 2020;15(9):e0236456. PubMed PMID: rayyan-131265190.
186. Adoga MP, Banwat EB, Forbi JC, Nimzing L, Pam CR, Gyar SD, et al. Human immunonodeficiency virus, hepatitis B virus and hepatitis C virus: sero-prevalence, co-infection and risk factors among prison inmates in Nasarawa State, Nigeria. J Infect Dev Ctries. 2009;3(7):539-47. Epub 2009/09/19. doi: 10.3855/jidc.472. PubMed PMID: 19762972.
187. Agaba PA, Meloni ST, Sule HM, Agbaji OO, Ekeh PN, Job GC, et al. Patients who present late to HIV care and associated risk factors in Nigeria. HIV Med. 2014;15(7):396-405. Epub 2014/03/04. doi: 10.1111/hiv.12125. PubMed PMID: 24580742.
188. Agbaji O, Thio CL, Meloni S, Graham C, Muazu M, Nimzing L, et al. Impact of hepatitis C virus on HIV response to antiretroviral therapy in Nigeria. J Acquir Immune Defic Syndr. 2013;62(2):204-7. PubMed PMID: rayyan-131263742.
189. Agwale SM, Tanimoto L, Womack C, Odama L, Leung K, Duey D, et al. Prevalence of HCV coinfection in HIV-infected individuals in Nigeria and characterization of HCV genotypes. J Clin Virol. 2004;31:S3-6. PubMed PMID: rayyan-131264668.
190. Ahmed SD, Cuevas LE, Brabin BJ, Kazembe P, Broadhead R, Verhoeff FH, et al. Seroprevalence of hepatitis B and C and HIV in Malawian pregnant women. The Journal of infection. 1998;37(3):248-51. Epub 1999/01/19. doi: 10.1016/s0163-4453(98)91983-1. PubMed PMID: 9892528.
191. Ajayi GO, Omilabu SA, Alamu D, Balogun Y, Badaru S. Seroprevalence of other antibodies (herpes, CMV, rubella, varicella, hepatitis B and C, syphilis, chlamydia, mumps, toxoplasmosis) in HIV-positive patients. Clin Exp Obstet Gynecol. 2011;38(2):172-4. PubMed PMID: rayyan-131265294.
192. Akinbami AA, Oshinaike OO, Adeyemo TA, Adediran A, Oshikomaiya BI, Ismail KA. Seroprevalence of hepatitis C infection in HIV patients using a rapid one-step test strip kit. Nig Q J Hosp Med. 2010;20(3):144-6. PubMed PMID: rayyan-131265254.
193. Akinniyi OG, Adetunji SO, Alawode-Obabiyi LA, Japhet MO, Donbraye E. Serological patterns of hepatitis B virus infection among people living with HIV in Ibadan, Nigeria. J Immunoassay Immunochem. 2021;42(4):444-52. Epub 2021/03/23. doi: 10.1080/15321819.2021.1895218. PubMed PMID: 33750262.
194. Alemayehu A, Tassachew Y, Sisay Z, Shimelis T. Prevalence and risk factors of Hepatitis C among individuals presenting to HIV testing centers, Hawassa city, Southern Ethiopia. BMC Res Notes. 2011;4:193. PubMed PMID: rayyan-131264626.
195. Alidjinou EK, Moukassa D, Ebatetou-Ataboho E, Mahoungou GH, Pambou JP, Sané F, et al. Higher levels of hepatitis C virus RNA found in blood donors co-infected with HIV as compared to HCV mono-infected donors. J Infect Dev Ctries. 2014;8(8):1068-71. Epub 2014/08/15. doi: 10.3855/jidc.4767. PubMed PMID: 25116677.
196. Ambassa AC, Djuidje NM, Guiateu TIM, Moundipa FP. Importance of biochemical analysis of the liver function in the management of disease progression in people living with HIV/AIDS and co-infected by HIV and hepatitis B virus in Cameroon. Journal of the Cameroon Academy of Sciences. 2016;13(1-2):13-20. doi: 10.4314/jcas.v13i1-2.
197. Amstutz A. Low rates of active hepatitis B and C infections among adults and children living with HIV and taking antiretroviral therapy: A multicenter screening study in Lesotho. BMJ Open. 2020. PubMed PMID: rayyan-131264060.

198. Andersson MI, Maponga TG, Ijaz S, Barnes J, Theron GB, Meredith SA, et al. The epidemiology of hepatitis B virus infection in HIV-infected and HIV-uninfected pregnant women in the Western Cape, South Africa. Vaccine. 2013;31(47):5579-84. PubMed PMID: rayyan-131262784.
199. Andersson MI, Maponga TG, Ijaz S, Theron G, Preiser W, Tedder RS. High HBV viral loads in HIV-infected pregnant women at a tertiary hospital, South Africa. J Acquir Immune Defic Syndr. 2012;60(4):e111-2. PubMed PMID: rayyan-131263442.
200. Andreotti M, Pirillo MF, Liotta G, Jere H, Maulidi M, Sagno JB, et al. The impact of HBV or HCV infection in a cohort of HIV-infected pregnant women receiving a nevirapine-based antiretroviral regimen in Malawi. BMC Infect Dis. 2014;14:180. PubMed PMID: rayyan-131263736.
201. Anigilaje EA, Olutola A. Human Immunodeficiency Virus And Hepatitis C Virus Co-Infection Among Children In An Antiretroviral Therapy Programme In Benue. The Internet Journal of Infectious Diseases. 2013;12(1).
202. Anyanwu NCJ, Sunmonu PT, Mathew MH. Viral hepatitis B and C co-infection with Human Immunodeficiency Virus among adult patients attending selected highly active anti-retroviral therapy clinics in Nigeria's capital. J Immunoassay Immunochem. 2020;41(2):171-83. PubMed PMID: rayyan-131265680.
203. Archampong TNA, Lartey M, Sagoe KW, Obo-Akwa A, Kenu E, Gillani FS, et al. Proportion and factors associated with Hepatitis B viremia in antiretroviral treatment naive and experienced HIV co-infected Ghanaian patients. BMC infectious diseases. 2016;16:14-. PubMed PMID: rayyan-131264863.
204. Ashir GM, Rabasa AI, Gofama MM, Bukbuk D, Abubakar H, Farouk GA. Study of hepatic functions and prevalence of hepatitis B surface antigenaemia in Nigerian children with human immunodeficiency virus infection. Niger J Med. 2009;18(3):260-2. PubMed PMID: rayyan-131265429.
205. Atina JO, Ogutu EO, Hardison WG, Mumo J. Prevalence of hepatitis A, B, C and human immunodeficiency virus seropositivity among patients with acute icteric hepatitis at the Kenyatta National Hospital, Nairobi. East Afr Med J. 2004;81(4):183-7. PubMed PMID: rayyan-131264670.
206. Attia KA, Eholié S, Messou E, Danel C, Polneau S, Chenal H, et al. Prevalence and virological profiles of hepatitis B infection in human immunodeficiency virus patients. World J Hepatol. 2012;4(7):218-23. PubMed PMID: rayyan-131264638.
207. Augusto A, Augusto O, Taquibo A, Nhachigule C, Siyawadya N, Gudo ES. High frequency of HBV in HIV-infected prisoners in Mozambique. Int J Prison Health. 2019;15(1):58-65. PubMed PMID: rayyan-131263441.
208. Ayana DA, Mulu A, Mihret A, Seyoum B, Aseffa A, Howe R. Hepatitis B virus seromarkers among HIV infected adults on ART: An unmet need for HBV screening in eastern Ethiopia. PLoS One. 2019;14(12):e0226922. PubMed PMID: rayyan-131263271.
209. Ayele W, Nokes DJ, Abebe A, Messele T, Dejene A, Enquselassie F, et al. Higher prevalence of anti-HCV antibodies among HIV-positive compared to HIV-negative inhabitants of Addis Ababa, Ethiopia. J Med Virol. 2002;68(1):12-7. PubMed PMID: rayyan-131263487.
210. Ayelign M, Aynalem M, Berhane N. Hepatitis and HIV Co-infection at University of Gondar Specialized Referral Hospital: Northwest Ethiopia. Hepat Med. 2021;13:113-20. Epub 2021/11/20. doi: 10.2147/hmer.S337817. PubMed PMID: 34795539; PubMed Central PMCID: PMCPMC8593689.
211. Ayuk J, Mphahlele J, Bessong P. Hepatitis B virus in HIV-infected patients in northeastern South Africa: prevalence, exposure, protection and response to HAART. S Afr Med J. 2013;103(5):330-3. PubMed PMID: rayyan-131263238.

212. Bado G, Penot P, N'Diaye MD, Amiel C, Hema A, Kamboulé EB, et al. Hepatitis B seroprevalence in HIV-infected patients consulting in a public day care unit in Bobo Dioulasso, Burkina Faso. Med Mal Infect. 2013;43(5):202-7. PubMed PMID: rayyan-131263199.
213. Balew M, Moges F, Yismaw G, Unakal C. Assessment of hepatitis B virus and hepatitis C virus infections and associated risk factors in HIV infected patients at Debretabor hospital, South Gondar, Northwest Ethiopia. Asian Pac J Trop Dis. 2014;4(1):1-7. doi: 10.1016/S2222-1808(14)60304-2. PubMed PMID: PMC4027354.
214. Balogun TM, Durojaiye IO, Sagoe A, Emmanuel S. Seroepidemiology of hepatitis-B surface antigenaemia in HIV positive patients. West Afr J Med. 2010;29(3):169-73. PubMed PMID: rayyan-131265148.
215. Balogun TM, Emmanuel S, Ojerinde EF. HIV, Hepatitis B and C viruses' coinfection among patients in a Nigerian tertiary hospital. The Pan African medical journal. 2012;12:100-. Epub 2012/08/08. PubMed PMID: 23133700.
216. Barros MF, Piedade J, Nunes G, Canas-Ferreira W, Silva AP, Champalimaud JL, et al. Active replication of hepatitis B virus (HBV) in HIV type 1 and in HIV type 2 infected patients. Rev Inst Med Trop Sao Paulo. 1996;38(4):253-8. PubMed PMID: rayyan-131261917.
217. Barth RE, Huijgen Q, Tempelman HA, Mudrikova T, Wensing AM, Hoepelman AI. Presence of occult HBV, but near absence of active HBV and HCV infections in people infected with HIV in rural South Africa. J Med Virol. 2011;83(6):929-34. PubMed PMID: rayyan-131264557.
218. Baseke J, Musenero M, Mayanja-Kizza H. Prevalence of hepatitis B and C and relationship to liver damage in HIV infected patients attending Joint Clinical Research Centre Clinic (JCRC), Kampala, Uganda. Afr Health Sci. 2015;15(2):322-7. PubMed PMID: rayyan-131264673.
219. Beghin JC, Ruelle J, Sokal E, Bachy A, Krishna M, Hall L, et al. Effectiveness of the South African expanded program of immunization against hepatitis B in children infected with human immunodeficiency virus-1 living in a resource-limited setting of Kwazulu-Natal. J Med Virol. 2017;89(1):182-5. PubMed PMID: rayyan-131262689.
220. Bell TG, Makondo E, Martinson NA, Kramvis A. Hepatitis B virus infection in human immunodeficiency virus infected southern African adults: occult or overt--that is the question. PLoS One. 2012;7(10):e45750. PubMed PMID: rayyan-131263255.
221. Benjelloun S, Bahbouhi B, Sekkat S, Bennani A, Hda N, Benslimane A. Anti-HCV seroprevalence and risk factors of hepatitis C virus infection in Moroccan population groups. Res Virol. 1996;147(4):247-55. Epub 1996/07/01. doi: 10.1016/0923-2516(96)89656-7. PubMed PMID: 8837233.
222. Bessimbaye N, Moussa AM, Mbanga D, Tidjani A, Mahamat SO, Ngawara MN, et al. [Seroprevalence of HBs Ag and of anti-HCV antibodies among HIV infected people in N'Djamena, Chad]. Bull Soc Pathol Exot. 2014;107(5):327-31. PubMed PMID: rayyan-131265212.
223. Bhattacharya D, Guo R, Tseng CH, Emel L, Sun R, Chiu SH, et al. Maternal HBV Viremia and Association With Adverse Infant Outcomes in Women Living With HIV and HBV. Pediatr Infect Dis J. 2021;40(2):e56-e61. PubMed PMID: rayyan-131264115.
224. Bivigou-Mboumba B, François-Souquière S, Deleplancque L, Sica J, Mouinga-Ondémé A, Amougou-Atsama M, et al. Broad Range of Hepatitis B Virus (HBV) Patterns, Dual Circulation of Quasi-Subgenotype A3 and HBV/E and Heterogeneous HBV Mutations in HIV-Positive Patients in Gabon. PLoS One. 2016;11(1):e0143869. PubMed PMID: rayyan-131262191.

225. Boateng R, Mutocheluh M. Sero-prevalence of Hepatitis B and C viral co-infections among HIV-1 infected ART-naïve individuals in Kumasi, Ghana. 2019;14(4):e0215377. PubMed PMID: rayyan-131265227.
226. Bouare N, Vaira D, Gothot A, Delwaide J, Bontems S, Seidel L, et al. Prevalence of HIV and HCV infections in two populations of Malian women and serological assays performances. World J Hepatol. 2012;4(12):365-73. Epub 2013/01/29. doi: 10.4254/wjh.v4.i12.365. PubMed PMID: 23355914; PubMed Central PMCID: PMCPMC3554800.
227. Bowring AL, Luhmann N, Pont S, Debaulieu C, Derozier S, Asouab F, et al. An urgent need to scale-up injecting drug harm reduction services in Tanzania: prevalence of blood-borne viruses among drug users in Temeke District, Dar-es-Salaam, 2011. The International journal on drug policy. 2013;24(1):78-81. Epub 2012/10/06. doi: 10.1016/j.drugpo.2012.08.005. PubMed PMID: 23036650.
228. Brandful JA, Apeagyei FA, Ampofo WK, Adu-Sarkodie Y, Ansah JE, Nuvor V, et al. Relationship between immunoclinical status and prevalence of viral sexually transmitted diseases among human immunodeficiency virus-1 seropositive patients in Ghana. Viral Immunol. 1999;12(2):131-7. Epub 1999/07/21. doi: 10.1089/vim.1999.12.131. PubMed PMID: 10413359.
229. Burnett RJ, Ngobeni JM, François G, Hoosen AA, Leroux-Roels G, Meheus A, et al. Increased exposure to hepatitis B virus infection in HIV-positive South African antenatal women. Int J STD AIDS. 2007;18(3):152-6. PubMed PMID: rayyan-131263806.
230. Buseri FI, Muhibi MA, Jeremiah ZA. Sero-epidemiology of transfusion-transmissible infectious diseases among blood donors in Osogbo, south-west Nigeria. Blood transfusion = Trasfusione del sangue. 2009;7(4):293-9. Epub 2009/12/17. doi: 10.2450/2009.0071-08. PubMed PMID: 20011640; PubMed Central PMCID: PMCPMC2782806.
231. Calisti G, Muhindo R, Boum Y, 2nd, Wilson LA, Foster GM, Geretti AM, et al. Epidemiology of HBV infection in a cohort of Ugandan HIV-infected patients and rate and pattern of lamivudine-resistant HBV infection in patients receiving antiretroviral therapy. Trans R Soc Trop Med Hyg. 2015;109(11):723-9. PubMed PMID: rayyan-131262776.
232. Carimo AA, Gudo ES, Maueia C, Mabunda N, Chambal L, Vubil A, et al. First report of occult hepatitis B infection among ART naive HIV seropositive individuals in Maputo, Mozambique. PloS one. 2018;13(1):e0190775-e. PubMed PMID: rayyan-131262955.
233. Chakraborty R, Rees G, Bourboulia D, Cross AM, Dixon JR, D'Agostino A, et al. Viral coinfections among African children infected with human immunodeficiency virus type 1. Clin Infect Dis. 2003;36(7):922-4. Epub 2003/03/26. doi: 10.1086/368207. PubMed PMID: 12652394.
234. Chambal LM, Gudo ES, Carimo A, Corte Real R, Mabunda N, Maueia C, et al. HBV infection in untreated HIV-infected adults in Maputo, Mozambique. PLoS One. 2017;12(7):e0181836. PubMed PMID: rayyan-131263078.
235. Chasela CS, Wall P, Drobeniuc J, King CC, Teshale E, Hosseinipour MC, et al. Prevalence of hepatitis C virus infection among human immunodeficiency virus-1-infected pregnant women in Malawi: the BAN study. J Clin Virol. 2012;54(4):318-20. PubMed PMID: rayyan-131264734.
236. Cherry CL, Affandi JS, Brew BJ, Creighton J, Djauzi S, Hooker DJ, et al. Hepatitis C seropositivity is not a risk factor for sensory neuropathy among patients with HIV. Neurology. 2010;74(19):1538-42. PubMed PMID: rayyan-131263320.
237. Chiesa A. Hepatitis B and HIV coinfection in Northern Uganda: Is a decline in HBV prevalence on the horizon? PLoS One. 2020;15(11):e0242278. PubMed PMID: rayyan-131263164.

238. Chisenga CC, Musukuma K, Chilengi R, Zürcher S, Munamunungu V, Siyunda A, et al. Field performance of the Determine HBsAg point-of-care test for diagnosis of hepatitis B virus co-infection among HIV patients in Zambia. J Trop Med. 2018;98:5-7. PubMed PMID: rayyan-131262948.
239. Coffie PA, Egger M, Vinikoor MJ, Zannou M, Diero L, Patassi A, et al. Trends in hepatitis B virus testing practices and management in HIV clinics across sub-Saharan Africa. BMC Infect Dis. 2017;17:706. PubMed PMID: rayyan-131265570.
240. Coffie PA, Tchounga BK, Bado G, Kabran M, Minta DK, Wandeler G, et al. Prevalence of hepatitis B and delta according to HIV-type: a multi-country cross-sectional survey in West Africa. PLoS One. 2017;17(1):466. PubMed PMID: rayyan-131264682.
241. Collenberg E, Ouedraogo T, Ganamé J, Fickenscher H, Kynast-Wolf G, Becher H, et al. Seroprevalence of six different viruses among pregnant women and blood donors in rural and urban Burkina Faso: A comparative analysis. J Med Virol. 2006;78(5):683-92. Epub 2006/03/24. doi: 10.1002/jmv.20593. PubMed PMID: 16555290.
242. Combe P, La Ruche G, Bonard D, Ouassa T, Faye-Ketté H, Sylla-Koko F, et al. Hepatitis B and C infections, human immunodeficiency virus and other sexually transmitted infections among women of childbearing age in Côte d'Ivoire, West Africa. Trans R Soc Trop Med Hyg. 2001;95(5):493-6. PubMed PMID: rayyan-131263147.
243. Croce F, Fedeli P, Dahoma M, Dehò L, Ramsan M, Adorni F, et al. Risk factors for HIV/AIDS in a low HIV prevalence site of sub-Saharan Africa. Tropical medicine & international health : TM & IH. 2007;12(9):1011-7. Epub 2007/09/19. doi: 10.1111/j.1365-3156.2007.01880.x. PubMed PMID: 17875012.
244. Cunha L, Plouzeau C, Ingrand P, Gudo JP, Ingrand I, Mondlane J, et al. Use of replacement blood donors to study the epidemiology of major blood-borne viruses in the general population of Maputo, Mozambique. J Med Virol. 2007;79(12):1832-40. Epub 2007/10/16. doi: 10.1002/jmv.21010. PubMed PMID: 17935167.
245. Dahoma M, Johnston LG, Holman A, Miller LA, Mussa M, Othman A, et al. HIV and related risk behavior among men who have sex with men in Zanzibar, Tanzania: results of a behavioral surveillance survey. AIDS and behavior. 2011;15(1):186-92. Epub 2009/12/10. doi: 10.1007/s10461-009-9646-7. PubMed PMID: 19997862.
246. Day SL, Odem-Davis K, Mandaliya KN, Jerome KR, Cook L, Masese LN, et al. Prevalence, clinical and virologic outcomes of hepatitis B virus co-infection in HIV-1 positive Kenyan women on antiretroviral therapy. PLoS One. 2013;8(3):e59346. PubMed PMID: rayyan-131264817.
247. Demir M, Phiri S, Heger E, Heller T, Kaiser R, Chaweza T, et al. Prevalence of Anti-HBs Without Anti-HBc Among HIV-Infected Adults Initiating Antiretroviral Therapy in Lilongwe, Malawi. J Acquir Immune Defic Syndr. 2018;78(3):e14-e5. PubMed PMID: rayyan-131264646.
248. Deressa T, Damtie D, Fonseca K, Gao S, Abate E, Alemu S, et al. The burden of hepatitis B virus (HBV) infection, genotypes and drug resistance mutations in human immunodeficiency virus-positive patients in Northwest Ethiopia. PLoS One. 2017;12(12):e0190149. PubMed PMID: rayyan-131262196.
249. Diale Q, Pattinson R, Chokoe R, Masenyetse L, Mayaphi S. Antenatal screening for hepatitis B virus in HIV-infected and uninfected pregnant women in the Tshwane district of South Africa. S Afr Med J. 2015;106(1):97-100. PubMed PMID: rayyan-131261986.
250. Diarra M, Konate A, Minta D, Sounko A, Dembele M, Toure CS, et al. [Epidemiologic aspects of human immunodeficiency virus and hepatitis virus infections]. Mali Med. 2006;21(2):27-30. PubMed PMID: rayyan-131262756.
251. Diop-Ndiaye H, Touré-Kane C, Etard JF, Lô G, Diaw P, Ngom-Gueye NF, et al. Hepatitis B, C seroprevalence and delta viruses in HIV-1 Senegalese patients at HAART

initiation (retrospective study). J Med Virol. 2008;80(8):1332-6. PubMed PMID: rayyan-131263285.
252. Diro E, Alemu S, A GY. Blood safety & prevalence of transfussion transmissible viral infections among donors at the Red Cross Blood Bank in Gondar University Hospital. Ethiopian medical journal. 2008;46(1):7-13. Epub 2008/08/21. PubMed PMID: 18711984.
253. Diwe CK, Okwara EC, Enwere OO, Azike JE, Nwaimo NC. Sero-prevalence of hepatitis B virus and hepatitis C virus among HIV patients in a suburban University Teaching Hospital in South-East Nigeria. Pan Afr Med J. 2013;16:7. PubMed PMID: rayyan-131265241.
254. Dovonou CA, Amidou SA, Kpangon AA, Traoré YA, Godjedo TP, Satondji AJ, et al. [Prevalence of hepatitis B in people infected with HIV in Parakou in Benin]. Pan Afr Med J. 2015;20:125. PubMed PMID: rayyan-131264692.
255. du Plessis R, Webber L, Saayman G. Bloodborne viruses in forensic medical practice in South Africa. The American journal of forensic medicine and pathology. 1999;20(4):364-8. Epub 2000/01/07. doi: 10.1097/00000433-199912000-00010. PubMed PMID: 10624931.
256. Dziuban EJ, Marton SA, Hughey AB, Mbingo TL, Draper HR, Schutze GE. Seroprevalence of hepatitis B in a cohort of HIV-infected children and adults in Swaziland. Int J STD AIDS. 2013;24(7):561-5. PubMed PMID: rayyan-131265237.
257. Ejele OA, Nwauche CA, Erhabor O. The prevalence of hepatitis B surface antigenaemia in HIV positive patients in the Niger Delta Nigeria. Niger J Med. 2004;13(2):175-9. PubMed PMID: rayyan-131264702.
258. Ekouevi DK, Coffie PA, Tchounga BK, Poda A, Jaquet A, Dabis F, et al. Prevalence of hepatitis C among HIV-1, HIV-2 and dually reactive patients: A multi-country cross-sectional survey in West Africa. J Public Health Afr. 2018;9(2):871. PubMed PMID: rayyan-131264720.
259. Elsharkawy A, Alem SA, Cordie A, Mohamed R, Meshaal S, Esmat G. Current status of hepatitis C virus among people living with human immunodeficiency virus in Egypt. Trans R Soc Trop Med Hyg. 2021. Epub 2021/12/02. doi: 10.1093/trstmh/trab176. PubMed PMID: 34850231.
260. Eze JC, Ibeziako NS, Ikefuna AN, Nwokoye IC, Uleanya ND, Ilechukwu GC. Prevalence and Risk Factors for Hepatitis C and Human Immunodeficiency Virus Coinfection Among Children in Enugu, Nigeria. African Journal of Infectious Diseases. 2014;8(1):5-8.
261. Ezechi OC, Kalejaiye OO, Gab-Okafor CV, Oladele DA, Oke BO, Musa ZA, et al. Sero-prevalence and factors associated with Hepatitis B and C co-infection in pregnant Nigerian women living with HIV infection. Pan Afr Med J. 2014;17:197. Epub 2014/11/15. doi: 10.11604/pamj.2014.17.197.2310. PubMed PMID: 25396023; PubMed Central PMCID: PMCPMC4229000.
262. Feldt T, Sarfo FS, Zoufaly A, Phillips RO, Burchard G, van Lunzen J, et al. Hepatitis E virus infections in HIV-infected patients in Ghana and Cameroon. J Clin Virol. 2013;58(1):18-23. PubMed PMID: rayyan-131263396.
263. Firnhaber C, Reyneke A, Schulze D, Malope B, Maskew M, MacPhail P, et al. The prevalence of hepatitis B co-infection in a South African urban government HIV clinic. S Afr Med J. 2008;98(7):541-4. PubMed PMID: rayyan-131264689.
264. Forbi JC, Gabadi S, Alabi R, Iperepolu HO, Pam CR, Entonu PE, et al. The role of triple infection with hepatitis B virus, hepatitis C virus, and human immunodeficiency virus (HIV) type-1 on CD4+ lymphocyte levels in the highly HIV infected population of North-Central Nigeria. Mem Inst Oswaldo Cruz. 2007;102(4):535-7. PubMed PMID: rayyan-131265077.

265. Franzeck FC, Ngwale R, Msongole B, Hamisi M, Abdul O, Henning L, et al. Viral hepatitis and rapid diagnostic test based screening for HBsAg in HIV-infected patients in rural Tanzania. PLoS One. 2013;8(3):e58468. PubMed PMID: rayyan-131265679.
266. Frempong MT, Ntiamoah P, Annani-Akollor ME. Hepatitis B and C infections in HIV-1 and non-HIV infected pregnant women in the Brong-Ahafo Region, Ghana. 2019;14(7):e0219922. PubMed PMID: rayyan-131263146.
267. Gededzha MP, Mphahlele MJ, Lukhwareni A, Selabe SG. Should routine serological screening for HCV be mandatory in HIV/AIDS patients enrolling for HAART in South Africa? S Afr Med J. 2010;100(12):814-5. PubMed PMID: rayyan-131265355.
268. Gededzha MP, Sondlane TH, Malinga LA, Burnett RJ, Lebelo RL, Blackard JT, et al. Molecular characterization of hepatitis B virus X gene in HIV-positive South Africans. Virus Genes. 2018;54(2):190-8. PubMed PMID: rayyan-131264186.
269. Gedefie A, Adamu A, Alemayehu E, Kassa Y, Belete MA. Hepatitis C Virus Infection among HIV-Infected Patients Attending Dessie Referral Hospital, Northeastern Ethiopia. International journal of microbiology. 2021;2021:6675851-. doi: 10.1155/2021/6675851. PubMed PMID: MEDLINE:33552160.
270. George CAA, Adetutu OJ, Oseni-Momodu E. Hepatitis B and C coinfection with human immunodeficiency virus in a tertiary centre in Jos, North Central, Nigeria. Nigerian Journal of Family Practice. 2018;9(1):22-8.
271. Geretti AM, Patel M, Sarfo FS, Chadwick D, Verheyen J, Fraune M, et al. Detection of highly prevalent hepatitis B virus coinfection among HIV-seropositive persons in Ghana. J Clin Microbiol. 2010;48(9):3223-30. PubMed PMID: rayyan-131262544.
272. Giuliano M, Pirillo MF, Lucaroni F, Liotta G, Andreotti M, Mancinelli S, et al. Lack of new HBV infections over 2 years of follow-up in HIV-positive women receiving ART up to 6 or 24 months after delivery. J Infect Dev Ctries. 2018;12(5):394-6. PubMed PMID: rayyan-131263981.
273. Goa A, Dana T, Bitew S, Arba A. Seroprevalence and associated factors of hepatitis B virus infection among HIV-positive adults attending an antiretroviral treatment clinic at Wolaita Sodo University Referral Hospital. Hepat Med. 2019;11:137-47. PubMed PMID: rayyan-131265182.
274. Gogela NA, Sonderup MW, Rebe K, Chivese T, Spearman CW. Hepatitis C prevalence in HIV-infected heterosexual men and men who have sex with men. S Afr Med J. 2018;108(7):568-72. PubMed PMID: rayyan-131263318.
275. Goverwa-Sibanda TP, Mupanguri C, Timire C, Harries AD, Ngwenya S, Chikwati E, et al. Hepatitis B infection in people living with HIV who initiate antiretroviral therapy in Zimbabwe. Public Health Action. 2020;10(3):97-103. PubMed PMID: rayyan-131263187.
276. Greer AE, Ou SS, Wilson E, Piwowar-Manning E, Forman MS, McCauley M, et al. Comparison of Hepatitis B Virus Infection in HIV-Infected and HIV-Uninfected Participants Enrolled in a Multinational Clinical Trial: HPTN 052. J Acquir Immune Defic Syndr. 2017;76(4):388-93. PubMed PMID: rayyan-131262411.
277. Gudo ES, Maueia C, Mabunda N, Chambal L, Vubil A, Flora A, et al. Hepatitis B infection among HIV infected individuals in Gabon: Occult hepatitis B enhances HBV DNA prevalence. PLoS One. 2018;13(1):e0190592. PubMed PMID: rayyan-131263180.
278. Guimarães Nebenzahl H, Lopes A, Castro R, Pereira F. Prevalence of human immunodeficiency virus, hepatitis C virus, hepatitis B virus and syphilis among individuals attending anonymous testing for HIV in Luanda, Angola. South African Medical Journal = Suid-Afrikaanse Tydskrif Vir Geneeskunde. 2013;103(3):186-8. doi: 10.7196/samj.6097.
279. Hadush H, Gebre-Selassie S, Mihret A. Hepatitis C virus and human immunodeficiency virus coinfection among attendants of voluntary counseling and testing

centre and HIV follow up clinics in Mekelle Hospital. Pan Afr Med J. 2013;14:107. PubMed PMID: rayyan-131263334.
280. Harania RS, Karuru J, Nelson M, Stebbing J. HIV, hepatitis B and hepatitis C coinfection in Kenya. Aids. 2008;22(10):1221-2. Epub 2008/06/06. doi: 10.1097/QAD.0b013e32830162a8. PubMed PMID: 18525268.
281. Harvard K, Hanley M, Gosling R, Chen I, Akindigh TM. Seroprevalence of hepatitis B virus co-infection among HIV-1-positive patients in North-Central Nigeria: The urgent need for surveillance. Malar J. 2019;8(1):622. PubMed PMID: rayyan-131265243.
282. Hawkins C, Christian B, Ye J, Nagu T, Aris E, Chalamilla G, et al. Prevalence of hepatitis B co-infection and response to antiretroviral therapy among HIV-infected patients in Tanzania. Aids. 2013;27(6):919-27. PubMed PMID: rayyan-131264688.
283. Hector J, Vinikoor M, Chilengi R, Ehmer J, Egger M, Wandeler G. No Impact of Hepatitis B Virus Infection on Early Mortality Among Human Immunodeficiency Virus-Infected Patients in Southern Africa. Clin Infect Dis. 2018;67(8):1310-1. PubMed PMID: rayyan-131264292.
284. Hoffmann CJ, Charalambous S, Martin DJ, Innes C, Churchyard GJ, Chaisson RE, et al. Hepatitis B virus infection and response to antiretroviral therapy (ART) in a South African ART program. Clin Infect Dis. 2008;47(11):1479-85. Epub 2008/10/22. doi: 10.1086/593104. PubMed PMID: 18937580; PubMed Central PMCID: PMCPMC2670447.
285. Hoffmann CJ, Charalambous S, Thio CL, Martin DJ, Pemba L, Fielding KL, et al. Hepatotoxicity in an African antiretroviral therapy cohort: the effect of tuberculosis and hepatitis B. Aids. 2007;21(10):1301-8. Epub 2007/06/05. doi: 10.1097/QAD.0b013e32814e6b08. PubMed PMID: 17545706.
286. Hoffmann CJ, Dayal D, Cheyip M, McIntyre JA, Gray GE, Conway S, et al. Prevalence and associations with hepatitis B and hepatitis C infection among HIV-infected adults in South Africa. Int J STD AIDS. 2012;23(10):e10-3. PubMed PMID: rayyan-131264573.
287. Hoffmann CJ, Mashabela F, Cohn S, Hoffmann JD, Lala S, Martinson NA, et al. Maternal hepatitis B and infant infection among pregnant women living with HIV in South Africa. J Int AIDS Soc. 2014;17(1):18871. PubMed PMID: rayyan-131264117.
288. Hønge B, Jespersen S, Medina C, Té D, da Silva Z, Ostergaard L, et al. Hepatitis B virus surface antigen and anti-hepatitis C virus rapid tests underestimate hepatitis prevalence among HIV-infected patients. HIV Med. 2014;15(9):571-6. PubMed PMID: rayyan-131263275.
289. Hønge BL, Jespersen S, Medina C, da Silva Té D, da Silva ZJ, Lewin SR, et al. Hepatitis C prevalence among HIV-infected patients in Guinea-Bissau: a descriptive cross-sectional study. Int J Infect Dis. 2014;28:35-40. PubMed PMID: rayyan-131263316.
290. Hønge BL, Jespersen S, Medina C, S TD, da Silva ZJ, Lewin S, et al. Hepatitis B and Delta virus are prevalent but often subclinical co-infections among HIV infected patients in Guinea-Bissau, West Africa: a cross-sectional study. PLoS One. 2014;9(6):e99971. PubMed PMID: rayyan-131263160.
291. Houghtaling L, Moh R, Abdou Chekaraou M, Gabillard D, Anglaret X, Eholié SP, et al. CD4+ T Cell Recovery and Hepatitis B Virus Coinfection in HIV-Infected Patients from Côte d'Ivoire Initiating Antiretroviral Therapy. AIDS Res Hum Retroviruses. 2018;34(5):439-45. PubMed PMID: rayyan-131262229.
292. Ifeorah IM, Bakarey AS, Adeniji JA, Onyemelukwe FN. Seroprevalence of hepatitis B and delta viruses among HIV-infected population attending anti-retroviral clinic in selected health facilities in Abuja, Nigeria. J Immunoassay Immunochem. 2017;38(6):608-19. PubMed PMID: rayyan-131265236.

293. Ikomey GM, Jacobs GB, Tanjong B, Mesembe MT, Eyoh A, Lyonga E, et al. Evidence of co and triple infections of Hepatitis B and C amongst HIV infected pregnant women in Buea, Cameroon. Health Sci Dis. 2016;17(2).
294. Ikpeme EE, Etukudo OM, Ekrikpo UE. Seroprevalence of HBV and HIV co-infection in children and outcomes following highly active antiretroviral therapy (HAART) in Uyo, South-South Nigeria. Afr Health Sci. 2013;13(4):955-61. PubMed PMID: rayyan-131265217.
295. Ilboudo D, Karou D, Nadembega WM, Savadogo A, Djeneba O, Pignatelli S, et al. Prevalence of human herpes virus-8 and hepatitis B virus among HIV seropositive pregnant women enrolled in the Mother-to-Child HIV Transmission Prevention Program at Saint Camille Medical Centre in Burkina Faso. Pak J Biol Sci. 2007;10(17):2831-7. PubMed PMID: rayyan-131264766.
296. Inyama P, Uneke C, Anyanwu G, Njoku M, Idoko J, Idoko J. Prevalence of antibodies to Hepatitis C virus among Nigerian patients with HIV infection. Online Journal of Health & Allied Sciences. 2005;4.
297. Ive P, MacLeod W, Mkumla N, Orrell C, Jentsch U, Wallis CL, et al. Low prevalence of liver disease but regional differences in HBV treatment characteristics mark HIV/HBV co-infection in a South African HIV clinical trial. PLoS One. 2013;8(12):e74900. PubMed PMID: rayyan-131264056.
298. Iwalokun BA, Hodonu SO, Olaleye BM, Olabisi OA. Seroprevalence and biochemical features of hepatitis B surface antigenemia in patients with HIV-1 infection in Lagos, Nigeria. Afr J Med Med Sci. 2006;35(3):337-43. PubMed PMID: rayyan-131265186.
299. Jackson JB, Guay L, Goldfarb J, Olness K, Ndugwa C, Mmiro F, et al. Hepatitis C virus antibody in HIV-1 infected Ugandan mothers. Lancet. 1991;337(8740):551. PubMed PMID: rayyan-131263339.
300. Jaquet A, Wandeler G, Nouaman M, Ekouevi DK, Tine J, Patassi A, et al. Alcohol use, viral hepatitis and liver fibrosis among HIV-positive persons in West Africa: a cross-sectional study. J Int AIDS Soc. 2017;19(1):21424. PubMed PMID: rayyan-131261969.
301. Jobarteh M, Malfroy M, Peterson I, Jeng A, Sarge-Njie R, Alabi A, et al. Seroprevalence of hepatitis B and C virus in HIV-1 and HIV-2 infected Gambians. Virol J. 2010;7:230. PubMed PMID: rayyan-131265229.
302. Jooste P, van Zyl A, Adland E, Daniels S, Hattingh L, Brits A, et al. Screening, characterisation and prevention of Hepatitis B virus (HBV) co-infection in HIV-positive children in South Africa. J Clin Virol. 2016;85:71-4. PubMed PMID: rayyan-131265122.
303. Joseph F, Rodrigue KW, Serges T, Salomon NP, Christian TN, Carlos TTM, et al. Hepatitis B infection and risk factors among children living with HIV in Yaounde, Cameroon: an integrated management. BMC Pediatr. 2019;19(1):366. PubMed PMID: rayyan-131263182.
304. Kaba D, Bangoura MA, Sylla MM, Sako FB, Diallo MS, Diallo I, et al. Prevalence and factors associated with hepatitis B in a cohort of HIV-infected children in the Pediatric Department at Donka National Hospital, Guinea. Pan Afr Med J. 2019;34:182. PubMed PMID: rayyan-131264595.
305. Kallestrup P, Zinyama R, Gomo E, Dickmeiss E, Platz P, Gerstoft J, et al. Low prevalence of hepatitis C virus antibodies in HIV-endemic area of Zimbabwe support sexual transmission as the major route of HIV transmission in Africa. Aids. 2003;17(9):1400-2. Epub 2003/06/12. doi: 10.1097/00002030-200306130-00019. PubMed PMID: 12799566.
306. Kamenya T, Damian DJ, Ngocho JS, Philemon RN, Mahande MJ, Msuya SE. The prevalence of hepatitis B virus among HIV-positive patients at Kilimanjaro Christian Medical Centre Referral Hospital, Northern Tanzania. Pan Afr Med J. 2017;28:275. PubMed PMID: rayyan-131264704.

307. Kania D, Sangaré L, Sakandé J, Koanda A, Nébié YK, Zerbo O, et al. A new strategy to improve the cost-effectiveness of human immunodeficiency virus, hepatitis B virus, hepatitis C virus, and syphilis testing of blood donations in sub-Saharan Africa: a pilot study in Burkina Faso. Transfusion. 2009;49(10):2237-40. Epub 2009/11/12. doi: 10.1111/j.1537-2995.2009.02276.x. PubMed PMID: 19903285.
308. Kapembwa KC, Goldman JD, Lakhi S, Banda Y, Bowa K, Vermund SH, et al. HIV, Hepatitis B, and Hepatitis C in Zambia. Journal of global infectious diseases. 2011;3(3):269-74. Epub 2011/09/03. doi: 10.4103/0974-777x.83534. PubMed PMID: 21887060; PubMed Central PMCID: PMCPMC3162815.
309. Kashala O, Mubikayi L, Kayembe K, Mukeba P, Essex M. Hepatitis B virus activation among central Africans infected with human immunodeficiency virus (HIV) type 1: pre-s2 antigen is predominantly expressed in HIV infection. J Infect Dis. 1994;169(3):628-32. PubMed PMID: rayyan-131263221.
310. Katusiime C, Schlech WF, 3rd, Parkes-Ratanshi R, Sempa J, Kambugu A. Characteristics of Sexually Transmitted Infections among High-Risk HIV-Positive Patients Attending an Urban Clinic in Uganda. J Int Assoc Provid AIDS Care. 2016;15(1):36-41. PubMed PMID: rayyan-131262267.
311. Kfutwah AK, Tejiokem MC, Njouom R. A low proportion of HBeAg among HBsAg-positive pregnant women with known HIV status could suggest low perinatal transmission of HBV in Cameroon. Virol J. 2012;9:62. PubMed PMID: rayyan-131264058.
312. Kibaya RM, Lihana RW, Kiptoo M, Songok EM, Ng'ang'a Z, Osman S, et al. Characterization of HBV Among HBV/HIV-1 Co-Infected Injecting Drug Users from Mombasa, Kenya. Curr HIV Res. 2015;13(4):292-9. PubMed PMID: rayyan-131262274.
313. Kilani B, Ammari L, Marrakchi C, Letaief A, Chakroun M, Ben Jemaa M, et al. Seroepidemiology of HCV-HIV coinfection in Tunisia. La Tunisie medicale. 2007;85(2):121-3. Epub 2007/08/02. PubMed PMID: 17665657.
314. Kilonzo SB, Gunda DW. Liver Fibrosis and Hepatitis B Coinfection among ART Naïve HIV-Infected Patients at a Tertiary Level Hospital in Northwestern Tanzania: A Cross-Sectional Study. 2017;2017:5629130. PubMed PMID: rayyan-131264021.
