# Supplementary material for: Epidemiology of hepatitis B virus and/or hepatitis C virus infections among people living with human immunodeficiency virus in Africa: A systematic review and meta-analysis
Source: PLoS One. 2022 May 31;17(5):e0269250. doi: 10.1371/journal.pone.0269250 (PMC9154112; doi:10.1371/journal.pone.0269250)

S4 Fig. Prevalence estimate of hepatitis C virus infection in PLHIV in Africa.

Study	Positive	Total	Prevalence (%)	95% CI	Weight
**HCV_Anti−HCV**					
Abera_Ethiopia	14	253	5.53	[3.06; 9.11]	0.5%
Abreha_Ethiopia	50	734	6.81	[5.10; 8.88]	0.5%
Adekunle_Nigeria	2	273	0.73	[0.09; 2.62]	0.5%
Adesina_Nigeria	29	1575	1.84	[1.24; 2.63]	0.5%
Adewole_Nigeria	6	260	2.31	[0.85; 4.95]	0.5%
Adoga_Nigeria	2	54	3.70	[0.45; 12.75]	0.4%
Agaba_Nigeria	1934	14487	13.35	[12.80; 13.91]	0.5%
Agbaji_Nigeria	183	1352	13.54	[11.76; 15.48]	0.5%
Agbaji_Nigeria	79	1431	5.52	[4.39; 6.83]	0.5%
Ahmed_Malawi	6	50	12.00	[4.53; 24.31]	0.4%
Ajayi_Nigeria	2	273	0.73	[0.09; 2.62]	0.5%
Akinbami_Nigeria	9	267	3.37	[1.55; 6.30]	0.5%
Alemayehu_Ethiopia	42	400	10.50	[7.67; 13.93]	0.5%
Alidjinou_Republic of the Congo	30	392	7.65	[5.22; 10.75]	0.5%
Amstutz_Lesotho	6	162	3.70	[1.37; 7.89]	0.5%
Amstutz_Lesotho	22	1318	1.67	[1.05; 2.52]	0.5%
Andreotti_Malawi	8	309	2.59	[1.12; 5.04]	0.5%
Anigilaje_Nigeria	9	395	2.28	[1.05; 4.28]	0.5%
Anyanwu_Nigeria	12	311	3.86	[2.01; 6.64]	0.5%
Atina_Kenya	0	26	0.00	[0.00; 13.23]	0.3%
Ayele_Ethiopia	79	4591	1.72	[1.36; 2.14]	0.5%
Ayelign_Ethiopia	7	81	8.64	[3.55; 17.00]	0.4%
Balew_Ethiopia	5	395	1.27	[0.41; 2.93]	0.5%
Balogun_Nigeria	15	102	14.71	[8.47; 23.09]	0.5%
Barth_South Africa	2	242	0.83	[0.10; 2.95]	0.5%
Baseke_Uganda	5	89	5.62	[1.85; 12.63]	0.4%
Benjelloun_Morocco	23	116	19.83	[13.00; 28.25]	0.5%
Bessimbaye_Chad	7	725	0.97	[0.39; 1.98]	0.5%
Bivigou−Mboumba_Gabon	56	762	7.35	[5.60; 9.44]	0.5%
Boateng_Ghana	22	40	55.00	[38.49; 70.74]	0.4%
Bouare_Mali	0	14	0.00	[0.00; 23.16]	0.2%
Bouare_Mali	0	41	0.00	[0.00; 8.60]	0.4%
Bowring_Tanzania	2	19	10.53	[1.30; 33.14]	0.3%
Bowring_Tanzania	45	93	48.39	[37.89; 58.99]	0.5%
Brandful_Ghana	15	182	8.24	[4.69; 13.23]	0.5%
Buseri_Nigeria	4	44	9.09	[2.53; 21.67]	0.4%
Cherry_South Africa	1	300	0.33	[0.01; 1.84]	0.5%
Combe_Ivory Coast	7	223	3.14	[1.27; 6.36]	0.5%
Cunha_Mozambique	3	217	1.38	[0.29; 3.99]	0.5%
Dahoma_Tanzania	25	65	38.46	[26.65; 51.36]	0.4%
Diarra_Mali	23	100	23.00	[15.17; 32.49]	0.5%
Diop−Ndiaye_Senegal	29	362	8.01	[5.43; 11.30]	0.5%
Diro_Ethiopia	6	27	22.22	[8.62; 42.26]	0.3%
Diwe_Nigeria	3	404	0.74	[0.15; 2.15]	0.5%
du Plessis_South Africa	1	30	3.33	[0.08; 17.22]	0.3%
Ekouevi_Burkina Faso	18	232	7.76	[4.66; 11.98]	0.5%
Ekouevi_Ivory Coast	12	535	2.24	[1.16; 3.89]	0.5%
Elsharkawy_Egypt	349	1004	34.76	[31.81; 37.80]	0.5%
Eze_Nigeria	8	118	6.78	[2.97; 12.92]	0.5%
Ezechi_Nigeria	37	2391	1.55	[1.09; 2.13]	0.5%
Feldt_Cameroon	5	200	2.50	[0.82; 5.74]	0.5%
Feldt_Cameroon	7	279	2.51	[1.01; 5.10]	0.5%
Forbi_Nigeria	20	180	11.11	[6.92; 16.64]	0.5%
Franzeck_Tanzania	10	272	3.68	[1.78; 6.66]	0.5%
Frempong_Ghana	6	148	4.05	[1.50; 8.61]	0.5%
Gededzha_South Africa	8	653	1.23	[0.53; 2.40]	0.5%
Gedefie_Ethiopia	13	249	5.22	[2.81; 8.76]	0.5%
George_Nigeria	95	2224	4.27	[3.47; 5.20]	0.5%
Gogela_South Africa	17	500	3.40	[1.99; 5.39]	0.5%
Guimarães _Angola	4	38	10.53	[2.94; 24.80]	0.4%
Hadush_Ethiopia	16	174	9.20	[5.35; 14.50]	0.5%
Harania_Kenya	4	378	1.06	[0.29; 2.69]	0.5%
Hoffmann_South Africa	1	981	0.10	[0.00; 0.57]	0.5%
Hoffmann_South Africa	0	53	0.00	[0.00; 6.72]	0.4%
Hoffmann_South Africa	0	50	0.00	[0.00; 7.11]	0.4%
Hønge_Guinea−Bissau	4	393	1.02	[0.28; 2.59]	0.5%
Ikomey_Cameroon	3	32	9.38	[1.98; 25.02]	0.3%
Inyama_Nigeria	28	490	5.71	[3.83; 8.15]	0.5%
Jackson_Uganda	0	96	0.00	[0.00; 3.77]	0.5%
Jaquet_Ivory Coast	1	348	0.29	[0.01; 1.59]	0.5%
Jaquet_Senegal	3	211	1.42	[0.29; 4.10]	0.5%
Jaquet_Togo	5	248	2.02	[0.66; 4.64]	0.5%
Kallestrup_Zimbabwe	1	124	0.81	[0.02; 4.41]	0.5%
Kania_Burkina Faso	3	49	6.12	[1.28; 16.87]	0.4%
Kapembwa_Zambia	4	323	1.24	[0.34; 3.14]	0.5%
Kilani_Tunisia	144	362	39.78	[34.70; 45.03]	0.5%
Kirakoya−Samadoulougou_Burkina Faso	73	761	9.59	[7.59; 11.91]	0.5%
Kubio_Ghana	4	33	12.12	[3.40; 28.20]	0.3%
Kwofie_Ghana	3	200	1.50	[0.31; 4.32]	0.5%
Ladep_Nigeria	90	1044	8.62	[6.99; 10.49]	0.5%
Ladep_Nigeria	2014	17882	11.26	[10.80; 11.74]	0.5%
Lassey_Ghana	1	17	5.88	[0.15; 28.69]	0.3%
Laurent_Cameroon	28	169	16.57	[11.30; 23.05]	0.5%
Laurent_Cameroon	3	35	8.57	[1.80; 23.06]	0.4%
Laurent_Democratic Republic of the Congo	2	31	6.45	[0.79; 21.42]	0.3%
Laurent_Democratic Republic of the Congo	29	390	7.44	[5.04; 10.50]	0.5%
Lawal_Nigeria	1	187	0.53	[0.01; 2.94]	0.5%
Lepretre_Senegal	13	22	59.09	[36.35; 79.29]	0.3%
Lesi_Nigeria	7	120	5.83	[2.38; 11.65]	0.5%
Loaarec_Kenya	11	4851	0.23	[0.11; 0.41]	0.5%
Loaarec_Malawi	2	365	0.55	[0.07; 1.97]	0.5%
Loaarec_Mozambique	30	2600	1.15	[0.78; 1.64]	0.5%
Loaarec_Uganda	18	7500	0.24	[0.14; 0.38]	0.5%
Lodenyo_South Africa	1	100	1.00	[0.03; 5.45]	0.5%
Maaref_Tunisia	33	125	26.40	[18.92; 35.03]	0.5%
Mabayoje_Nigeria	12	80	15.00	[8.00; 24.74]	0.4%
Mabayoje_Nigeria	65	280	23.21	[18.40; 28.61]	0.5%
Mabeya_Kenya	30	300	10.00	[6.85; 13.97]	0.5%
Manyazewal_Ethiopia	18	500	3.60	[2.15; 5.63]	0.5%
Mavenyengwa_Namibia	1	75	1.33	[0.03; 7.21]	0.4%
Mbanya_Cameroon	2	20	10.00	[1.23; 31.70]	0.3%
Mboto_Gambia	9	101	8.91	[4.16; 16.24]	0.5%
Mboto_Gambia	9	101	8.91	[4.16; 16.24]	0.5%
Mirzoyan_Libya	283	294	96.26	[93.40; 98.12]	0.5%
Moore_Malawi	22	300	7.33	[4.65; 10.89]	0.5%
Mphahlele_South Africa	3	160	1.88	[0.39; 5.38]	0.5%
Msuya_Tanzania	1	44	2.27	[0.06; 12.02]	0.4%
Mudawi_Sudan	6	358	1.68	[0.62; 3.61]	0.5%
Mugusi S_Tanzania	12	387	3.10	[1.61; 5.35]	0.5%
Mullis_Uganda	31	500	6.20	[4.25; 8.69]	0.5%
Mulu_Ethiopia	51	269	18.96	[14.45; 24.16]	0.5%
Munyemana_Rwanda	52	417	12.47	[9.46; 16.03]	0.5%
Muriuki_Kenya	30	300	10.00	[6.85; 13.97]	0.5%
Muro_Tanzania	0	157	0.00	[0.00; 2.32]	0.5%
Musa_Nigeria	13	132	9.85	[5.35; 16.25]	0.5%
Musyoki_South Africa	8	34	23.53	[10.75; 41.17]	0.4%
Mwatelah_Kenya	24	133	18.05	[11.92; 25.65]	0.5%
Nagalo_Burkina Faso	16	100	16.00	[9.43; 24.68]	0.5%
Nagalo_Burkina Faso	35	433	8.08	[5.69; 11.06]	0.5%
Nagu_Tanzania	47	260	18.08	[13.60; 23.30]	0.5%
Naniche_Mozambique	8	132	6.06	[2.65; 11.59]	0.5%
Ndjoyi−Mbiguino_Gabon	14	491	2.85	[1.57; 4.74]	0.5%
Newton_Nigeria	30	200	15.00	[10.35; 20.72]	0.5%
Nkengasong_Cameroon	12	80	15.00	[8.00; 24.74]	0.4%
Nnakenyi_Nigeria	219	4663	4.70	[4.11; 5.34]	0.5%
Noubiap_Cameroon	38	51	74.51	[60.37; 85.67]	0.4%
Noubiap_Cameroon	3	22	13.64	[2.91; 34.91]	0.3%
Ntagirabiri_Burundi	112	1053	10.64	[8.84; 12.66]	0.5%
Nyirenda_Malawi	8	172	4.65	[2.03; 8.96]	0.5%
O'Reilly_Uganda	6	250	2.40	[0.89; 5.15]	0.5%
Obienu_Nigeria	12	180	6.67	[3.49; 11.36]	0.5%
Ocama_Uganda	2	77	2.60	[0.32; 9.07]	0.4%
Odama_Nigeria	43	363	11.85	[8.71; 15.62]	0.5%
Ogwu−Richard_Nigeria	43	183	23.50	[17.56; 30.32]	0.5%
Ojide_Nigeria	24	342	7.02	[4.55; 10.26]	0.5%
Okeke_Nigeria	3	401	0.75	[0.15; 2.17]	0.5%
Okoth_Kenya	4	247	1.62	[0.44; 4.09]	0.5%
Okwuraiwe_Nigeria	346	10214	3.39	[3.05; 3.76]	0.5%
Olatunji_Nigeria	0	96	0.00	[0.00; 3.77]	0.5%
Olokoba_Nigeria	9	200	4.50	[2.08; 8.37]	0.5%
Omosigho_Nigeria	19	250	7.60	[4.64; 11.61]	0.5%
Oshitani_Zambia	1	182	0.55	[0.01; 3.02]	0.5%
Otegbayo_Nigeria	86	1779	4.83	[3.88; 5.94]	0.5%
Pappoe_Ghana	2	394	0.51	[0.06; 1.82]	0.5%
Patel_Botswana	2	252	0.79	[0.10; 2.84]	0.5%
Pawlotsky_Central African Republic	1	30	3.33	[0.08; 17.22]	0.3%
Peter_Nigeria	9	232	3.88	[1.79; 7.24]	0.5%
Pirillo_Rwanda	4	82	4.88	[1.34; 12.02]	0.4%
Pirillo_Uganda	9	165	5.45	[2.52; 10.10]	0.5%
Plamondon_Guinea−Bissau	4	252	1.59	[0.43; 4.01]	0.5%
Rabenau_Lesotho	1	205	0.49	[0.01; 2.69]	0.5%
Rebbani_Morocco	27	503	5.37	[3.57; 7.71]	0.5%
Rouet_Gabon	67	762	8.79	[6.88; 11.03]	0.5%
Rouet_Ivory Coast	2	280	0.71	[0.09; 2.56]	0.5%
Rouet_Ivory Coast	6	501	1.20	[0.44; 2.59]	0.5%
Rusine_Rwanda	23	402	5.72	[3.66; 8.46]	0.5%
Sadoh_Nigeria	8	155	5.16	[2.25; 9.92]	0.5%
Sagoe_Ghana	5	138	3.62	[1.19; 8.25]	0.5%
Salpini_Cameroon	51	212	24.06	[18.47; 30.39]	0.5%
Salu_Nigeria	15	393	3.82	[2.15; 6.22]	0.5%
Seremba_Uganda	26	194	13.40	[8.95; 19.02]	0.5%
Shimelis_Ethiopia	15	477	3.14	[1.77; 5.13]	0.5%
Simpore_Burkina Faso	10	207	4.83	[2.34; 8.70]	0.5%
Simpore_Burkina Faso	9	58	15.52	[7.35; 27.42]	0.4%
Simpore_Burkina Faso	8	108	7.41	[3.25; 14.07]	0.5%
Sonderup_South Africa	11	301	3.65	[1.84; 6.44]	0.5%
Soni_South Africa	0	20	0.00	[0.00; 16.84]	0.3%
Sutcliffe_Malawi	35	279	12.54	[8.90; 17.01]	0.5%
Tamo_Cameroon	10	75	13.33	[6.58; 23.16]	0.4%
Taye_Ethiopia	25	387	6.46	[4.22; 9.39]	0.5%
Telatela_Tanzania	23	167	13.77	[8.94; 19.95]	0.5%
Tess_Tanzania	1	22	4.55	[0.12; 22.84]	0.3%
Tessema_Ethiopia	4	239	1.67	[0.46; 4.23]	0.5%
Torpey_Ghana	23	4023	0.57	[0.36; 0.86]	0.5%
Tremeau−Bravard_Nigeria	10	443	2.26	[1.09; 4.11]	0.5%
Umutesi_Rwanda	5470	116868	4.68	[4.56; 4.80]	0.5%
Walusansa_Uganda	4	122	3.28	[0.90; 8.18]	0.5%
Wandeler_Mozambique	0	1057	0.00	[0.00; 0.35]	0.5%
Wandeler_Zambia	1	755	0.13	[0.00; 0.74]	0.5%
Wekesa_Uganda	33	1784	1.85	[1.28; 2.59]	0.5%
Wester_Botswana	0	50	0.00	[0.00; 7.11]	0.4%
Wondimeneh_Ethiopia	22	400	5.50	[3.48; 8.21]	0.5%
Xie_Equatorial Guinea	8	230	3.48	[1.51; 6.74]	0.5%
Ya'Aba_Nigeria	5	203	2.46	[0.80; 5.65]	0.5%
Ya’aba_Nigeria	28	1200	2.33	[1.56; 3.35]	0.5%
Ya’aba_Nigeria	26	554	4.69	[3.09; 6.80]	0.5%
Yakubu_Nigeria	37	180	20.56	[14.91; 27.20]	0.5%
Yendewa_Sierra Leone	0	47	0.00	[0.00; 7.55]	0.4%
Yendewa_Sierra Leone	0	49	0.00	[0.00; 7.25]	0.4%
Yendewa_Sierra Leone	2	88	2.27	[0.28; 7.97]	0.4%
Yendewa_Sierra Leone	9	211	4.27	[1.97; 7.94]	0.5%
Yimer_Ethiopia	20	851	2.35	[1.44; 3.61]	0.5%
Zeba_Burkina Faso	9	378	2.38	[1.09; 4.47]	0.5%
**Random effect meta−analysis**		**247701**	**5.65**	**[4.85; 6.50]**	**92.1%**
**Prediction interval**	**.**	**.**		**[0.00; 20.55]**	**−−**
Heterogeneity: I^2 = 98.1% [98.0%; 98.2%], τ^2 = 0.0130, p = 0					
**HCV_HCV RNA**					
Agwale_Nigeria	12	146	8.22	[4.32; 13.92]	0.5%
Andreotti_Malawi	1	309	0.32	[0.01; 1.79]	0.5%
Chakraborty_Kenya	0	60	0.00	[0.00; 5.96]	0.4%
Hønge_Guinea−Bissau	8	576	1.39	[0.60; 2.72]	0.5%
Jobarteh_Gambia	3	572	0.52	[0.11; 1.53]	0.5%
King_Ghana	4	408	0.98	[0.27; 2.49]	0.5%
Laurent_Cameroon	21	169	12.43	[7.86; 18.37]	0.5%
Musyoki_South Africa	26	34	76.47	[58.83; 89.25]	0.4%
Ndjomou_Cameroon	8	124	6.45	[2.83; 12.32]	0.5%
Simpore_Burkina Faso	0	58	0.00	[0.00; 6.16]	0.4%
Sonderup_South Africa	10	301	3.32	[1.60; 6.02]	0.5%
Torpey_Ghana	20	4023	0.50	[0.30; 0.77]	0.5%
Waddell_Tanzania	0	100	0.00	[0.00; 3.62]	0.5%
Yendewa_Sierra Leone	0	47	0.00	[0.00; 7.55]	0.4%
Yendewa_Sierra Leone	0	49	0.00	[0.00; 7.25]	0.4%
Yendewa_Sierra Leone	0	88	0.00	[0.00; 4.11]	0.4%
Zeba_Burkina Faso	9	378	2.38	[1.09; 4.47]	0.5%
**Random effect meta−analysis**		**7442**	**2.51**	**[0.96; 4.64]**	**7.9%**
**Prediction interval**	**.**	**.**		**[0.00; 15.14]**	**−−**
Heterogeneity: I^2 = 93.4% [90.9%; 95.2%], τ^2 = 0.0111, p < 0.0001					
**Overall random effect meta−analysis**		**255143**	**5.37**	**[4.62; 6.17]**	**100.0%**
**Prediction interval**				**[0.00; 20.19]**	
Heterogeneity: I^2 = 98.0% [97.9%; 98.2%], τ^2 = 0.0132, p = 0					
Test for subgroup differences: χ^2_1 = 6.78, df = 1 (p = 0.0092)					

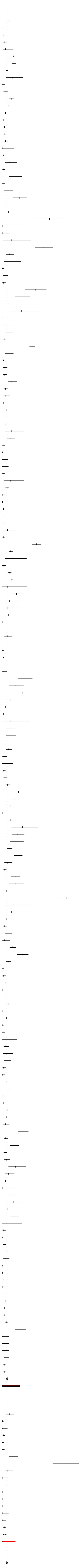

Supplement: S4 Fig — (PDF) [file pone.0269250.s013.pdf]
