# Supplementary material for: Epidemiology of hepatitis B virus and/or hepatitis C virus infections among people living with human immunodeficiency virus in Africa: A systematic review and meta-analysis
Source: PLoS One. 2022 May 31;17(5):e0269250. doi: 10.1371/journal.pone.0269250 (PMC9154112; doi:10.1371/journal.pone.0269250)
Supplement: Attachment — Submitted filename: Response to Reviewers.doc [file pone.0269250.s017.doc]

**Review Comments to the Author**

**Reviewer #1:** Thank you for a very good manuscript. This is very thorough and all expected aspects for the systematic review and metanalysis have been presented. I find very tittle to comment on because the limitations have been identified and discussed too.

Authors: We thank the reviewer for this appreciation.

1. the period for the review, dies it end 2021? Or it was 2022 as stated in the manuscript?

Authors: As indicated in the manuscript, the study ends in 2022. Indeed, our search strategy was first applied in the databases in February 2021. Due to the delay in finalizing the review we had to reapply our search strategy in January 2022 as indicated in the methodology section.

2. Why did you limit the language to English and French? These are not the only languages spoken officially in Africa. This limitation is not mentioned at all in the manuscript. Kindly address this.

Authors: We thank the reviewer for this suggestion. We added this limit in the discussion section.

3. There are few typographical errors which needs to be corrected so kindly go through

Authors: Thank you, we have carefully revised it.

4. In the discussion, I expected some recommendations concerning the quality of the publications.

Authors: Thank you for this suggestion. Half of the studies in our review were at moderate risk of bias. Our sensitivity analysis, that we added in the result section, showed that this risk of bias did not affect our overall results.

“Although about half of the studies had a moderate risk of bias, sensitivity analyzes showed no difference between the overall results and the results including only the studies with a low risk of bias (Table 1).”

**Reviewer #2:** Thank you for the opportunity to review this manuscript. It is well performed and the data is well presented. The finding are relevant and important.

Authors: Thank you for this appreciation.

1. The issue of epidemiology, notably HBV-HIV is addressed in the discussion. I may suggest to the authors that it makes somewhat more sense to introduce the issue of HBV being mostly acquired in childhood in sub Saharan Africa, with HIV later in adulthood. This is true for most with HIV-HBV co-infection and may well add to the burden of liver disease. The reasons as to why the HBV rate is somewhat higher in the co-infected population, perhaps warrants some more discussion, although understandably the reasons are not entirely clear.

Authors: Thank you for this comment which we fully agree. We have added the comment below in the discussion to support this aspect.

“Although this specific transmission pattern in Africa of HBV and HIV in childhood and adulthood respectively is true for most cases, it is evident that many other reasons not fully described and/or clear exist and warrant more attention.”

2. The authors comments on HBV DNA presence in co-infection as a metric [Twelve studies reported the HBV infection prevalence based on the detection of HBV DNA; 17.1% [95% CI=11.4-23.7]. I assume these are ART naive patients in these studies - as most ART would likely contain if not lamivudine, but tenofovir ?

Authors: Thank you for the comment for which we fully agree. After verification, 4 of the studies focus obviously on ART naive patients, 2 studies have a mixture of naive and on ART subjects, and for the remaining 6 studies the ART status of the patients is unclear and/or not reported.

3. Would there be any value looking at HIV-HCV in sub-populations e.g. PWID in the manuscript?

Authors: thank you for this suggestion. We added a paragraph in the discussion section about this.
